# Sultones and Sultines via a Julia–Kocienski Reaction of Epoxides

**DOI:** 10.1002/anie.201508467

**Published:** 2015-10-27

**Authors:** Geoffrey M T Smith, Paul M Burton, Christopher D Bray

**Affiliations:** Department of Chemistry, Queen Mary University of London Mile End Road, London, E1 4NS (UK) E-mail: c.bray@qmul.ac.uk; Syngenta, Jealott's Hill International Research Centre Bracknell, Berkshire, RG42 6EY (UK)

**Keywords:** anion-relay, anions, spiro compounds, sulfur heterocycles, synthetic methods

## Abstract

The development of the homologous Julia–Kocienski reaction has led to the discovery of two new reaction modes of epoxides with sulfones. These pathways allow rapid and direct access to a range of γ-sultones and γ-sultines.

Sulfur containing heterocycles play a major role in the pharmaceutical, agrochemical, materials and petrochemical industries. First made over 125 years ago,[1] sultones and sultines (the thia-analogues of lactones) (Figure 1) are among the oldest known sulfur heterocycles.[2] Sultines react akin to their carbocyclic cousins and undergo nucleophilic substitution at sulfur. Sultines are used as lactone bioisosteres,[3] occur as natural products,[4] find use in the perfume industry[5] and are characterized as one of the distinctive olfactants of Sauternes wines.[6] In contrast, ring-opening of sultones occurs with cleavage of the C–O, rather than S–O bond. Sultones act as sulfoxylating agents[7] and are important in a wide variety of fields including soap manufacture, drug discovery,[8] polymer modification,[9] imaging[10] and energy storage.[11] A myriad of synthetic methodologies involve sultones and sultines as key intermediates[12] and they have been used in many total syntheses.[13]

**Figure 1:**
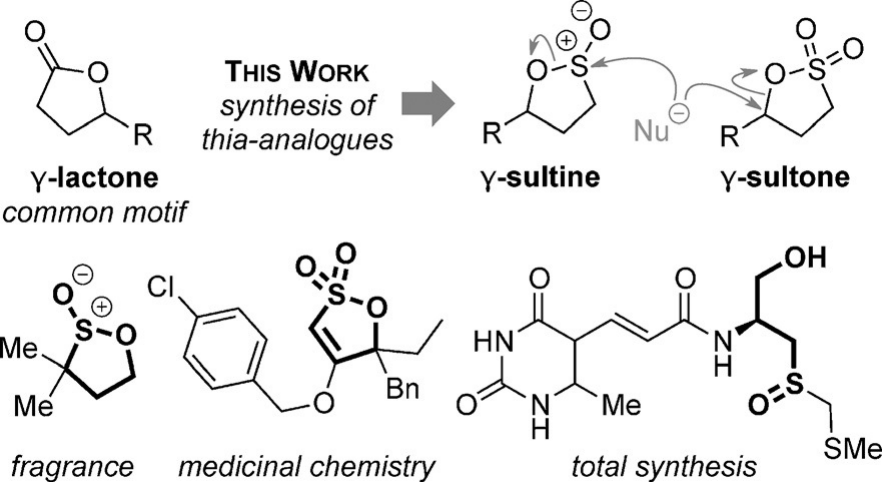
Reactivity profile and selected uses of sultones and sultines.

Despite their usefulness, synthesis of these fundamental heterocycles is surprisingly difficult, especially given how long they have been known. There are some notable routes to β- and δ-sultones,[14] but existing routes to γ-sultones and to sultines in general are invariably lengthy and low yielding,[12] produce racemates,[15] utilize chiral auxiliaries[16] or are based on chiral pool approaches.[13a, 17] Herein, we present two new reaction modes of epoxides, that leads either to γ-sultones or to γ-sultines in one or two steps respectively (Figure 2).[18]

**Figure 2:**
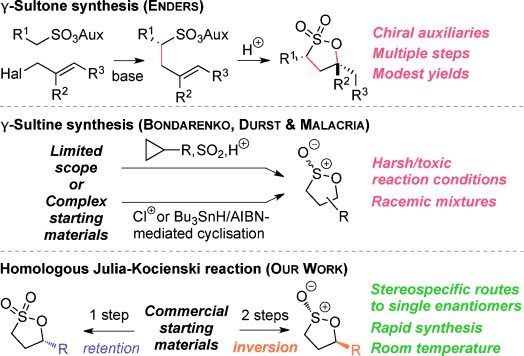
Current leading routes to sultones and sultines versus our work.

The Julia–Kocienski reaction of aldehydes and ketones is one of the preeminent methods for the stereocontrolled synthesis of alkenes.[19] Related to our interest in three-membered rings,[20] we wondered whether a homologous variant of this reaction might be developed. Epoxides were the first electrophiles considered since they are readily available as single enantiomers via a multitude of methods.[21] It was thought that a Julia–Kocienski sulfone for example, **1 a** (Scheme 1) could ring-open an epoxide (**2**) to give a γ-alkoxysulfone **3**. Anion-relay[22] (Smiles rearrangement)[23] of **3** would give a sulfinate for example, **6**, whose fate was unclear but by analogy with the known phosphorus chemistry[24] we anticipated loss of SO_2_[25] and cyclization to create a new general method for cyclopropane synthesis.

**Scheme 1:**
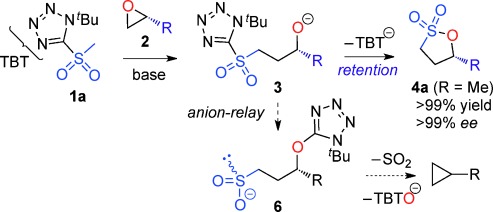
A new reaction mode of epoxides with sulfones to give γ-sultones.

Evaluation of a series of epoxide, sulfone, base, temperature, solvent and Lewis-acid combinations ultimately led us to react TBTSO_2_Me[26] **1 a** and (*R*)-propylene oxide **2 a** (R=Me) with LiN(SiMe_3_)_2_ as base at room temperature. However, rather than a cyclopropane, this gave the γ-sultone (*R*)-**4 a** (R=Me) in >99 % yield and *ee*.[27] This indicates the initially formed γ-alkoxysulfone **3** (R=Me) did not undergo the expected anion-relay, but instead directly cyclized on sulfur with loss of the tetrazolide as a leaving group.[28] As a result, the stereochemistry of the original epoxide is completely retained in the γ-sultone product. An operationally simple one-step synthesis of γ-sultones is therefore achieved. A range of epoxides were then examined to determine the scope of this new process (Table 1). A variety of alkyl substituted (enantiopure)[21] terminal epoxides reacted with **1 a** to give the γ-sultones **4 b**–**i** in good to excellent yields. Substrates bearing a protected alcohol, amine and ketone as well as containing a halogen were converted to the corresponding γ-sultones **4 j**–**m**. A *bis*-epoxide was examined as a substrate, but even with five equiv of base/sulfone only the *mono*-sultonylated product **4 n** was obtained, which suggests that formation of the first sultone ring retards that of the second. The γ-sultones **4 f**–**n** are of interest since they could allow for further functionalization and demonstrate the functional group tolerance of this process. Curiously, styrene oxide simply returned the starting materials. We next examined disubstituted epoxides; *cis*-1,2-epoxybutane gave solely *trans*-sultone **4 o** whereas *trans*-1,2-epoxybutane, gave solely the *cis*-sultone **4 p**, clear evidence for the proposed reaction pathway involving a single inversion of stereochemistry and revealing increased steric bulk on the epoxide could be tolerated. In view of the widespread interest from discovery chemists in spirocycles[29] we extended this study to 1,1-dialkyl-substituted epoxides, and pleasingly, we were able to synthesize the sultones **4 q**–**v**.

**Table 1:** One-step synthesis of γ-sultones from epoxides

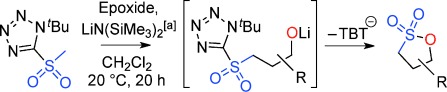
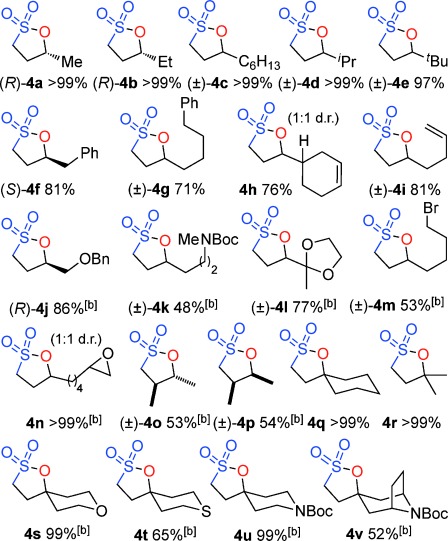

[a] 1 Equiv of base/sulfone unless otherwise stated. [b] 3 Equiv of base/sulfone.

Our inability to form sultones from arylepoxides was intriguing. We therefore synthesized the γ-hydroxysulfone **5 a** (Scheme 2) by BF_3_-mediated ring-opening of (*S*)-styrene oxide with sulfone **1 a** with KN(SiMe_3_)_2_ as base (76 % yield).[27] This intermediate was treated with a variety of bases. Upon treatment with DBU, the product was neither a cyclopropane nor even a sultone, but instead the γ-sultines **7 a** (*trans*:*cis* 76:24) in 76 % yield and >99 % *ee*.[27] This indicates a second reaction pathway where anion-relay had occurred but, rather than loss of SO_2,_ the sulfinate **6 a** had directly cyclized through oxygen. This is unusual since sulfinates primarily alkylate on sulfur and rarely on oxygen.[30] No γ-aryl-γ-sultone products were ever detected from this reaction but the γ-sultines **7 a** could be oxidized (to **4 w**) or alternatively photolysed to give cyclopropylbenzene,[31] one of the products originally mooted by us for this process. In this instance, the stereocentre of the epoxide has been inverted and this pathway is stereodivergent from the one that produces γ-sultones.[32]

**Scheme 2:**
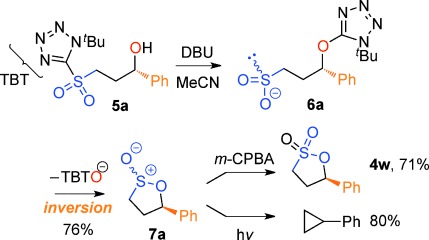
An alternative reaction pathway to give γ-sultines.

To uncover further examples of γ-sultine formation using the homologous Julia-Kocienski reaction we examined the reactions of other γ-hydroxysulfones **5** with DBU (Table 2). γ-Sultine products **7 b** were isolated from the 2-naphthyloxirane derived substrate **5 b**. In a similar manner, the vinyl substituted γ-sultines **7 c** could be observed through in situ NMR monitoring, though they could not be isolated. The unstable sulfinic acid **8** (entry 4) which results from **5 d** via anion-relay/protonation could also be observed but even under forcing conditions it did not cyclize to a γ-sultine.

**Table 2:** Synthesis of γ-sultines through anion-relay/cyclization

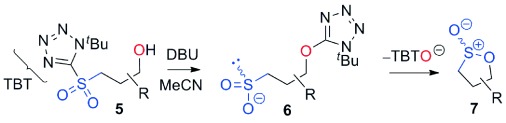					
Entry	γ-Hydroxysulfone		Product(s)		Yield [%]^[a]^
1	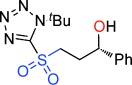	**5 a**	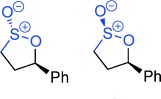	**7 a** (76:24)	76
2	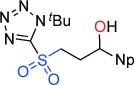	**5 b**	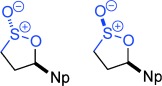	**7 b** (2:1)	51
3	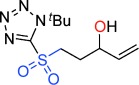	**5 c**	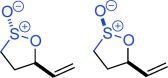	**7 c** (1:1)	>99^[b]^
4	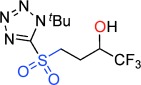	**5 d**	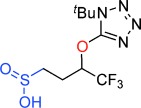	**8**	>99^[b]^
5	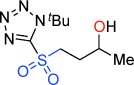	**5 e**	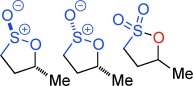	**7 e** (69:25:6)	>99^[b,c]^
6	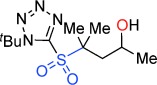	**5 f**	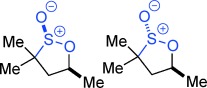	**7 f** (1:1)	21
7	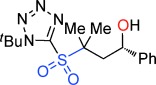	**5 g**	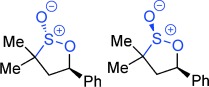	**7 g** (17:83)	47
8	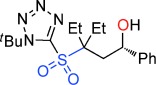	**5 h**	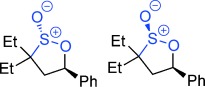	**7 h** (23:77)	45
9	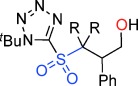	**5 i**, R=Me	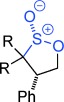	**7 i**	65
10		**5 j**, R=Et		**7 j**	62

[a] Isolated yield. [b] Conversion as judged by ^1^H NMR spectroscopy. [c] Isolated as a complex mixture alongside TBT-derived by-products.

The two reaction pathways that produce γ-sultones and γ-sultines proceed via similar γ-alkoxysulfone intermediates (**3**). Collectively, the results thus far suggest that when these have been formed using a lithium amide base and are substituted with an alkyl group (i.e. for all compounds in Table 1), the oxy-anion is sufficiently nucleophilic to directly displace a tetrazolide as leaving group and give γ-sultones as products. Conversely, when they are formed from γ-hydroxysulfones (**5**) with DBU and bear softer/less electron rich (withdrawing) groups for example, Ph, vinyl (i.e. those in Table 2), these are unable to directly attack on sulfur and instead reaction is diverted along the pathway involving anion-relay to give γ-sultines. We hypothesized that use of the propylene oxide derived substrate **5 e** would provide one of the most evenly balanced scenarios between these two situations. Treatment of this compound with DBU in MeCN gave an inseparable mixture of the sultines **7 e** alongside traces of the sultone **4 a** (Table 2, entry 5). In contrast treatment of **5 e** with LiN(SiMe_3_)_2_ gave solely the sultone **4 a** (68 % isolated yield). This demonstrates that the two reaction pathways can compete, however use of Et-, ^*i*^Pr- or ^*t*^Bu-substituted γ-hydroxysulfones **5** led exclusively to the sultones **4 b**,**d**,**e** in >90 % yield in each case irrespective of the base used. This reveals how delicately balanced the stereoelectronics are for the two pathways. We next reasoned that despite the Thorpe–Ingold effect, increased substitution adjacent to sulfur would disfavor direct ring-closure as it would be pseudo neopentyl and might lead exclusively to γ-sultine formation. This was indeed found to be the case and the γ-alkoxysulfone **5 f** derived from ring-opening of propylene oxide with TBTSO_2_^*i*^Pr **1 b** gave clean, albeit slow, conversion to γ-sultine products on treatment with DBU (as judged by ^1^H NMR spectroscopy), though the labile sultines **7 f** were only isolated in 21 % yield. Cyclization of the styrene oxide derived γ-alkoxysulfones **5 g** and **5 h** proceeded with greater ease indicating that cyclization at a benzylic position was more favourable. Both diastereomers of **7 g** were suitable for X-ray crystallographic analysis. Of note was the fact that in each case, the S=O bonds were oriented pseudo-axial,[33] forcing one of the methyl substituents into a seemingly unfavorable position. This unusual observation can be rationalized by the presence of an anomeric-like effect, a phenomenon which has been proposed previously for sultines.[34] Finally, the regioisomeric substrates **5 i** and **5 j** underwent smooth cyclization to the sultines **7 i** and **7 j** demonstrating that substitution in the β-position was possible and that the need for an electron withdrawing substituent at the γ-position can be avoided if direct attack on sulfur can be prevented. Sultines **7 i** and **7 j** being isolated solely as the *cis*-diastereomers indicated high levels of 1,3-stereocontrol for this substitution pathway.

In conclusion we present the first examples of a homologous Julia–Kocienski reaction which reveals two mechanistically novel reaction pathways of epoxides with sulfones. These two pathways provide access to γ-sultones and γ-sultines in very rapid one and two-pot processes in a stereocontrolled manner, something which existing methods fail to achieve for either heterocycle. Sulfur containing heterocycles are prevalent in drugs[35] and there is a current desire within discovery chemistry to introduce saturated chiral scaffolds,[36] as it is seen as a way to improve clinical success. Sultines and sultones represent an interesting and largely unexplored area of chemical space which is ripe for exploration given that ready access to them is now possible.

## Supplementary material

As a service to our authors and readers, this journal provides supporting information supplied by the authors. Such materials are peer reviewed and may be re-organized for online delivery, but are not copy-edited or typeset. Technical support issues arising from supporting information (other than missing files) should be addressed to the authors.
