# Supplementary material for: Sultones and Sultines via a Julia–Kocienski Reaction of Epoxides
Source: Angew Chem Int Ed Engl. 2015 Oct 27;54(50):15236–40. doi: 10.1002/anie.201508467 (PMC4691334; doi:10.1002/anie.201508467)
Supplement: Supplementary file 1 [file anie0054-15236-SD1.pdf]

Angewandte Chemie
Eine Zeitschrift der Gesellschaft Deutscher Chemiker

Supporting Information

Sultones and Sultines via a Julia–Kocienski Reaction of Epoxides

*Geoffrey M. T. Smith, Paul M. Burton, and Christopher D. Bray**

anie_201508467_sm_miscellaneous_information.pdf

-Supporting Information-

Contents

1. General Experimental Details **S2**

2. Experimental procedures and spectral data for heteroarylalkylsulfones **1** **S2**

3. Experimental procedures and spectral data for sultones **4a-w** **S3**

4. Experimental procedures and spectral data for γ-hydroxysulfones **5a-j** **S9**

5. Experimental procedures and spectral data for sultines **7a-j** **S11**

6. Spectral data for reaction by-products **S15**

7. ^{1}H and ^{13}C NMR data for heteroarylalkylsulfones **1a-c** **S16**

8. ^{1}H and ^{13}C NMR data for sultones **4a-v** **S20**

9. ^{1}H and ^{13}C NMR data for γ-hydroxysulfones **5a-j** **S42**

10. ^{1}H and ^{13}C NMR data for sultines **7a-j** **S51**

11. ^{1}H and ^{13}C NMR data for reaction by-products **S63**

12. X-ray crystallographic data **S65**

1. General Experimental Details

Commercially available reagents were used as received without further purification. All reactions requiring anhydrous conditions were conducted in flame-dried apparatus under an atmosphere of argon. Dry dichloromethane (DCM) and tetrahydrofuran (THF) were obtained by passing commercially pre-dried formulations through activated alumina columns. Analytical thin-layer chromatography (TLC) was performed on silica gel plates (0.25mm) precoated with a fluorescent indicator. Standard flash chromatography procedures were performed using Kieselgel 60 (40-63 µm). Petrol refers to the fraction boiling between 40–60 °C. Brine refers to a saturated aqueous solution of NaCl. Preparative HPLC was performed on a Waters Fraction Lynx system comprising a 2767 injector/collector with a 2545 gradient pump, two 515 isocratic pumps, SFO, 2998 photodiode array, 2424 ELSD and a 3100 mass spectrometer. Infrared spectra were recorded directly as neat liquids or solids on a Bruker Tensor 37 FTIR machine fitted with a PIKE MIRacle ATR accessory. ^{1}H and ^{13}C NMR spectra were recorded in $CDCl_3$ at 400 and 100 MHz respectively on Bruker AV400 machines. Chemical shifts (δ) are reported using the residual solvent resonance as the internal standard ($CDCl_3$: δ_H 7.26; δ_C 77.2). ^{1}H NMR data are reported as follows: chemical shift (δ), multiplicity (s = singlet, d = doublet, t = triplet, q = quartet, sep = septet, m = multiplet, dd = doublet of doublets, ddd = doublet of doublets of doublets, ddq = doublet of doublet of quartets, dddd = doublet of doublets of doublets of doublets, tt = triplet of triplets), coupling constants (Hz) and integration. ^{1}H NMR signals were assigned using standard 2D NMR techniques. High resolution mass spectra were obtained from the EPSRC NMSF located at Swansea University. X-ray crystallography was performed both at Queen Mary University of London and by the EPSRC NCS service located at Southampton University.

2. Experimental procedures and spectral data for heteroarylalkylsulfones 1:

1-(*tert*-Butyl)-1*H*-tetrazole-5-thiol (TBTSH) S1

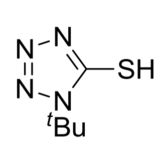

Prepared according to the procedure reported by Aïssa[1] using NaN_3[2] (14.20 g, 217 mmol), ^{t}BuNCS (25.00 g, 217 mmol) in H_2O (33 ml) and ^{i}PrOH (62 ml). The title compound **S1** was isolated as a pale yellow crystalline powder (34.30 g, 99 %); mp 96–97 °C (lit.[3] 97–98 °C); ^{1}H NMR (400 MHz, $CDCl_3$) δ 1.85 (s, 9H); ^{13}C NMR (101 MHz, $CDCl_3$) δ 162.9, 63.6, 27.6. Spectral data were in accordance with those in the literature.[1]

1-(*tert*-Butyl)-5-(methylsulfonyl)-1*H*-tetrazole (TBTSO$_2$Me) 1a

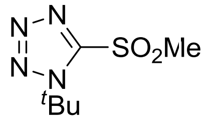

1-(*tert*-Butyl)-5-(methylsulfonyl)-1*H*-tetrazole was prepared according to the procedure reported by Aïssa1 using TBTSH (10 g, 63.2 mmol), MeI (3.93 ml, 63.2 mmol) and NaH (2.27 g, 60% dispersion in mineral oil) in THF (100 ml); TBTSMe (10.8 g, 63.2 mmol), $Mo_7O_{24}(NH_4)_6$ (7.78 g, 6.3 mmol), H_2O_2 (25 ml, 30% solution) in EtOH (25 ml). The title compound **1a** was isolated as a white crystalline powder (12.80 g, 99%); mp = 84–85.5 °C (lit.[1] 83–87 °C); IR (neat) ν_{max}/cm^{-1} = 3009, 2925, 1789, 1764, 1573, 1468, 1413, 1371, 1332, 1277, 1247, 1237, 1202, 1163, 1124, 1112, 1078, 1023, 1010; ^{1}H NMR (400 MHz, $CDCl_3$) δ 3.66 (s, 3H), 1.86 (s, 9H); ^{13}C NMR (100 MHz, $CDCl_3$) δ 154.6, 65.5, 44.7, 29.67; HRMS (ESI) calc'd for $C_6H_{13}N_4O_2S$ $[M+H]^+$ 205.0754; found 205.0754.

1-(*tert*-Butyl)-5-(isopropylsulfonyl)-1*H*-tetrazole (TBTSO$_2$^{i}Pr) 1b

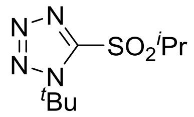

To a solution of TBTSH (3.00 g, 18.9 mmol) and ^{i}PrI (1.89 ml, 18.9 mmol) in dry THF (30 ml) was added NaH (1.37 g, 60% dispersion in mineral oil, 34.0 mmol) at 0 °C under an Ar atmosphere. The mixture was left to stir at 20–25 °C for 20 h, after which time the reaction mixture was concentrated *in vacuo*. The reaction was quenched by careful dropwise addition of water (5 ml) followed by HCl solution (1 M, 50 ml). The reaction mixture was extracted with EtOAc (50 ml). After separation, the aqueous phase was further extracted with EtOAc (20 ml) and the combined organic phases were successively washed with water (20 ml) and brine (20ml), dried over $MgSO_4$ and the solvent removed *in vacuo*. The residue (3.68 g) thus obtained was diluted in EtOH (30 mL), and cooled to 0 °C before $Mo_7O_{24}(NH_4)_6$ (2.26 g, 1.82 mmol) was added followed by dropwise addition of H_2O_2 (14 mL, 30% solution). After stirring at 20 °C for 20h, the bulk of the EtOH was removed *in vacuo*. The mixture was then diluted in CH_2Cl_2 (50 ml) and water (50 ml). The separated organic layer was washed with brine (50 ml), dried over $MgSO_4$, filtered and the solvent removed *in vacuo*. The *title compound* was

(1) C. Aïssa, *J. Org. Chem.* **2006,** *71,* 360.
(2) TBTSH is also commercially available.
(3) P. J. Kocienski, A. Bell, P. R. Blakemore, *Synlett* **2000**, 365.

isolated as white crystals (4.20 g, 95%); mp = 61–63 °C; IR (neat) ν_{max}/cm^{-1} = 2994, 2945, 1465, 1409, 1392, 1371, 1329, 1290, 1272, 1255, 1244, 1205, 1168, 1149, 1119, 1072, 1057, 1022; ^{1}H NMR (400 MHz, $CDCl_3$) δ 4.28 (sept, *J* = 6.8 Hz, 1H), 1.87 (s, 9H), 1.54 (d, *J* = 6.8 Hz, 6H); ^{13}C NMR (100 MHz, $CDCl_3$) δ 153.3, 65.6, 57.3, 29.8, 15.5; HRMS (ESI) calc'd for $C_8H_{17}N_4O_2S$ [M+H]$^+$ 233.1067; found 233.1068.

1-(*tert*-Butyl)-5-(pentan-3-ylsulfonyl)-1*H*-tetrazole (TBTSO$_2$CHEt$_2$) 1c

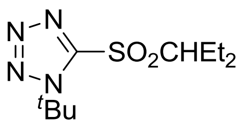

To a solution of TBTSH (5.00 g, 32.0 mmol) in ethanol (50 ml) was added KOH (1.79 g, 32.0 mmol) and the mixture heated to reflux. After 30 min, ^{n}PrBr (3.16 ml, 34.8 mmol) was added and the reaction was held at reflux for 16 h after which time the reaction mixture was concentrated *in vacuo*. The residue was diluted in EtOAc (100 ml) and H_2O (100 ml). After separation, the aqueous phase was further extracted with EtOAc (30 ml) and the combined organic phases were successively washed with water (50 ml) and brine (50ml), dried over $MgSO_4$ and the solvent removed *in vacuo*. The residue (6.21 g) thus obtained was diluted in EtOH (100 mL), and cooled to 0 °C before $Mo_7O_{24}(NH_4)_6$ (3.83 g, 3.10 mmol) was added followed by dropwise addition of H_2O_2 (30 mL, 30% solution). After stirring at 20 °C for 5h, the bulk of the EtOH was removed *in vacuo*. The mixture was then diluted in CH_2Cl_2 (100 ml) and water (100 ml). The separated organic layer was washed with brine (100 ml), dried over $MgSO_4$, filtered and the solvent removed *in vacuo* to give a colourless oil (7.20 g). This oil was diluted in THF (30 ml) and cooled to –78 °C before a solution of $LiN(SiMe_3)_2$ (31 ml, 1 M in hexanes, 31 mmol) was added dropwise and then left to stir for 30 min. After this time, EtBr (2.52 ml, 34.0 mmol) was added and the mixture was warmed to 20 °C over 6 h. The reaction was quenched by the addition of 1M HCl (100 ml) and diluted with EtOAc (100 ml) before being separated. The aqueous phase was further extracted with EtOAc (30 ml) and the combined organic phases were successively washed with water (50 ml) and brine (50 ml), dried over $MgSO_4$, filtered and the solvent removed *in vacuo.* The residue was purified by flash column chromatography (30% Et_2O/petrol) to give the *title compound* **1c** as clear oil (3.51 g, 40% over 3 steps); IR (neat) ν_{max}/ cm^{-1} = 2977, 2941, 2882, 1462, 1407, 1376, 1323, 1240, 1207, 1158, 1118, 1073, 1043, 1022; ^{1}H NMR (400 MHz, $CDCl_3$) δ 4.01 (tt, *J* = 7.1 & 4.7 Hz, 1H), 2.03 (m, 2H), 1.95 (m, 2H), 1.86 (s, 9H), 1.13 (t, *J* = 7.4 Hz, 6H); ^{13}C NMR (100 MHz, $CDCl_3$) δ 154.1, 67.6, 65.6, 29.8, 20.5, 11.1; HRMS (ESI) calc'd for $C_{10}H_{21}N_4O_2S$ [M+H]$^+$ 261.1380; found 261.1377.

3. Experimental Procedures for Sultones 4a-v:

General Procedure A: The formation of sultones from epoxides with $LiN(SiMe_3)_2$ in CH_2Cl_2 (1 eq of sulfone).

To a solution of TBTSO$_2$Me **1a** (204 mg, 1.00 mmol) in dry CH_2Cl_2 (3 ml), the corresponding epoxide (1.10 mmol) was added and stirred at 20–25 °C. To this, $LiN(SiMe_3)_2$ (1.10 ml, 1.0 M in hexanes, 1.10 mmol) was added dropwise turning the solution yellow. The mixture was left to stir at 20–25 °C for 20 h, after which time a white precipitate formed. The reaction was poured into sat. aq. NH_4Cl solution (15 ml) and extracted with EtOAc (2 × 15 ml). The combined organic phases were washed with brine (15 ml), dried over $MgSO_4$, filtered and concentrated *in vacuo* to give a residue which was purified by flash column chromatography as described for each entry to yield the title compounds.

General Procedure B: The formation of sultones from epoxides with $LiN(SiMe_3)_2$ in CH_2Cl_2 (3 eq of sulfone).

To a solution of TBTSO$_2$Me **1a** (612 mg, 3.00 mmol) in dry CH_2Cl_2 (9 ml), the corresponding epoxide (1.00 mmol) was added and stirred at 20–25 °C. To this, $LiN(SiMe_3)_2$ (1.0 M in hexanes, 3.30 ml, 3.30 mmol) was added dropwise turning the solution yellow. The mixture was left to stir at 20–25 °C for 20 h, after which time a white precipitate formed. The reaction was poured into sat. aq. NH_4Cl solution (30 ml) and extracted with EtOAc (2 × 30 ml). The combined organic phases were washed with brine (30 ml), dried over $MgSO_4$, filtered and concentrated *in vacuo* to give a residue which was purified by flash column chromatography as described for each entry to yield the title compounds.

Spectral Data for Sultones 4a-v:

(*R*)-5-Methyl-1,2-oxathiolane 2,2-dioxide (4a).

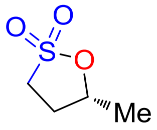

According to General Procedure A, commercial (*R*)-propylene oxide (77 µl, 1.10 mmol) gave following flash column chromatography (SiO_2, 60% Et_2O/petrol) the *title compound* **4a** (135 mg, >99%) as clear oil; IR (neat) ν_{max}/cm^{-1} = 2924, 1452, 1421, 1336, 1270, 1270, 1197, 1154, 1028;

^{1}H NMR (400 MHz, $CDCl_3$) δ 4.82–4.73 (m, 1H), 3.35 (dddd, *J* = 13.2, 8.9, 4.3 & 0.5 Hz, 1H), 3.29 – 3.22 (m, 1H); 2.63 (dddd, *J* = 13.4, 6.1, 5.9 & 4.3 Hz, 1H), 2.28 (dddd, *J* = 13.4, 9.1, 9.0, 8.9 Hz, 1H), 1.52 (d, *J* = 6.1 Hz, 3H); ^{13}C NMR (100 MHz, $CDCl_3$) δ 79.2, 46.0, 31.3, 20.8; HRMS (ESI) calc'd for $C_4H_{12}O_3N_1S_1$ $[M + NH_4]^+$ 154.0532; found 154.0530; $[\alpha]_D^{25}$ +1.42 (*c* 0.7, CH_2Cl_2); Enantiomeric excess[4] >99% by HPLC (Daicel IC column, ^{i}PrOH/hexane = 10/90, 30 °C, flow rate 1.0 ml/min, λ = 212 nm); t_R mj = 13.87 min, t_R mn = 15.17 min.

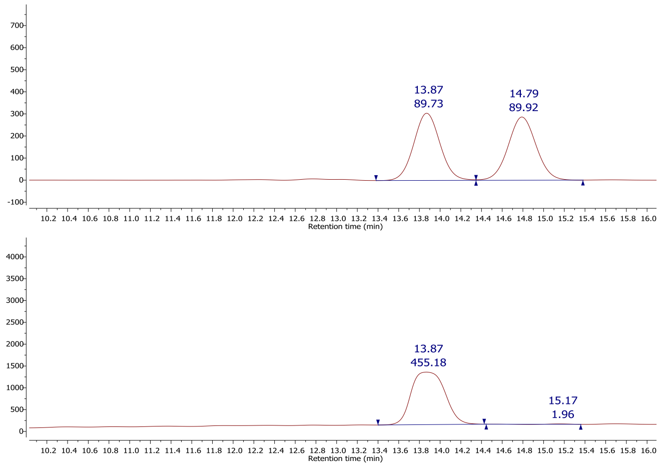

(±)-α,α-dibenzyl **4a**	
Peak	Area
13.87	89.73
14.79	89.92

(*R*)-α,α-dibenzyl **4a**	
Peak	Area
13.87	455.18
15.17	1.96

(*R*)-5-Ethyl-1,2-oxathiolane 2,2-dioxide (4b).

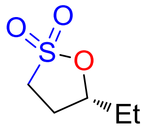

According to General Procedure A, commercial (*R*)-1,2-epoxybutane (95 µl, 1.10 mmol) gave, following flash column chromatography (SiO_2, 60% Et_2O/petrol) the *title compound* **4b** as clear oil (150 mg, >99%); IR (neat) ν_{max}/cm^{-1} = 2974, 1962, 1692, 1456, 1338, 1274, 1194, 1155, 1049; ^{1}H NMR (400 MHz, $CDCl_3$) δ 4.63–4.52 (m, 1H), 3.36–3.29 (m, 1H), 3.26 (ddd, *J* = 13.1, 7.9, 6.6 Hz, 1H), 2.59 (dddd, *J* = 13.3, 7.5, 6.0, 4.5 Hz, 1H), 2.30 (dddd, *J* = 13.3 , 9.4, 9.2 & 8.8 Hz, 1H), 1.96–1.82 (m, 1H), 1.76 (ddq, *J* = 14.7, 7.5 & 5.3 Hz, 1H), 1.04 (t, *J* = 7.5 Hz, 3H); ^{13}C NMR (100 MHz, $CDCl_3$) δ 83.7, 45.6, 29.1, 28.3, 9.4; HRMS (ESI) calc'd for $C_5H_{14}O_3N_1S_1$ $[M + NH_4]^+$ 168.0689; found 168.0687; $[\alpha]_D^{25}$ +16.5 (*c* 0.43, CH_2Cl_2); Enantiomeric excess4 >99% via HPLC (Daicel IC column, ^{i}PrOH/hexane = 15/85, 30 °C, flow rate 1.0 ml/min, λ = 212 nm) t_R mj = 11.87 min, t_R mn = 12.60 min.

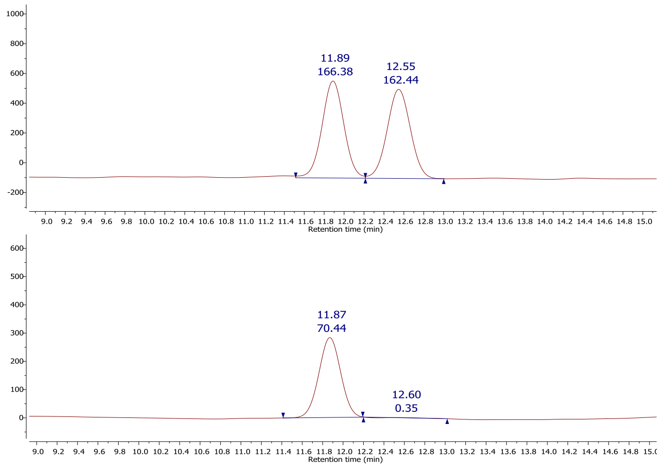

(±)-α,α-dibenzyl **4b**	
Peak	Area
11.89	166.38
12.55	162.44

(*R*)-α,α-dibenzyl **4b**	
Peak	Area
11.87	70.44
12.60	0.35

5-Hexyl-1,2-oxathiolane 2,2-dioxide (4c).

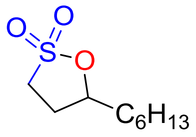

According to General Procedure A, 1,2-epoxyoctane (169 µl, 1.10 mmol) gave, following flash column chromatography (SiO_2, 35% Et_2O/petrol), the *title compound* **4c** as clear oil (205 mg, >99%); IR (neat) ν_{max}/cm^{-1} = 2955, 2928, 2858, 2211, 1459, 1421, 1343, 1270, 1190, 1157, 1066, 1001; ^{1}H NMR (400 MHz, $CDCl_3$) δ 4.67–4.57 (m, 1H), 3.31 (ddd, *J* = 13.2, 9.3, 4.3, Hz, 1H), 3.26 (m, 1H), 2.63–2.55 (m, 1H), 2.29 (dddd, *J* = 13.3, 9.1, 9.1, 9.0Hz, 1H), 1.93–1.81 (m, 1H), 1.74–1.63 (m, 1H), 1.54–1.30 (m, 8H), 0.89 (t, *J* = 6.9, 3H); ^{13}C NMR (100 MHz, $CDCl_3$) δ 82.9, 45.8, 35.3, 31.7, 29.7, 28.9, 25.2, 22.6, 14.1; HRMS calc'd for $C_9H_{22}O_3S_1N_1$ $[M + NH_4]^+$ 224.1315; found 224.1316.

(4) *In lieu* of a chromophore, sultones **4a and 4b** were α,α-dibenzylated using ^{n}BuLi and BnBr prior to analysis by chiral HPLC, see: D. Enders, W. Harnying, N. Vignola, *Eur. J. Org. Chem.* **2003**, 3939.

5-*iso*-Propyl-1,2-oxathiolane 2,2-dioxide (4d).

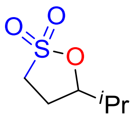

According to General Procedure A, 1,2-epoxy-3-methylbutane (117 µl, 1.10 mmol) gave, following flash column chromatography (SiO_2, 40% Et_2O/petrol), the *title compound* **4d** as a white solid (163 mg, >99%); mp 75–76 °C; ^{1}H NMR (400 MHz, $CDCl_3$) δ 4.34 (ddd, *J* = 9.2, 7.2 & 6.1 Hz, 1H), 3.35–3.10 (m, 2H), 2.53 (dddd, *J* = 13.3, 8.0, 6.0 & 4.1 Hz, 1H), 2.35 (ddd, *J* = 18.5, 13.3 & 9.3 Hz, 1H), 1.96–1.86 (m, 1H), 1.06 (d, *J* = 6.8 Hz, 3H), 0.98 (d, *J*= 6.9, 3H); ^{13}C NMR (100 MHz, $CDCl_3$) δ 87.3, 46.1, 33.0, 27.4, 18.4, 17.4; IR (neat) v_{max}/cm^{-1} = 2967, 2933, 2879, 1551, 1470, 1341, 1268, 1157; HRMS (EI) calc'd for $C_6H_{13}O_3S_1$ $[M + H]^+$ 165.0580; found 165.0575.

5-(*tert*-Butyl)-1,2-oxathiolane 2,2-dioxide (4e).

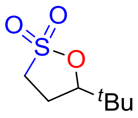

According to General Procedure A, 1,2-epoxy-3,3-dimethylbutane (135 µl, 1.10 mmol) gave, following flash column chromatography (SiO_2, 30% Et_2O/petrol), the *title compound* **4e** as white powder (179 mg, 97%); mp 78–80 °C; IR (neat) v_{max}/cm^{-1} = 2962, 2874, 1733, 1477, 1422, 1399, 1368, 1333, 1302, 1261, 1193, 1157, 1091, 1040, 1017; ^{1}H NMR (400 MHz, $CDCl_3$) δ 4.33 (dd, *J* = 8.3 & 7.5 Hz, 1H), 3.41–3.17 (m, 2H), 2.49–2.34 (m, 2H), 1.00 (s, 9H); ^{13}C NMR (100 MHz, $CDCl_3$) δ 89.8, 46.3, 34.2, 25.0, 24.9; HRMS (ESI) calc'd for $C_7H_{18}O_3S_1N_4$ $[M + NH_4]^+$ 196.1002; found 196.1002. This compound was further characterised by x-ray crystallographic analysis. See section 12 (S65).

(*S*)-5-Benzyl-1,2-oxathiolane 2,2-dioxide (4f).

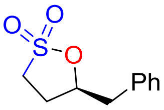

According to General Procedure A, (*S*)-2-benzyl oxirane[5] (134 mg, 1.10 mmol) gave, following flash column chromatography (SiO_2, 30% Et_2O/petrol), the *title compound* **4f** as colourless needles (171 mg, 81%); mp = 74–75 °C; IR (neat) v_{max}/cm^{-1} = 3023, 2966, 1602, 1497, 1457, 1418, 1378, 1332, 1272, 1183, 1155, 1078, 1032; ^{1}H NMR (400 MHz, $CDCl_3$) δ 7.36–7.22 (m, 5H), 4.83 (dddd, *J* = 12.7, 8.4, 6.4 & 6.4 Hz, 1H), 3.26–3.18 (m, 3H), 2.98 (dd, *J* = 14.0 & 6.4 Hz, 1H), 2.58–2.49 (m, 1H), 2.37 (ddd, *J* = 17.5, 13.3 & 8.9 Hz, 1H); ^{13}C NMR (101 MHz, $CDCl_3$) δ 134.9, 129.5, 129.0, 127.6, 82.2, 45.7, 41.2, 29.0; HRMS (+ve EI) calc'd for $C_{10}H_{12}O_3S_1$ $[M]^+$ 212.0502; found 212.0501. $[\alpha]_D^{25}$ –29.3 (*c* 0.38, CH_2Cl_2); This compound was further characterised by x-ray crystallographic analysis. See section 12 (S65).

5-(4-Phenylbutyl)-1,2-oxathiolane 2,2-dioxide (4g)

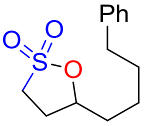

According to General Procedure A, 6-phenyl-1-hexene oxide[6] (193 mg, 1.10 mmol) gave, following flash column chromatography (SiO_2, 30% Et_2O/petrol), the *title compound* **4g** as thick oil (180 mg, 71%); IR (neat) v_{max}/cm^{-1} = 3027, 2935, 2860, 1604, 1497, 1455, 1343, 1271, 1195, 1158, 1043; ^{1}H NMR (400 MHz, $CDCl_3$) δ 7.30–7.15 (m, 5H), 4.66–4.56 (m, 1H), 3.35–3.19 (m, 2H), 2.63 (t, *J* = 7.5 Hz, 2H), 2.60–2.54 (m, 1H), 2.28 (dddd, *J* = 13.5, 9.4, 9.2 & 9.0Hz, 1H) , 1.95–1.85 (m, 1H), 1.76–1.64 (m, 3H), 1.60-1.40 (m, 2H); ^{13}C NMR (100 MHz, $CDCl_3$) δ 142.1, 128.5, 128.5, 126.0, 82.7, 45.8, 35.8 35.2, 31.1, 29.7, 24.8; HRMS (ESI) calc'd for $C_{13}H_{19}O_3S_1$ $[M + H]^+$ 255.1048; found 255.1047

5-(Cyclohex-3-en-1-yl)-1,2-oxathiolane 2,2-dioxide (4h).

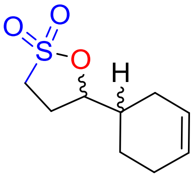

According to the general procedure, 2-(cyclohex-3-en-1-yl)oxirane (~1:1 mixture of diastereomers) (136 mg, 1.10 mmol) gave, following flash column chromatography (SiO_2, 30% Et_2O/petrol), the *title compound* **4h** (~1:1 mixture of diastereomers) as white powder (153 mg, 76 %); mp = 62–63 °C; IR (neat) v_{max}/cm^{-1} = 3025, 2919, 2838 1427, 1329, 1189, 1157, 1046; ^{1}H NMR (600 MHz, $CDCl_3$) δ 5.76–5.60 (m, 2H), 4.51–4.41 (m, 1H), 3.33 (ddt, *J* = 13.0, 8.6, 4.0 Hz, 1H), 3.26 (ddd, *J* = 13.1, 9.7, 8.2 Hz, 1H), 2.62–2.50 (m, 1H), 2.47–2.34 (m, 1H), 2.18–1.92 (m, 5H), 1.83–1.72 (m, 1H), 1.47–1.34 (m, 1H); ^{13}C NMR (151 MHz, $CDCl_3$) δ 127.9, 126.8, 125.3, 124.3, 86.1, 85.6, 45.9, 45.8, 38.6, 38.6, 27.7, 27.6, 27.5, 26.3, 24.7, 24.5, 24.2, 23.8; HRMS (ESI) calc'd for $C_9H_{15}O_3S_1$ $[M + H]^+$ 203.0736; found 203.0735.

---

(5)S. E. Schaus, B. D. Brandes, J. F. Larrow, M. Tokunaga, K. B. Hansen, A. E. Gould, M. E. Furrow, E. N. Jacobsen, *J. Am. Chem. Soc*. **2002**, *124*, 1307.
(6)P. Radha Krishna, R. Srinivas, *Tetrahedron Lett*. **2007**, 48, 2013.

5-(But-3-en-1-yl)-1,2-oxathiolane 2,2-dioxide (4i).

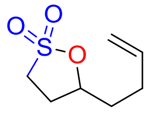

According to General Procedure A, 1,2-epoxy-5-hexene (124 µl, 1.10 mmol) gave, following flash column chromatography (SiO_2, 60% Et_2O/petrol), the *title compound* **4i** as waxy oil (153 mg, 81 %); IR (neat) ν_{max}/cm^{-1} = 3079, 2940, 1641, 1450, 1420, 1340, 1270, 1192, 1156, 1072, 1023; ^{1}H NMR (400 MHz, $CDCl_3$) δ 5.78 (dddd, *J* = 16.9, 10.1, 6.7 & 6.7 Hz, 1H), 5.12–4.98 (m, 2H), 4.70–4.58 (m, 1H), 3.38–3.18 (m, 2H), 2.60 (dddd, *J* = 13.0, 8.1, 6.0, 4.2 Hz, 1H), 2.37–2.17 (m, 3H), 1.97 (dtd, *J* = 14.3, 8.1 & 6.0 Hz, 1H), 1.84–1.69 (m, 1H); ^{13}C NMR (100 MHz, $CDCl_3$) δ 136.5, 116.4, 81.9, 45.7, 34.5, 29.6, 29.4; HRMS (ESI) calc'd for $C_7H_{13}O_3S_1$ $[M + H]^+$ 177.0580; found 177.0577.

(*R*)-5-((Benzyloxy)methyl)-1,2-oxathiolane 2,2-dioxide (4j).

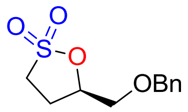

According to General Procedure B, commercial (*R*)-(+)-glycidyl benzyl ether (>97% ee) (153 µl, 1.00 mmol) gave, following flash column chromatography (SiO_2, 30% Et_2O/petrol) the *title compound* **4j** as yellow oil (208 mg, 86%); IR (neat) ν_{max}/cm^{-1} = 2862, 1721, 1496, 1453, 1342, 1270, 1196, 1161, 1097, 1026; ^{1}H NMR (400 MHz, $CDCl_3$) δ 7.40–7.28 (m, 5H), 4.77 (tt, *J* = 6.9, 4.2 Hz, 1H), 4.63, 4.58 (ABq, *J* = 11.9 Hz, 2H), 3.72, 3.68 (d of ABq, *J* = 11.1 & 3.9 Hz, 2H), 3.39–3.17 (m, 1H), 2.63 (ddt, *J* = 13.3, 8.1, 7.1 Hz, 1H), 2.49 (ddt, *J* = 13.3, 8.8, 6.7 Hz, 1H); ^{13}C NMR (100 MHz, $CDCl_3$) δ 137.4, 128.7, 128.2, 127.9, 80.1, 73.9, 70.8, 45.1, 25.8; HRMS (ESI) calc'd for $C_{11}H_{18}O_4S_1N_1$ $[M + NH_4]^+$ 260.0951; found 260.0948; $[\alpha]_D^{25}$ –16.4 (*c* 0.55, CH_2Cl_2); Enantiomeric excess >97% by HPLC (Daicel IC column, *i*-PrOH/hexane = 5/95, 30 °C, flow rate 1.0 mL/min, λ = 212 nm); t_R mj = 7.02 min, t_R mn = 6.53 min.

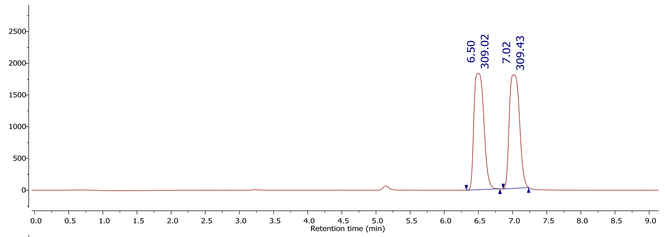

(±)-4j

Peak	Area
6.50	309.02
7.02	309.43

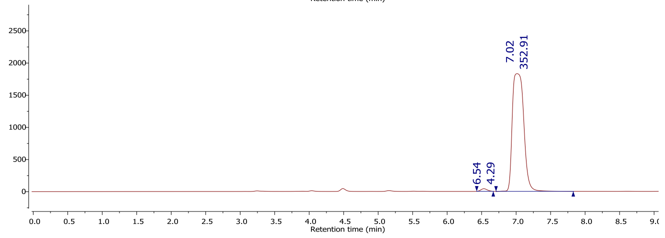

(*R*)-4j

Peak	Area
6.54	4.29
7.02	352.91

***tert*-Butyl (4-(2,2-dioxo-1,2-oxathiolan-5-yl)propyl)(methyl)carbamate (4k).**

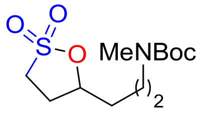

According to General Procedure B, 5-(*N*-Boc-*N*-methylamino)-1,2-epoxypentane[7] (215 mg, 1.00 mmol) gave, following flash column chromatography (SiO_2, 30% Et_2O/petrol), the *title compound* **4k** as colourless oil (141 mg, 48%); IR (neat) ν_{max}/cm^{-1} = 2974, 2935, 1683, 1481, 1454, 1424, 1394, 1364, 1344, 1245, 1216, 1153, 1099, 1049; ^{1}H NMR (400 MHz, $CDCl_3$) δ 4.67 (bs, 1H), 3.40–3.15 (m, 4H), 2.84 (bs, 3H), 2.67–2.55 (m, 1H), 2.35–2.23 (m, 1H), 1.88–1.60 (m, 4H), 1.45 (s, 9H); ^{13}C NMR (100 MHz, $CDCl_3$) δ 155.8, 82.2, 79.5, 47.5, 45.7, 34.1, 32.3, 29.6, 28.4, 23.4; HRMS (ESI) calc'd for $C_{12}H_{23}N_1O_5S_1Na$ $[M + Na]^+$ 316.1189; found 316.1185.

5-(2-Methyl-1,3-dioxolan-2-yl)-1,2-oxathiolane 2,2-dioxide (4l).

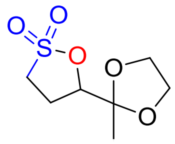

According to General Procedure B, 2-methyl-2-(oxiran-2-yl)-1,3-dioxolane[8] (131 mg, 1.00 mmol) gave, following flash column chromatography (SiO_2, 50% Et_2O/petrol) the *title compound* **4l** as clear oil (160 mg, 77%); IR (neat) ν_{max}/cm^{-1} = 2989, 2897, 1452, 1418, 1378, 1343, 1293 1255, 1226, 1189, 1156, 1099, 1047, 1008; ^{1}H NMR (400 MHz, $CDCl_3$) δ 4.55 (dd, *J* = 7.3 & 6.7 Hz, 1H), 4.11–3.99 (m, 4H), 3.35 (ddd, *J* = 12.7, 8.7, 7.7 Hz, 1H), 3.23 (ddd, *J* = 12.7, 7.8, 6.6 Hz, 1H), 2.65–2.48 (m, 2H), 1.37 (s, 3H); ^{13}C NMR (100 MHz, $CDCl_3$) δ 108.3, 83.4, 66.1, 66.1, 44.9, 24.7, 20.9.; HRMS (ESI) calc'd for $C_7H_{16}O_5S_1N_1$ $[M + NH_4]^+$ 226.0744; found 226.0745.

(7) D. M . Hodgson, N. J. Reynolds, S. J. Coote, *Tetrahedron Letters* **2002**, *43*, 7895-7897.
(8) P. Page, C. Blonski, J. Perie, *Bioorg. Med. Chem.* **1999**, *7*, 1403.

5-(4-Bromobutyl)-1,2-oxathiolane 2,2-dioxide (4m).

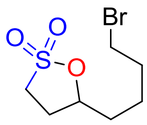

According to General Procedure B, 2-(4-bromobutyl)oxirane[9] (179 mg, 1.00 mmol) gave, following flash column chromatography (SiO_2, 50% Et_2O/petrol) the *title compound* **4m** as a clear oil (136 mg, 53%); IR (neat) ν_{max}/cm^{-1} = 2945, 1455, 1339, 1269, 1241, 1192, 1155, 1031; ^{1}H NMR (400 MHz, $CDCl_3$) δ 4.71–4.57 (m, 1H), 3.42 (t, *J* = 6.6 Hz, 2H), 3.33 (ddd, *J* = 13.3, 9.0 & 4.3 Hz, 1H), 3.29–3.19 (m, 1H), 2.67–2.57 (m, 1H), 2.31 (dddd, *J* = 13.0, 9.3, 9.0 & 9.0 Hz, 1H), 1.96–1.56 (m, 6H); ^{13}C NMR (100 MHz, $CDCl_3$) δ 82.3, 45.7, 34.5, 33.1, 32.1, 29.6, 24.0; HRMS (ESI) calc'd for $C_7H_{17}Br_1O_3S_1N_1$ [M + NH_4]$^+$ 274.0107; found 274.0106.

5,5'-(butane-1,4-diyl)bis(1,2-oxathiolane 2,2-dioxide) (4n).

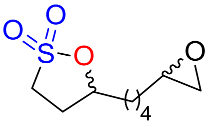

According to General Procedure B, 1,2,7,8-diepoxyoctane (~1:1 mixture of diastereomers) (142 mg, 1.00 mmol) gave, following flash column chromatography (SiO_2, 60% Et_2O/petrol) the *title compound* **4n** (~1:1 mixture of diastereomers) as heavy oil (220 mg, >99%); IR (neat) ν_{max}/cm^{-1} = 2935, 2863, 1686, 1456, 1416, 1339, 1269, 1187, 1156, 1037; ^{1}H NMR (400 MHz, $CDCl_3$) δ 4.67–4.57 (m, 1H), 3.36–3.19 (m, 2H), 2.93–2.86 (m, 1H), 2.77–2.72 (m, 1H), 2.65–2.54 (m, 1H), 2.48–2.44 (m, 1H), 2.35–2.23 (m, 1H), 1.93–1.42 (m, 8H); ^{13}C NMR (100 MHz, $CDCl_3$) 82.6, 82.6, 52.2, 52.2, 47.1, 47.1, 45.8, 45.8, 35.3, 35.2, 32.3, 32.3, 29.7, 29.7, 25.8, 25.7, 25.2, 25.1. HRMS (ESI) calc'd for $C_9H_{20}O_4S_1N_1$[M + NH_4]$^+$ 238.1113; found 238.1110.

(4*R,5*R**)-4,5-Dimethyl-1,2-oxathiolane 2,2-dioxide (4o).**

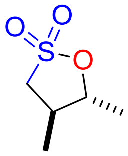

According to General Procedure B, *cis*–2,3–epoxybutane (87 µl, 1.00 mmol) gave, following flash column chromatography (SiO_2, 50% Et_2O/petrol) the *title compound* **4o** as clear oil (81 mg, 53%); IR (neat) ν_{max}/cm^{-1} = 2985, 2941, 1432, 1424, 1369, 1274, 1198, 1157, 1047; ^{1}H NMR (400 MHz, $CDCl_3$) δ 4.33 (dq, *J* = 9.2 & 6.1 Hz, 1H), 3.48 (dd, *J* = 13.0 & 8.1 Hz, 1H), 2.96 (dd, *J* = 13.0 & 10.5 Hz, 1H), 2.68–2.54 (m, 1H), 1.50 (d, *J* = 6.7 Hz, 3H), 1.21 (d, *J* = 6.1 Hz, 3H); ^{13}C NMR (100 MHz, $CDCl_3$) δ 85.3, 53.2, 39.8, 18.5, 15.8; HRMS calc'd for $C_5H_{11}O_3S_1$ [M + H]$^+$ 151.0423; found 151.0422.

(4*R,5*S**)-4,5-Dimethyl-1,2-oxathiolane 2,2-dioxide (4p).**

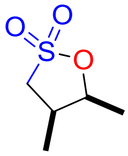

According to General Procedure B, *trans*–2,3–epoxybutane (87 µl, 1.00 mmol) gave, following flash column chromatography (SiO_2, 50% Et_2O/petrol) the *title compound* **4p** as clear oil (81 mg, 54%); IR (neat) ν_{max}/cm^{-1} = 2984, 2937, 1456, 1423, 1352, 1278, 1196, 1157, 1025; ^{1}H NMR (400 MHz, $CDCl_3$) δ 4.85 (dq, *J* = 6.6 & 5.6 Hz, 1H), 3.45 (dd, *J* = 13.2 & 6.6, Hz, 1H), 3.04 (dd, *J* = 13.2 & 7.1 Hz, 1H), 3.01–2.86 (m, 1H), 1.42 (d, *J* = 6.6 Hz, 6H), 1.21 (d, *J* = 7.1 Hz, 6H); ^{13}C NMR (100 MHz, $CDCl_3$) δ 82.4, 52.7, 36.1, 15.6, 13.8; HRMS calc'd for $C_5H_{11}O_3S_1$ [M + H]$^+$ 151.0423; found 151.0424.

1-Oxa-2-thiaspiro[4.5]decane 2,2-dioxide (4q).

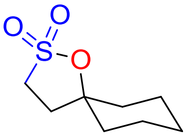

According to General Procedure A, methylenecyclohexane oxide (112 mg, 1.10 mmol) gave, following flash column chromatography (SiO_2, 40% Et_2O/petrol), the *title compound* **4q** as thick oil (189 mg, >99 %); IR (neat) ν_{max}/cm^{-1} = 2933, 2862, 1681, 1447, 1424, 1375, 1329, 1321, 1269, 1239, 1190, 1155, 1122, 1099, 1025; ^{1}H NMR (400 MHz, $CDCl_3$) δ 3.30 (dd, *J* = 7.7 & 7.5 Hz, 2H), 2.39 (dd, *J* = 7.7 & 7.5 Hz, 2H), 2.08–1.99 (m, 2H), 1.77–1.24 (m, 8H); ^{13}C NMR (100 MHz, $CDCl_3$) δ 90.9, 45.2, 37.3, 34.7, 24.7, 22.4; HRMS (ESI) calc'd for $C_8H_{15}O_3S_1$ [M + H]$^+$ 191.0736; found 191.0736.

5,5-Dimethyl-1,2-oxathiolane 2,2-dioxide (4r).

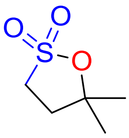

According to General Procedure A, isobutylene oxide (97 µl, 1.10 mmol) gave, following flash column chromatography (SiO_2, 40% Et_2O/petrol), the *title compound* **4r** as thick oil (179 mg, >99 %); IR (neat) ν_{max}/cm^{-1} = 2976, 1697, 1446, 1372, 1196, 1132, 1102, 1029; ^{1}H NMR (400 MHz, $CDCl_3$) δ 3.34 (t, *J* = 7.6 Hz, 2H), 2.45 (t, *J* = 7.6 Hz, 2H), 1.57 (s, 6H); ^{13}C NMR (100 MHz, $CDCl_3$) δ 88.9, 46.0, 36.1, 28.4; HRMS (ESI) calc'd for $C_5H_{11}O_3S_1$ [M + H]$^+$ 151.0423; found 151.0420.

(9) S. Sévigny, I. Bouchard, S. Motallebi, Y. Zhao, *Macromolecules* **2003**, *36*, 9033.

1,8-Dioxa-2-thiaspiro[4.5]decane 2,2-dioxide (4s)

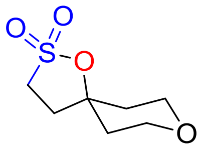

According to General Procedure B, 4-tetrahydropyranylidene oxide (114 mg, 1.00 mmol) gave, following flash column chromatography (SiO_2, 50% Et_2O/petrol) the *title compound* **4s** as white powder (191 mg, 99%); mp 83–85 °C; IR (neat) ν_{max}/cm^{-1} = 3018, 2971, 2931, 2873, 1707, 1476, 1435, 1372, 1343, 1314, 1250, 1175, 1158; ^{1}H NMR (400 MHz, $CDCl_3$) δ 3.88–3.73 (m, 4H), 3.34 (dd, *J* = 7.7, 7.7 Hz, 2H), 2.45 (dd, *J*= 7.7, 7.7 Hz, 2H), 2.08–2.00 (m, 2H), 1.89–1.79 (m, 2H); ^{13}C NMR (100 MHz, $CDCl_3$) δ 87.0, 64.1, 45.0, 37.3, 35.1; HRMS (ESI) calc'd for $C_7H_{16}O_4SN$ $[M + NH_4]^+$ 210.0795; found 210.0796.

1-Oxa-2,8-dithiaspiro[4.5]decane 2,2-dioxide (4t)

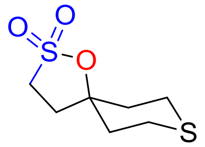

According to General Procedure B, 1-oxa-6-thiaspiro[2.5]octane (130 mg, 1.00 mmol) gave, following flash column chromatography (SiO_2, 60% Et_2O/petrol) the *title compound* **4t** (135 mg, 65%) as colourless oil; IR (neat) ν_{max}/cm^{-1} = 3029, 2945, 2920, 1735, 1453, 1428, 1376, 1336, 1326, 1289, 1273, 1257, 1217, 1174, 1160, 1110, 1079, 1038, 1006; ^{1}H NMR (400 MHz, $CDCl_3$) δ 3.31 (t, *J* = 7.5 Hz, 2H), 3.02 (ddd, *J* = 14.2, 11.6 & 2.5, 2H), 2.55–2.48 (m, 2H), 2.39 (t, *J* = 7.5 Hz, 2H), 2.37–2.33 (m, 2H), 1.89 (ddd, *J* = 14.2, 11.6 & 3.8 Hz, 2H); ^{13}C NMR (100 MHz, $CDCl_3$) δ 88.0, 44.9, 38.4, 35.7, 24.6; HRMS calc'd for $C_7H_{13}O_3S_2$ $[M + H]^+$ 209.0301; found 209.0301.

***tert*-Butyl 1-oxa-2-thia-8-azaspiro[4.5]decane-8-carboxylate 2,2-dioxide (4u)**

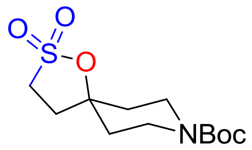

According to general Procedure B, *tert*-butyl 1-oxa-6-azaspiro[2.5]octane-6-carboxylate (213 mg, 1.00 mmol) gave, following flash column chromatography (SiO_2, 50% Et_2O/petrol) the *title compound* **4u** as white powder (191 mg, 99%); mp 131–133 °C; IR (neat) ν_{max}/cm^{-1} = 3019, 2978, 2957, 2930, 2909, 1686, 1481, 1464, 1448, 1418, 1394, 1384, 1365, 1353, 1342, 1334, 1316, 1282, 1271, 1242, 1192, 1178, 1159, 1137, 1085, 1063, 1036; ^{1}H NMR (400 MHz, $CDCl_3$) δ 3.94 (bs, 2H), 3.34 (t, *J* = 7.8 Hz, 2H), 3.16 (bt, *J* = 12.6 Hz, 2H), 2.42 (t, *J* = 7.8, Hz, 2H), 2.06 (bdd, *J* = 14.2 & 2.0, 2H), 1.72–1.63 (m, 2H), 1.46 (s, 9H); ^{13}C NMR (100 MHz, $CDCl_3$) δ 154.6, 87.7, 80.2, 45.0, 40.2, 36.6, 34.9, 28.5; HRMS (ESI) calc'd for $C_{12}H_{22}NO_5S_1$ $[M + H]^+$ 292.1213; found 292.1209.

***tert*-Butyl (1*R*,3*r*,5*S*)-8-azaspiro[bicyclo[3.2.1]octane-3,5'-[1,2]oxathiolane]-8-carboxylate 2',2'-dioxide (4v)**

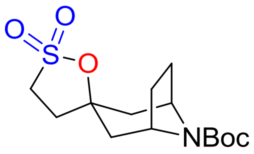

According to general Procedure B, *tert*-butyl (1R,3r,5S)-8-azaspiro[bicyclo[3.2.1]octane-3,2'-oxirane]-8-carboxylate[10] (239 mg, 1.00 mmol) gave, following flash column chromatography (SiO_2, 60% Et_2O/petrol) the *title compound* **4v** as colourless oil (151 mg, 52%); IR (neat) ν_{max}/cm^{-1} = 3019, 2976, 2926, 1687, 1467, 1445, 1428, 1368, 1332, 1315, 1270, 1258, 1223, 1201, 1152, 1124, 1110, 1095, 1054, 1044, 1017; ^{1}H NMR (400 MHz, $CDCl_3$) δ 4.27 (bs, 2H), 3.25 (t, *J* = 7.8 Hz, 2H), 2.35 (t, *J* = 7.8 Hz, 2H), 2.26–1.87 (m, 8H), 1.46 (s, 9H); ^{13}C NMR (100 MHz, $CDCl_3$) δ 153.3, 89.2, 79.9, 52.8, 52.3, 44.7, 41.3, 41.5, 38.2, 28.6, 27.6, 27.2; HRMS (ESI) calc'd for $C_{14}H_{24}O_5S_1N_1$ $[M + H]^+$ 318.1370; found 318.1364.

5-Phenyl-1,2-oxathiolane 2,2-dioxide (4w)

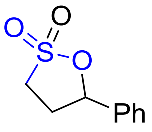

Sultine (±)-**7a** (52 mg, 0.28 mmol) was dissolved in PhH (1 ml) to which *m*-CPBA (84 mg, 0.34 mmol) was added in a single portion. The reaction mixture was left to stir at rt. for 48 h after which time the solution was washed with 10% aqueous $NaHCO_3$ solution (2 ml) then H_2O (3 ml). The separated organic phase was dried over $MgSO_4$ and the solvent was evaporated *in vacuo* to give the title compound **4w** (39 mg, 71%) as colourless oil; ^{1}H NMR (400 MHz, $CDCl_3$) δ 7.46–7.36 (m, 5H), 5.61 (dd, *J* = 9.2 & 6.0 Hz 1H), 3.53–3.34 (m, 2H), 2.89 (dddd, *J* = 13.6, 7.6, 6.0 & 4.1, 1H), 2.66 (dddd, *J* = 13.6, 10.0, 9.2 & 8.4 Hz, 1H); ^{13}C (100 MHz, $CDCl_3$) δ 136.9, 129.5, 129.1, 125.9, 83.2, 77.12, 46.3, 32.5. These data are consistent with those already published.[11]

---

(10) N. Schlienger, B. W. Lund, J. Pawlas, F. Badalassi, F. Bertozzi, R. Lewinsky, A. Fejzic, M. B. Thygesen, A. Tabatabaei, S. R. Bradley, L. R. Gardellm, F. Piu, R. Olsson, *J. Med. Chem.* **2009**, *52*, 7186.
(11) O. B. Bondarenko, T. I. Voevodskaya, L. G. Saginova, V. A. Tafeenko, Y. S. Shabarov, *J. Org. Chem. USSR (English Translation)*, **1987**, *23*, 155.

4. Experimental Procedures for γ-hydroxysulfones 5a-j:

General Procedure C: The BF_3-Mediated Ring-Opening of Terminal Epoxides by Sulfones

$KN(SiMe_3)_2$ (0.70 M in Hexanes, 2.5 eq.) was added to a mixture of the appropriate sulfone (1.0 eq) and epoxide (2.0 eq.) in 1,2-dimethoxyethane (DME) which had been pre-cooled to –40 °C under an atmosphere of Ar. To this was added $BF_3{\cdot}Et_2O$ (2.0 eq.) and the resultant mixture was stirred at –40 °C for 18 h. The reaction was quenched by addition of sat. aq. NH_4Cl solution and extracted with EtOAc. The combined organic phases were washed with brine, dried over $MgSO_4$, filtered and concentrated *in vacuo*. The residue was dry loaded onto silica[12] using Et_2O and purified by flash column chromatography as described for each entry to yield the title compounds.

Spectral Data for γ-hydroxysulfones 5a-j

(*S*)-3-((1-(*tert*-Butyl)-1*H*-tetrazol-5-yl)sulfonyl)-1-phenylpropan-1-ol (5a)

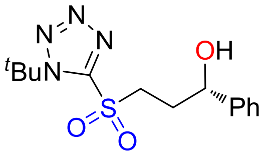

According to General Procedure C, $TBTSO_2Me$ **1a** (300 mg, 1.47 mmol), (*R*)-styrene oxide (336 µl, 2.94 mmol), $KN(SiMe_3)_2$ (5.25 ml, 0.70 M in Hexanes, 3.67 mmol), $BF_3{\cdot}Et_2O$ (0.36 ml, 2.94 mmol) and DME (5 ml) gave, following purification by flash column chromatography (SiO_2, 60% Et_2O/petrol), the *title compound* **5a** as light yellow oil (361 mg, 76%); IR (neat) ν_{max}/cm^{-1} = 3411, 2974, 2935, 2360, 2341, 1463, 1407, 1376, 1335, 1238, 1208, 1158, 1123; ^1H NMR (400 MHz, $CDCl_3$) δ 7.40–7.28 (m, 5H), 4.94 (dd, *J* = 6.4 & 6.4 Hz, 1H), 4.05–3.88 (m, 2H), 2.45–2.35 (m, 2H), 1.84 (s, 9H); ^{13}C NMR (100 MHz, $CDCl_3$) δ 153.9, 142.8, 128.8, 127.3, 125.7, 72.1, 65.5, 53.7, 31.7, 29.7; HRMS (ESI) calc'd for $C_{14}H_{20}N_4O_3S$ $[M]^+$ 324.1256; found 324.1257.

3-((1-(*tert*-Butyl)-1*H*-tetrazol-5-yl)sulfonyl)-1-(naphthalen-2-yl)propan-1-ol (5b)

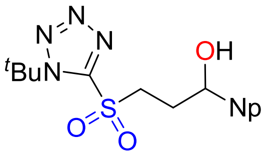

According to General Procedure C, $TBTSO_2Me$ **1a** (300 mg, 1.47 mmol), 2-naphthyloxirane (500 mg, 2.94 mmol), $KN(SiMe_3)_2$ (5.25 ml, 0.7 M in Hexanes, 3.67 mmol), $BF_3{\cdot}Et_2O$ (0.36 ml, 2.94 mmol) and DME (5 ml) gave, following purification by flash column chromatography (SiO_2, 20% EtOAc/petrol) the *title compound* **5b** as colourless oil (93 mg, 17%); IR (neat) ν_{max}/cm^{-1} = 3325, 3061, 3030, 2965, 2928, 2869, 1493, 1453, 1394, 1366, 1330, 1267, 1204, 1182, 1083, 1000; ^1H NMR (400 MHz, $CDCl_3$) δ 7.86–7.49 (m, 7H), 5.16–5.08 (m, 1H), 4.05–3.97 (m, 2H), 2.56–2.46 (m, 2H), 1.85 (s, 9H); ^{13}C NMR (100 MHz, $CDCl_3$) δ 153.9, 140.0, 133.2, 128.8, 128.0, 127.7, 126.5, 126.5, 126.3, 124.6, 123.4, 72.2, 65.5, 53.7, 31.6, 29.7; HRMS (APCI) calc'd for $C_{18}H_{23}N_4O_3S$ $[M + H]^+$ 375.1485; found 375.1481.

4-((1-(*tert*-Butyl)-1*H*-tetrazol-5-yl)sulfonyl)-1,1,1-trifluorobutan-2-ol (5d)

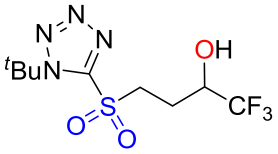

According to General Procedure A, 2-(trifluoromethyl)oxirane (86 µl, 1.0 mmol) gave, following purification by flash column chromatography (SiO_2, 20% EtOAc/petrol) as clear oil (314 mg, 99%); IR (neat) ν_{max}/cm^{-1} = 3428, 2991, 2944, 1708, 1480, 1466, 1447, 1408, 1377, 1336, 1274, 1239, 1153, 1126, 1099, 1045, 1024; ^1H NMR (400 MHz, $CDCl_3$) δ 4.30–4.20 (m, 1H), 4.15–3.98 (m, 2H), 2.98 (d, *J* = 6.1, 1H), 2.53–2.42 (m, 1H), 2.39–2.27 (m, 1H), 1.86 (s, 9H); ^{13}C NMR (100 MHz, $CDCl_3$) δ 153.9, 124.6 (q, *J* = 282.0 Hz), 68.5 (q, *J* = 32.3Hz), 65.9, 52.6, 29.8, 23.7; HRMS (ESI) calc'd for $C_9H_{16}F_3N_4O_3S$ $[M + H]^+$ 317.0890; found 317.0893.

4-((1-(*tert*-Butyl)-1*H*-tetrazol-5-yl)sulfonyl)butan-2-ol (5e)

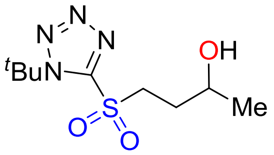

According to General Procedure C, $TBTSO_2Me$ **1a** (300 mg, 1.47 mmol), propylene oxide (0.205 ml, 2.94 mmol), $KN(SiMe_3)_2$ (5.25 ml, 0.7 M in Hexanes, 3.67 mmol), $BF_3{\cdot}Et_2O$ (0.36 ml, 2.94 mmol) and DME (5 ml) gave, following purification by flash column chromatography (SiO_2, 30% EtOAc/petrol) the *title compound* **5e** as colourless oil (246 mg, 64%); IR (neat) ν_{max}/cm^{-1} = 3357, 2984, 2360, 2341, 1635, 1407, 1376, 1207, 1158, 1122; ^1H NMR (400 MHz, $CDCl_3$) δ 4.07–3.91 (m, 3H), 2.20 (dddd, *J* = 13.8, 9.7, 6.1 & 3.7 Hz, 1H), 2.04 (dddd, *J* = 13.8, 9.7, 8.6, 5.5 Hz, 1H), 1.86 (s, 9H), 1.67 (d, *J* = 5.2 Hz, 1H), 1.30 (d, *J* = 6.2 Hz, 3H); ^{13}C NMR (100 MHz, $CDCl_3$) δ 154.2, 66.1, 65.7, 53.9, 31.7, 29.8 23.8; HRMS (ESI) calc'd for $C_9H_{19}N_4O_3S$ $[M + H]^+$ 263.1172; found 263.1173.

---

(12) The use of Et_2O proved advantageous since it triturated the majority of insoluble polymeric epoxide.

4-((1-(*tert*-Butyl)-1*H*-tetrazol-5-yl)sulfonyl)-4-methylpentan-2-ol (5f)

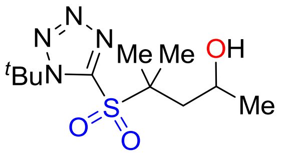

According to General Procedure C, $TBTSO_2{}^iPr$ **1b** (696 mg, 3.00 mmol), propylene oxide (420 µl, 6 mmol), $KN(SiMe_3)_2$ (10.4 ml, 7.50 mmol), $BF_3{\cdot}Et_2O$ (0.74 ml, 6 mmol) and DME (10 ml) gave, following purification by flash column chromatography (SiO_2, 50% EtOAc/petrol) the *title compound* **5f** as white powder (478 mg, 55%); mp 85–87 °C; IR (neat) v_{max}/cm^{-1} = 3545, 3001, 2975, 2943, 2891, 1470, 1397, 1377, 1319, 1259, 1240, 1195, 1175, 1133, 1112, 1077, 1044, 1026; ^1H NMR (400 MHz, $CDCl_3$) δ 4.22–4.11 (m, 1H), 2.33–2.19 (m, 2H), 1.87 (s, 9H), 1.76 (s, 3H), 1.74 (s, 3H), 1.28 (d, *J* = 6.3 Hz, 3H); ^{13}C NMR (100 MHz, $CDCl_3$) δ 152.8, 70.3, 66.3, 65.0, 43.8, 30.0, 25.9, 22.5, 21.0; HRMS (EI) calc'd for $C_{11}H_{23}N_4O_3S$ $[M + H]^+$ 291.1485; found 291.1486.

The ring-opening of (*R*)-styrene oxide by $TBTSO_2{}^iPr$ 1b.

According to General Procedure C, $TBTSO_2{}^iPr$ **1b** (696 mg, 3.00 mmol), (*R*)-styrene oxide (690 µl, 6.0 mmol), $KN(SiMe_3)_2$ (10.4 ml, 7.5 mmol), $BF_3{\cdot}Et_2O$ (0.74 ml, 6.00 mmol) and DME (10 ml) gave, following purification by flash column chromatography (SiO_2, 50% Et_2O/petrol), the *regioisomers* **5g** (256 mg, 24%) and **5i** (261 mg, 25%):

(*S*)-3-((1-(*tert*-Butyl)-1*H*-tetrazol-5-yl)sulfonyl)-3-methyl-1-phenylbutan-1-ol (5g)

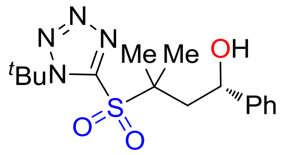

White powder; mp 113–114 °C; IR (neat) v_{max}/cm^{-1} = 3545, 3521, 3436, 3030, 2957, 2873, 1494, 1455, 1405, 1376, 1318, 1277, 1242, 1202, 1165, 1130, 1106, 1057, 1026; ^1H NMR (400 MHz, $CDCl_3$) δ 7.40–7.27 (m, 5H), 5.08–5.02 (m, 1H), 2.62–2.47 (m, 2H), 2.04 (bd, *J* = 1.9 Hz, 1H), 1.86 (s, 9H), 1.84 (s, 3H), 1.83 (s, 3H); ^{13}C NMR (100 MHz, $CDCl_3$) δ 152.8, 144.6, 128.9, 128.2, 125.8, 71.6, 70.3, 66.3, 43.9, 30.0, 22.8, 20.9. HRMS (ESI) calc'd for $C_{16}H_{25}N_4O_3S$ $[M + H]^+$ 353.1642; found 353.1638.

3-((1-(*tert*-Butyl)-1*H*-tetrazol-5-yl)sulfonyl)-3-methyl-2-phenylbutan-1-ol (5i):

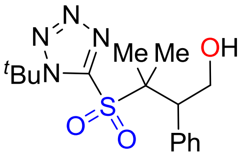

White powder; mp 104–107 °C; IR (neat) v_{max}/cm^{-1} = 3530, 3027. 2963, 2917, 2896, 1492, 1455, 1437, 1402, 1364, 1325, 1204, 1178, 1156, 1101, 1087; ^1H NMR (400 MHz, $CDCl_3$) δ 7.40–7.28 (m, 5H), 4.41–4.31 (m, 1H), 4.16–4.03 (m, 2H), 1.87 (s, 9H), 1.77 (s, 3H), 1.40 (s, 3H); ^{13}C NMR (100 MHz, $CDCl_3$) δ 153.3, 137.4, 130.2, 128.9, 128.2, 73.2, 66.5, 62.7, 51.3, 30.0, 23.7, 18.3; HRMS (ESI) calc'd for $C_{16}H_{25}N_4O_3S$ $[M + H]^+$ 353.1642; found 353.1645.

The ring-opening of (*R*)-styrene oxide by $TBTSO_2CHEt_2$ 1c

According to General Procedure C, $TBTSO_2CHEt_2$ **1c** (725 mg, 2.80 mmol), (*R*)-styrene oxide (640 µl, 5.6 mmol) $KN(SiMe_3)_2$ (9.0 ml, 6.3 mmol), $BF_3{\cdot}Et_2O$ (0.63 ml, 5.1 mmol) and DME (9 ml) gave, following purification by flash column chromatography (SiO_2, 50% Et_2O/petrol), the *regioiomers* **5h** (203 mg, 19%) and **5j** (262 mg, 25%) as thick oils.

(*S*)-3-((1-(*tert*-Butyl)-1*H*-tetrazol-5-yl)sulfonyl)-3-ethyl-1-phenylpentan-1-ol (5h)

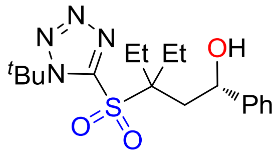

IR (neat) v_{max}/cm^{-1} = 3382, 2975, 2212, 1710, 1602, 1560, 1495, 1455, 1374, 1314, 1244, 1202, 1155, 1131, 1101, 1025, 1005; ^1H NMR (400 MHz, $CDCl_3$) δ 7.39–7.27 (m, 5H), 5.14 (dt, *J* = 9.7 & 2.5 Hz, 1H), 2.64 (dd, *J* = 15.7 & 9.8 Hz, 1H), 2.52–2.34 (m, 5H), 2.25 (dq, *J* = 15.0 & 7.5 Hz, 1H), 1.88 (s, 9H), 1.09 (t, *J* = 7.5 Hz, 3H), 1.06 (t, *J* = 7.5 Hz, 3H); ^{13}C NMR (100 MHz, $CDCl_3$) δ 154.1, 145.1, 128.8, 127.9, 125.7, 77.4, 70.6, 66.4, 42.7, 30.0, 26.3, 26.0, 8.9, 8.8; HRMS (EI) calc'd for $C_{18}H_{29}N_4O_3S$ $[M + H]^+$ 381.1955; found 381.1950.

3-((1-(*tert*-Butyl)-1*H*-tetrazol-5-yl)sulfonyl)-3-ethyl-2-phenylpentan-1-ol (5j)[13]

(13) This compound partially degraded to the corresponding sultine and was co-polar with the tetrazole containing by-product (**S3**), which can clearly be seen in the NMR spectra.

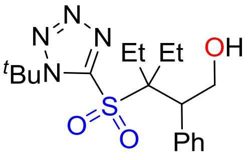

IR (neat) ν_{max}/cm^{-1} = 3434, 3249, 2981, 2215, 1712, 1499, 1458, 1377, 1366, 1308, 1245, 1204, 1146, 1100; ^{1}H NMR (400 MHz, $CDCl_3$) δ 7.40–7.27 (m, 5H), 4.25–4.19 (m, 2H), 3.99 (dd, *J* = 7.7 & 6.0 Hz, 1H), 2.49 (dq, *J* = 7.5, 15.0 Hz, 1H), 2.40 (dq, *J* = 7.5, 15.0 Hz, 1H), 2.33 (dq, *J* = 7.5 Hz, 15.0), 2.23 (dq, *J* = 7.5, 15.0 Hz, 1H), 1.86 (s, 9H), 1.14 (t, *J* = 7.5 Hz, 3H), 0.93 (t, *J* = 7.5 Hz, 3H); ^{13}C NMR (100 MHz, $CDCl_3$) δ 153.2, 136.8, 130.8, 128.8, 128.2, 81.5, 66.4, 62.6, 51.0, 29.9, 29.3, 26.0, 9.4, 9.1; HRMS (EI) calc'd for $C_{18}H_{29}N_4O_3S$ $[M + H]^+$ 381.1955; found 381.1958.

5. Experimental Procedures for Sultines 7a-j:

General procedure D: The cyclisation of γ-hydroxysulfones 5 to γ-sultines 7.

To a solution of the appropriate γ-hydroxysulfone **5** in MeCN (1 M), DBU (1.2 eq.) was added and allowed to stir at 20–25 °C for 24 h. After this time the reaction mixture was quenched with aq. HCl (1 M) and extracted with EtOAc. The combined organic phases were washed with brine, dried over $MgSO_4$, filtered and concentrated *in vacuo*. The residues were purified by flash column chromatography as described for each entry to afford the following sultine products.

5-Phenyl-1,2-oxathiolane-2-oxide[14] (7a)

According to General Procedure D, γ-hydroxysulfone (*S*)-**5a** (153 mg, 0.47 mmol), DBU (85 µl, 0.57mmol) and MeCN (1 ml) gave, following flash column chromatography (SiO_2, 30% EtOAc/petrol) the title compound **7a** as light yellow oil (64 mg, 76%, 76:24 / *trans:cis*). The product derived from (*S*)-styrene oxide was analyzed as a mixture of diastereomers, using chiral HPLC (Daicel IC column, *i*-PrOH/hexane = 15/85, 30 °C, flow rate 1.0 ml/min, λ = 212 nm).

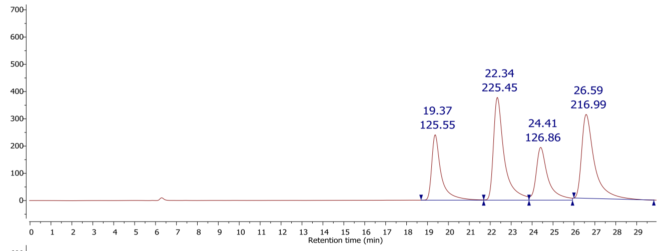

(±)-7a

Peak	Area
19.37	125.55
22.34	225.45
24.41	126.86
26.59	216.99

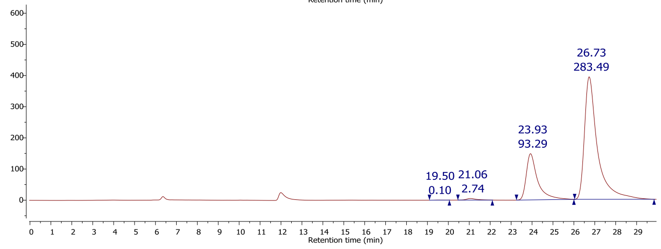

(2*S*,5*S*)-7a (*trans*)

Peak	Area
21.06	2.74
26.73	283.49

(2*R*,5*S*)-7a (*cis*)

Peak	Area
19.50	0.10
23.93	92.12

Analytically pure samples were separated by preparative HPLC using a SUPERCILGOLD dC_{18} 5µ 30x100mm column according to the following gradient table:

Time (mins)	Solvent A (%)	Solvent B (%)	Flow (ml / mn)
0.00	95	5	33
1.50	95	5	33
1.51	74	26	33
13.0	74	26	33
13.2	0	100	33
19.0	0	100	33
19.1	95	5	33
19.95	95	5	33
20.0	95	5	33

Solvent A: H_2O with 0.05% TFA.
Solvent B: CH_3CN with 0.05% TFA

(14) N. K. Sharma, F. De Reinach-Hirtzbach, T. Durst, *Can. J. Chem.* **1976**, *54*, 3012.

(2*R*,5*R*)-7a (*trans*, major):

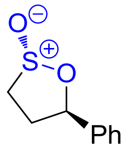

Clear oil; IR (neat) v_{max}/cm^{-1} = 3031, 1670, 1493, 1456, 1414, 1369, 1309, 1209, 1141, 1028; ^{1}H NMR (400 MHz, $CDCl_3$) δ 7.46–7.30 (m, 5H), 6.06 (dd, *J* = 6.7 & 6.7 Hz, 1H), 3.39 (ddd, *J* = 12.8, 9.2 & 7.7 Hz, 1H), 3.07 (ddd, *J* = 12.8, 7.5 & 5.2 Hz, 1H), 2.94 (dddd, *J* = 12.8, 7.7, 7.5 & 6.7 Hz, 1H), 2.23 (dddd, *J* = 12.8, 9.2, 6.7 & 5.2 Hz, 1H); ^{13}C NMR (100 MHz, $CDCl_3$) δ 138.2, 128.9, 128.8, 126.0, 87.9, 58.9, 31.5; Enantiomeric excess: > 98 % ee (Daicel IC column, ^{i}PrOH/hexane = 15/85, 30 °C, flow rate 1.0 ml/min, λ = 212 nm).

(2*S*,5*R*)-7a (*cis*, minor):

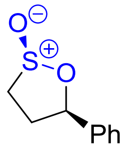

White solid; mp = 54–56 °C; IR (neat) v_{max}/cm^{-1} = 3028, 1674, 1498, 1458, 1322, 119, 1136; ^{1}H NMR (400 MHz, $CDCl_3$) δ 7.47–7.31 (m, 5H), 5.49 (dd, *J* = 10.8 & 6.0 Hz, 1H), 3.27 (dd, J = 12.2, 5.5Hz, 1H), 3.02 (ddd, *J* = 13.7, 12.2, 7.0 Hz, 1H), 2.75–2.54 (m, 2H); ^{13}C NMR (100 MHz, $CDCl_3$) δ 138.7, 128.9, 128.9, 128.7, 127.3, 92.9, 58.7 31.9; Enantiomeric excess: > 99 % ee (Daicel IC column, ^{i}PrOH/hexane = 15/85, 30 °C, flow rate 1.0 ml/min, λ = 212 nm); the racemate of this compound was further characterised by x-ray crystallographic analysis. See section 12 (S65).

Cyclopropylbenzene

Sultines **7a** (79 mg, 0.40 mmol) in PhH (8 ml) and Me_2CO (2 ml) (both degassed and dried over mol. sieves prior to use) were photolyzed for 40 min using a 400 W high pressure mercury immersion lamp modified to use FEP tubing (0.8 mm ID x 1mm OD) as described in the literature.[15] Solvent was removed *in vacuo* and the residue was purified by column chromatography (SiO_2, 30% EtOAc/petrol) to give the title compound (38 mg, 80%) as light yellow oil; ^{1}H NMR (400 MHz, $CDCl_3$) δ 7.37–7.18 (m, 5H), 2.02–1.98 (m, 1H), 1.09–1.05 (m, 2H), 0.81–0.77 (m, 2H); ^{13}C NMR (101 MHz, $CDCl_3$) δ 144.0, 128.4, 125.7, 125.5, 15.5, 9.3.

5-(Naphthalen-2-yl)-1,2-oxathiolane 2-oxide (7b)

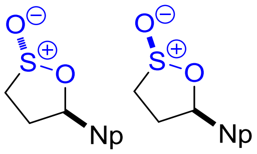

According to General Procedure D, alcohol **5b** (82 mg, 0.22 mmol), DBU (40 μl, 0.26 mmol) and MeCN (0.50 ml) gave, following flash column (SiO_2, 20% EtOAc/petrol), the *title compounds* **7b,** an inseparable mixture of diastereomers, as white solid (25 mg, 51%, 2:1 dr / *trans:cis*); mp (mixture) = 125–127 °C; IR (neat mixture) v_{max}/cm^{-1} = 3054, 2937, 2212, 1960, 1842, 1783, 1709, 1600, 1505, 1471, 1453, 1439, 1414, 1394, 1373, 1330, 1304, 1273, 1259, 1241, 1178, 1151, 1144, 1133, 1126, 11000, 1017; HRMS (mixture) (ESI) calc'd for $C_{13}H_{13}O_2S$ [M + H]$^{+}$ 233.0631, found; 233.0629; ***trans*-7b:**^{1}H NMR (400 MHz, $CDCl_3$) δ 7.92–7.37 (m, 4.6H), 6.22 (dd, *J* = 6.7 & 6.7 Hz, 0.65H), 3.40 (ddd, *J* = 12.3, 9.3 & 7.6Hz, 0.65H), 3.13–2.96 (m, 0.65H), 2.80–2.72 (m, 0.65H), 2.32 (dddd, *J* = 12.8, 9.5, 4.7 & 6.7 Hz, 0.65H); ^{13}C NMR (100 MHz, $CDCl_3$) δ 135.7, 133.4, 133.2, 129.0, 128.2, 127.9, 126.8, 126.7, 125.2, 123.5, 87.9, 58.9, 31.5; ***cis*-7b**: ^{1}H NMR (400 MHz, $CDCl_3$) δ 7.92–7.37 (m, 2.4H), 5.65 (dd, *J* = 8.7, 0.35H), 3.32–3.26 (m, 0.35H), 3.13–2.96 (m, 3 x 0.35H); ^{13}C NMR (101 MHz, $CDCl_3$) δ 136.1, 133.5, 133.2, 129.1, 128.2, 127.9, 126.6, 126.6, 126.6, 124.9, 93.0, 58.9, 31.9.

5-Methyl-1,2-oxathiolane 2-oxide 7e

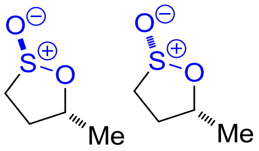

To a solution of γ-hydroxysulfone **5e** (143 mg, 0.65 mmol) in MeCN (1.5 ml), DBU (117 μl, 0.78 mmol) was added and allowed to stir at 20 °C for 24 h. After this time the reaction mixture was quenched with aq. HCl (1 M, 1.5 ml) and extracted with EtOAc (2 x 3 ml). The combined organic phases were washed with brine, dried over $MgSO_4$, filtered and the solvent removed *in vacuo* to give the title compound as a mixture of two diastereomers as well as sultone **4a** (***trans*-7e**:***cis*-7e**:**4a** 69:25:6); selected data: ***trans*-7b** ^{1}H NMR (400 MHz, $CDCl_3$) 1.40 (d, *J* = 6.3 Hz, 3H); ***cis*-7b** ^{1}H NMR (400 MHz, $CDCl_3$) 4.67 (dqd, *J* = 10.4, 6.1 & 6.0 Hz, 1H), 3.13 (ddd, *J* = 12.3, 6.0 & 1.0 Hz, 1H), 1.61 (d, *J* = 6.1 Hz, 3H); **mixture**, HRMS: calc'd $C_4H_9O_3S$ [M+H]$^{+}$ 121.0318; found 121.0314.

(15) B. D. A. Hook, W. Dohle, P. R. Hirst, M. Pickworth, M. B. Berry, K. Booker-Milburn, *J. Org. Chem.* **2005**, *70*, 7558.

3,3-Dimethyl-5-methyl-1,2-oxathiolane 2-oxide (7f)

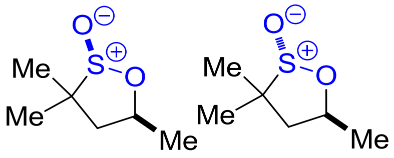

According to General Procedure D, alcohol **5f** (291 mg, 1.00 mmol), DBU (0.178 ml, 1.2 mmol) and MeCN (2ml) for 72 h gave, following flash column chromatography (SiO_2, 10 % Et_2O/petrol) the *title compound* as mixture of diastereomers (31 mg, 21 %, 50:50 / *trans:cis*) as clear oil; IR (neat mixture) ν_{max}/cm^{-1} = 3184, 3127, 3111, 2977, 2937, 2874, 2774, 2210, 1707, 1552, 1460, 1397, 1371, 1341, 1274, 1233, 1214, 1157, 1125, 1100, 1061, 1027; ***trans*-7f:** ^{1}H NMR (400 MHz, $CDCl_3$) δ 5.28 (ddq, 1H), 2.60 (dd, *J* = 7.6, 14.3 Hz, 1H), 2.35 (dd, *J* = 5.6, 14.3 Hz, 1H), 1.62 (s, 6 H), 1.45 (d, *J* = 6.2 Hz, 3H); ^{13}C NMR (100 MHz, $CDCl_3$) δ 79.3, 59.5, 44.8, 28.4, 19.9; ***cis*-7f:** ^{1}H NMR (400 MHz, $CDCl_3$) δ 5.19 (m, 1H), 1.77 (m, 2H), 1.63 (s, 6H), 1.42 (d, *J* = 6.2 Hz, 3H); ^{13}C NMR (101 MHz, $CDCl_3$) δ 80.1, 59.6, 45.5, 28.4, 20.3.

3,3-Dimethyl-5-phenyl-1,2-oxathiolane 2-oxide (7g)

According to General Procedure D, alcohol **5g** (246 mg, 0.69 mmol), DBU (124 µl, 0.838 mmol) and MeCN (1.5ml) gave, following flash column chromatography (SiO_2, 40 % ether /petrol), the following *diastereomers*:

(2*R*,5*R*)-7g (*trans*, minor):

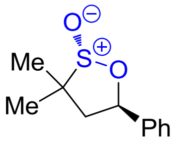

(11 mg; 8%) as colourless oil; mp = 53–56 °C; IR (neat) ν_{max}/cm^{-1} =2976, 2937, 2864, 1729, 1544, 1452, 1359, 1214, 1115, 1023; ^{1}H NMR (400 MHz, $CDCl_3$) δ 7.44–7.30 (m, 5H), 6.08 (dd, *J* = 7.0 & 7.0 Hz, 1H), 2.64 (dd, *J* = 12.7 & 7.0 Hz, 1H), 2.06 (dd, *J* = 12.7 & 7.0 Hz, 1H) 1.45 (s, 3H), 1.35 (s, 3H); ^{13}C NMR (100 MHz, $CDCl_3$) δ 139.3, 128.8, 128.4, 125.9, 87.8, 69.6, 45.4, 24.1, 21.3. HRMS (CI) calc'd for $C_{11}H_{15}O_2S$ $[M + H]^+$ 211.0787; found 211.0784. This compound was further characterised by x-ray crystallographic analysis. See section 12 (S65).

(2*S*,5*R*)-7g (*cis*, major)

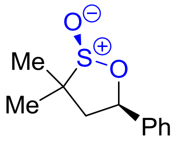

(56 mg; 39%) as colourless oil; mp = 84–85 °C; IR (neat) ν_{max}/cm^{-1} = 2933, 1720, 1602, 1547, 1494, 1456, 1370, 1236, 1154, 1118, 1074, 1026; ^{1}H NMR (400 MHz, $CDCl_3$) δ 7.45–7.28 (m, 5H), 5.61 (dd, *J* = 10.7 & 5.9, Hz, 1H), 2.41 (dd, *J* = 13.2 & 10.7 Hz, 1H), 2.26 (dd, *J* = 13.2 & 5.9 Hz, 1H), 1.44 (s, 3H), 1.40 (s, 3H); ^{13}C NMR (101 MHz, $CDCl_3$) δ 139.23, 128.8, 128.5, 127.4, 91.9, 69.9, 45.3, 20.5, 19.9. HRMS (CI) calc'd for $C_{11}H_{15}O_2S$ $[M + H]^+$ 211.0787; found 211.0787; Enantiomeric excess: > 99 % ee (Daicel IC column, ^{i}PrOH/hexane = 15/85, 30 °C, flow rate 1.0 ml/min, λ = 212 nm); t_R mj = 11. min, t_R mn = 19.50 min. This compound was further characterised by x-ray crystallographic analysis. See section 12 (S65).

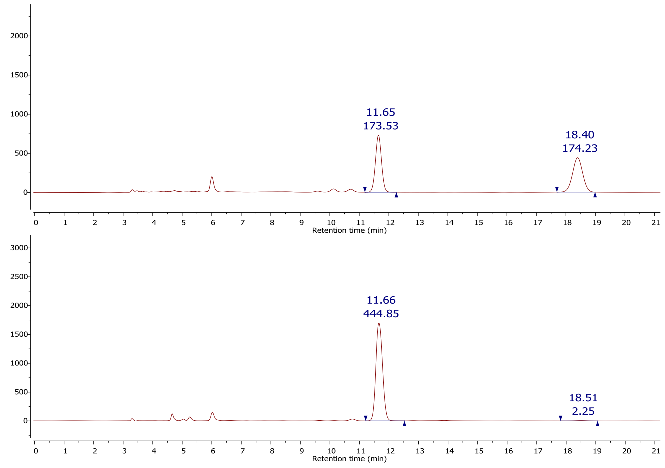

(±)- *cis*-7g

Peak	Area
11.65	173.53
18.40	174.23

(2*S*, 5*R*)-7g

Peak	Area
11.66	444.85
18.51	2.25

3,3-Diethyl-5-phenyl-1,2-oxathiolane 2-oxide (7h)

According to General Procedure D, alcohol **5h** (151 mg, 0.397 mmol), DBU (71 µl, 0.477 mmol) and MeCN (1 ml) gave, following flash column chromatography (SiO_2, 30 % Et_2O/petrol) the following *diastereomers:*

(2*R*,5*R*)-7h (*trans*, minor)

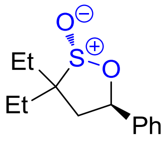

(10 mg, 11%) as colourless oil; IR (neat) ν_{max}/cm^{-1} = 2969, 2941, 1496, 1452, 1383, 1310, 1294, 1213, 1181, 1129, 1077, 1030, 1010; ^{1}H NMR (400 MHz, $CDCl_3$) δ 7.48–7.28 (m, 5H), 6.06 (dd, *J* = 7.3 & 7.3 Hz, 1H), 2.48 (dd, *J* = 12.8 & 7.3 Hz, 1H), 2.01–1.90 (m, 1H), 1.88–1.77 (m, 2H),

1.63–1.54 (m, 1H), 1.02 (t, *J* = 7.4 Hz, 3H), 0.95 (t, *J* = 7.4, 3H); ^{13}C NMR (100 MHz, $CDCl_3$) δ 139.2, 128.8, 128.4, 125.9, 87.3, 78.3, 42.1, 25.5, 23.1, 9.7, 8.9. HRMS (EI) calc'd for $C_{13}H_{19}O_2S$ $[M+H]^+$ 239.1100; found 239.1099.

(2*S*,5*R*)-7h (*cis*, major)

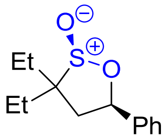

(32 mg, 34%) as colourless oil; IR (neat) v_{max}/cm^{-1} =2970, 1495, 1454, 1383, 1286, 1219, 1202, 1113, 1077, 1030; ^{1}H NMR (400 MHz, CDCl3) δ 7.45–7.27 (m, 5 H), 5.57 (dd, *J* = 6.0, 11.0 Hz, 1H), 2.37 (dd, *J* = 13.5 & 6.0 Hz, 1H), 2.20 (dd, *J* = 11.0, 13.5 Hz, 1H), 1.94 (dq, *J* = 14.9 & 7.5 Hz, 1H), 1.85 (dq, *J* = 14.9 & 7.5 Hz, 1H), 1.81 (dq, *J* = 14.9 & 7.5 Hz, 1H), 1.66 (dq, *J* = 14.9 & 7.5 Hz, 1H), 1.08 (t, *J* = 7.5 Hz, 3H), 1.02 (t, *J* = 7.5 Hz, 3H); ^{13}C NMR (101 MHz, $CDCl_3$) δ 139.5, 128.8, 128.4, 127.4, 91.9, 78.9, 42.0, 22.8, 20.9, 9.5, 8.7. HRMS (CI) calc'd for $C_{13}H_{19}O_2S$ $[M + H]^+$ 239.1100; found 239.1097.

(2*R,4*S**)-3,3-Dimethyl-4-phenyl-1,2-oxathiolane 2-oxide (7i)**

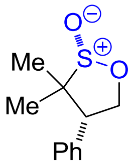

According to General Procedure D, alcohol **5i** (197 mg, 0.56 mmol), DBU (100 μl, 0.67 mmol) and MeCN (1.2 ml) gave, following flash column chromatography (SiO_2, 50% Et_2O/petrol), the *title compound* as white powder (76 mg, 65%); IR (neat) v_{max}/cm^{-1} = 2966, 2914, 2856, 1603, 1499, 1453, 1389, 1372, 1331, 1250, 1187, 1180, 1077; ^{1}H NMR (400 MHz, $CDCl_3$) δ 7.45–7.27 (m, 5H), 5.00 (dd, *J* = 8.5 & 8.5 Hz, 1H), 4.91 (dd, *J* = 9.5 & 8.5 Hz, 1H), 3.26 (dd, *J* = 9.5 & 8.5 Hz, 1H), 1.46 (s, 3H), 0.96 (s, 3H); ^{13}C NMR (100 MHz, $CDCl_3$) δ 136.4, 129.5, 128.8, 127.8, 79.5, 71.0, 55.6, 25.7, 18.4; HRMS calc'd for $C_{11}H_{15}O_2S$ $[M + H]^+$ 211.0787; found 211.0783. This compound was further characterised by x-ray crystallographic analysis. See section 12 (S65).

(2*R,4*S**)-3,3-Diethyl-4-phenyl-1,2-oxathiolane 2-oxide (7j)**

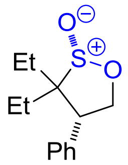

According to the General Procedure, alcohol **5j** (184 mg, 0.484 mmol) DBU (86 μl, 0.58 mmol) and MeCN (1 ml) gave, following flash column chromatography (50 % Et_2O/petrol) the *title compound* as clear oil (71 mg, 62%); IR (neat) v_{max}/cm^{-1} = 3061, 3028, 2968, 2940, 2879, 1738, 1602, 1493, 1454, 1382, 1327, 1220, 1127, 1112, 1084, 1062, 1033; ^{1}H NMR (400 MHz, $CDCl_3$) δ 7.45-7.27 (m, 5H), 5.03 (dd, *J* = 9.7 & 8.2 Hz, 1H), 4.92 (dd, *J* = 9.7 & 8.2 Hz, 1H), 3.34 (dd, *J* = 8.2 & 8.2 Hz, 1H), 1.97 (dq, *J* = 14.7 & 7.4 Hz, 1H), 1.67 (dq, *J* = 14.7 & 7.4 Hz, 1H), 1.57 (dq, *J* = 14.7 & 7.4 Hz, 1H), 1.14 (dq, *J* = 7.4, 14.7 Hz, 1H), 1.06 (t, *J* = 7.4 Hz, 3H), 0.81 (t, *J* = 7.4 Hz, 3H); ^{13}C NMR (100 MHz, $CDCl_3$) δ 137.3, 130.0, 128.5, 127.3, 81.6, 79.7, 51.9, 26.1, 21.4, 9.4, 8.0; HRMS calc'd for $C_{13}H_{19}O_2S$ $[M + H]^+$ 239.1100; found 239.1098.

3-((1-(*tert*-Butyl)-1*H*-tetrazol-5-yl)oxy)-4,4,4-trifluorobutane-1-sulfinic acid (9)

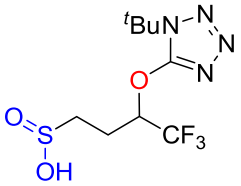

According to General Procedure D, alcohol **5d** (247 mg, 0.78 mmol), DBU (131 μl, 0.94 mmol) and MeCN (2 ml) gave without the need for further purification, the *title compound* **9** as a clear oil (304 mg, 99%) which decomposed within 24 h; IR (neat) v_{max}/cm^{-1} = 2963, 1498, 1465, 1379, 1260, 1221, 1122, 1029; ^{1}H NMR (400 MHz, $CDCl_3$) δ 8.49 (bs, 1H), 5.64 (bs, 1H), 3.03–2.91 (m, 2H), 2.60–2.39 (m, 2H), 1.67 (s, 9H); ^{13}C NMR (100 MHz, $CDCl_3$) δ 160.2, 123.2 (q, *J* = 282 Hz), 78.7 (d, *J* = 33 Hz), 61.7, 46.1, 28.3, 23.9. LRMS (CI) calc'd for $C_9H_{18}F_3N_5O_3S$ $[M + NH_3]^+$ 333.11, found 333.09.

6. Spectral data for reaction by-products

1-(*tert*-Butyl)-1*H*-tetrazole (TBTH) S2

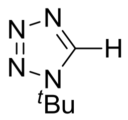

This by-product was observed via *in situ* NMR monitoring of sultone formation experiments. For characterisation an analytically pure sample was isolated: ^{1}H NMR (400 MHz, $CDCl_3$) δ 3.36 (s, 1H), 1.31 (s, 9H); ^{13}C NMR (101 MHz, $CDCl_3$) δ 114.4, 53.8, 29.3. HRMS calc'd for $C_5H_{11}N_4$ $[M + H]^+$ 127.0978; found 127.0976. These data matched those previously published.[16]

1-(*tert*-butyl)-1*H*-tetrazol-5(4*H*)-one S3

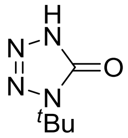

This by-product was observed via *in situ* NMR monitoring of sultine formation experiments. An authentic sample was prepared as previously reported[17] for comparison: to an ice cold suspension of NaN_3 (4.57 g) in dry THF (13 ml), anhydrous $AlCl_3$ (3.65 g) was added under Ar with stirring. The mixture was heated under reflux for 3 h, after which ^{t}BuNCO (2.47 g) was added. The mixture was heated under reflux for a further 20 h before it was cooled on ice and conc. HCl (5 ml) was added. The mixture was concentrated *in vacuo* and 10 ml H_2O added. Cooling resulted in the formation of off-white needles (1.485 g, 10%) that were collected by suction filtration and dried in a vacuum desiccator; mp 102–103 °C, ^{1}H NMR (400 MHz, $CDCl_3$) δ 13.67 (bs, 1H), 1.66 (s, 9H); ^{13}C NMR (100 MHz, $CDCl_3$) δ 153.7, 59.5, 27.9.

(16) K. Nishiyama, M. Oba, A. Watnabe, *Tetrahedron*, **1987**, *43*, 693.
(17) H. Quast, L. Bieber, *Chemische Berichte*, **1981**, *114*, 3253.

7. ^{1}H and ^{13}C NMR data for heteroarylalkylsulfones 1

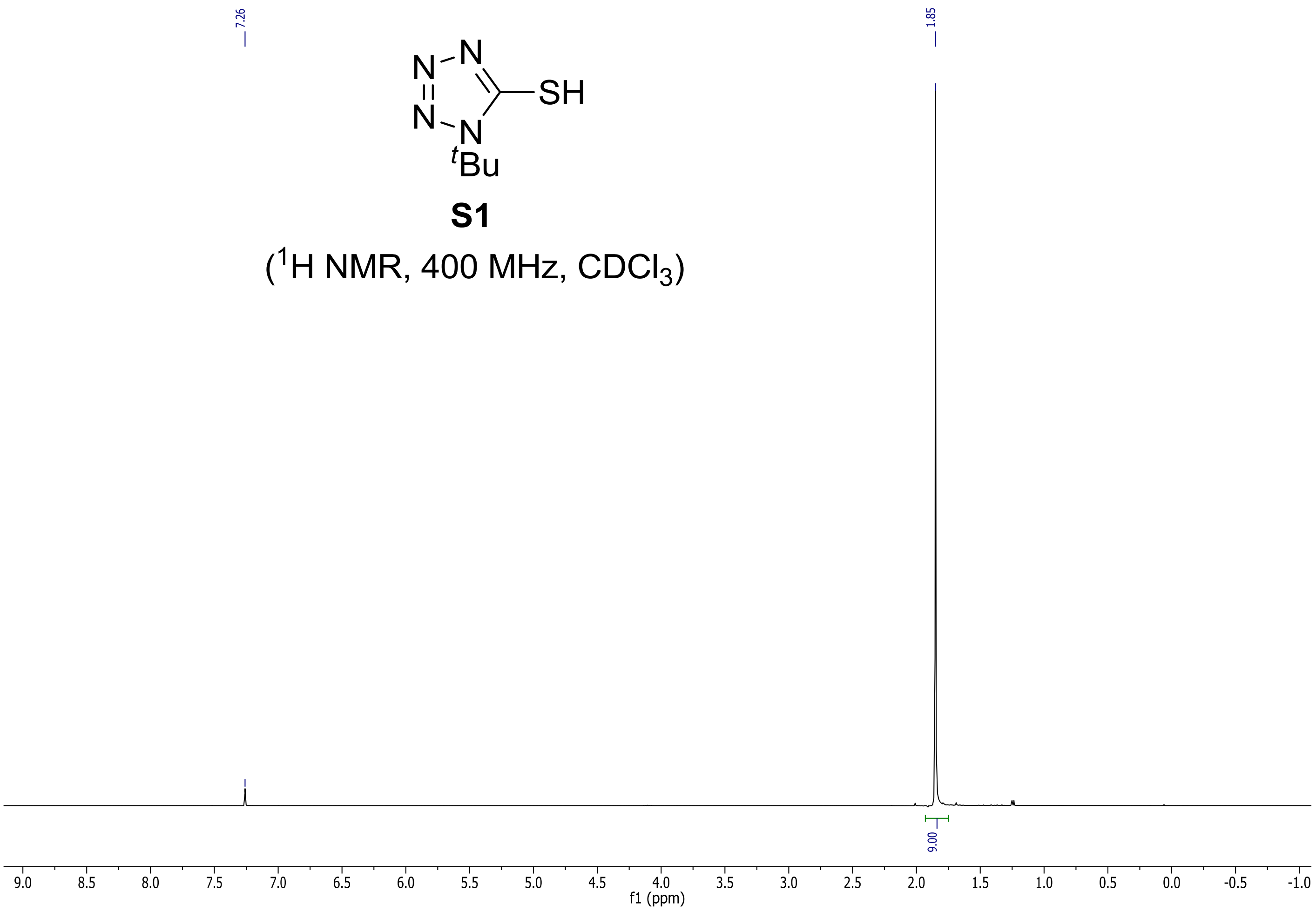

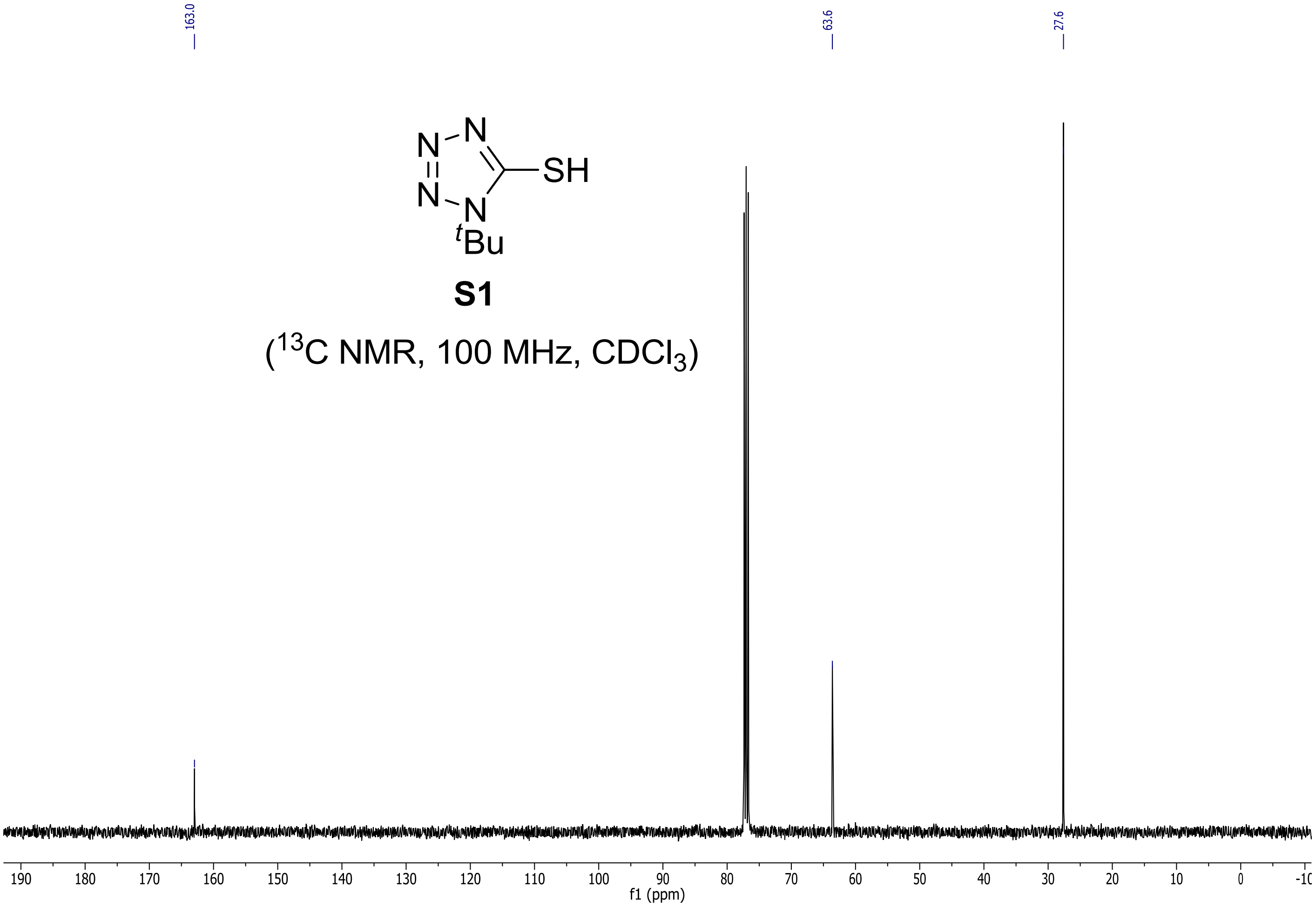

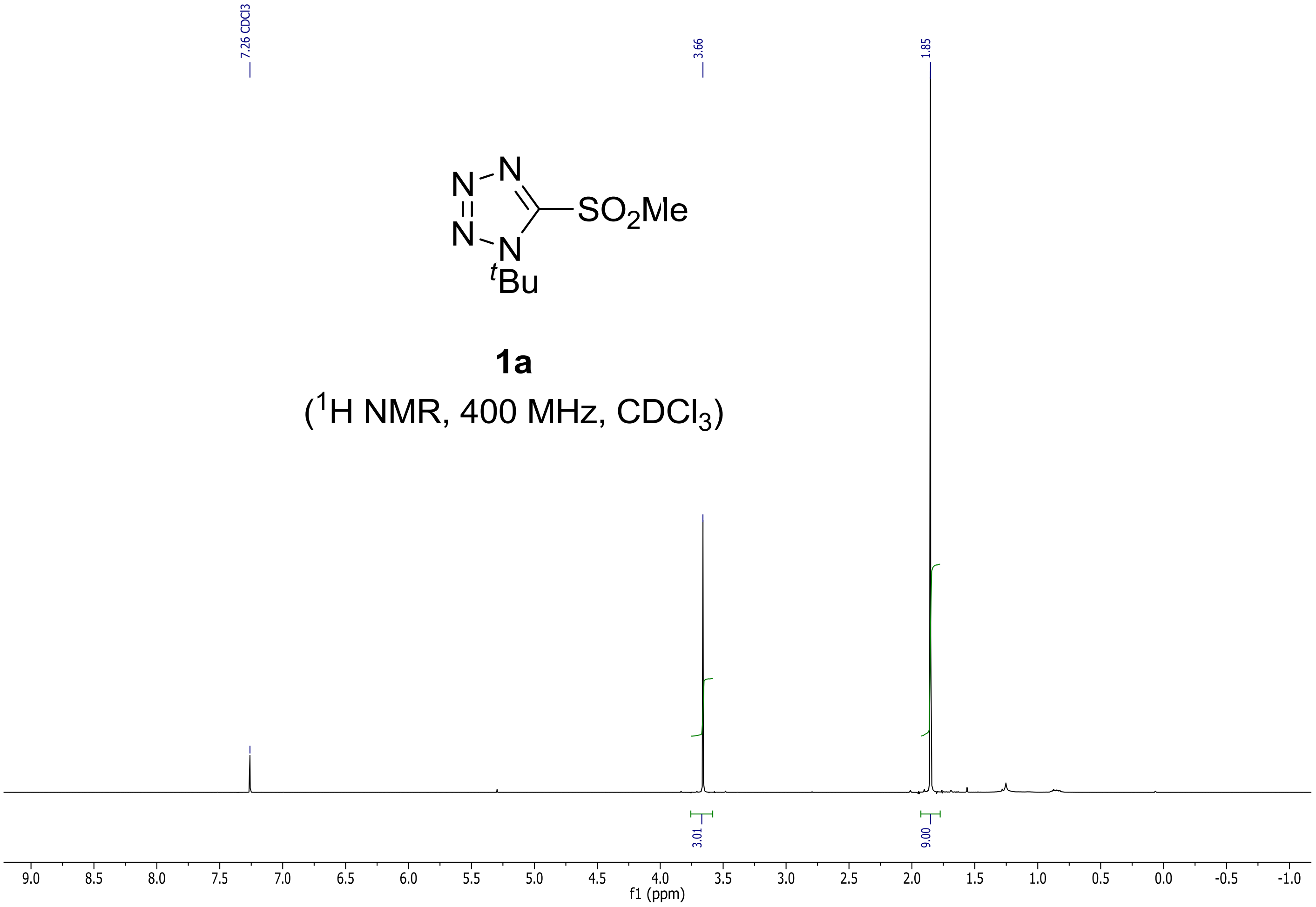
7.26 CDCl3
3.66
1.85
SO2Me
tBu
1a
(1H NMR, 400 MHz, CDCl3)
3.01
9.00
f1 (ppm)

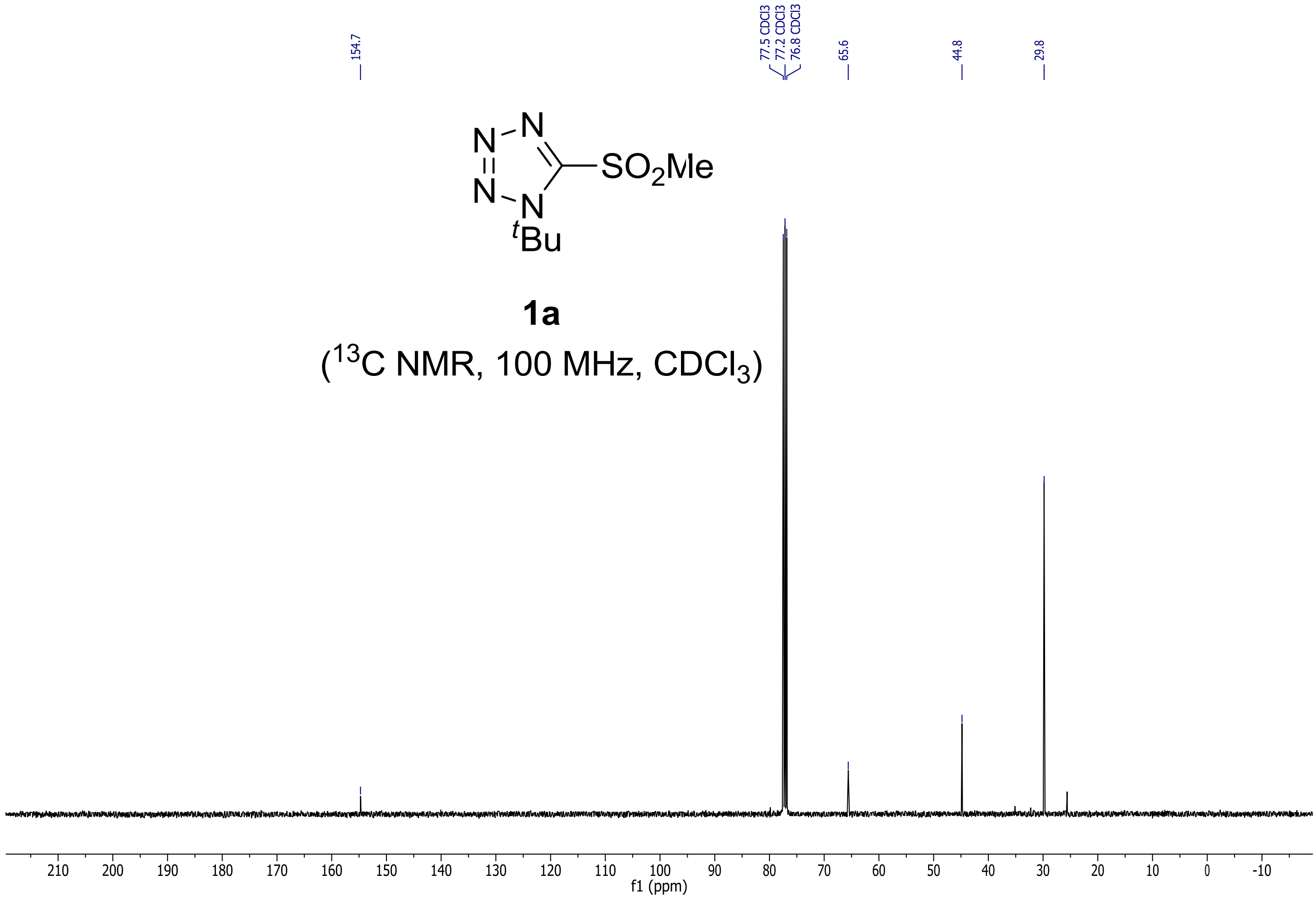
154.7
77.5 CDCl3
77.2 CDCl3
76.8 CDCl3
65.6
44.8
29.8
SO2Me
tBu
1a
(13C NMR, 100 MHz, CDCl3)
f1 (ppm)

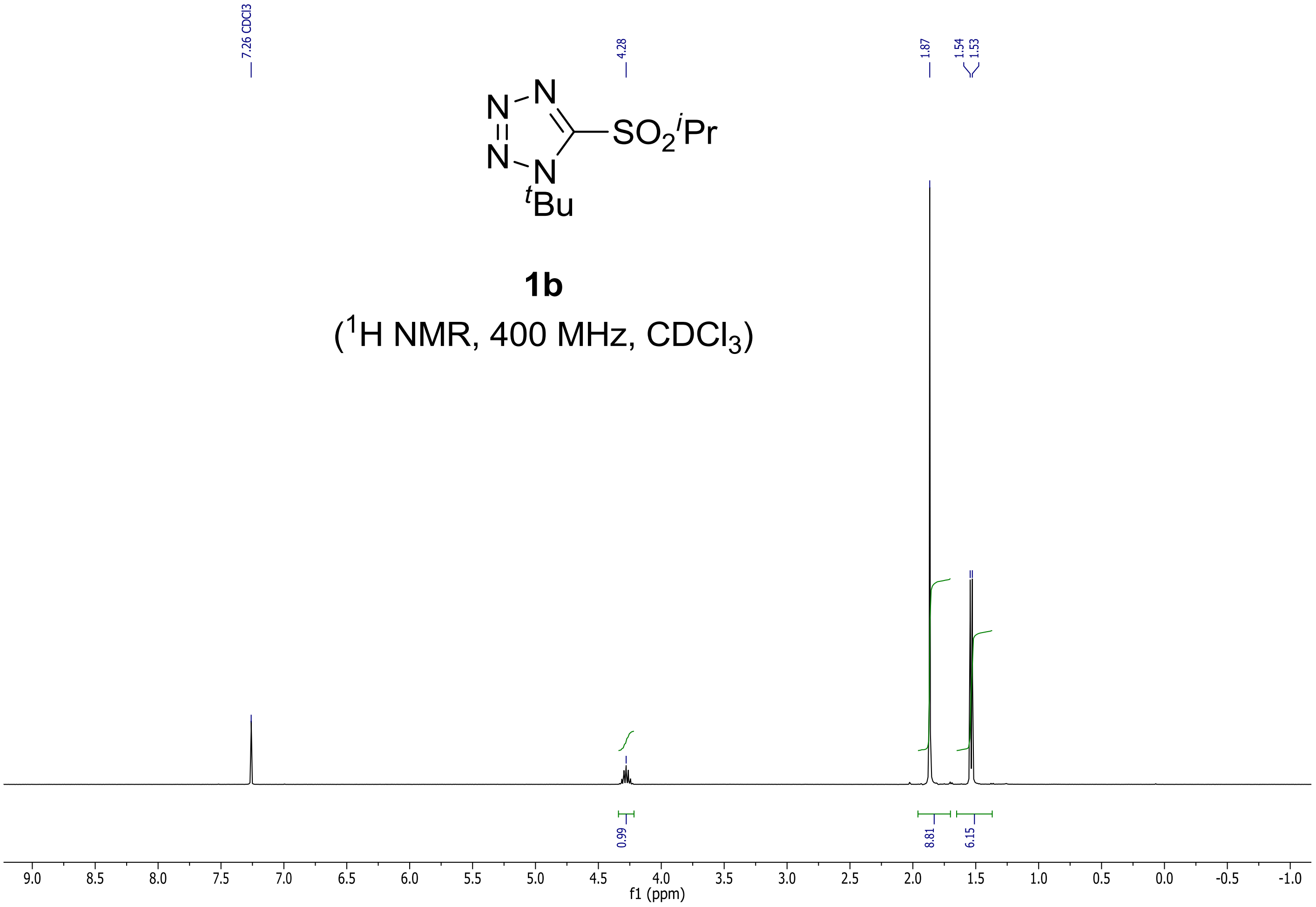

7.26 CDCl3
4.28
1.87
1.54
1.53
SO2iPr
tBu
1b
(1H NMR, 400 MHz, CDCl3)
0.99
8.81
6.15
9.0 8.5 8.0 7.5 7.0 6.5 6.0 5.5 5.0 4.5 4.0 3.5 3.0 2.5 2.0 1.5 1.0 0.5 0.0 -0.5 -1.0
f1 (ppm)

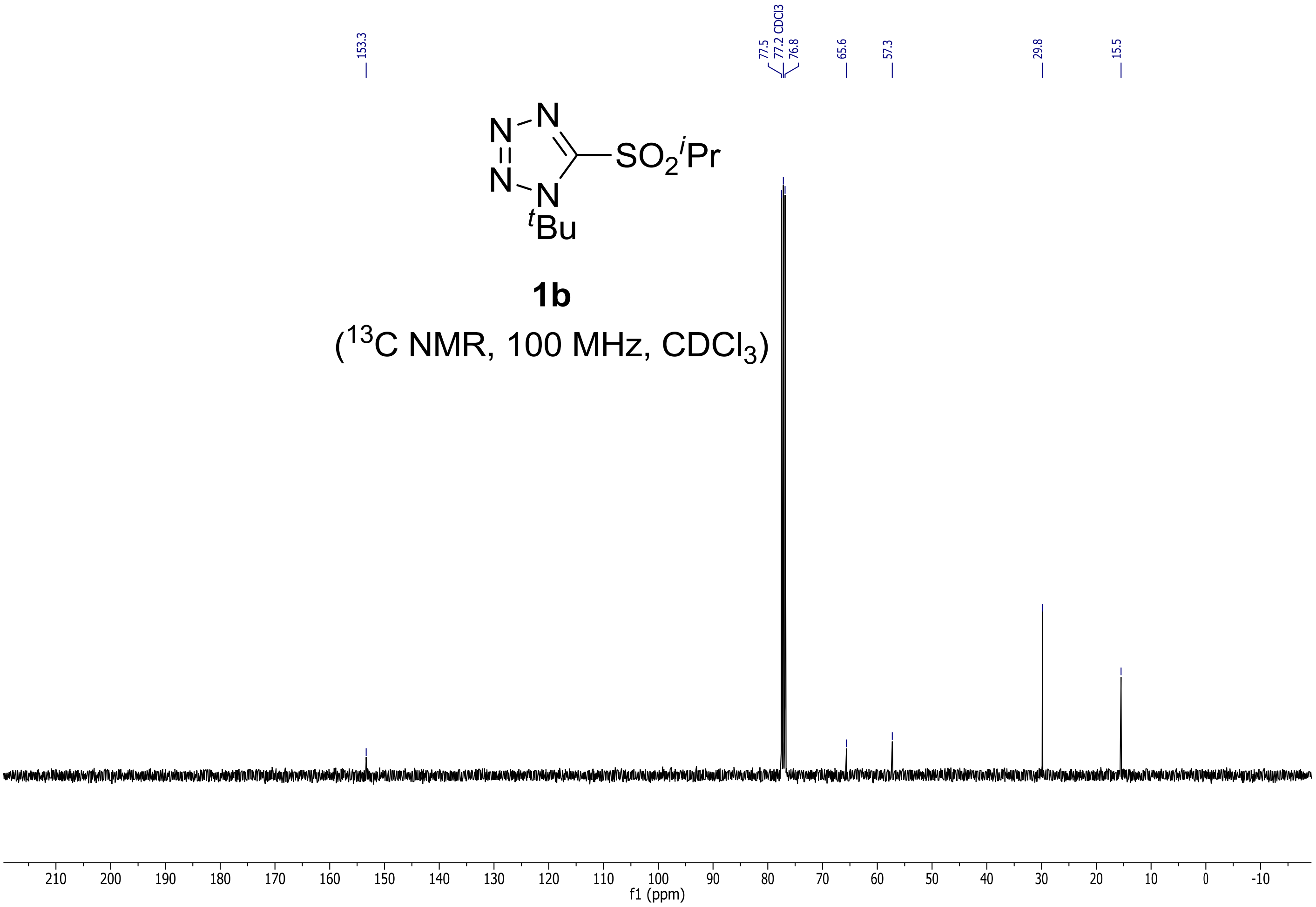

153.3
77.5
77.2 CDCl3
76.8
65.6
57.3
29.8
15.5
SO2iPr
tBu
1b
(13C NMR, 100 MHz, CDCl3)
210 200 190 180 170 160 150 140 130 120 110 100 90 80 70 60 50 40 30 20 10 0 -10
f1 (ppm)

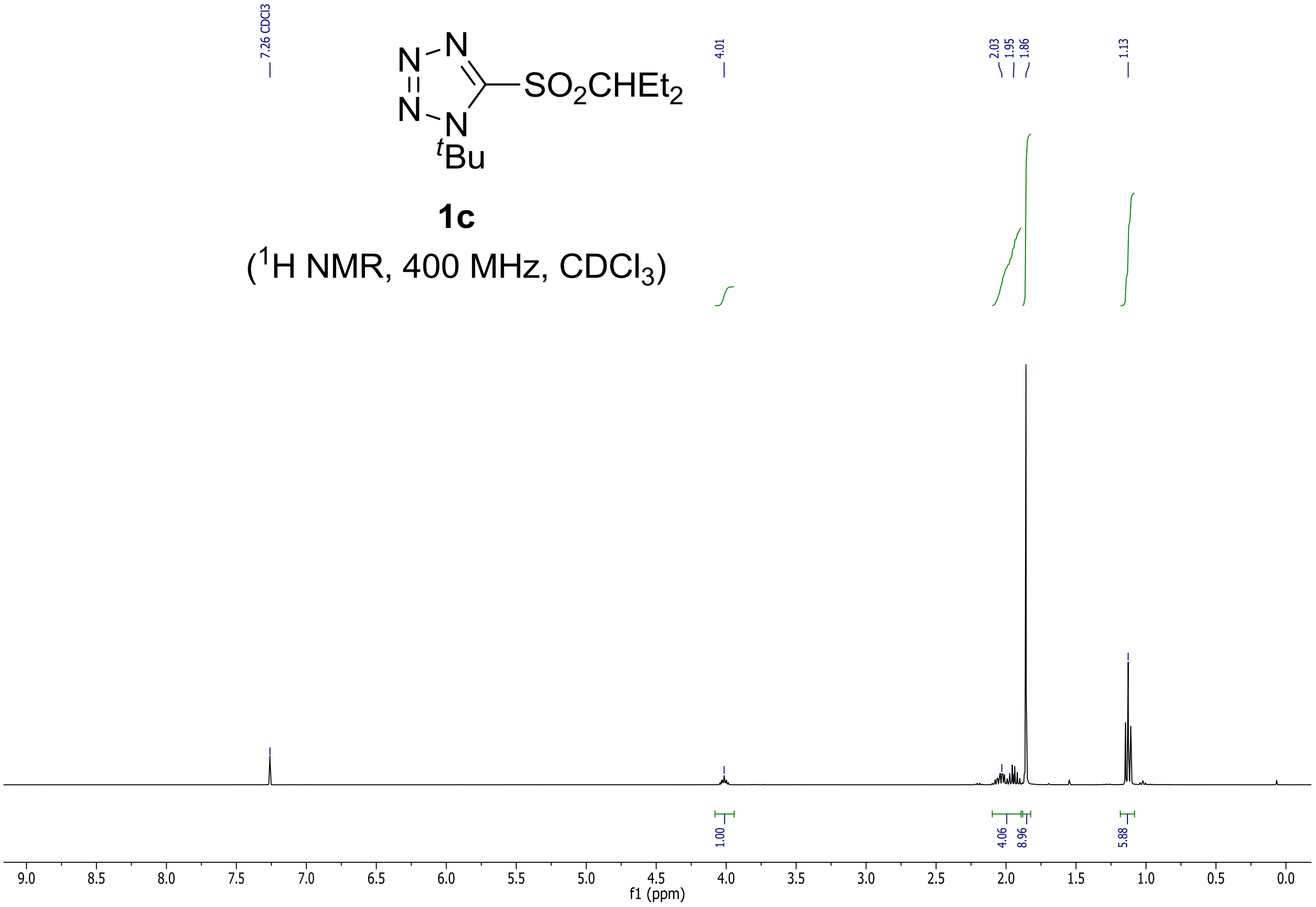
7.26 CDCl3
4.01
2.03
1.95
1.86
1.13
SO2CHEt2
tBu
1c
(1H NMR, 400 MHz, CDCl3)
1.00
4.06
8.96
5.88
9.0 8.5 8.0 7.5 7.0 6.5 6.0 5.5 5.0 4.5 4.0 3.5 3.0 2.5 2.0 1.5 1.0 0.5 0.0
f1 (ppm)

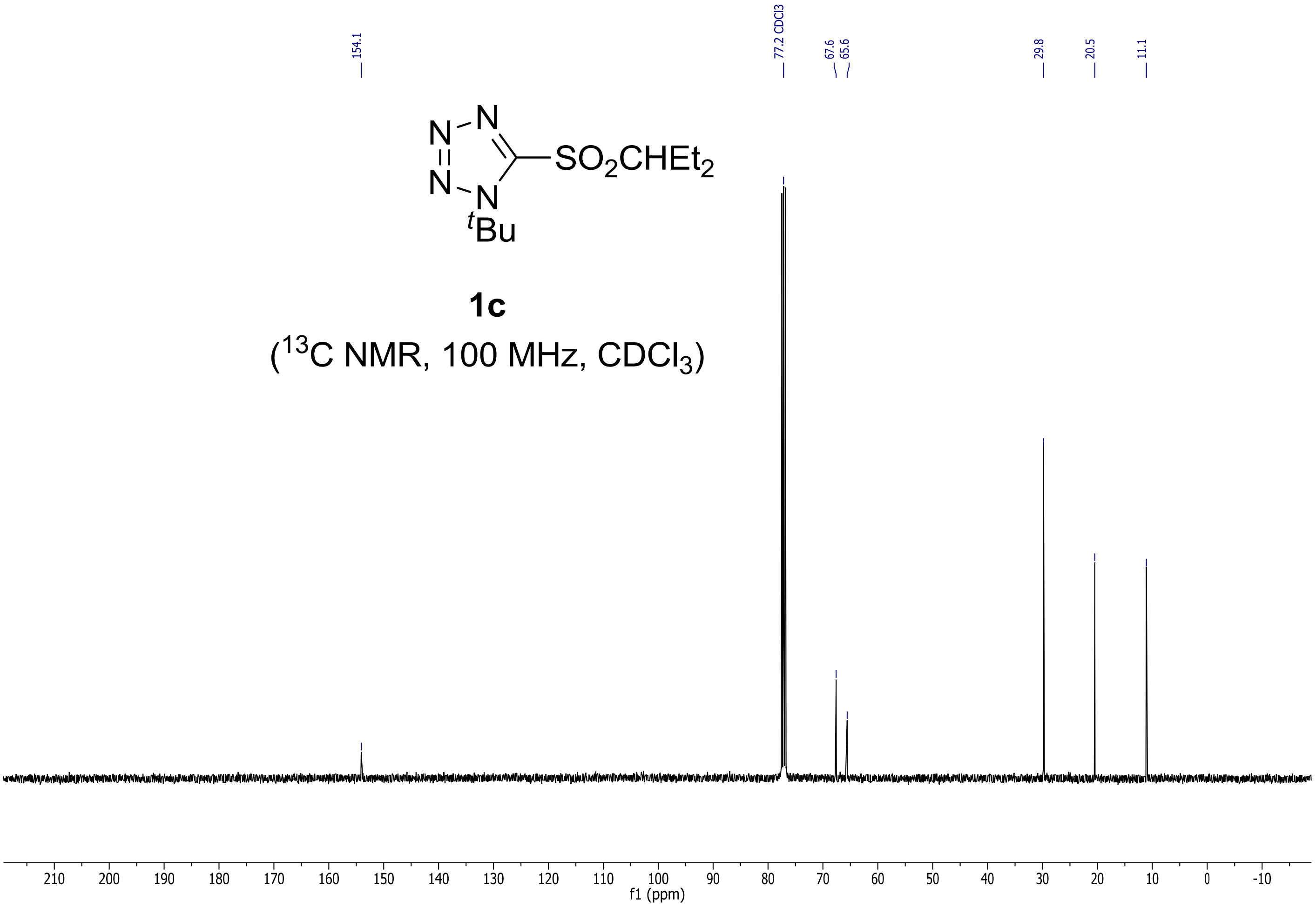
154.1
77.2 CDCl3
67.6
65.6
29.8
20.5
11.1
SO2CHEt2
tBu
1c
(13C NMR, 100 MHz, CDCl3)
210 200 190 180 170 160 150 140 130 120 110 100 90 80 70 60 50 40 30 20 10 0 -10
f1 (ppm)

8. ^{1}H and ^{13}C NMR data for sultones 4a-v

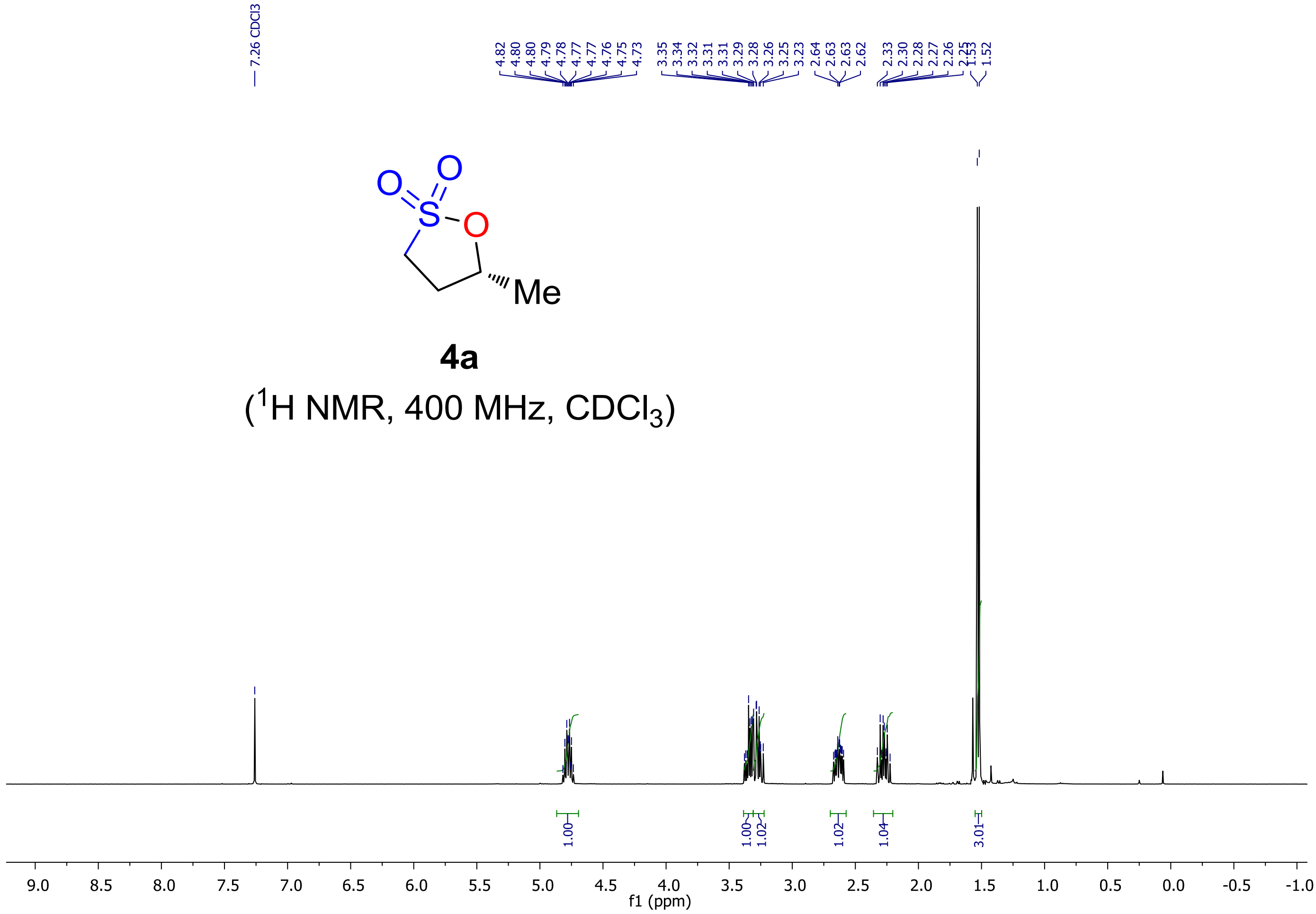

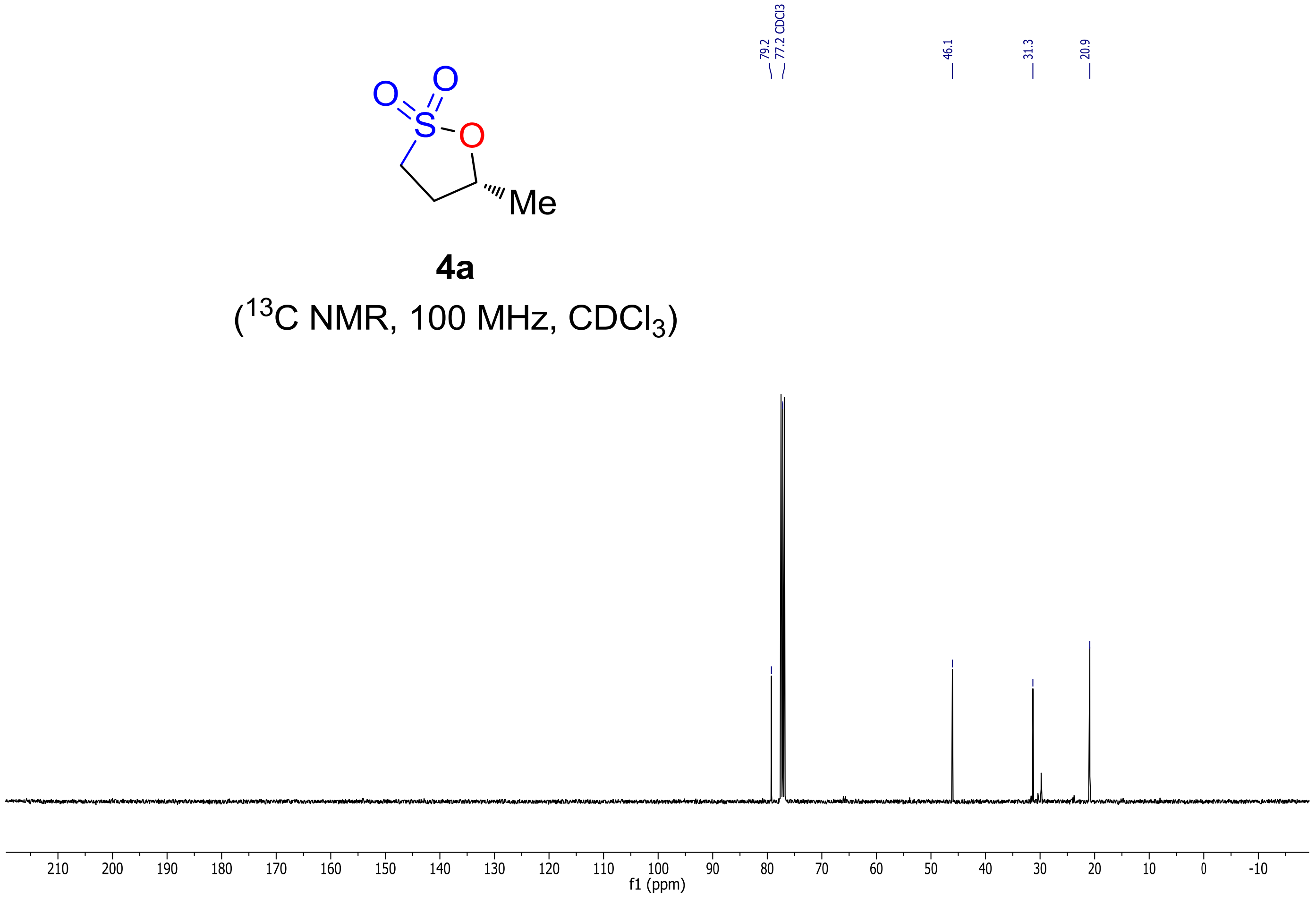

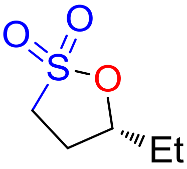

4b

(^{1}H NMR, 400 MHz, $CDCl_3$)

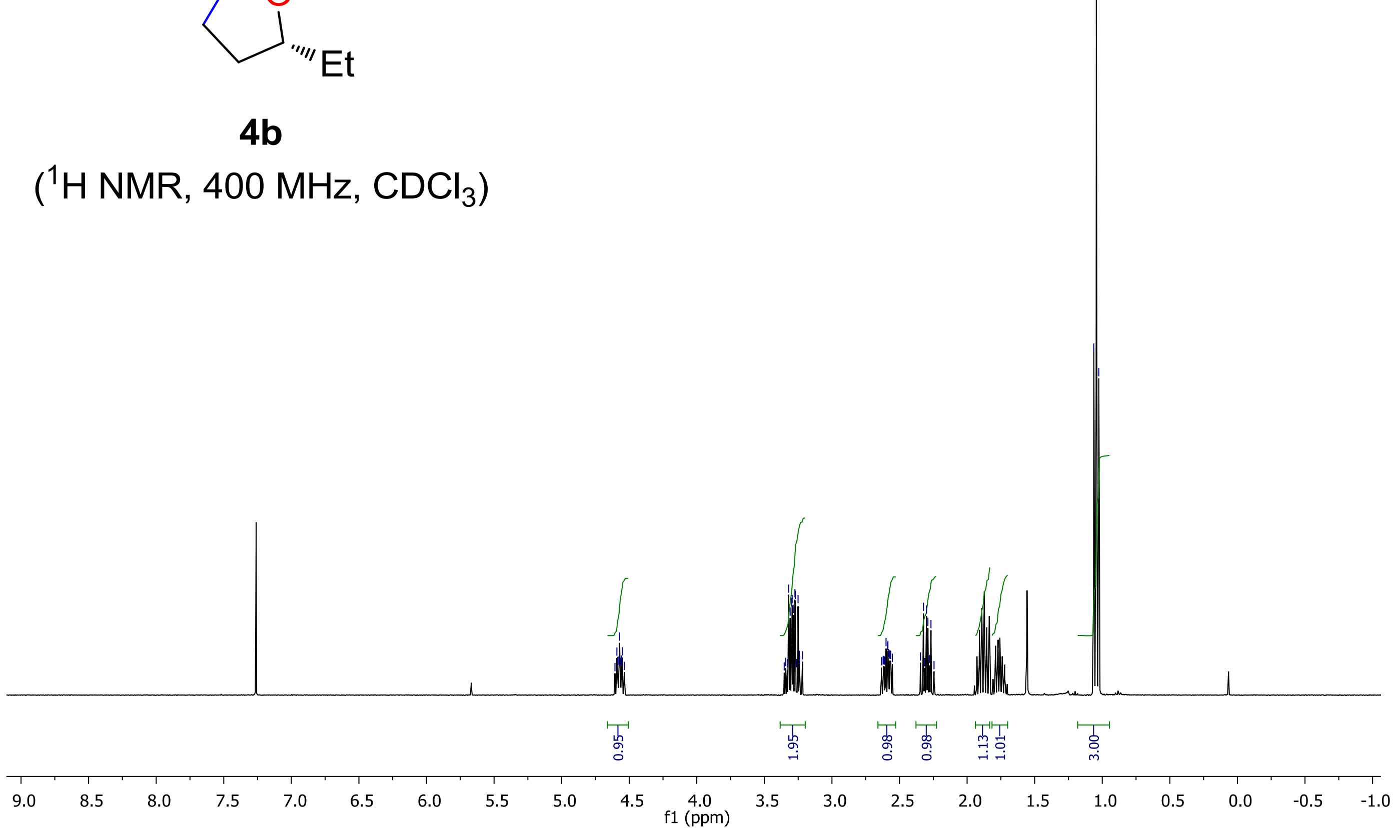

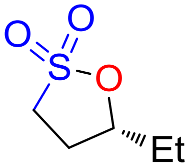

4b

(^{13}C NMR, 100 MHz, $CDCl_3$)

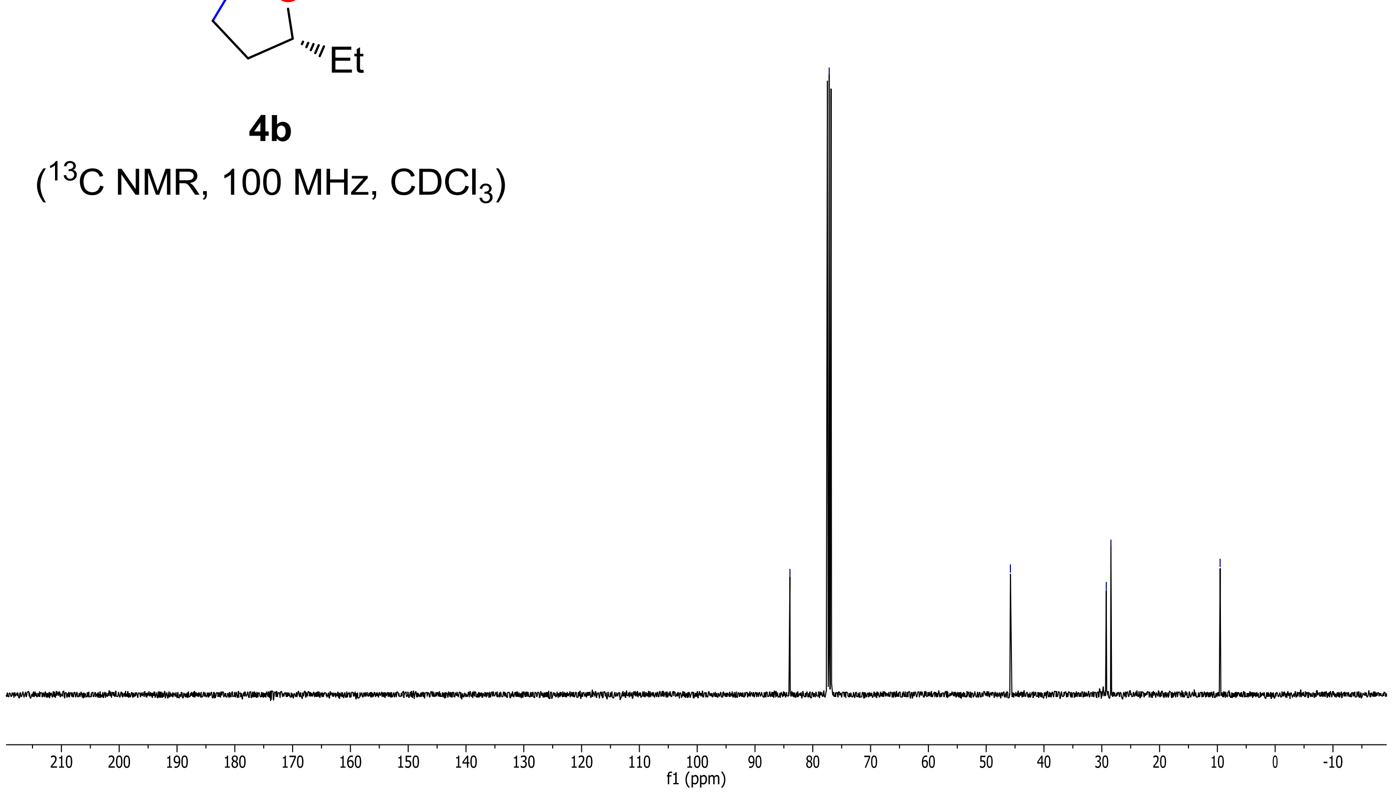

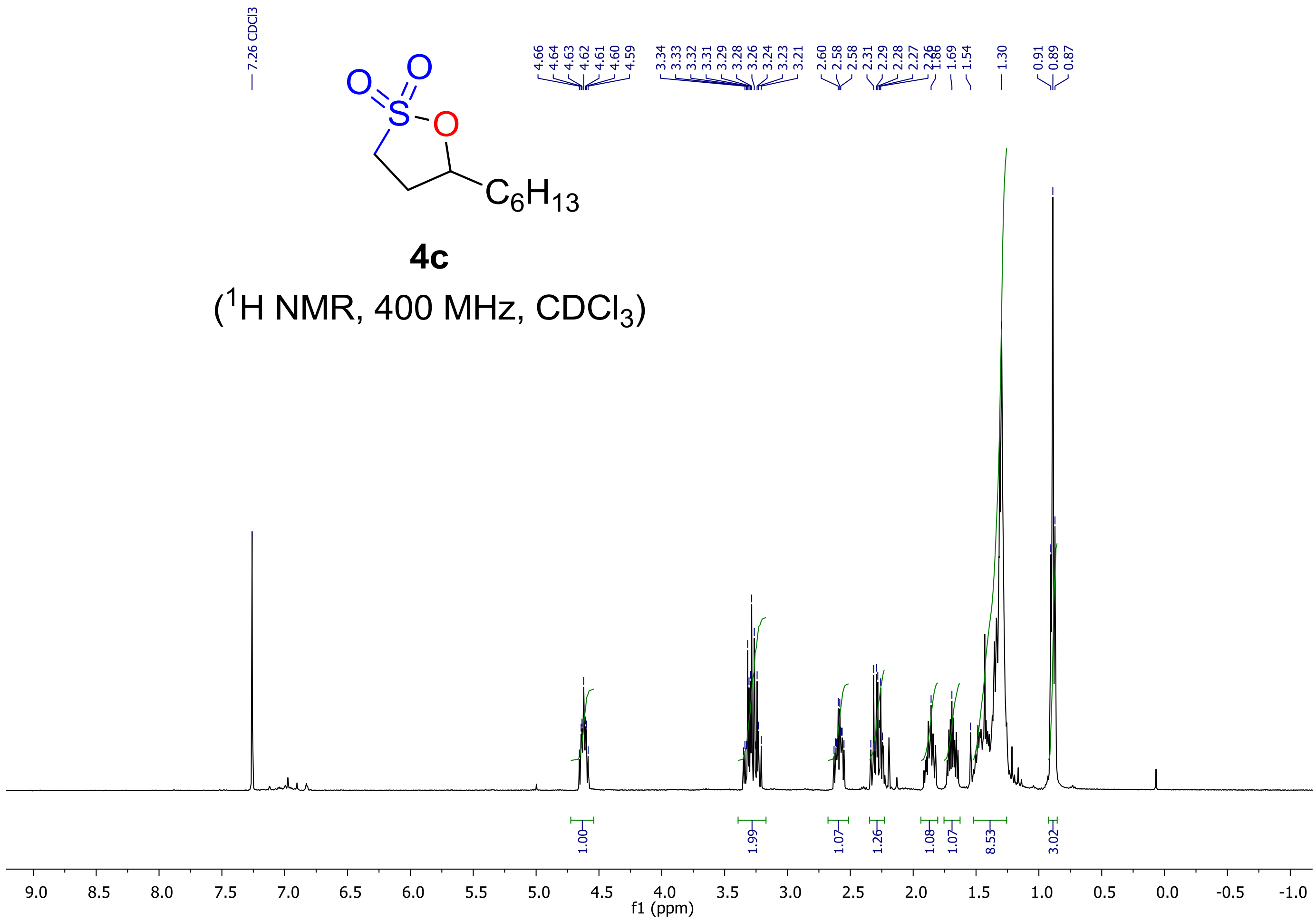

4c

(^{1}H NMR, 400 MHz, $CDCl_3$)

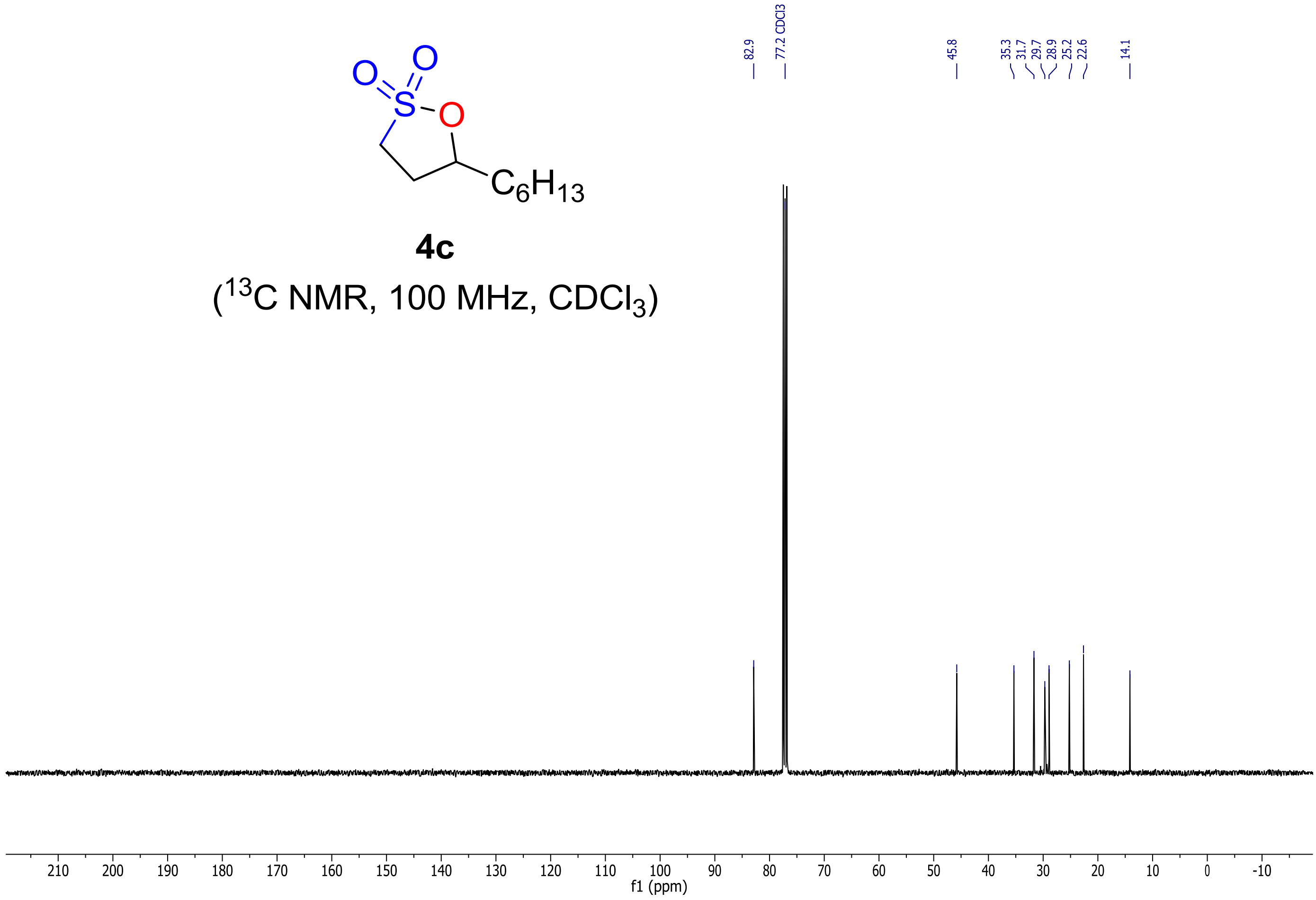

4c

(^{13}C NMR, 100 MHz, $CDCl_3$)

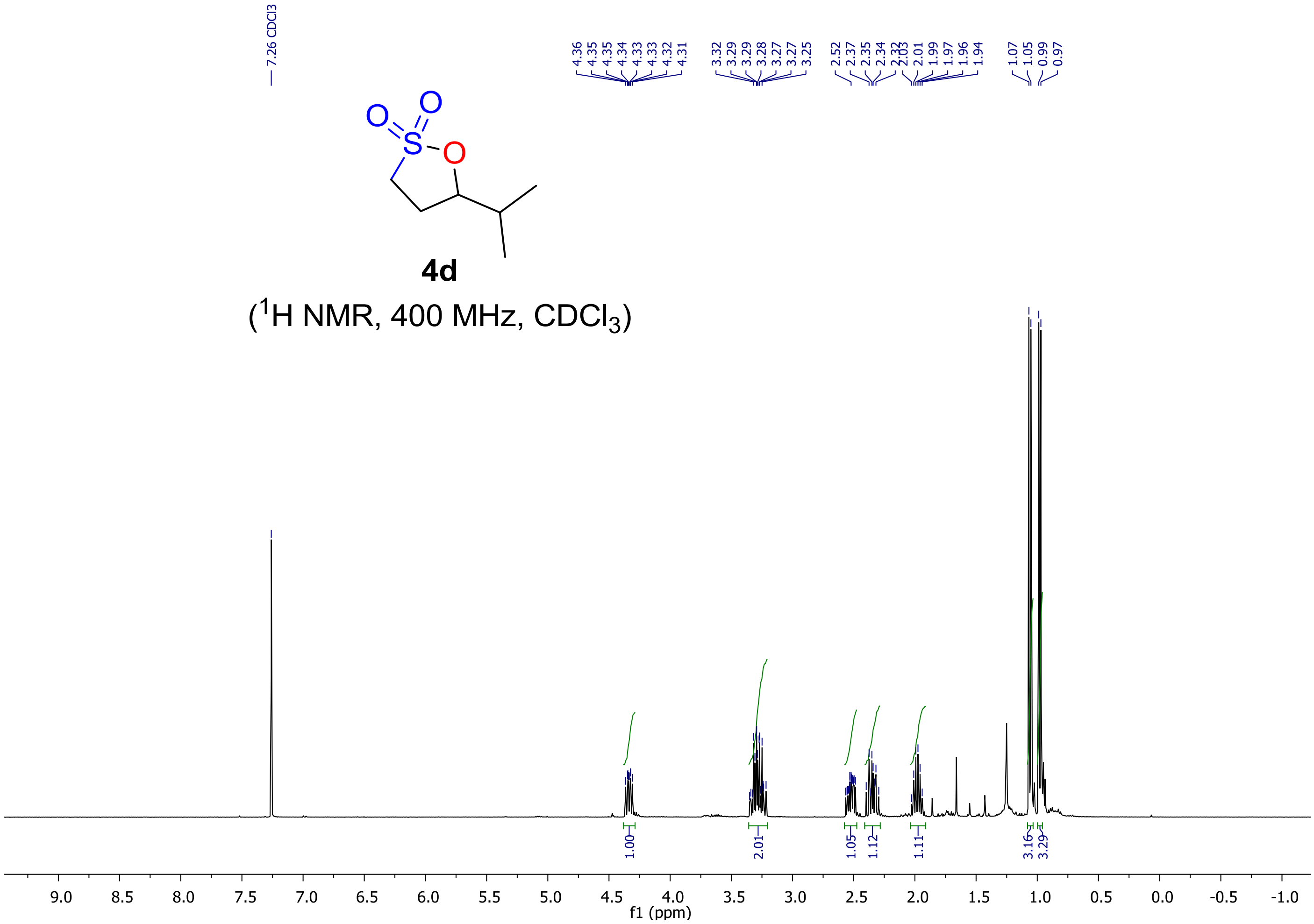
7.26 CDCl3
4.36
4.35
4.35
4.34
4.33
4.33
4.32
4.31
3.32
3.29
3.29
3.28
3.27
3.27
3.25
2.52
2.37
2.35
2.34
2.32
2.03
2.01
1.99
1.97
1.96
1.94
1.07
1.05
0.99
0.97
4d
(1H NMR, 400 MHz, CDCl3)
1.00
2.01
1.05
1.12
1.11
3.16
3.29
9.0
8.5
8.0
7.5
7.0
6.5
6.0
5.5
5.0
4.5
4.0
3.5
3.0
2.5
2.0
1.5
1.0
0.5
0.0
-0.5
-1.0
f1 (ppm)

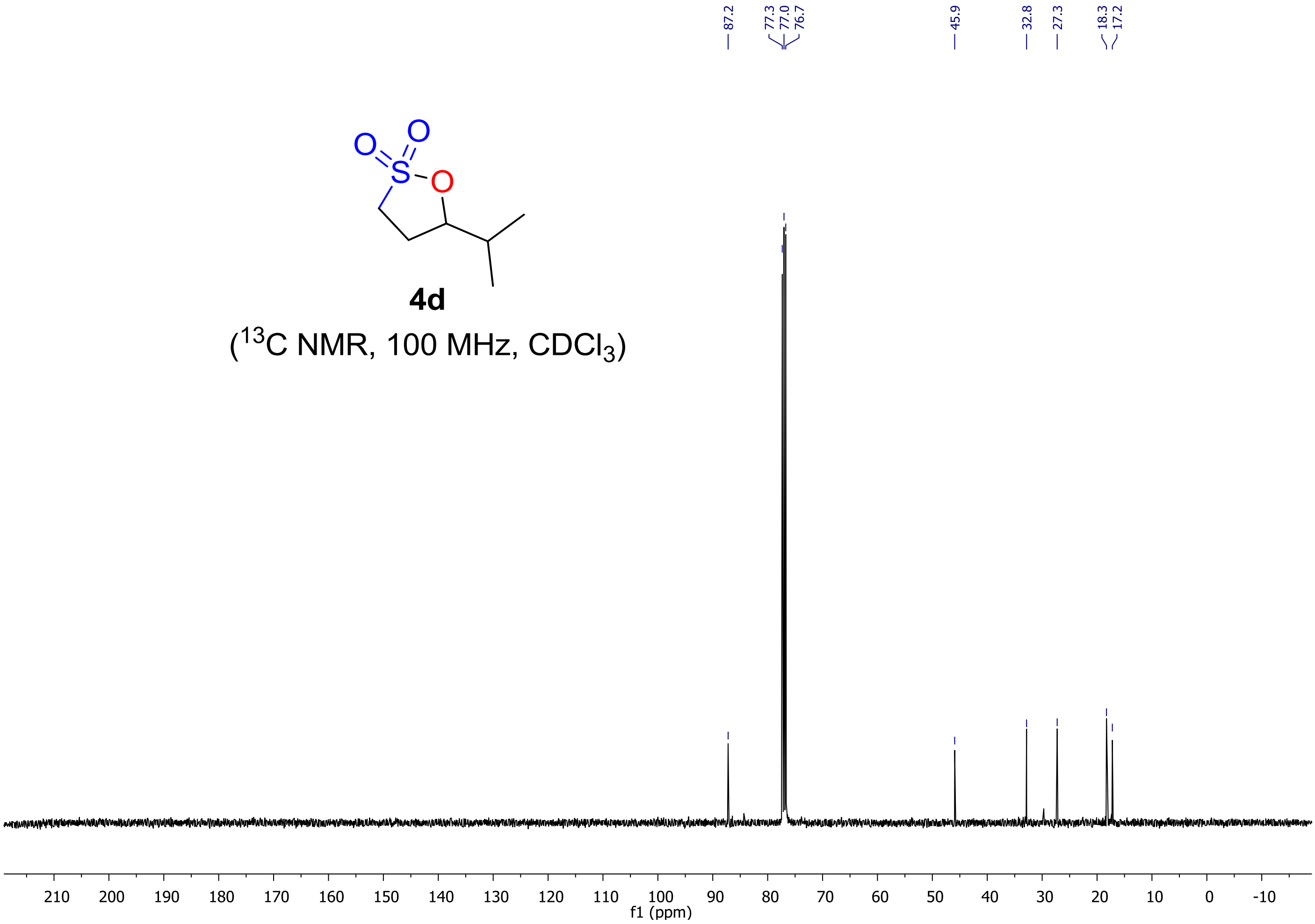
87.2
77.3
77.0
76.7
45.9
32.8
27.3
18.3
17.2
4d
(13C NMR, 100 MHz, CDCl3)
210
200
190
180
170
160
150
140
130
120
110
100
90
80
70
60
50
40
30
20
10
0
-10
f1 (ppm)

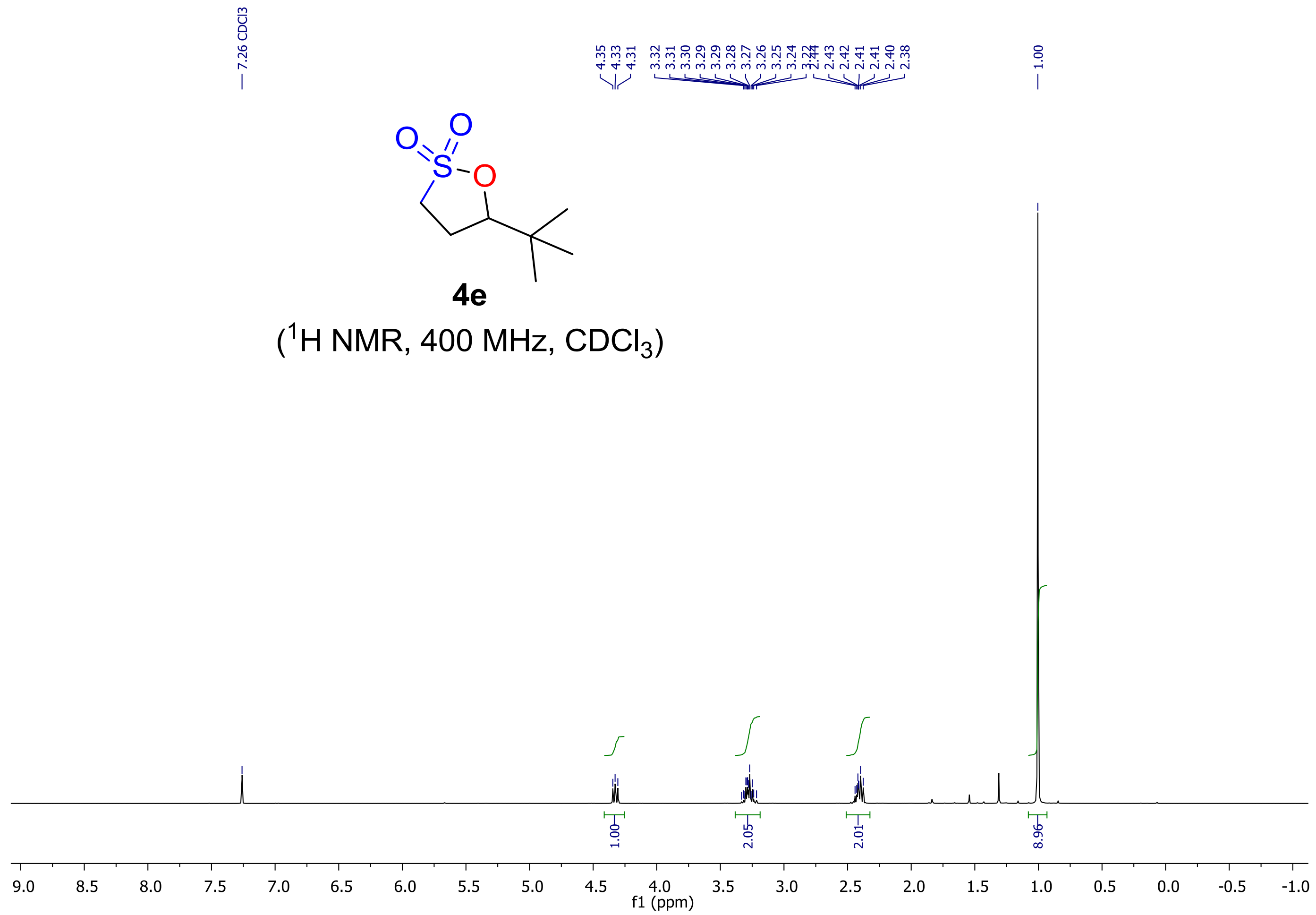
7.26 CDCl3
4.35
4.33
4.31
3.32
3.31
3.30
3.29
3.29
3.28
3.27
3.26
3.25
3.24
3.22
2.44
2.43
2.42
2.41
2.41
2.40
2.38
1.00
4e
(^{1}H NMR, 400 MHz, $CDCl_3$)
1.00
2.05
2.01
8.96
9.0
8.5
8.0
7.5
7.0
6.5
6.0
5.5
5.0
4.5
4.0
3.5
3.0
2.5
2.0
1.5
1.0
0.5
0.0
-0.5
-1.0
f1 (ppm)

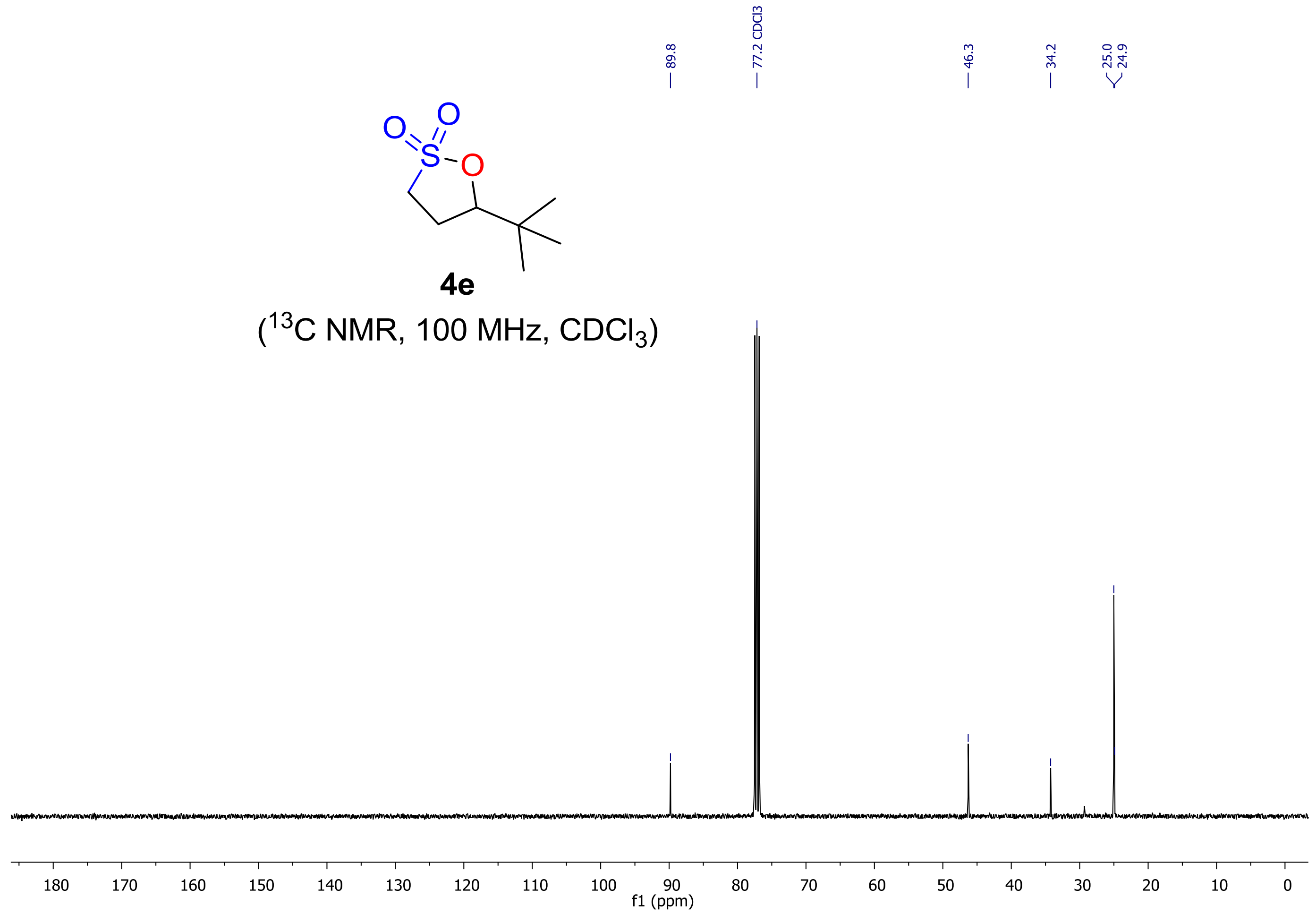
89.8
77.2 CDCl3
46.3
34.2
25.0
24.9
4e
(^{13}C NMR, 100 MHz, $CDCl_3$)
180
170
160
150
140
130
120
110
100
90
80
70
60
50
40
30
20
10
0
f1 (ppm)

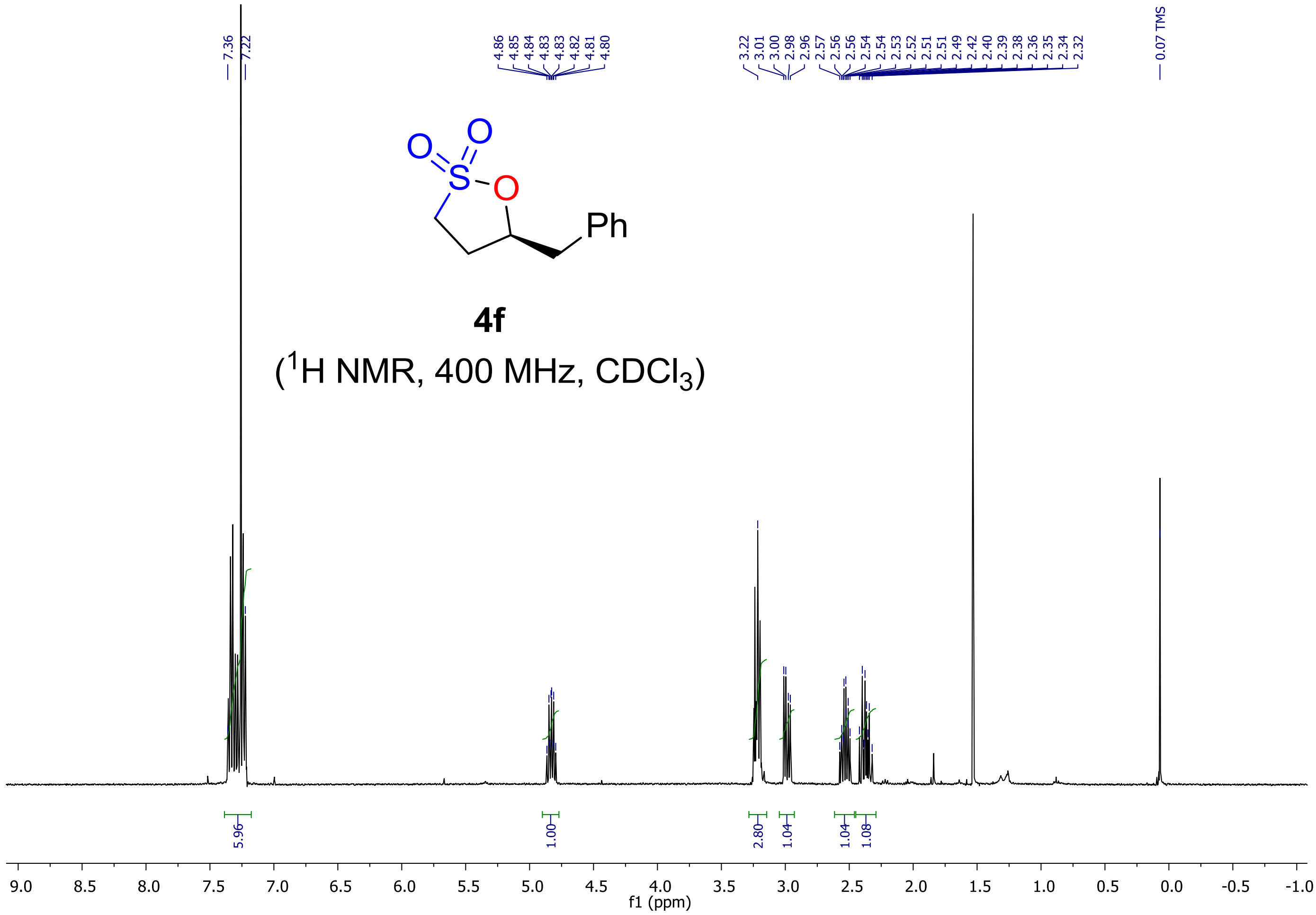

4f

(^{1}H NMR, 400 MHz, $CDCl_3$)

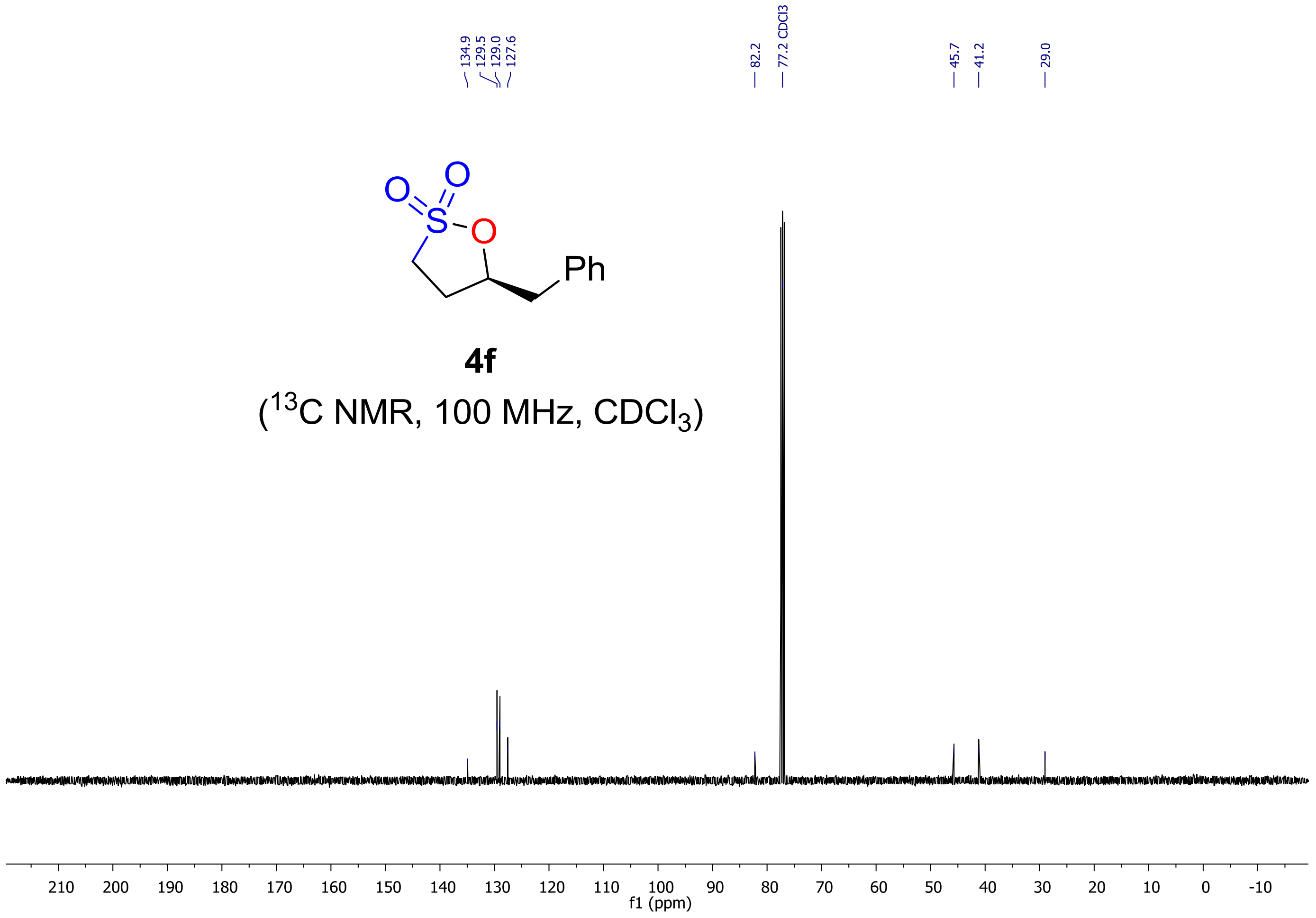

4f

(^{13}C NMR, 100 MHz, $CDCl_3$)

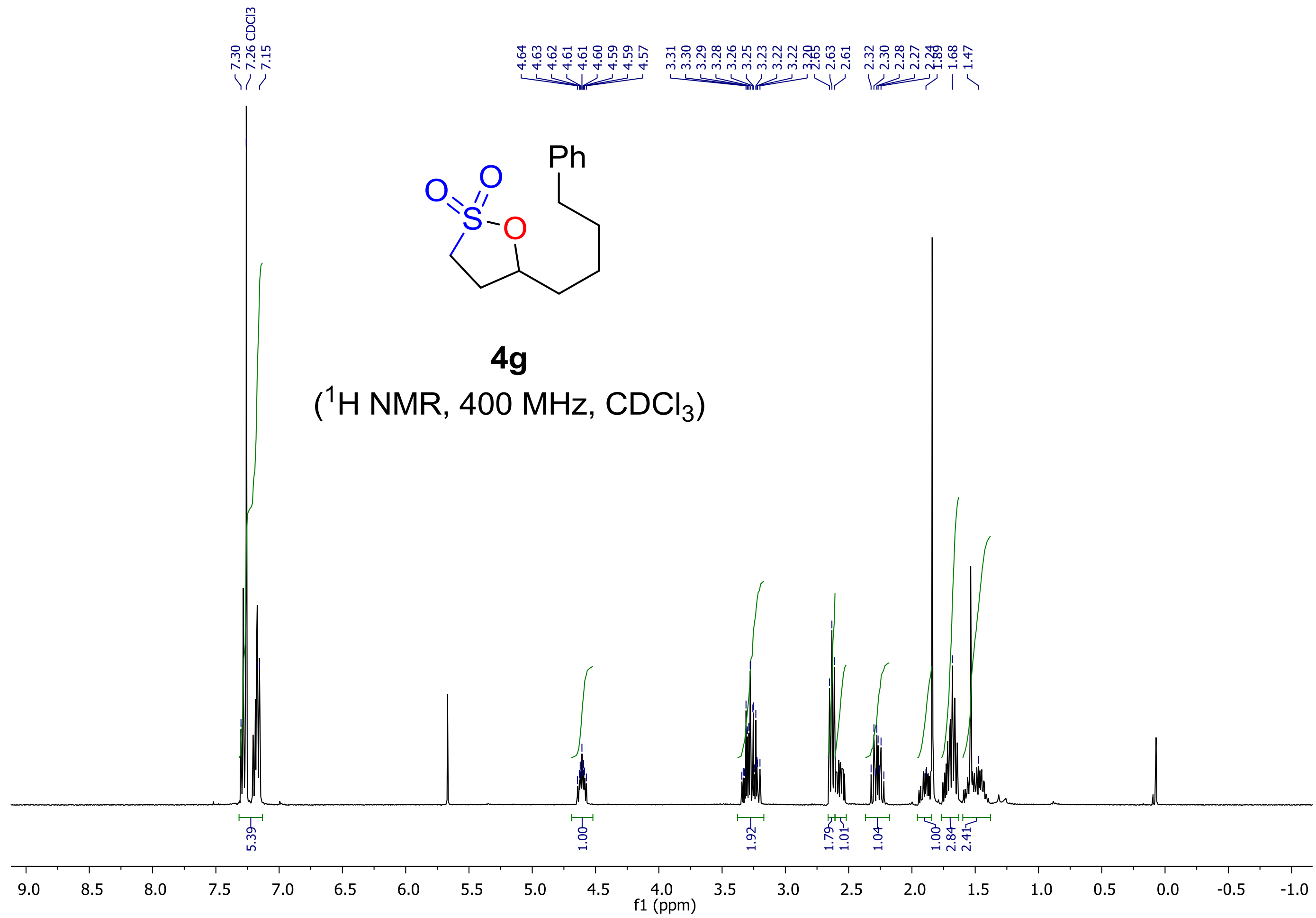
Ph
4g
(1H NMR, 400 MHz, CDCl3)
f1 (ppm)

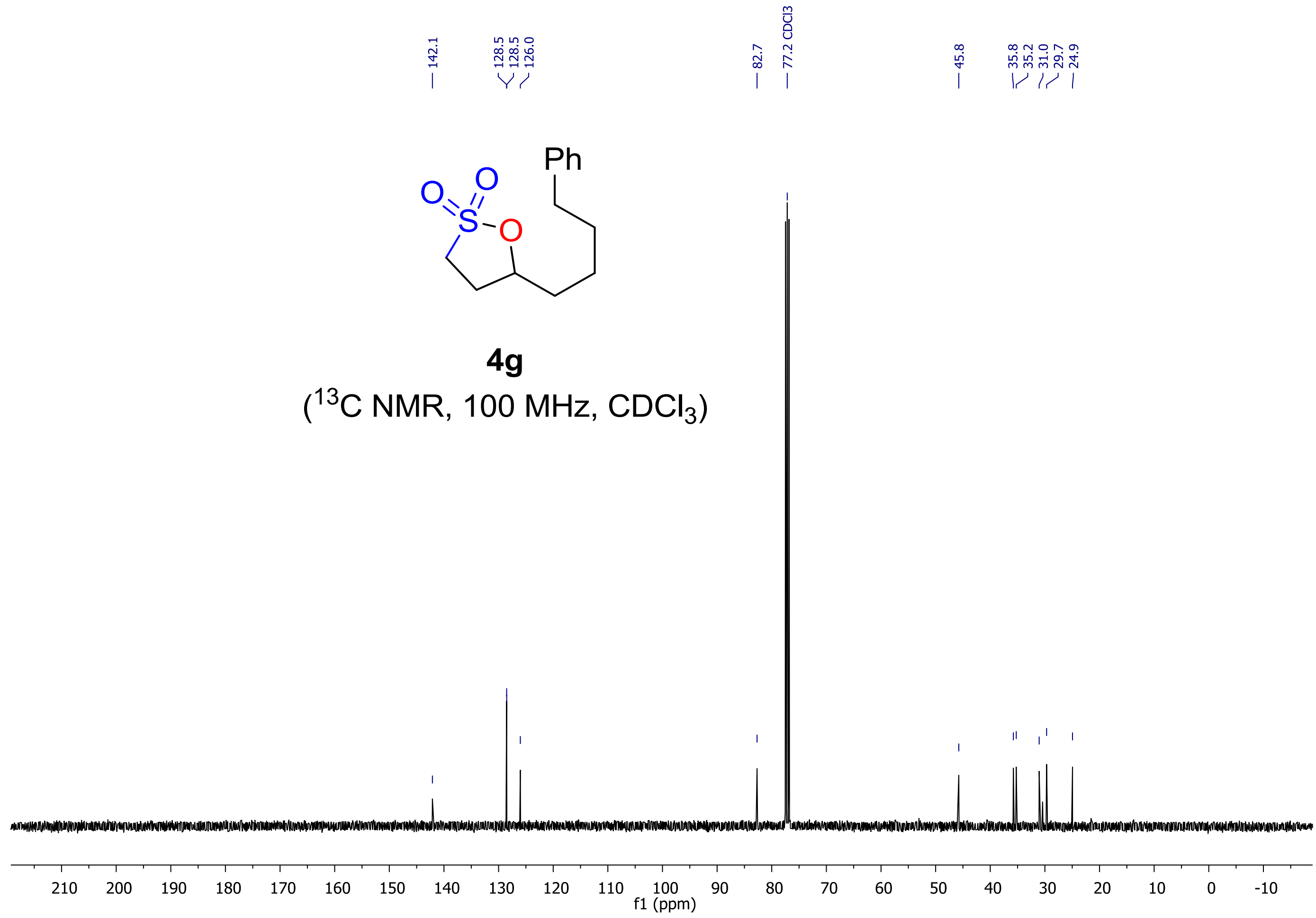
142.1
128.5
128.5
126.0
82.7
77.2 CDCl3
45.8
35.8
35.2
31.0
29.7
24.9
Ph
4g
(13C NMR, 100 MHz, CDCl3)
f1 (ppm)

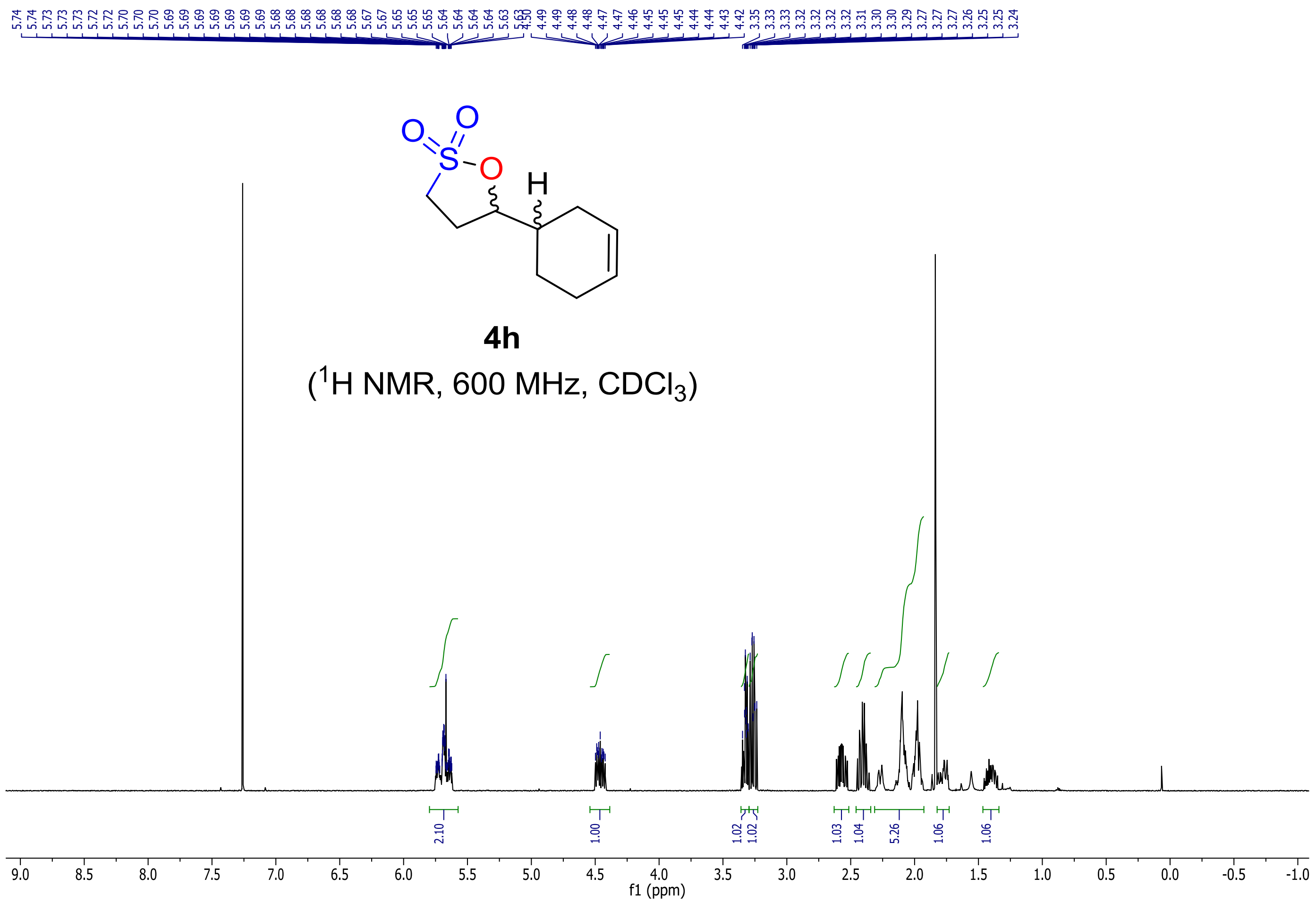
4h
(1H NMR, 600 MHz, CDCl3)
2.10
1.00
1.02
1.02
1.03
1.04
5.26
1.06
1.06
f1 (ppm)

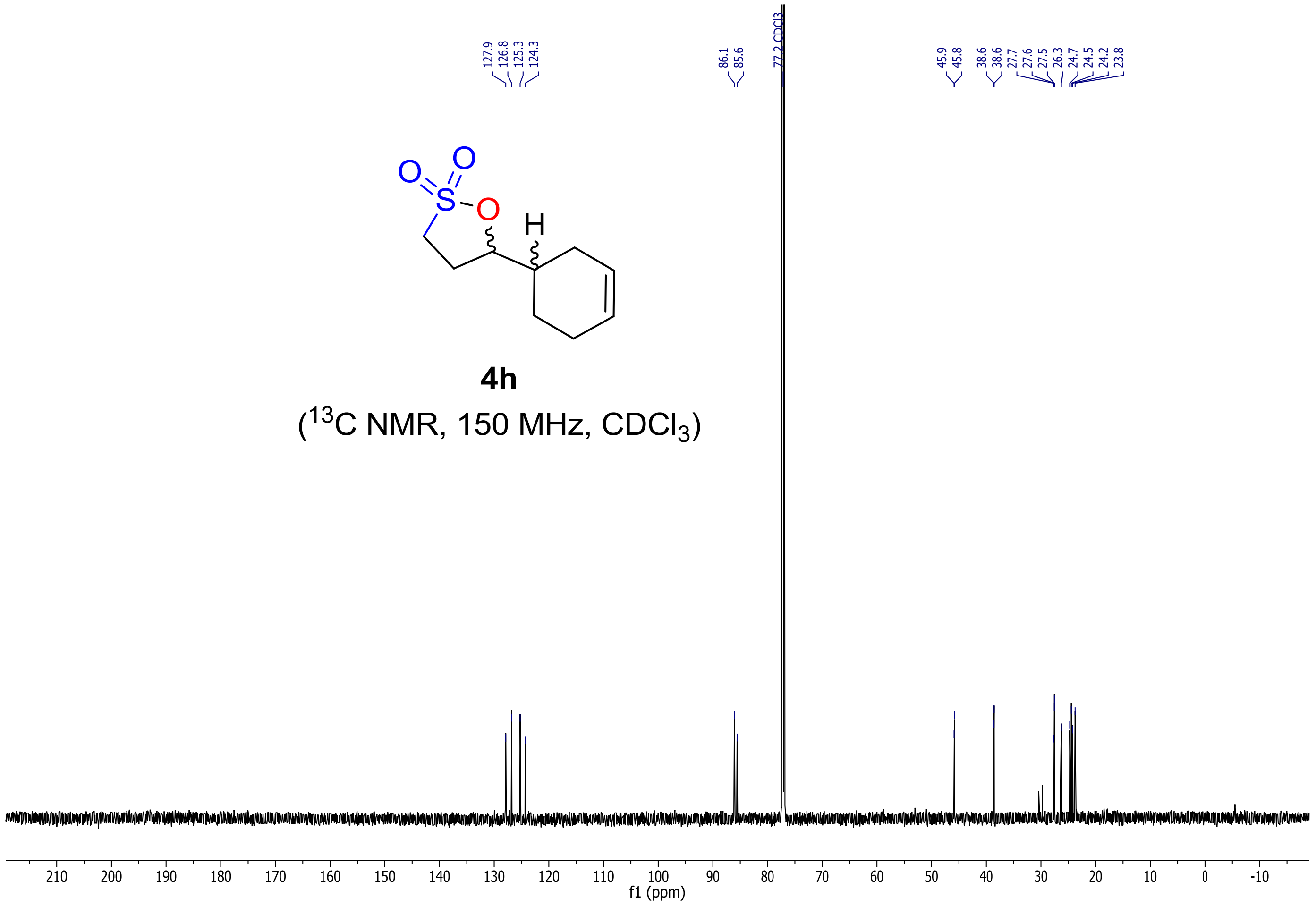
127.9
126.8
125.3
124.3
86.1
85.6
77.2 CDCl3
45.9
45.8
38.6
38.6
27.7
27.6
27.5
26.3
24.7
24.5
24.2
23.8
4h
(13C NMR, 150 MHz, CDCl3)
f1 (ppm)

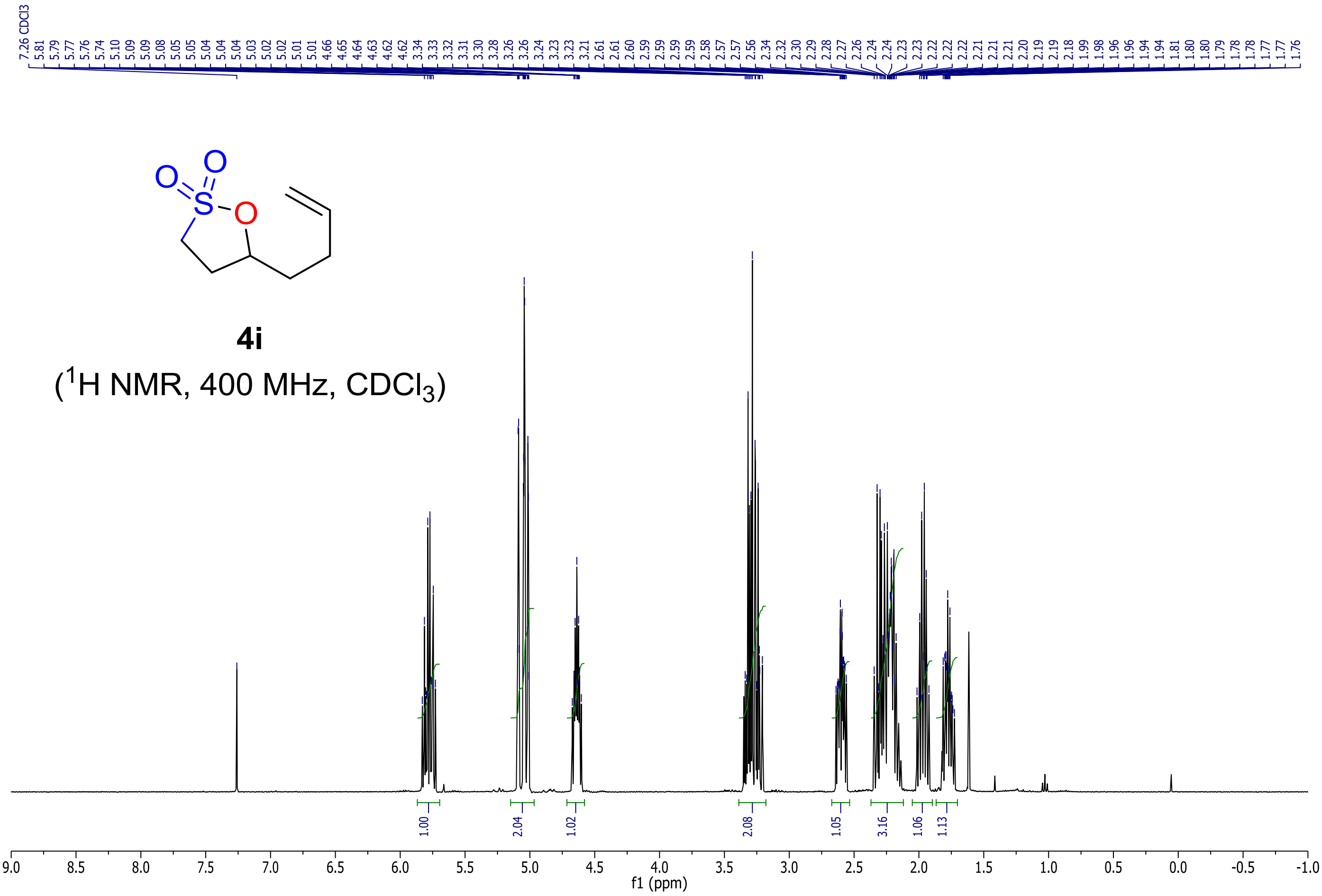
4i
(¹H NMR, 400 MHz, CDCl3)
7.26 CDCl3
1.00
2.04
1.02
2.08
1.05
3.16
1.06
1.13
f1 (ppm)

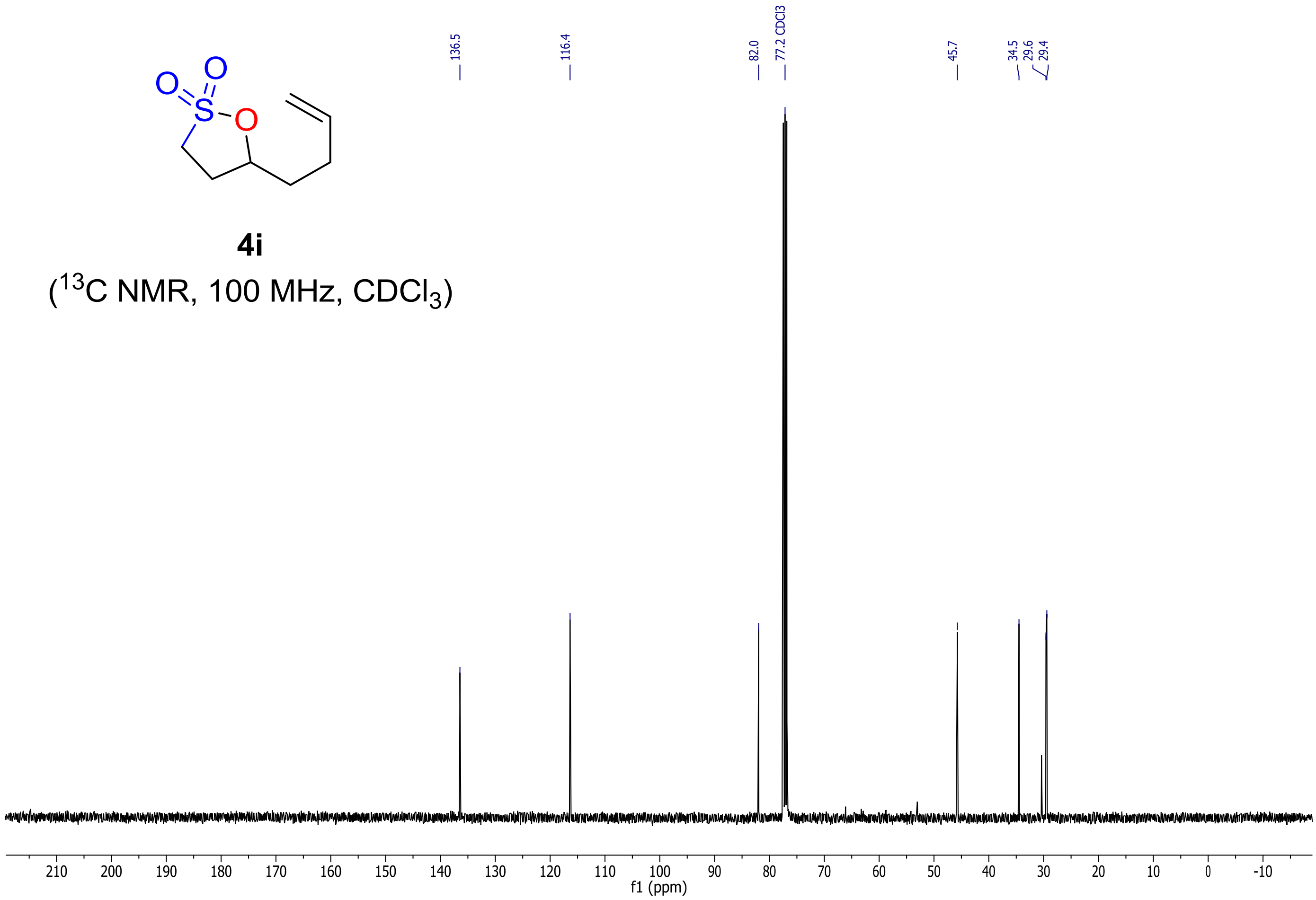
4i
(¹³C NMR, 100 MHz, CDCl3)
136.5
116.4
82.0
77.2 CDCl3
45.7
34.5
29.6
29.4
f1 (ppm)

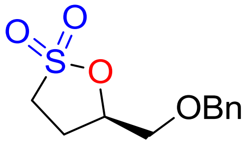

4j

(^{1}H NMR, 400 MHz, $CDCl_3$)

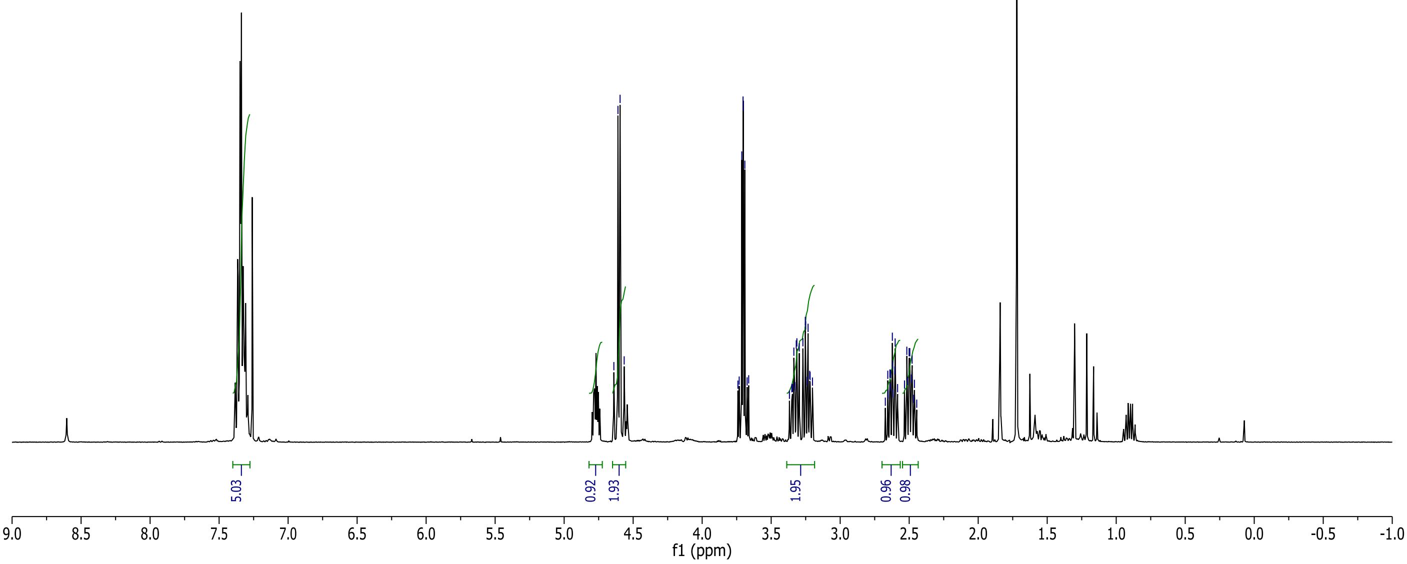

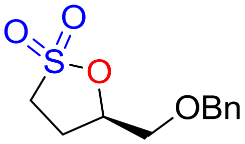

4j

(^{13}C NMR, 100 MHz, $CDCl_3$)

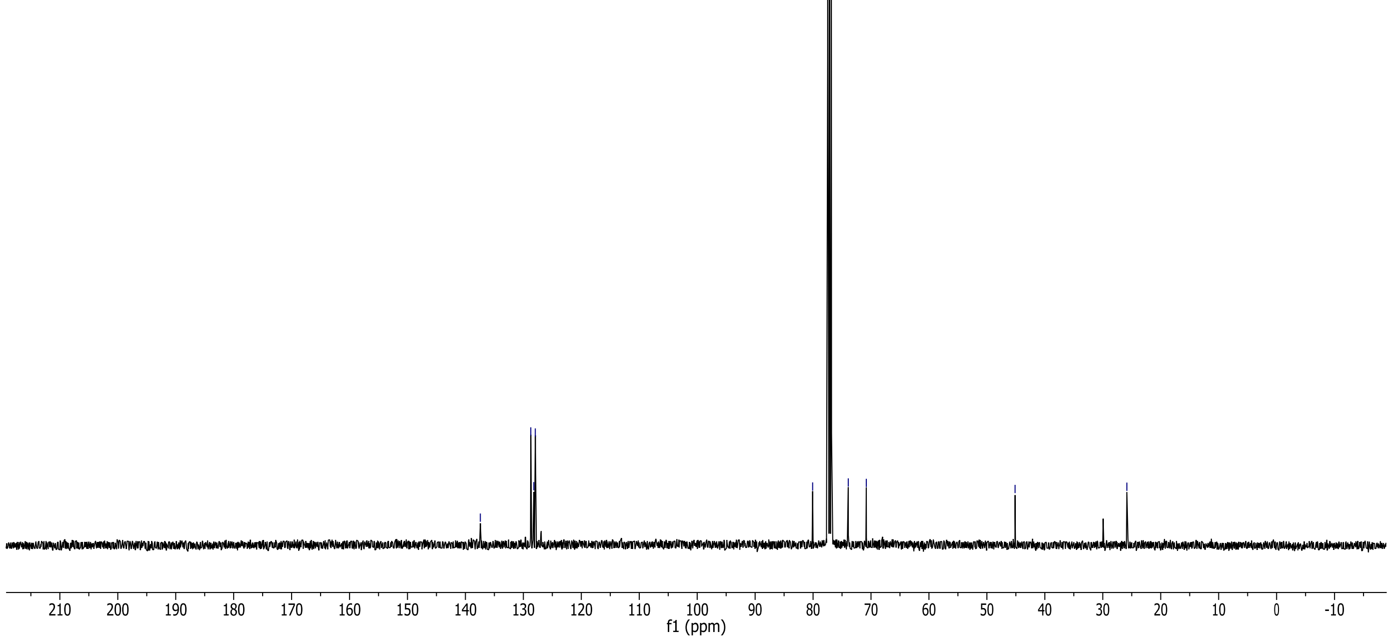

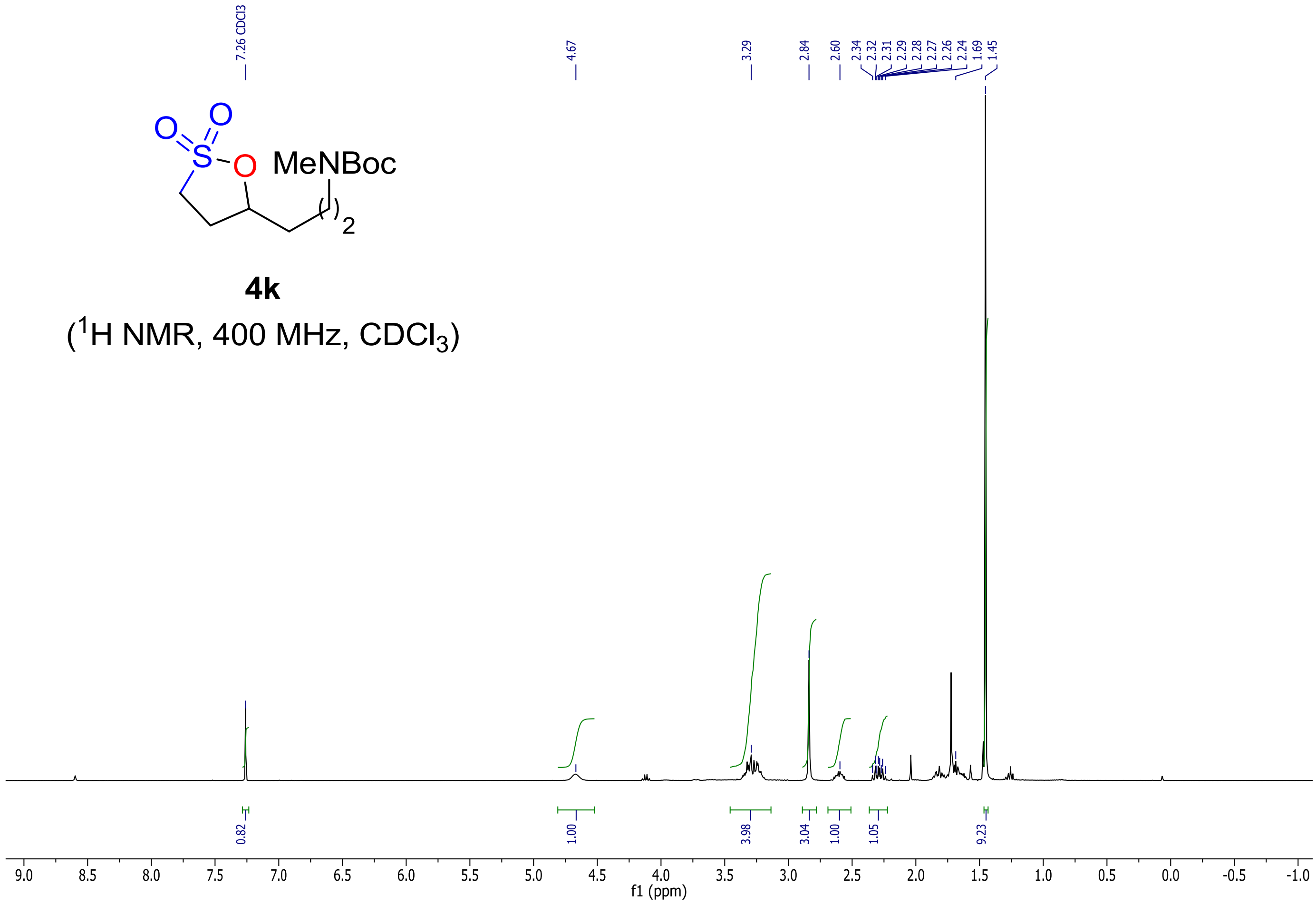
7.26 CDCl3
4.67
3.29
2.84
2.60
2.34
2.32
2.31
2.29
2.28
2.27
2.26
2.24
1.69
1.45
MeNBoc
2
4k
(^{1}H NMR, 400 MHz, $CDCl_3$)
0.82
1.00
3.98
3.04
1.00
1.05
9.23
f1 (ppm)

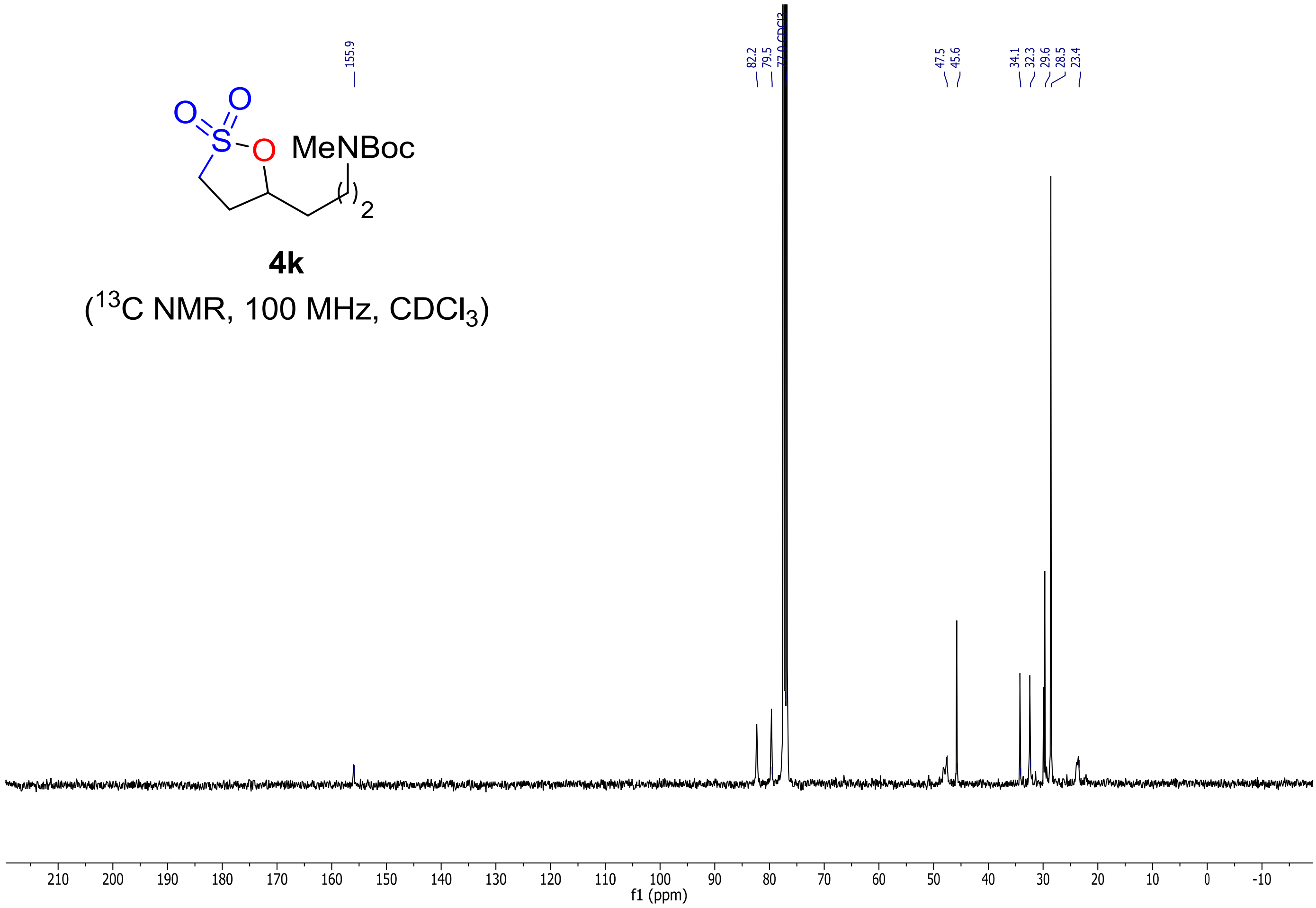
155.9
82.2
79.5
77.0 CDCl3
47.5
45.6
34.1
32.3
29.6
28.5
23.4
MeNBoc
2
4k
(^{13}C NMR, 100 MHz, $CDCl_3$)
f1 (ppm)

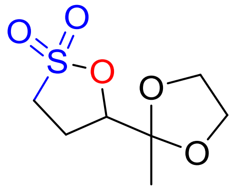

4l

(^{1}H NMR, 400 MHz, $CDCl_3$)

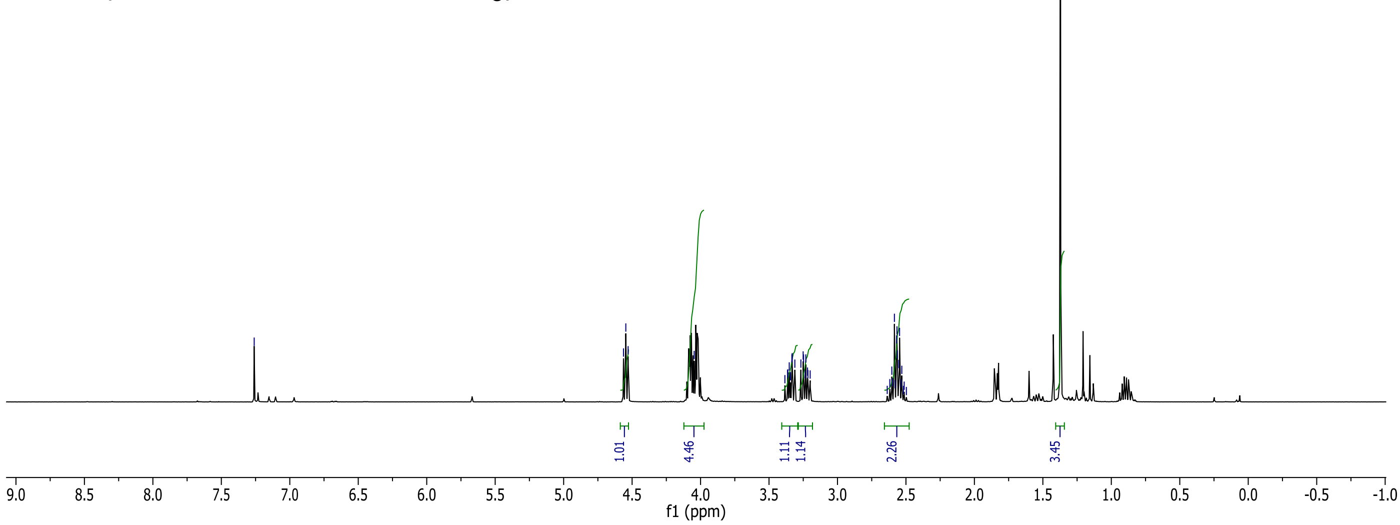

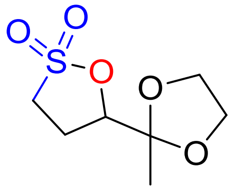

4l

(^{13}C NMR, 100 MHz, $CDCl_3$)

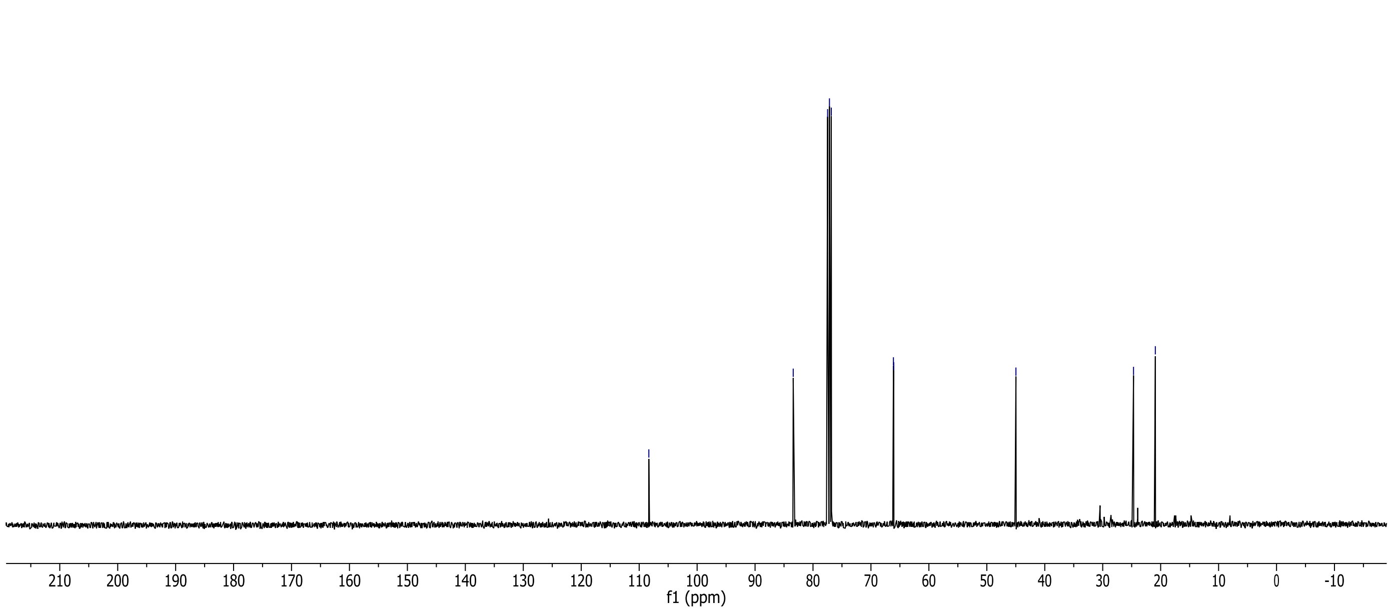

4m

(^{1}H NMR, 400 MHz, $CDCl_3$)

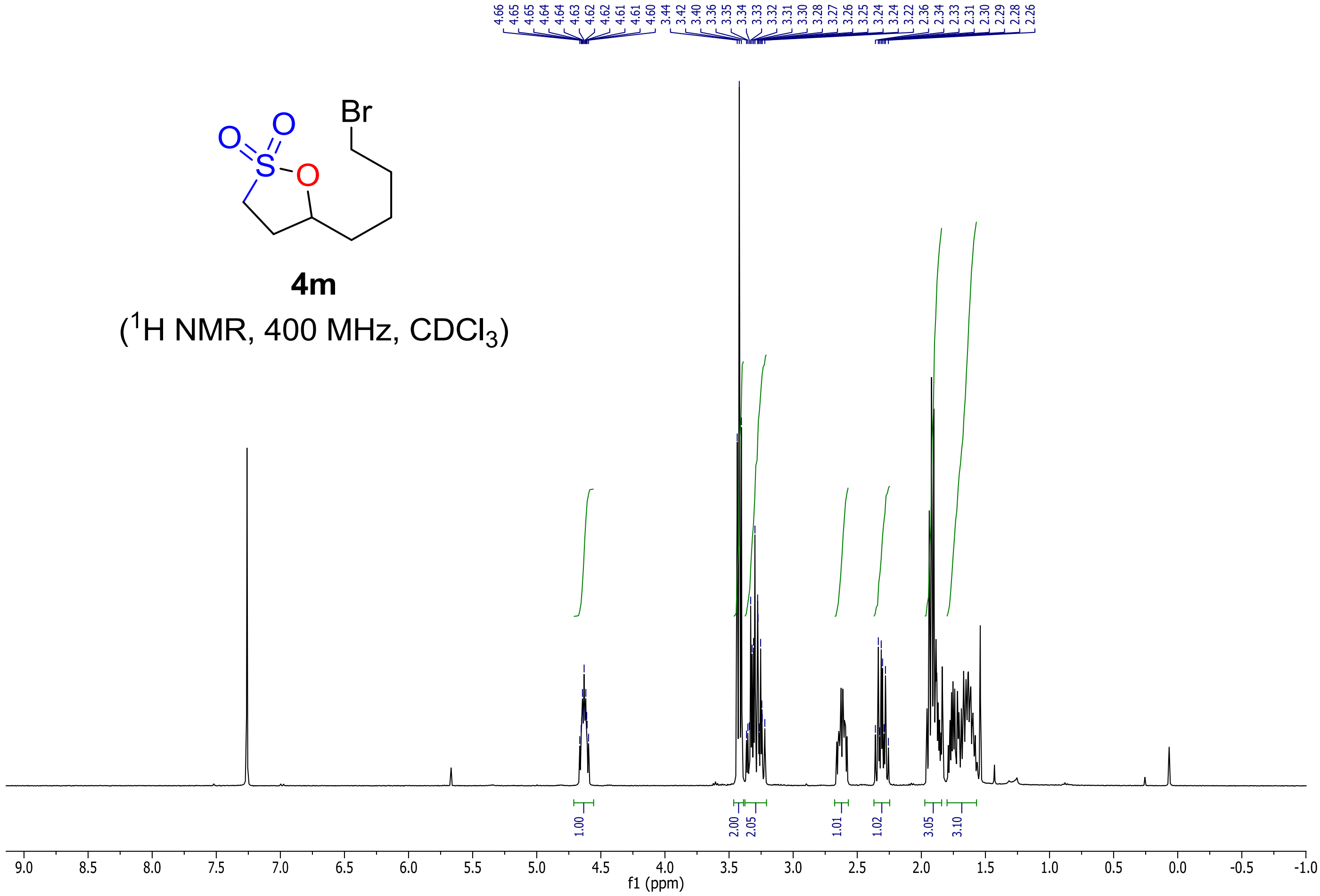

4m

(^{13}C NMR, 100 MHz, $CDCl_3$)

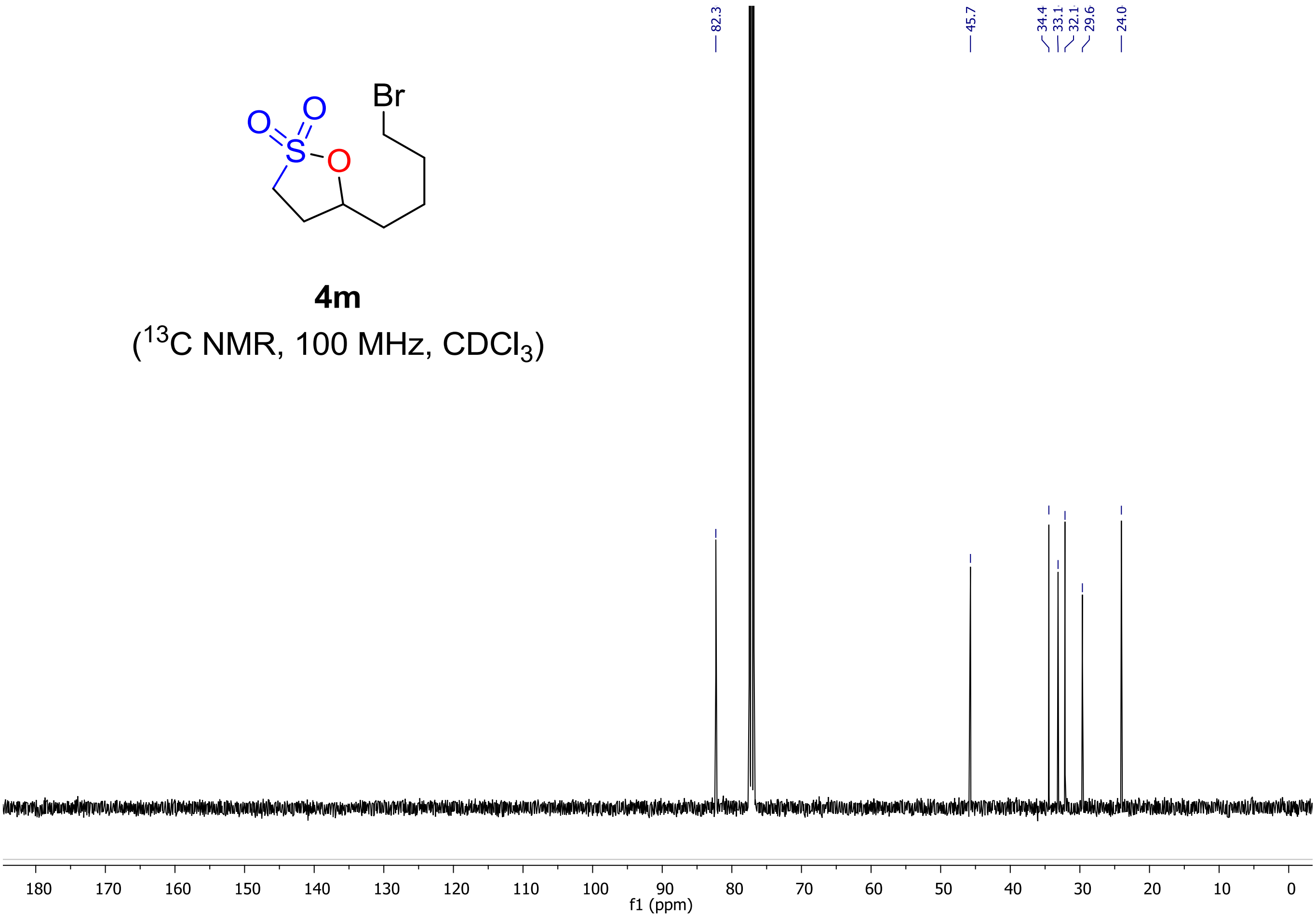

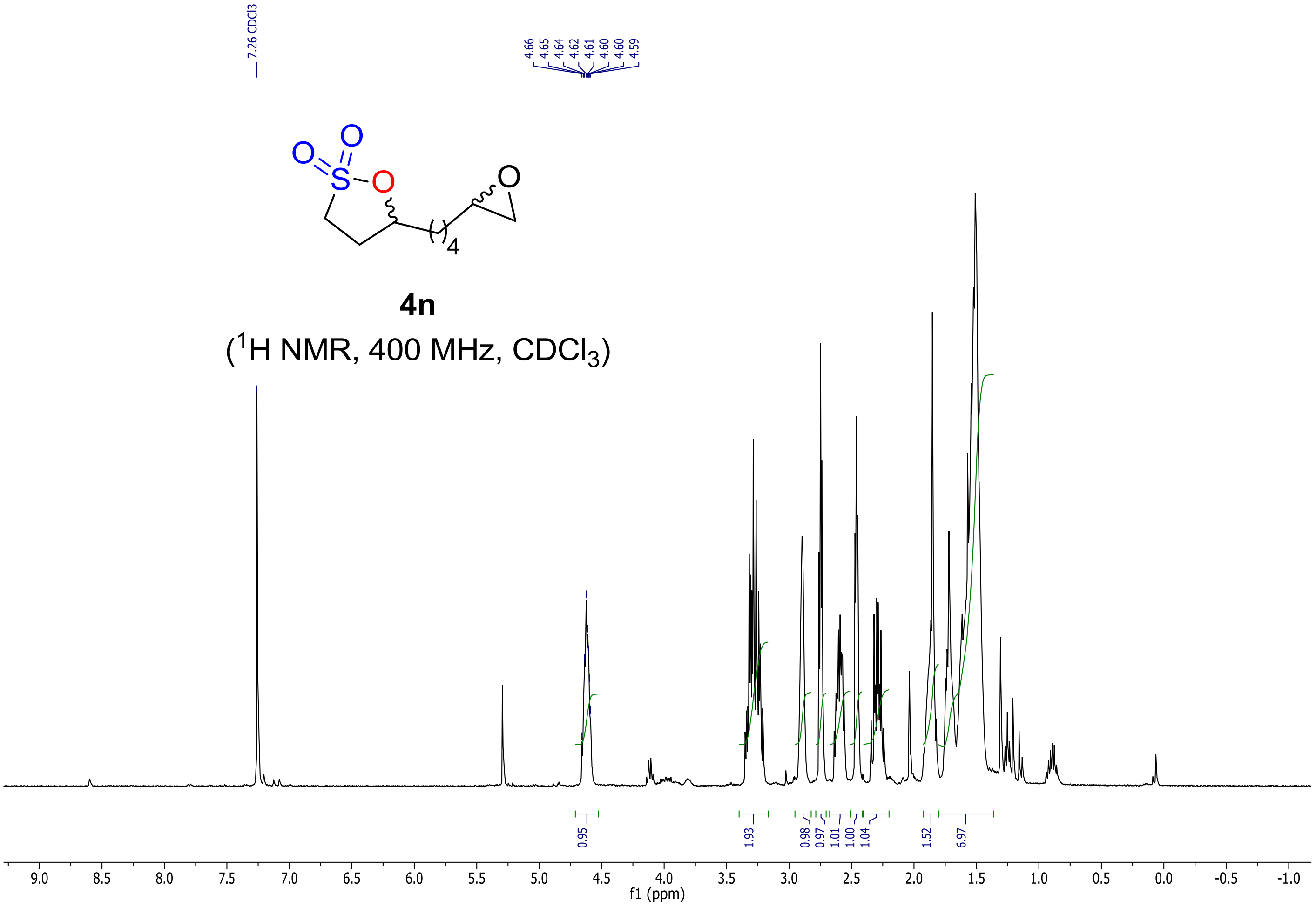
7.26 CDCl3
4.66
4.65
4.64
4.62
4.61
4.60
4.60
4.59
4n
(¹H NMR, 400 MHz, CDCl₃)
0.95
1.93
0.98
0.97
1.01
1.00
1.04
1.52
6.97
9.0 8.5 8.0 7.5 7.0 6.5 6.0 5.5 5.0 4.5 4.0 3.5 3.0 2.5 2.0 1.5 1.0 0.5 0.0 -0.5 -1.0
f1 (ppm)

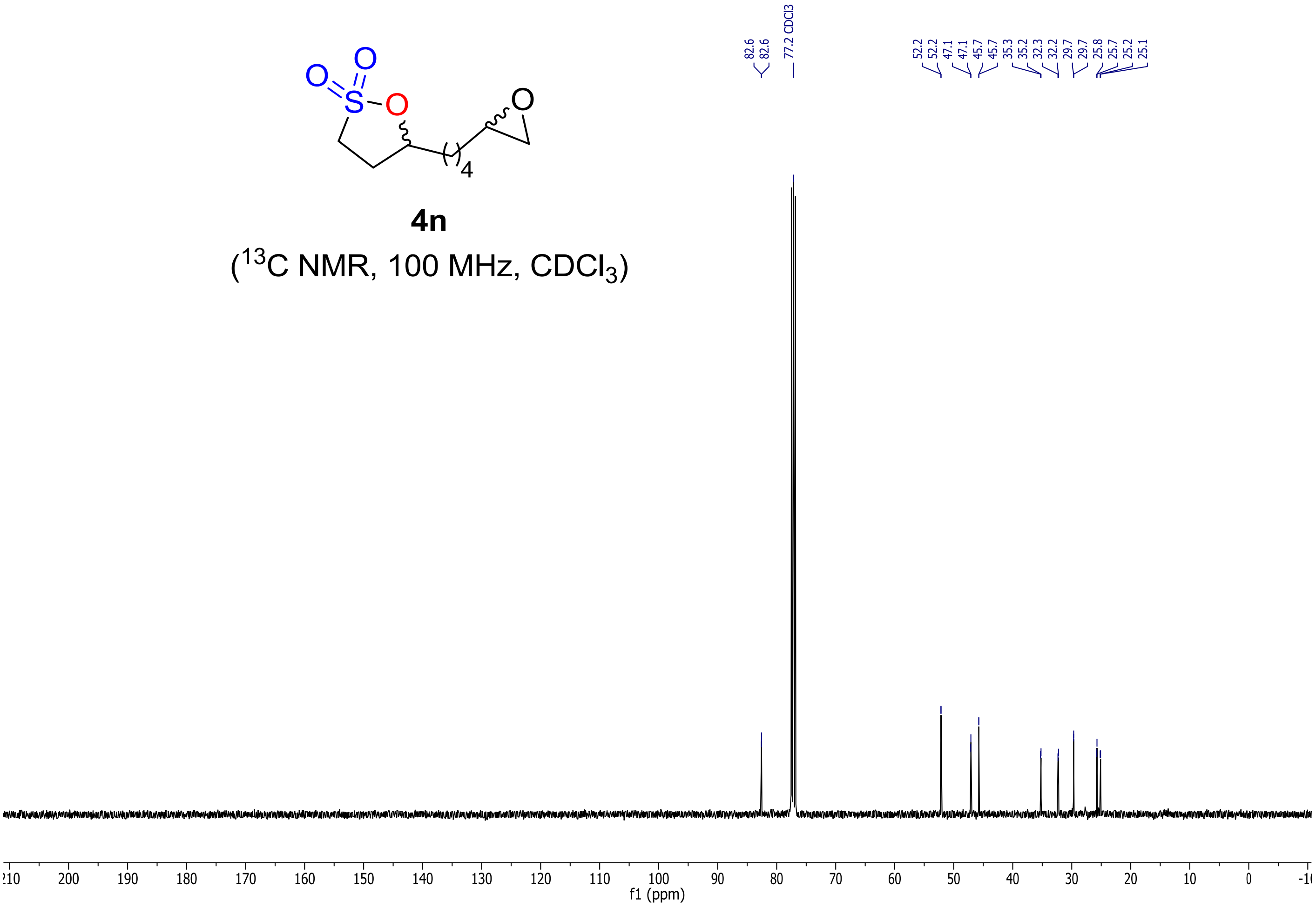
82.6
82.6
77.2 CDCl3
52.2
52.2
47.1
47.1
45.7
45.7
35.3
35.2
32.3
32.2
29.7
29.7
25.8
25.7
25.2
25.1
4n
(¹³C NMR, 100 MHz, CDCl₃)
200 190 180 170 160 150 140 130 120 110 100 90 80 70 60 50 40 30 20 10 0
f1 (ppm)

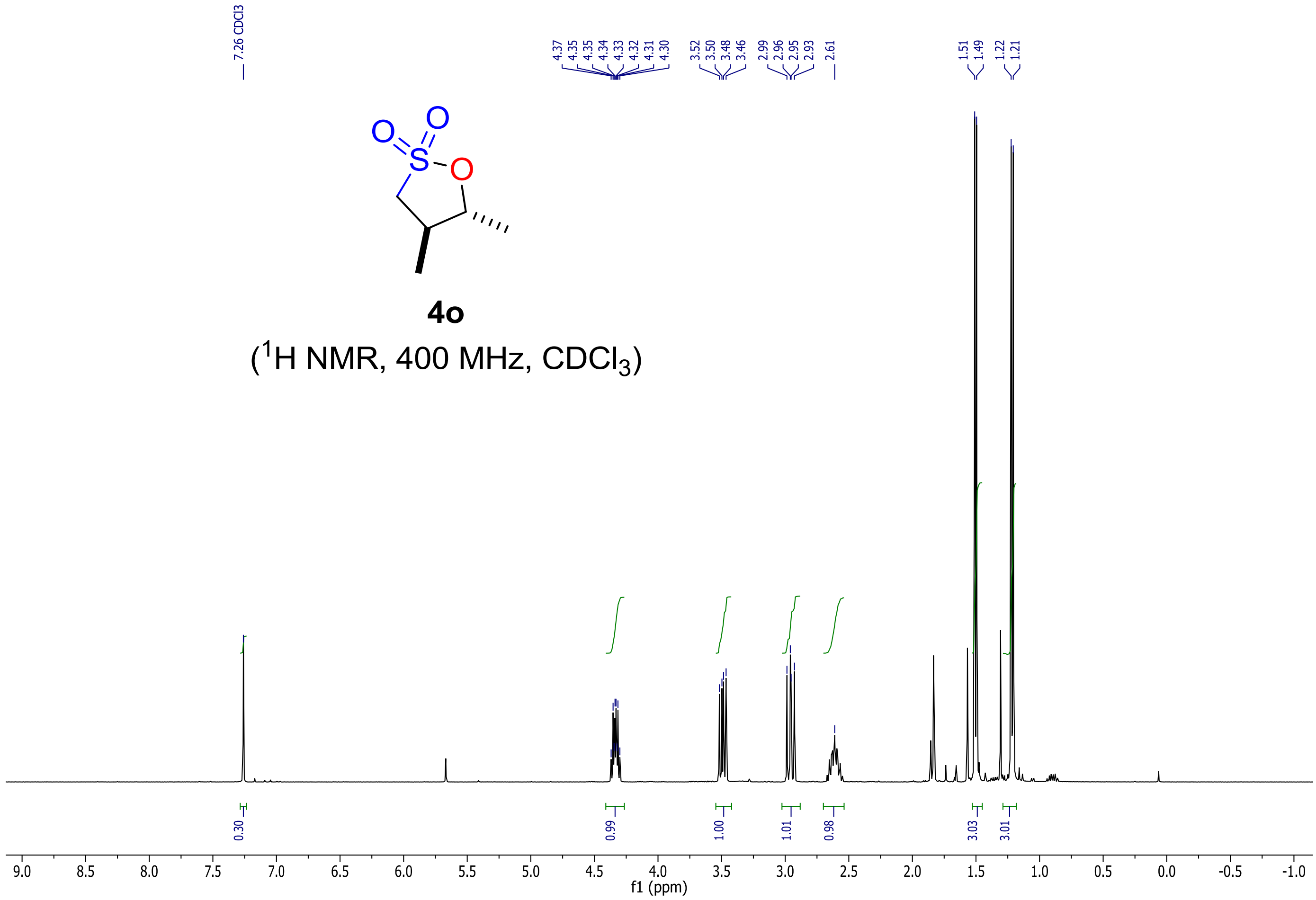

4o

(^{1}H NMR, 400 MHz, $CDCl_3$)

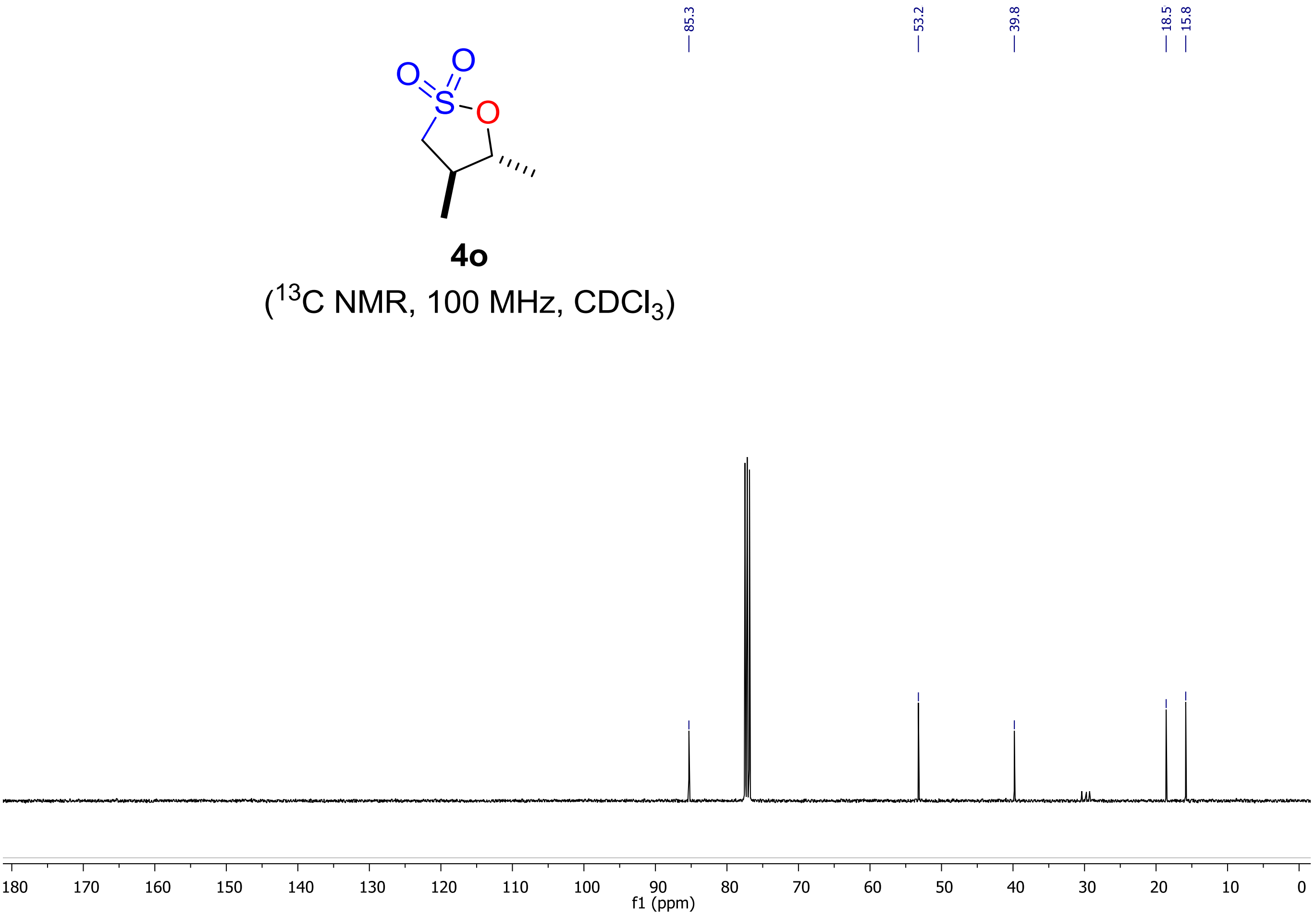

4o

(^{13}C NMR, 100 MHz, $CDCl_3$)

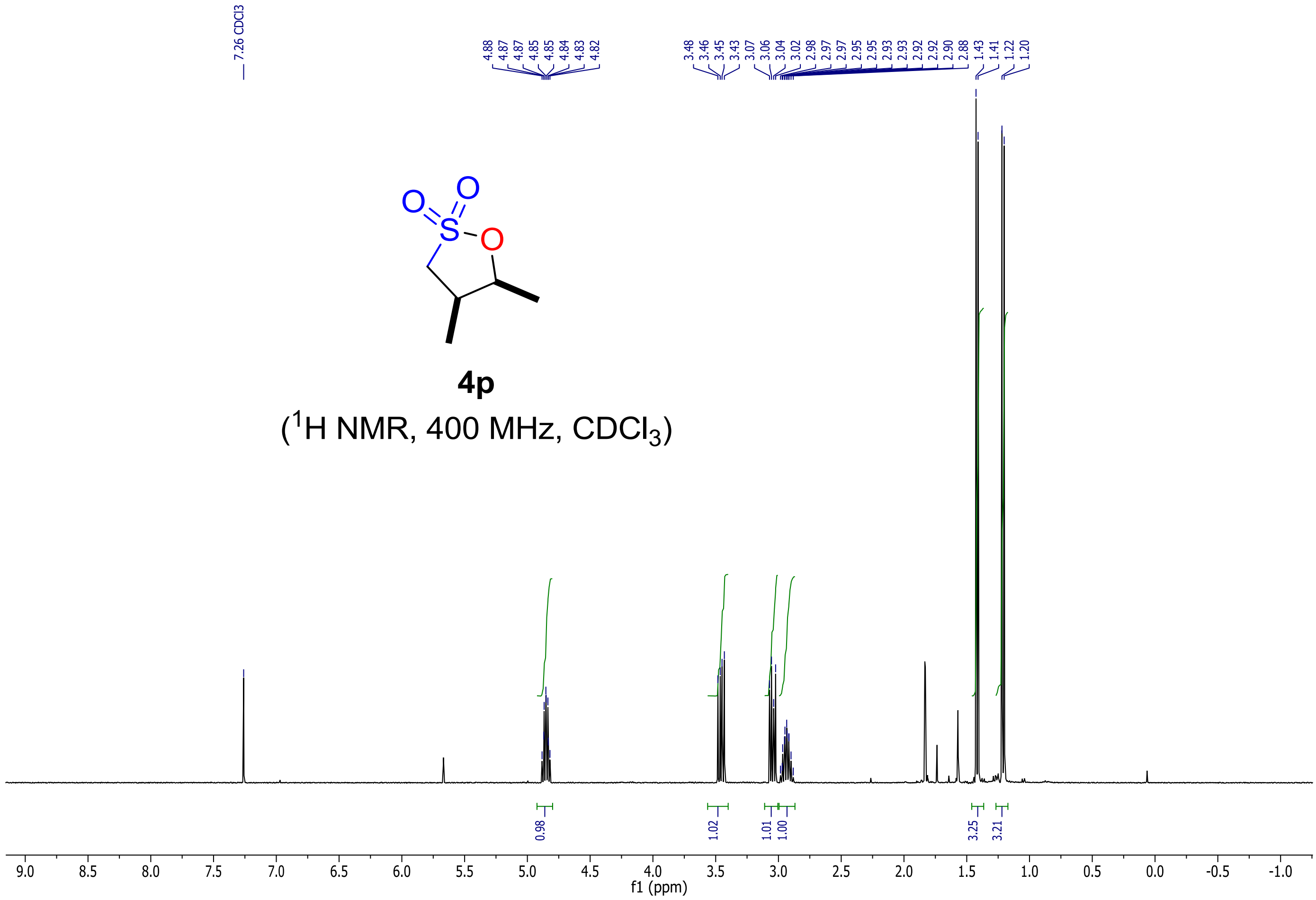

4p

(^{1}H NMR, 400 MHz, $CDCl_3$)

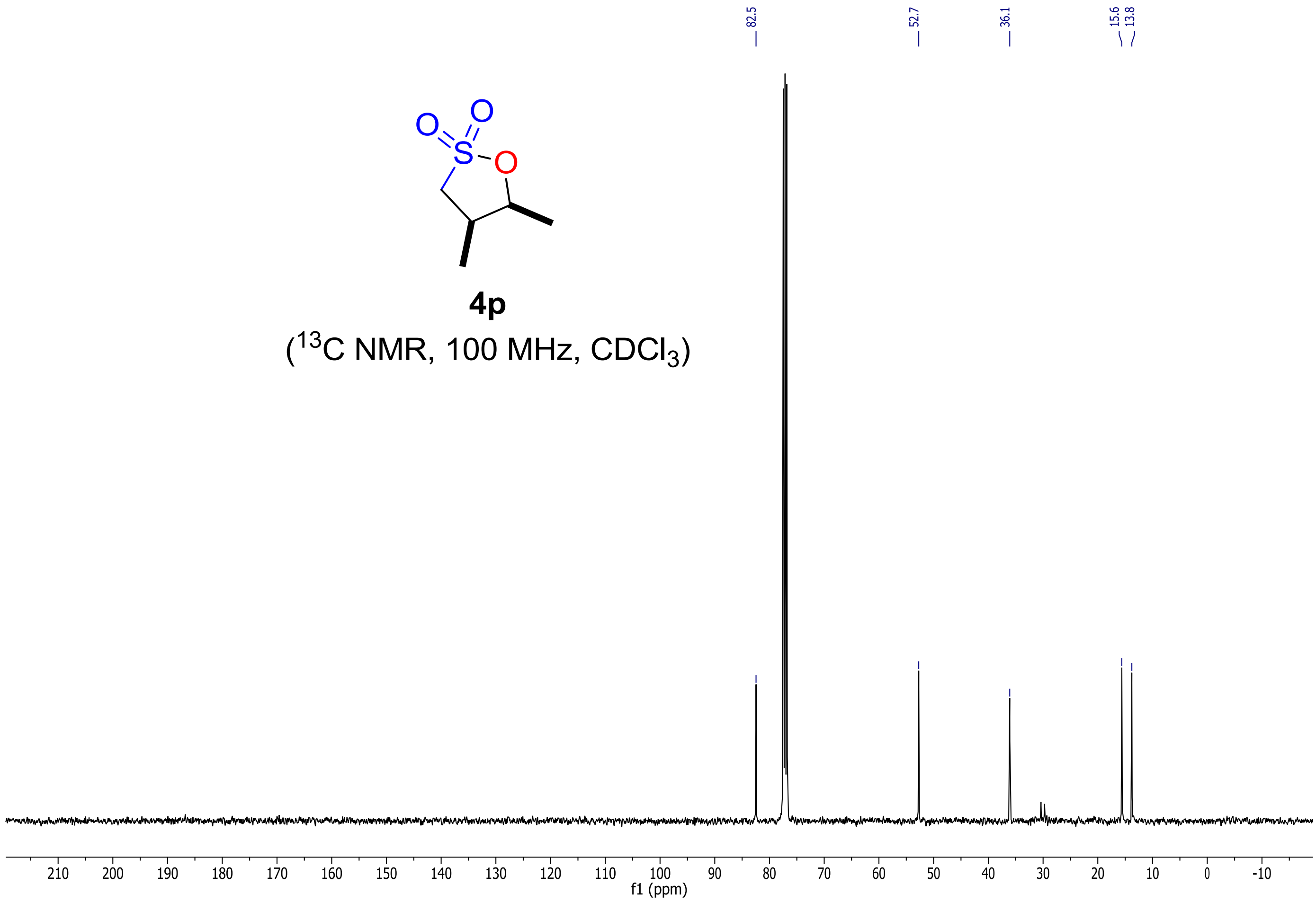

4p

(^{13}C NMR, 100 MHz, $CDCl_3$)

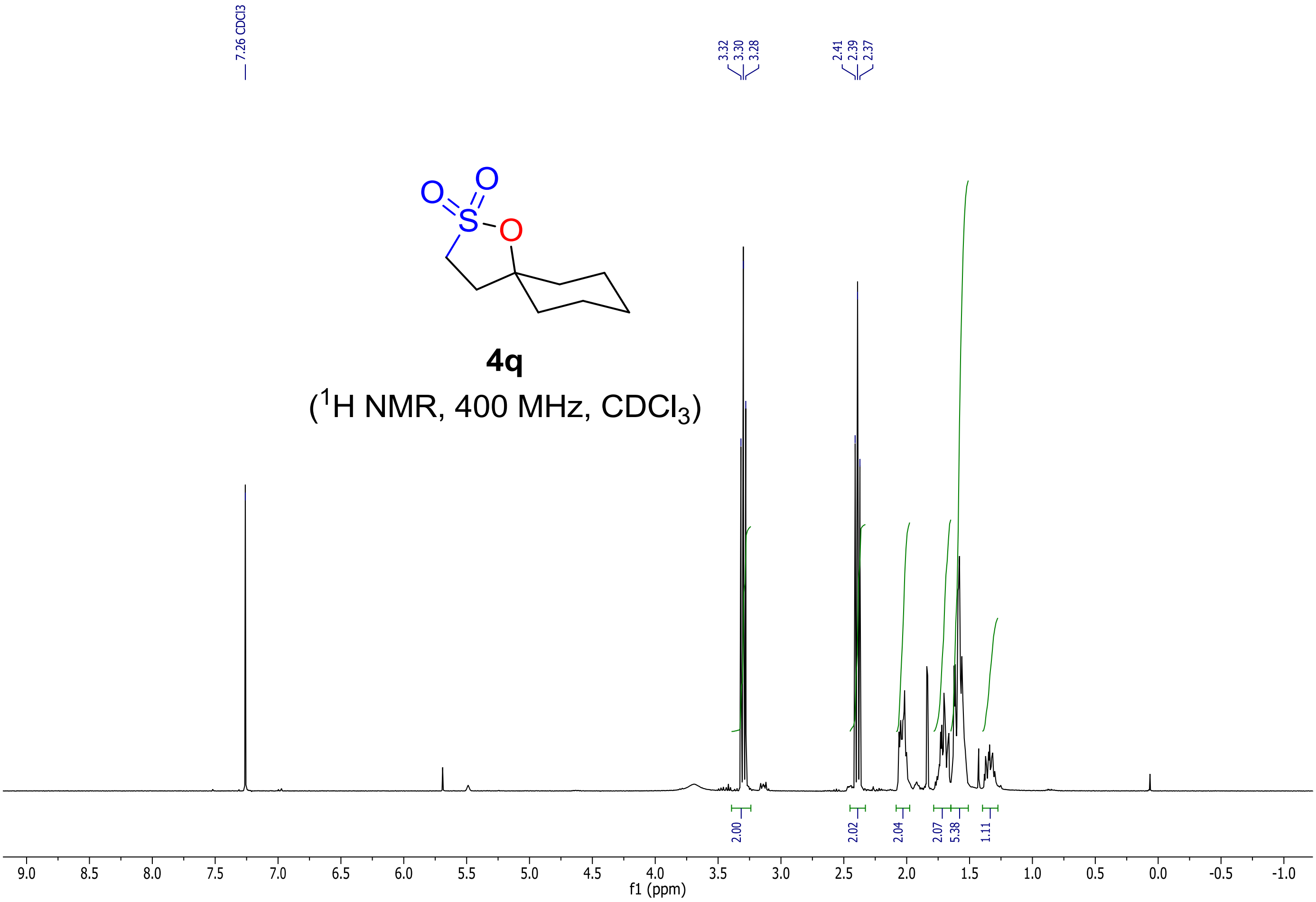

4q
(^{1}H NMR, 400 MHz, $CDCl_3$)

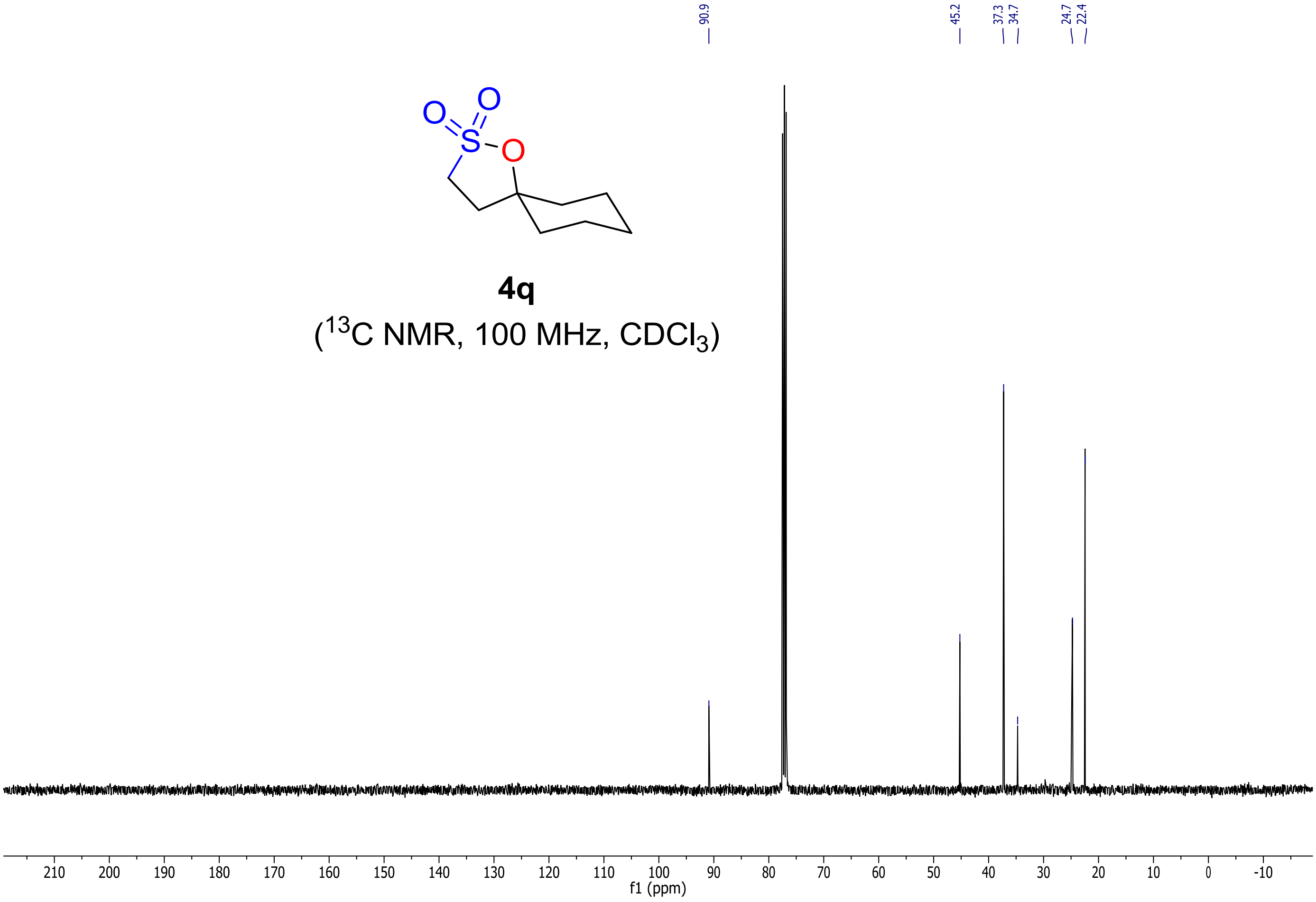

4q
(^{13}C NMR, 100 MHz, $CDCl_3$)

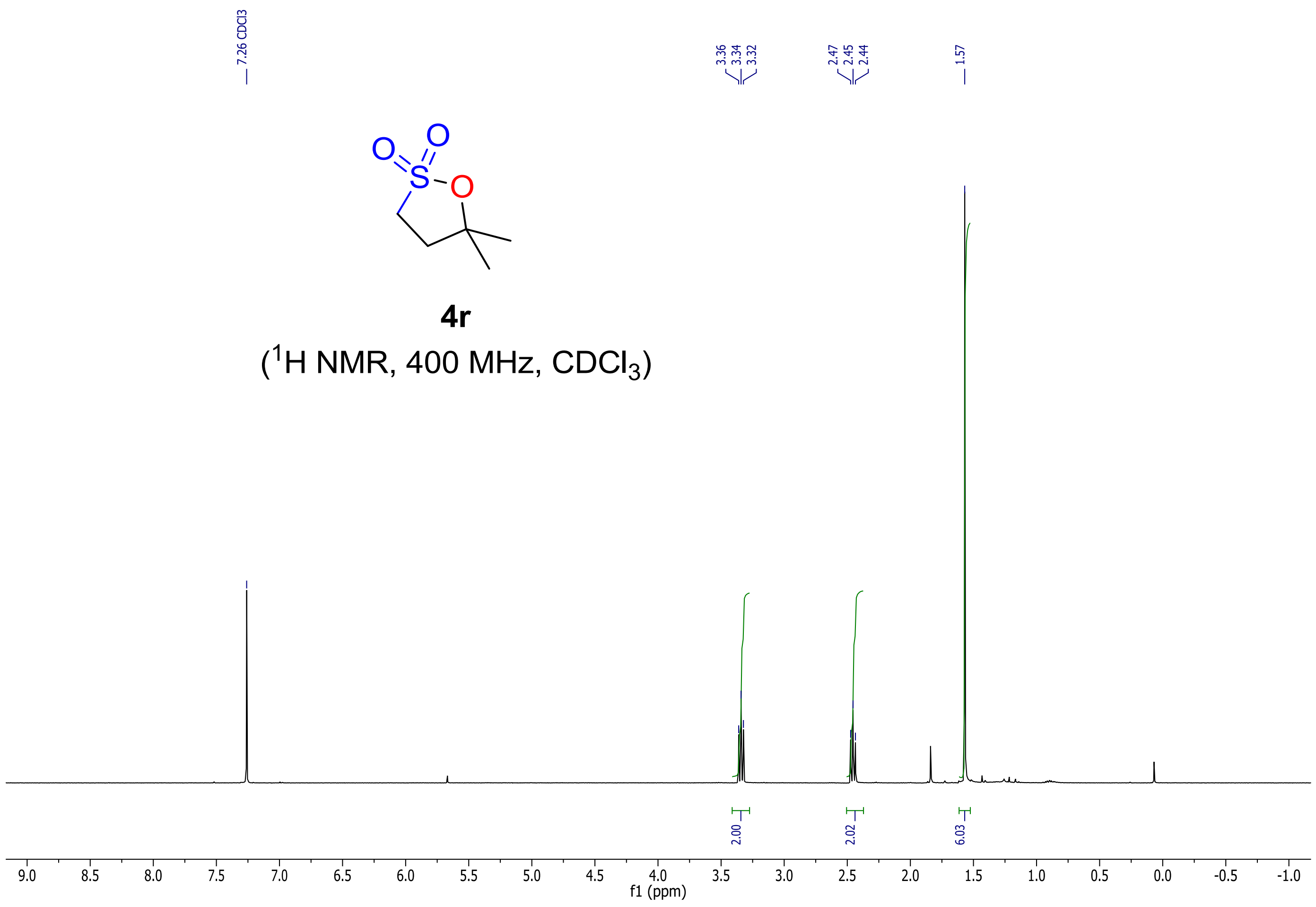
4r
(¹H NMR, 400 MHz, CDCl₃)
7.26 CDCl3
3.36
3.34
3.32
2.47
2.45
2.44
1.57
2.00
2.02
6.03
f1 (ppm)

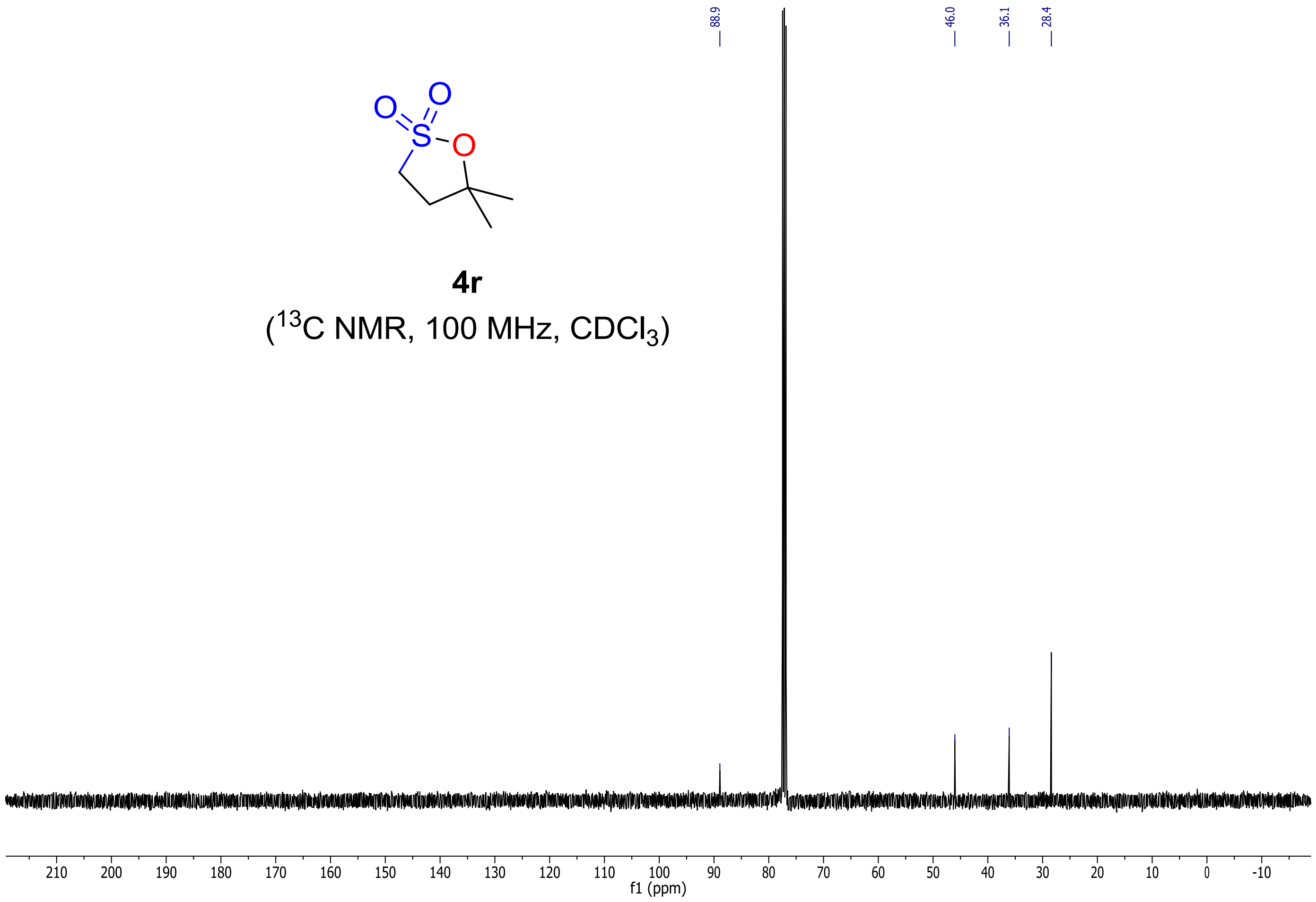
4r
(¹³C NMR, 100 MHz, CDCl₃)
88.9
46.0
36.1
28.4
f1 (ppm)

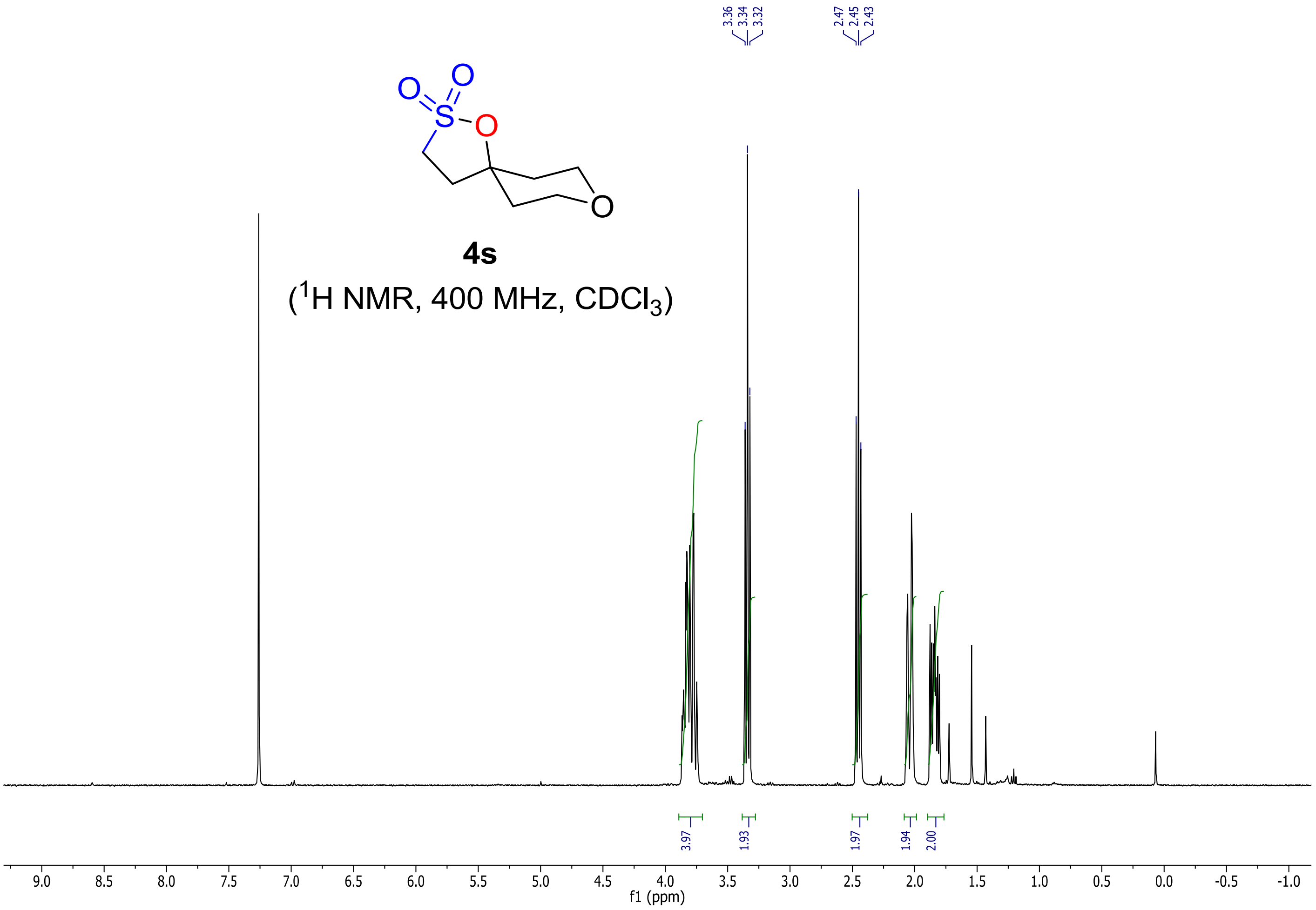

3.36
3.34
3.32
2.47
2.45
2.43
4s
(1H NMR, 400 MHz, CDCl3)
3.97
1.93
1.97
1.94
2.00
f1 (ppm)

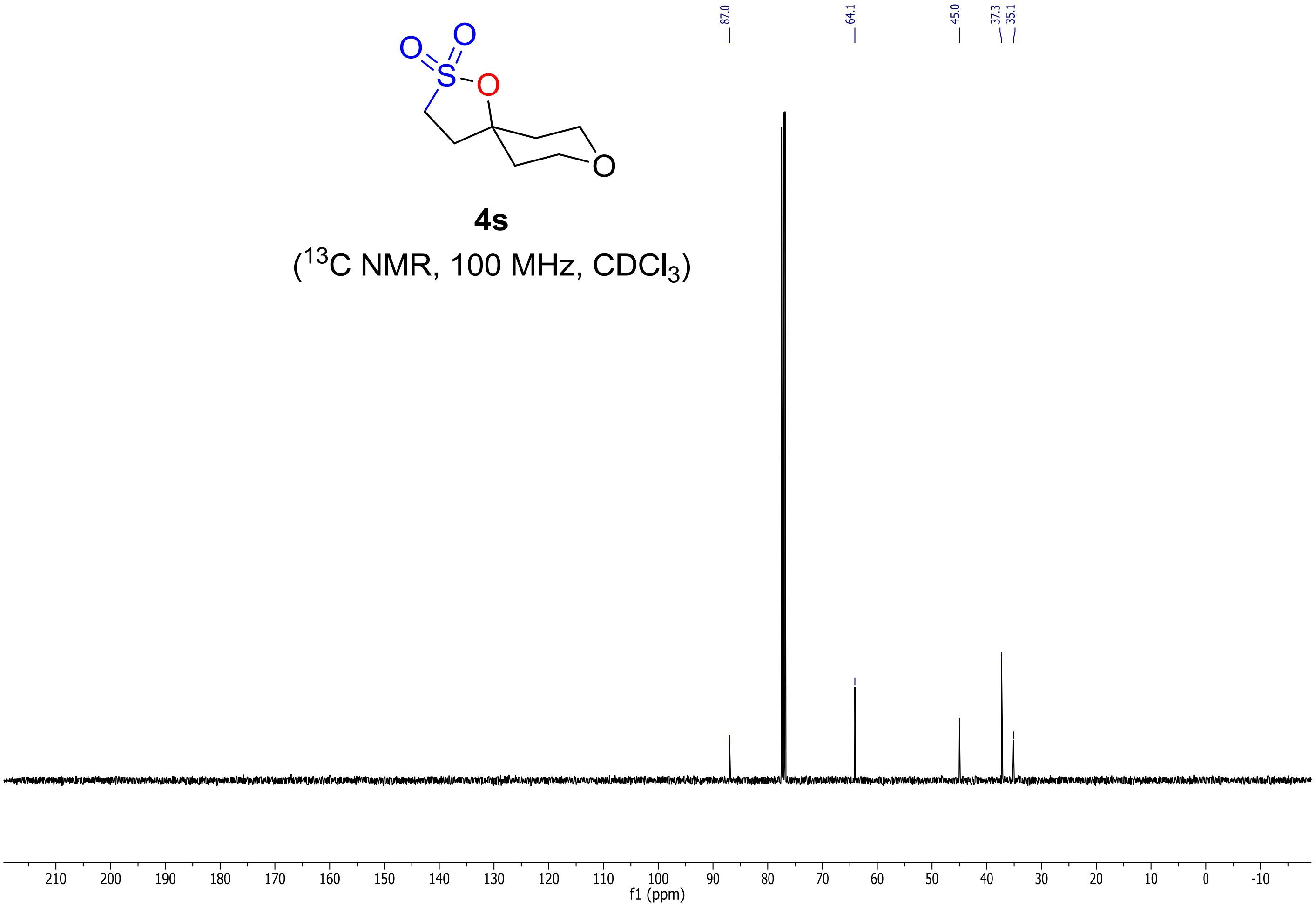

87.0
64.1
45.0
37.3
35.1
4s
(13C NMR, 100 MHz, CDCl3)
f1 (ppm)

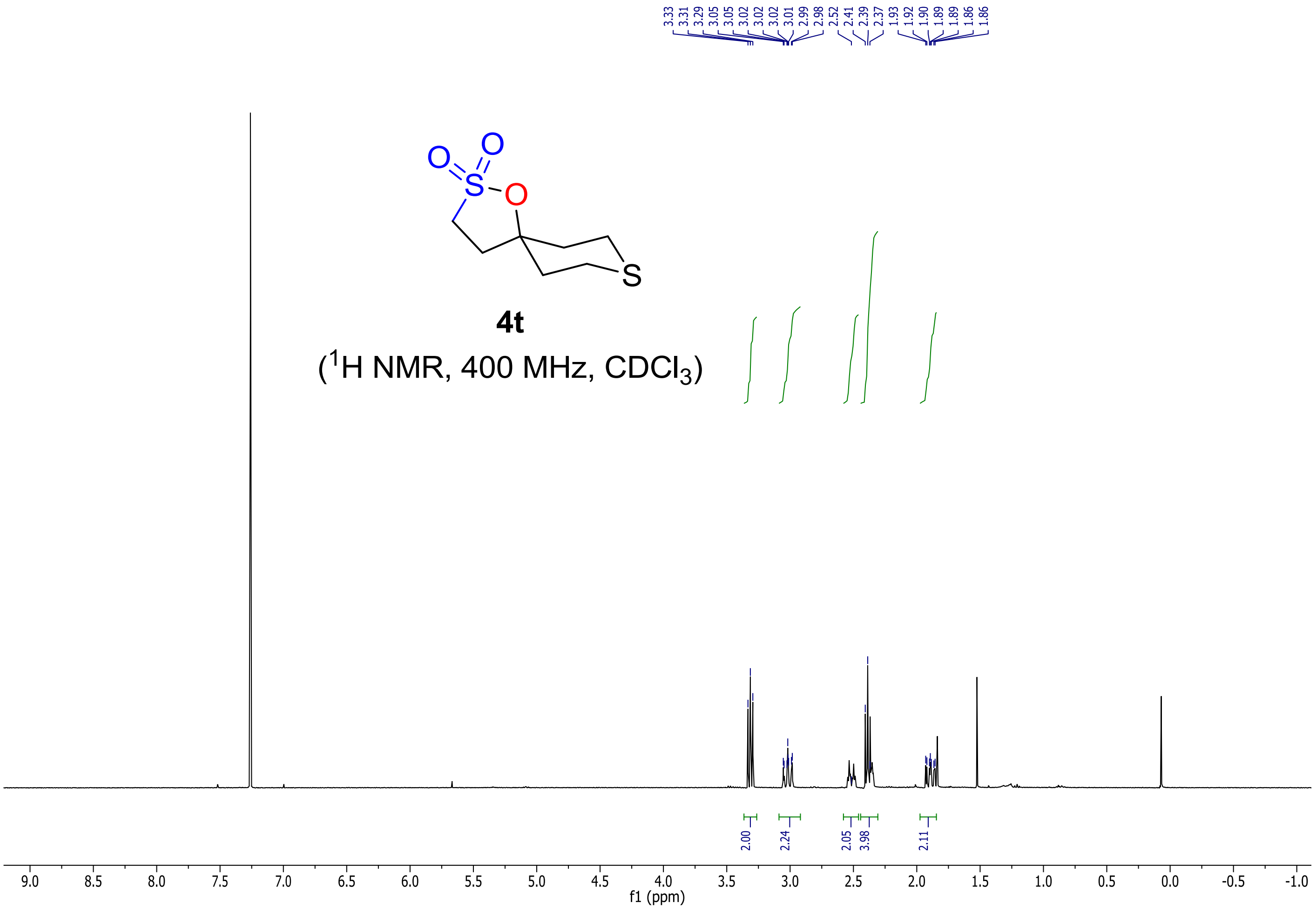
3.33
3.31
3.29
3.05
3.05
3.02
3.02
3.02
3.01
2.99
2.98
2.52
2.41
2.39
2.37
1.93
1.92
1.90
1.89
1.89
1.86
1.86
O
O
S
O
S
4t
(1H NMR, 400 MHz, CDCl3)
2.00
2.24
2.05
3.98
2.11
9.0 8.5 8.0 7.5 7.0 6.5 6.0 5.5 5.0 4.5 4.0 3.5 3.0 2.5 2.0 1.5 1.0 0.5 0.0 -0.5 -1.0
f1 (ppm)

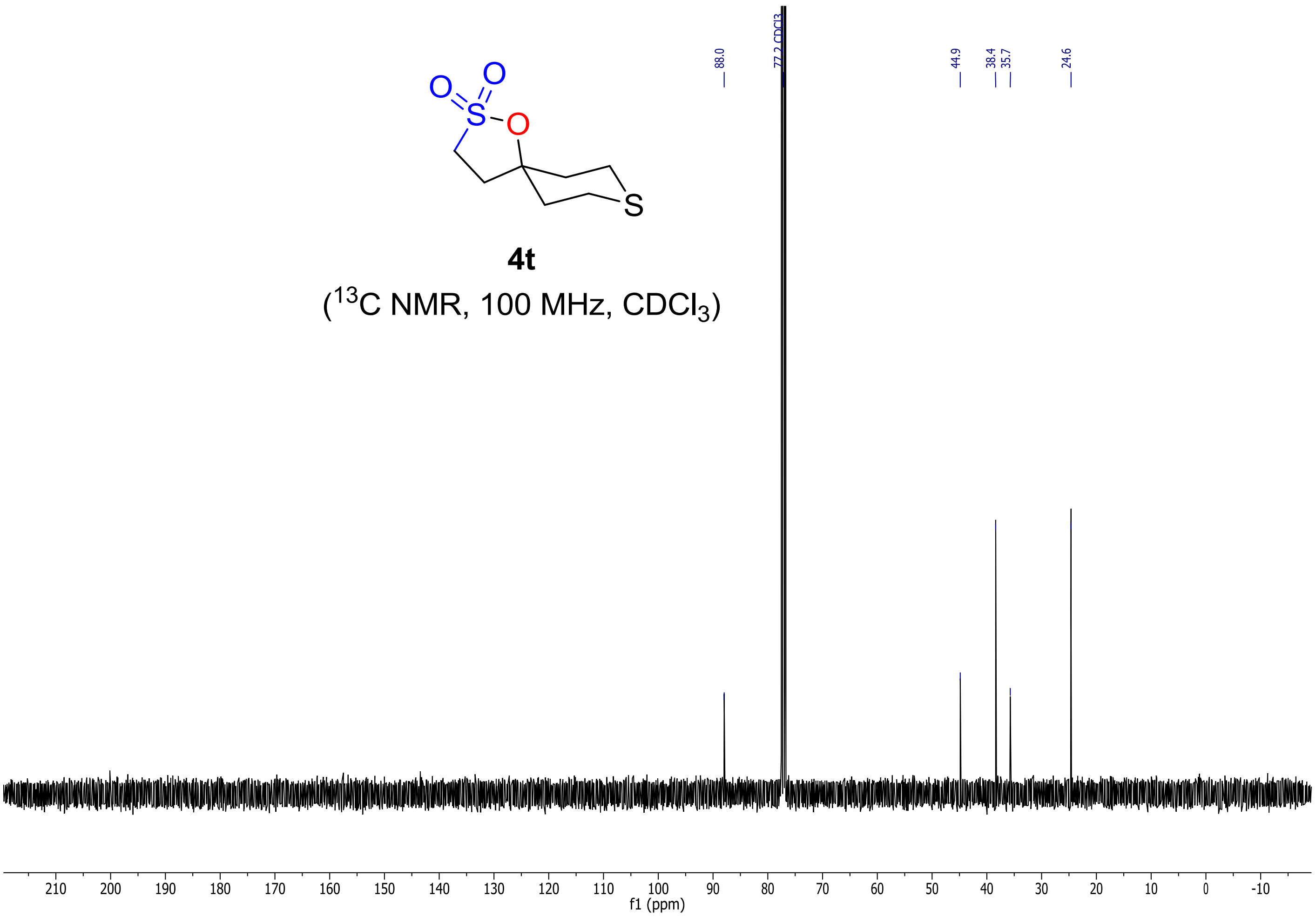
88.0
77.2 CDCl3
44.9
38.4
35.7
24.6
O
O
S
O
S
4t
(13C NMR, 100 MHz, CDCl3)
210 200 190 180 170 160 150 140 130 120 110 100 90 80 70 60 50 40 30 20 10 0 -10
f1 (ppm)

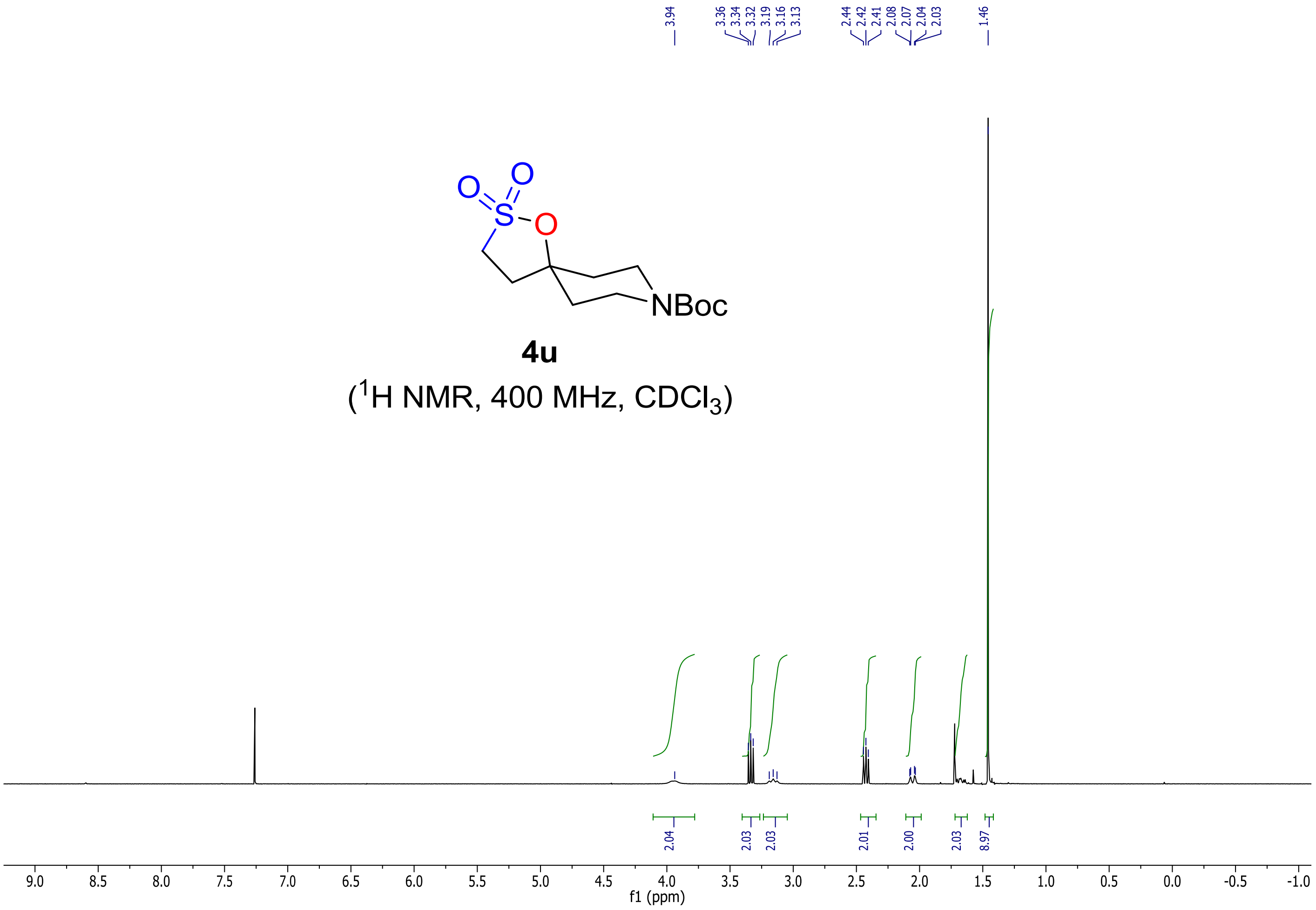
3.94
3.36
3.34
3.32
3.19
3.16
3.13
2.44
2.42
2.41
2.08
2.07
2.04
2.03
1.46
NBoc
4u
(1H NMR, 400 MHz, CDCl3)
2.04
2.03
2.03
2.01
2.00
2.03
8.97
f1 (ppm)

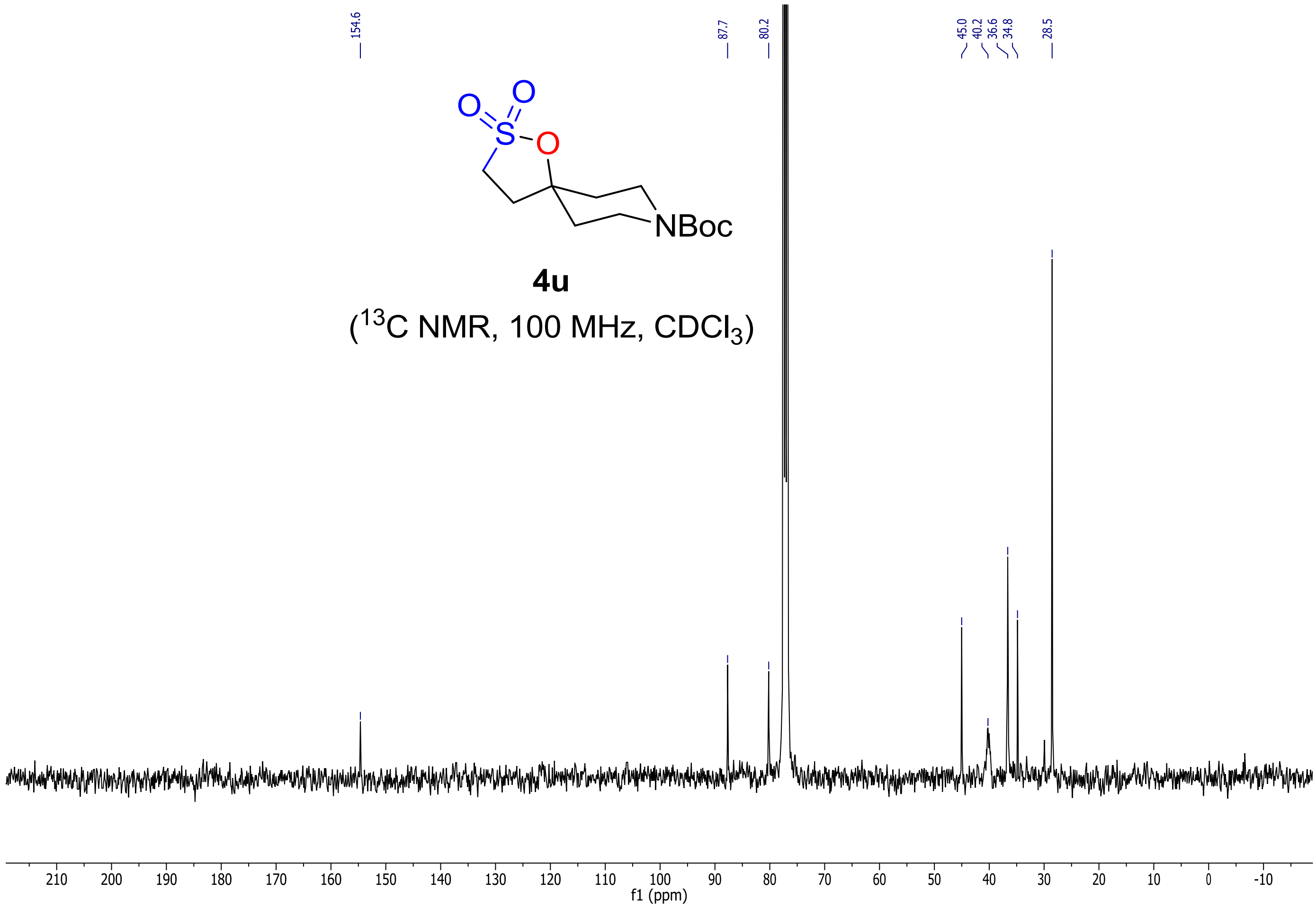
154.6
87.7
80.2
45.0
40.2
36.6
34.8
28.5
NBoc
4u
(13C NMR, 100 MHz, CDCl3)
f1 (ppm)

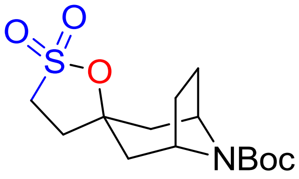

4v

(^{1}H NMR, 400 MHz, $CDCl_3$)

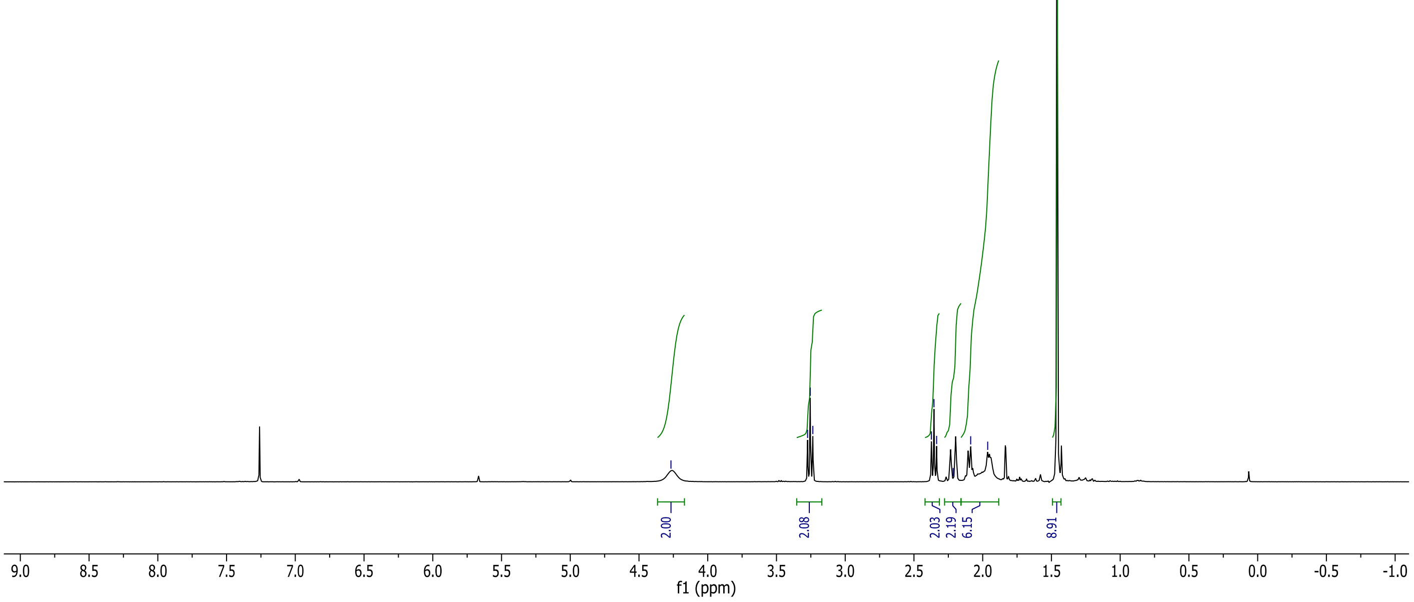

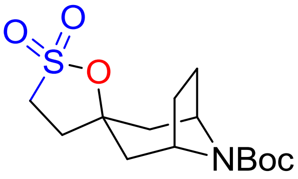

4v

(^{13}C NMR, 100 MHz, $CDCl_3$)

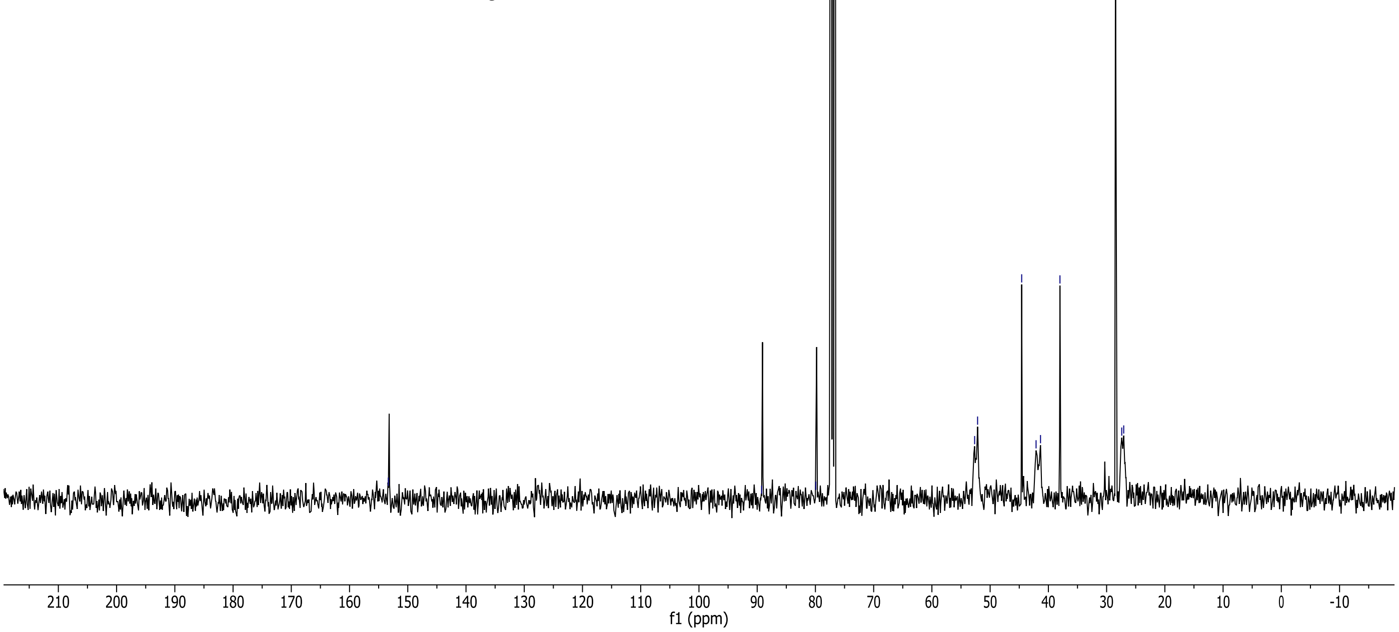

9. ^{1}H and ^{13}C NMR data for γ-hydroxysulfones 5a-j

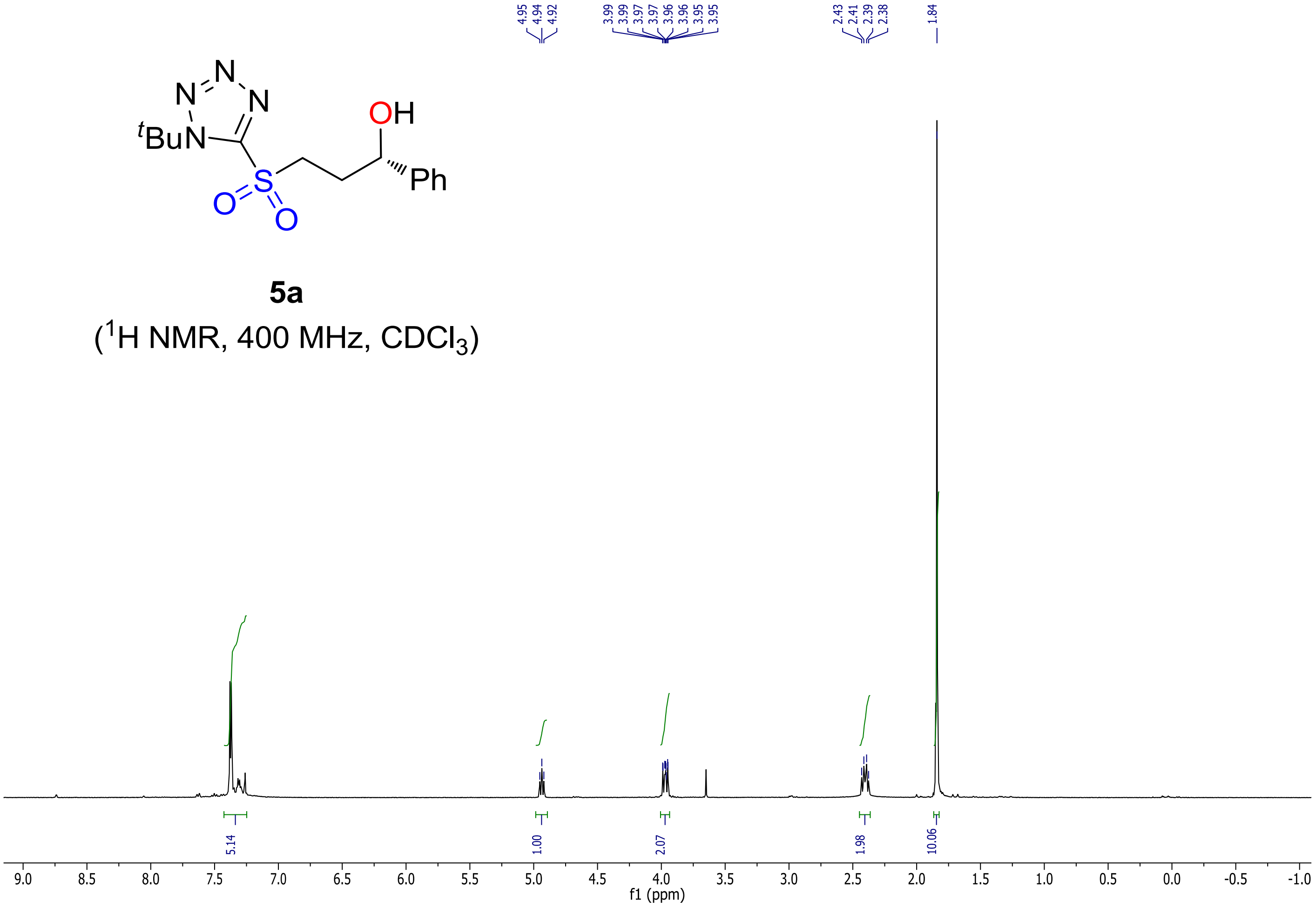

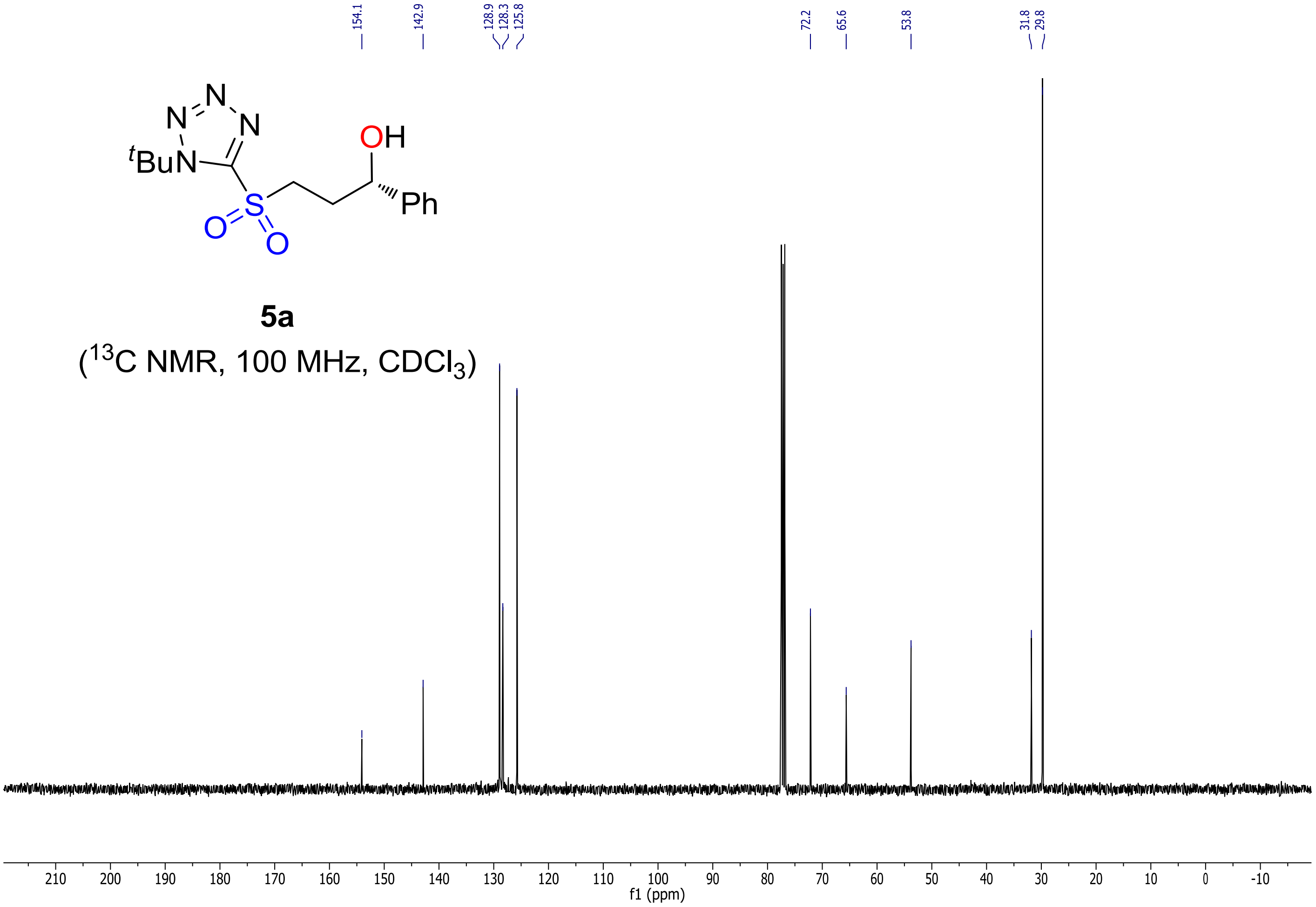

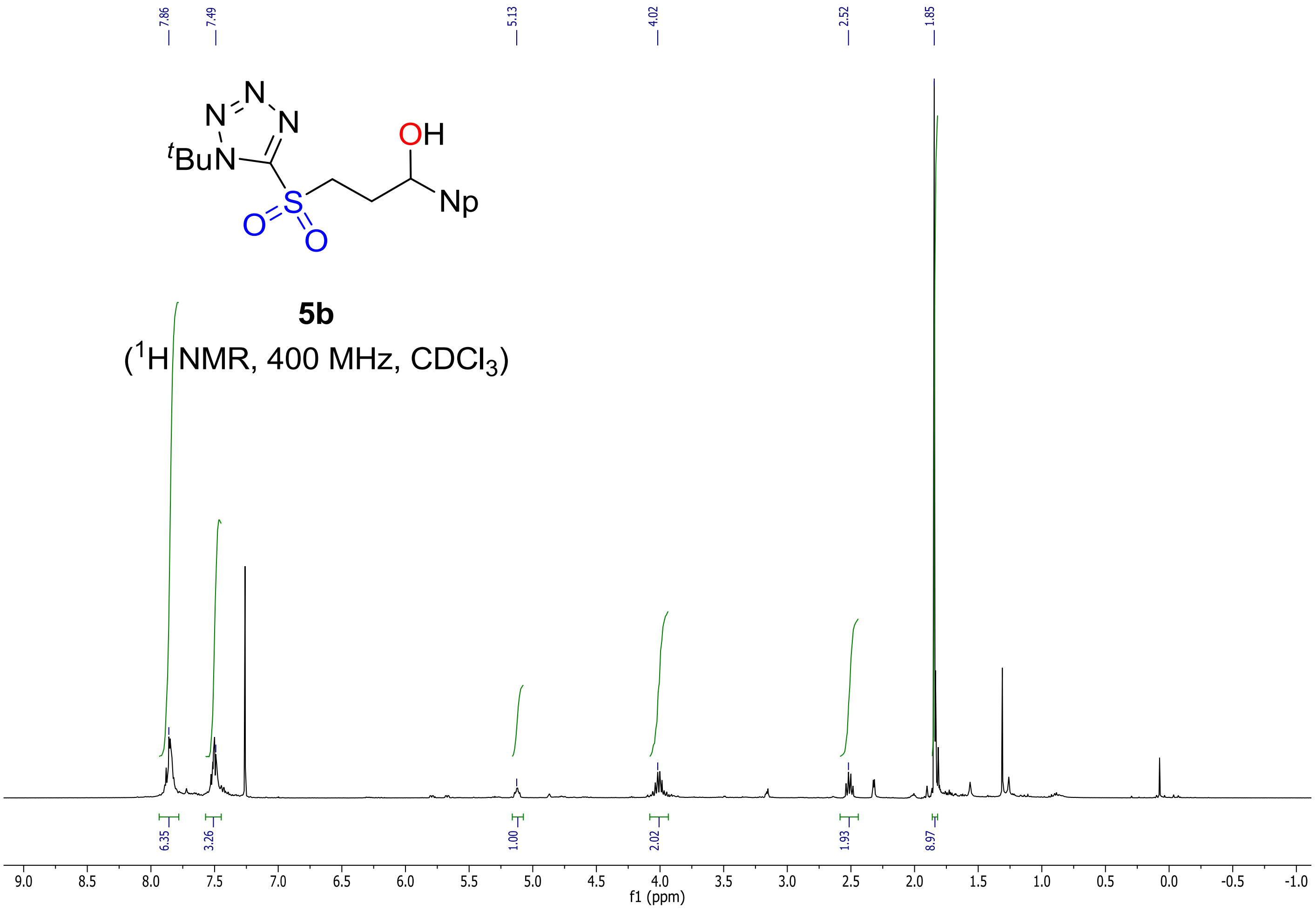

5b
(^{1}H NMR, 400 MHz, $CDCl_3$)

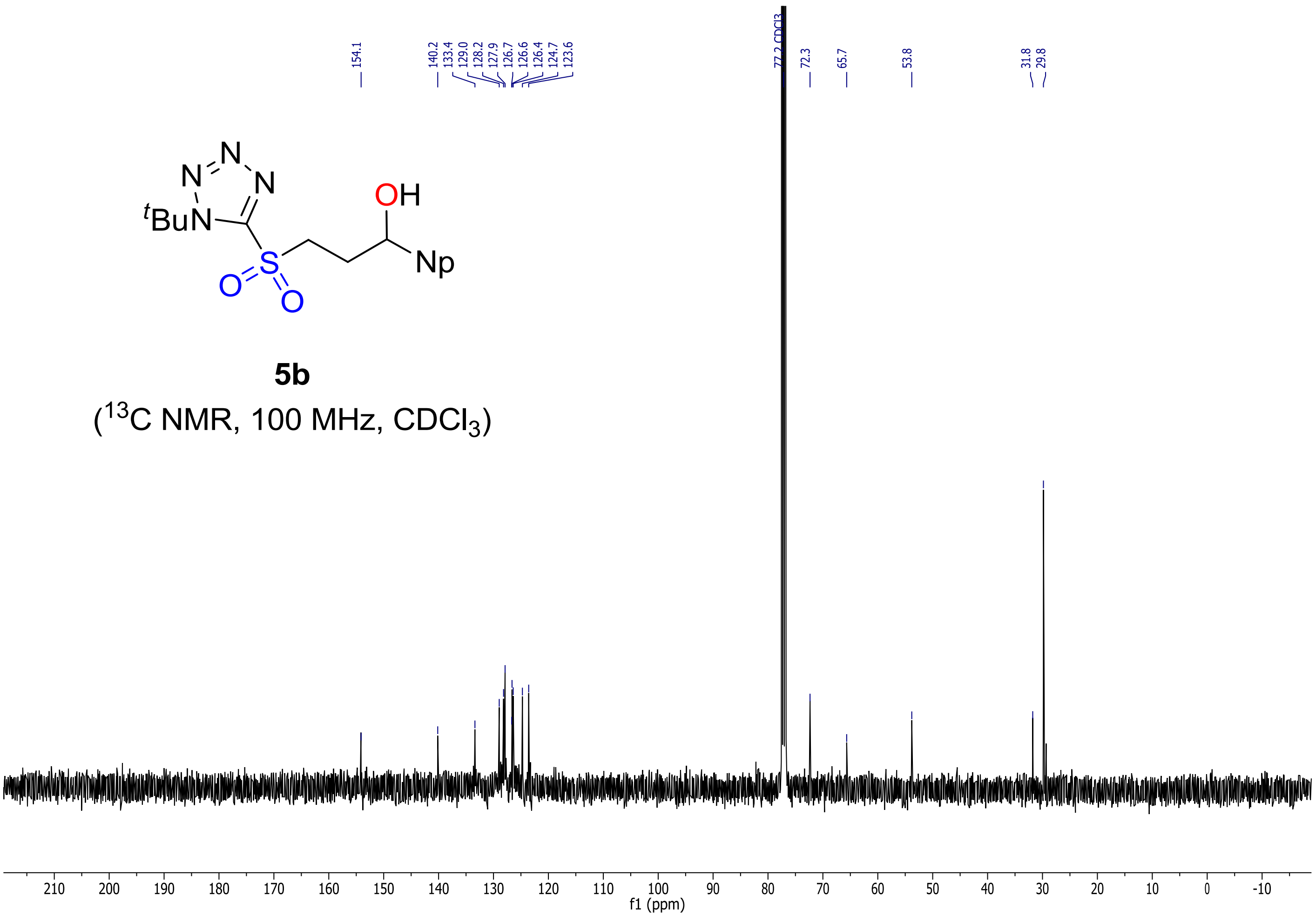

5b
(^{13}C NMR, 100 MHz, $CDCl_3$)

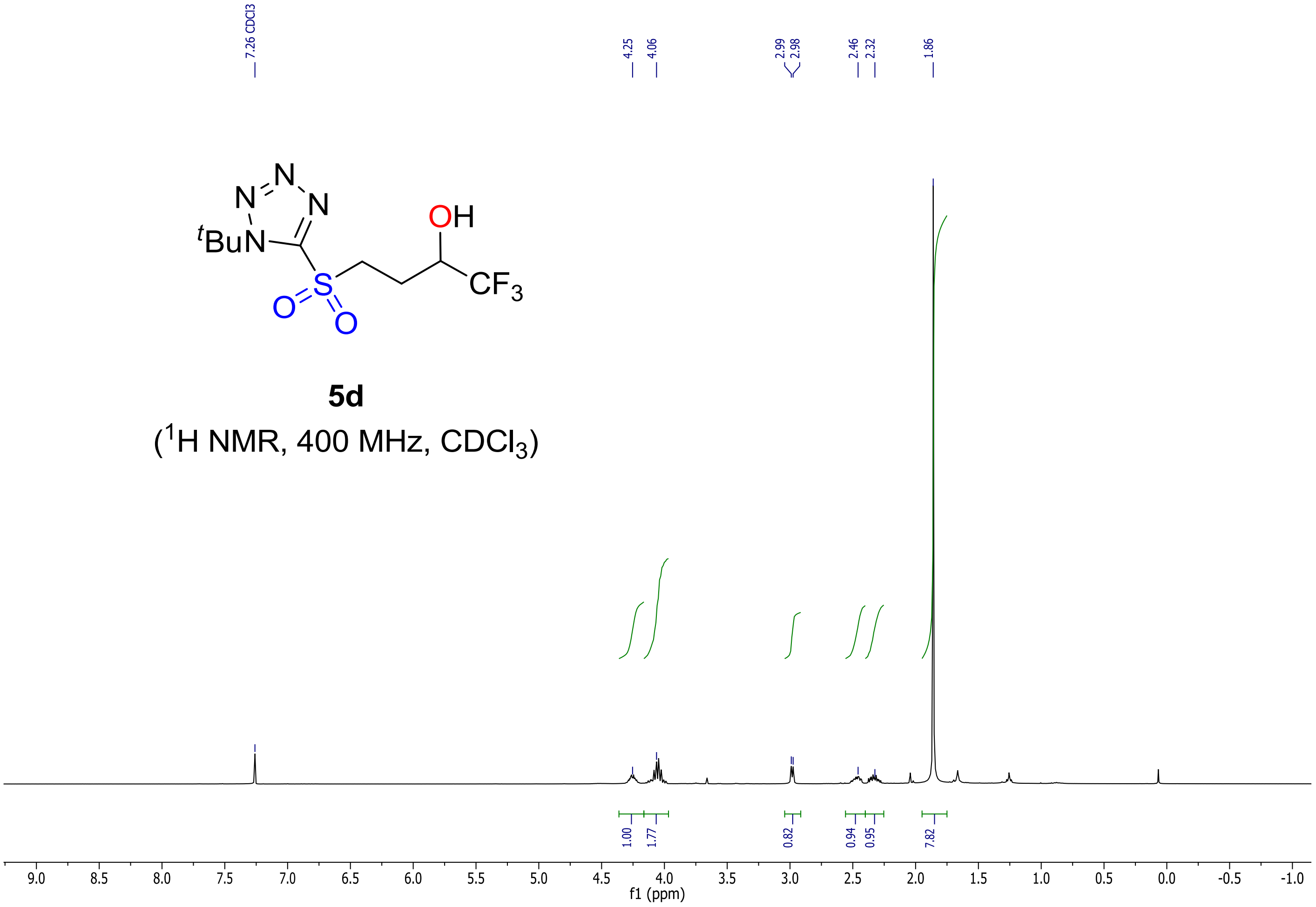

5d
(^{1}H NMR, 400 MHz, $CDCl_3$)

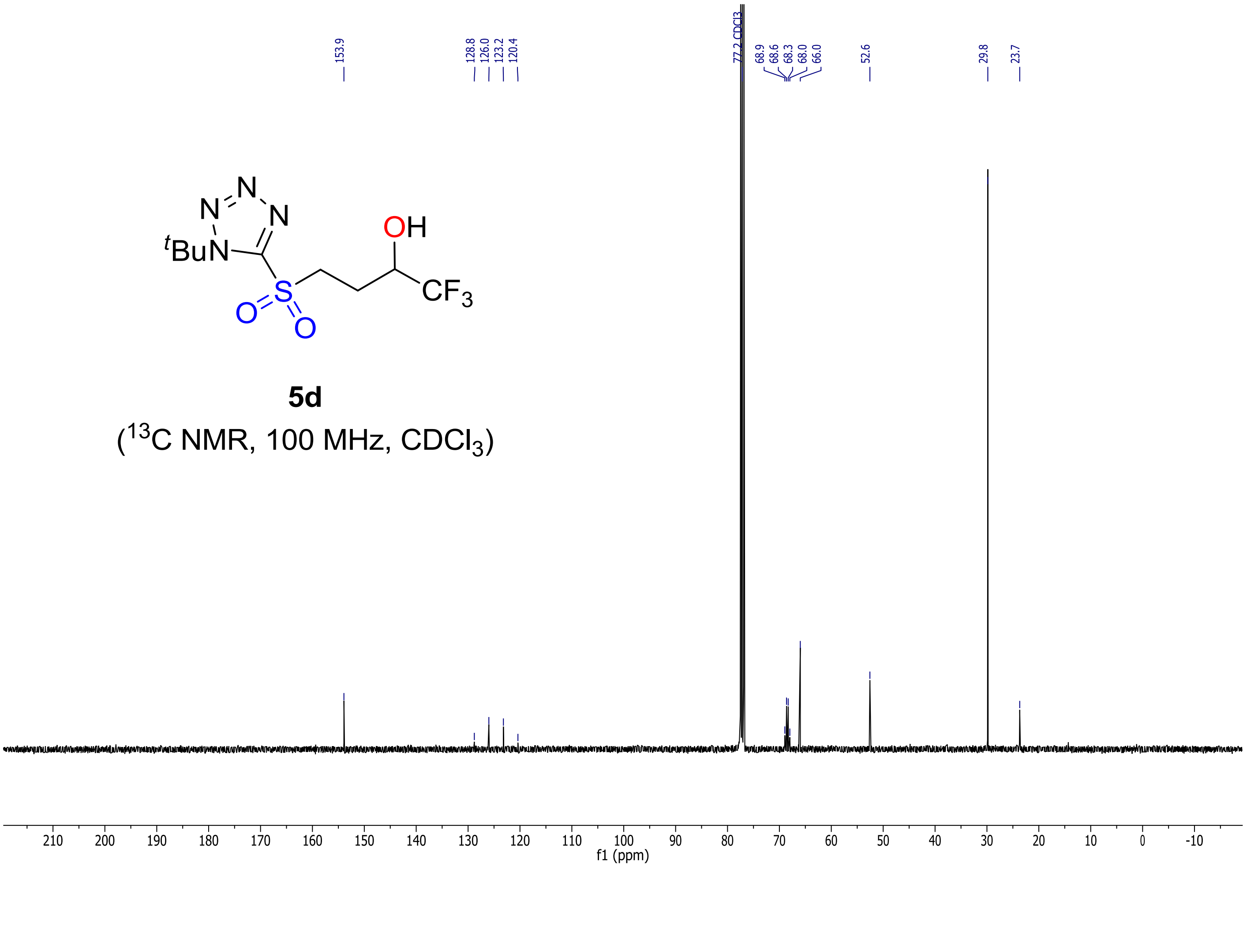

5d
(^{13}C NMR, 100 MHz, $CDCl_3$)

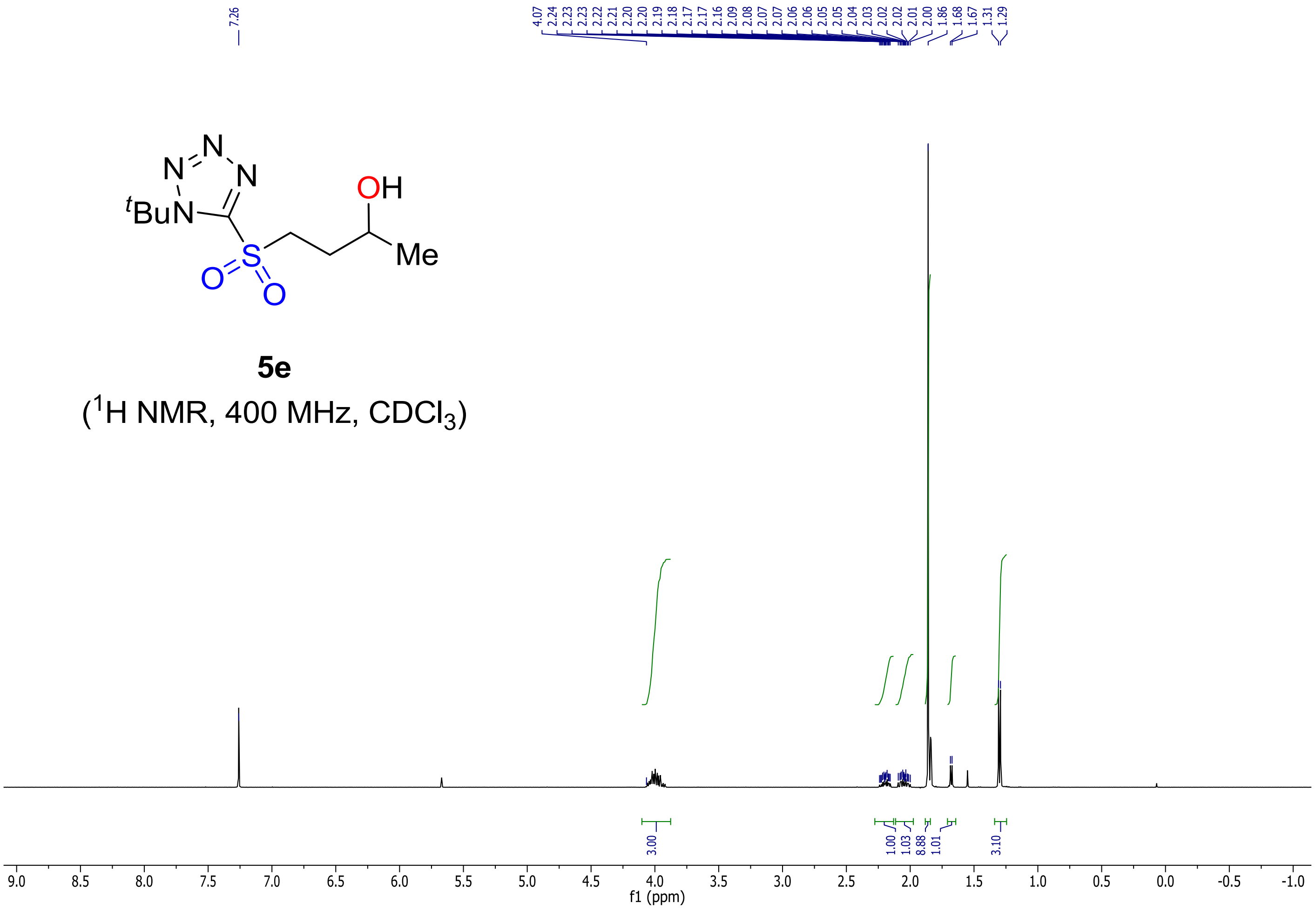

7.26
4.07 2.24 2.23 2.23 2.22 2.21 2.20 2.20 2.19 2.18 2.17 2.17 2.16 2.09 2.08 2.07 2.07 2.06 2.06 2.05 2.05 2.04 2.03 2.02 2.02 2.01 2.00 1.86 1.68 1.67 1.31 1.29
N
N
N
N
tBuN
OH
Me
S
O
O
5e
(1H NMR, 400 MHz, CDCl3)
3.00
1.00
1.03
8.88
1.01
3.10
9.0 8.5 8.0 7.5 7.0 6.5 6.0 5.5 5.0 4.5 4.0 3.5 3.0 2.5 2.0 1.5 1.0 0.5 0.0 -0.5 -1.0
f1 (ppm)

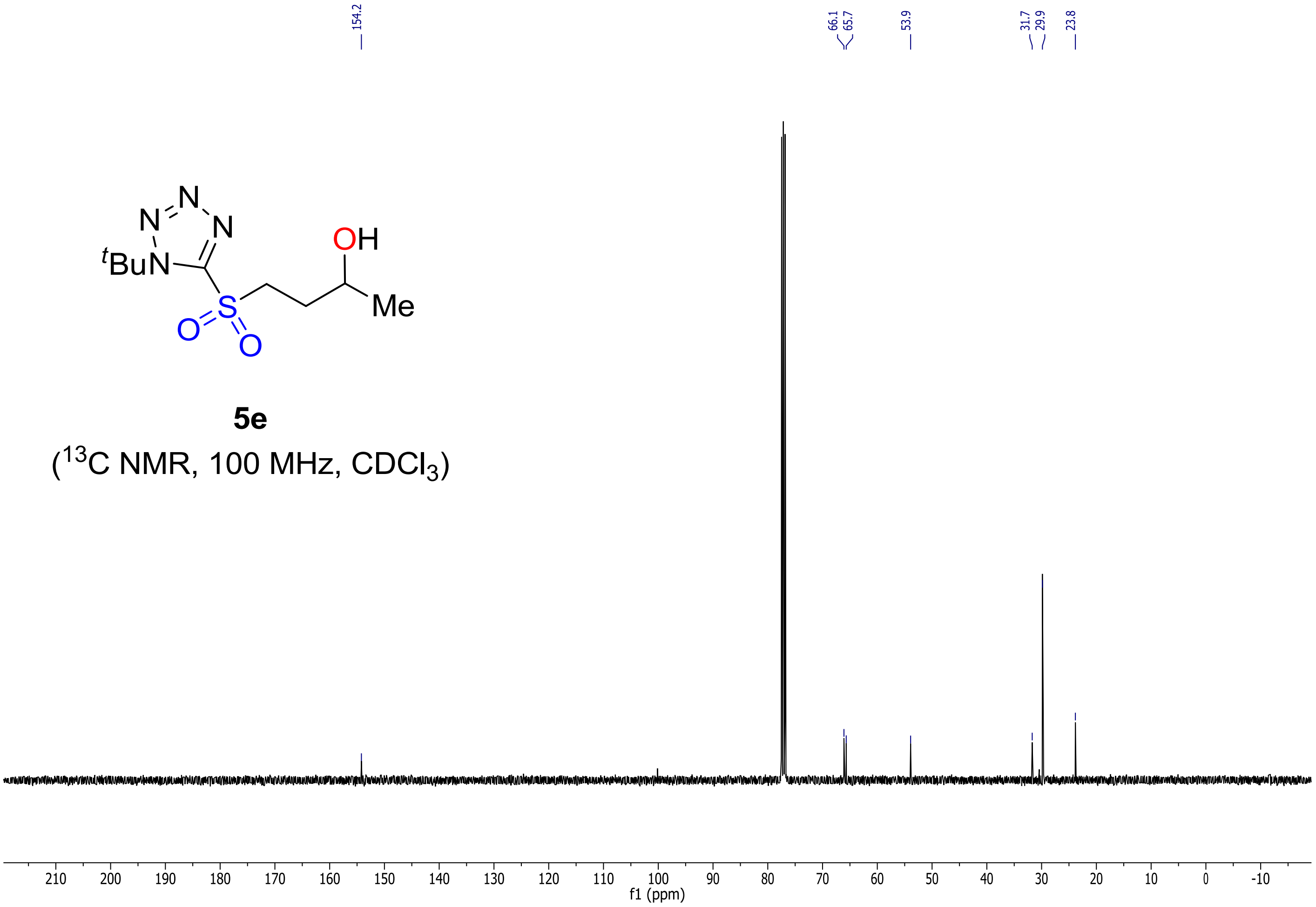

154.2
66.1 65.7
53.9
31.7 29.9
23.8
N
N
N
N
tBuN
OH
Me
S
O
O
5e
(13C NMR, 100 MHz, CDCl3)
210 200 190 180 170 160 150 140 130 120 110 100 90 80 70 60 50 40 30 20 10 0 -10
f1 (ppm)

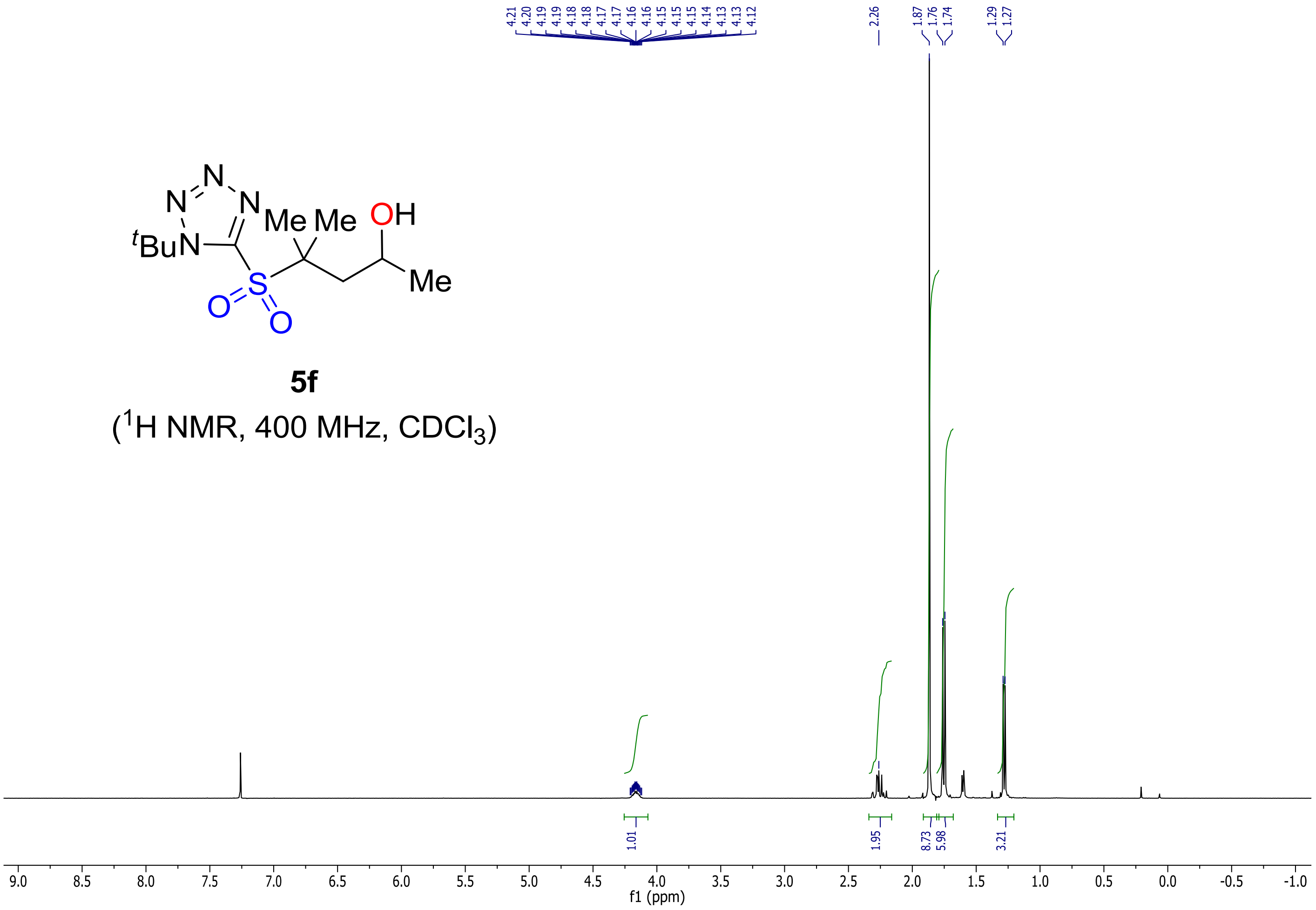

5f
(^{1}H NMR, 400 MHz, $CDCl_3$)

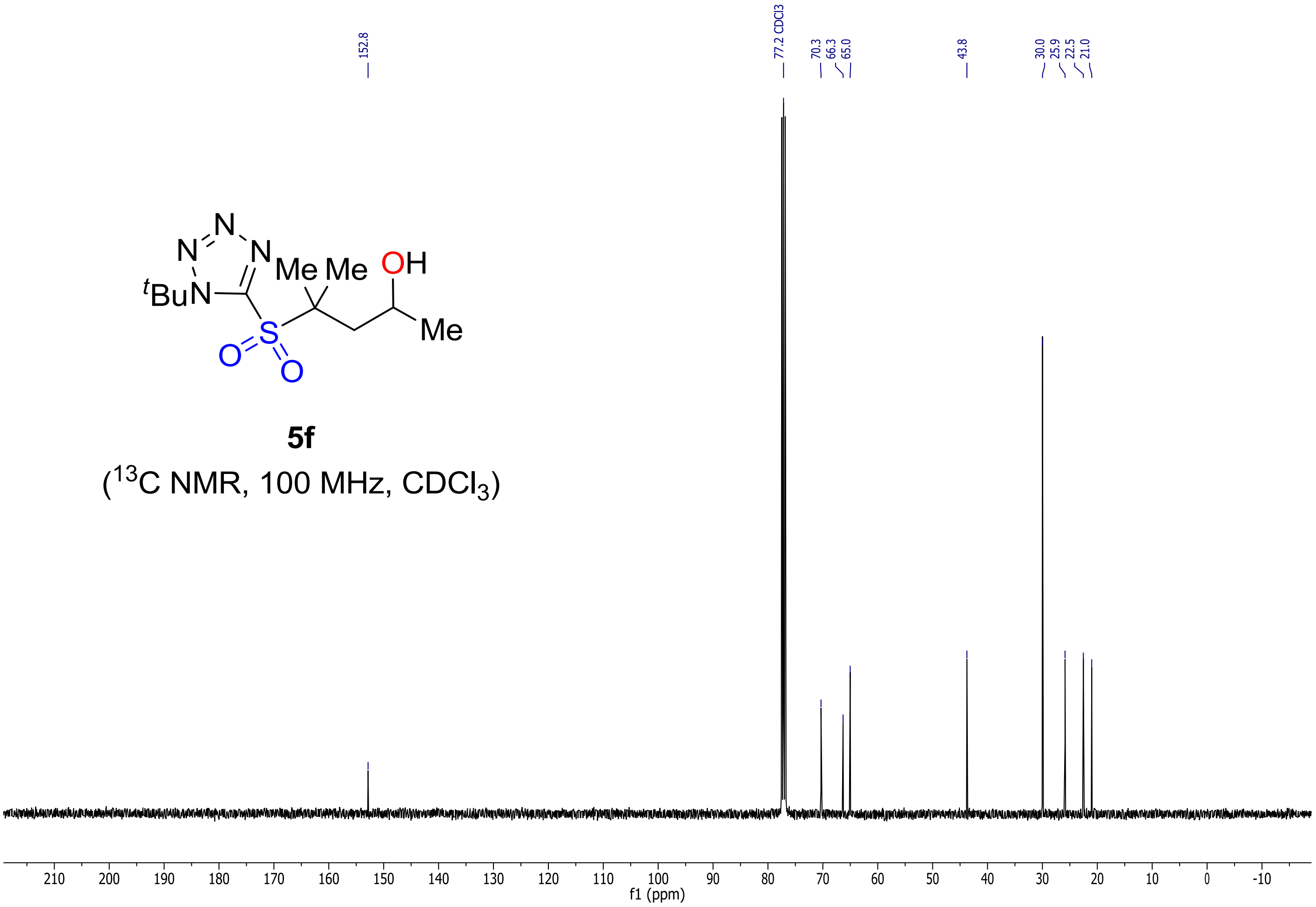

5f
(^{13}C NMR, 100 MHz, $CDCl_3$)

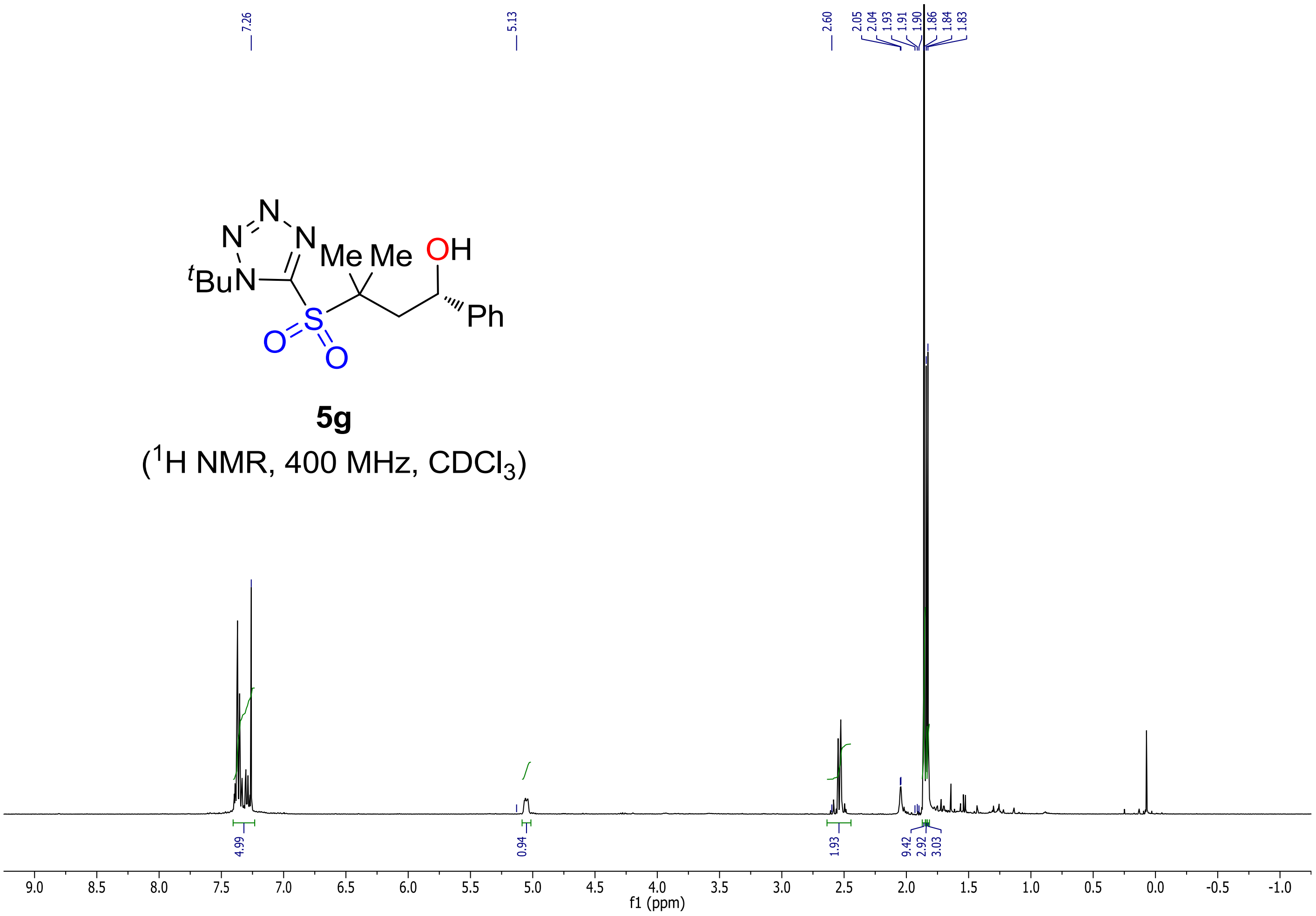
7.26
5.13
2.60
2.05
2.04
1.93
1.91
1.90
1.86
1.84
1.83
N
N
N
tBuN
Me Me
OH
Ph
S
O
O
5g
(1H NMR, 400 MHz, CDCl3)
4.99
0.94
1.93
9.42
2.92
3.03
f1 (ppm)

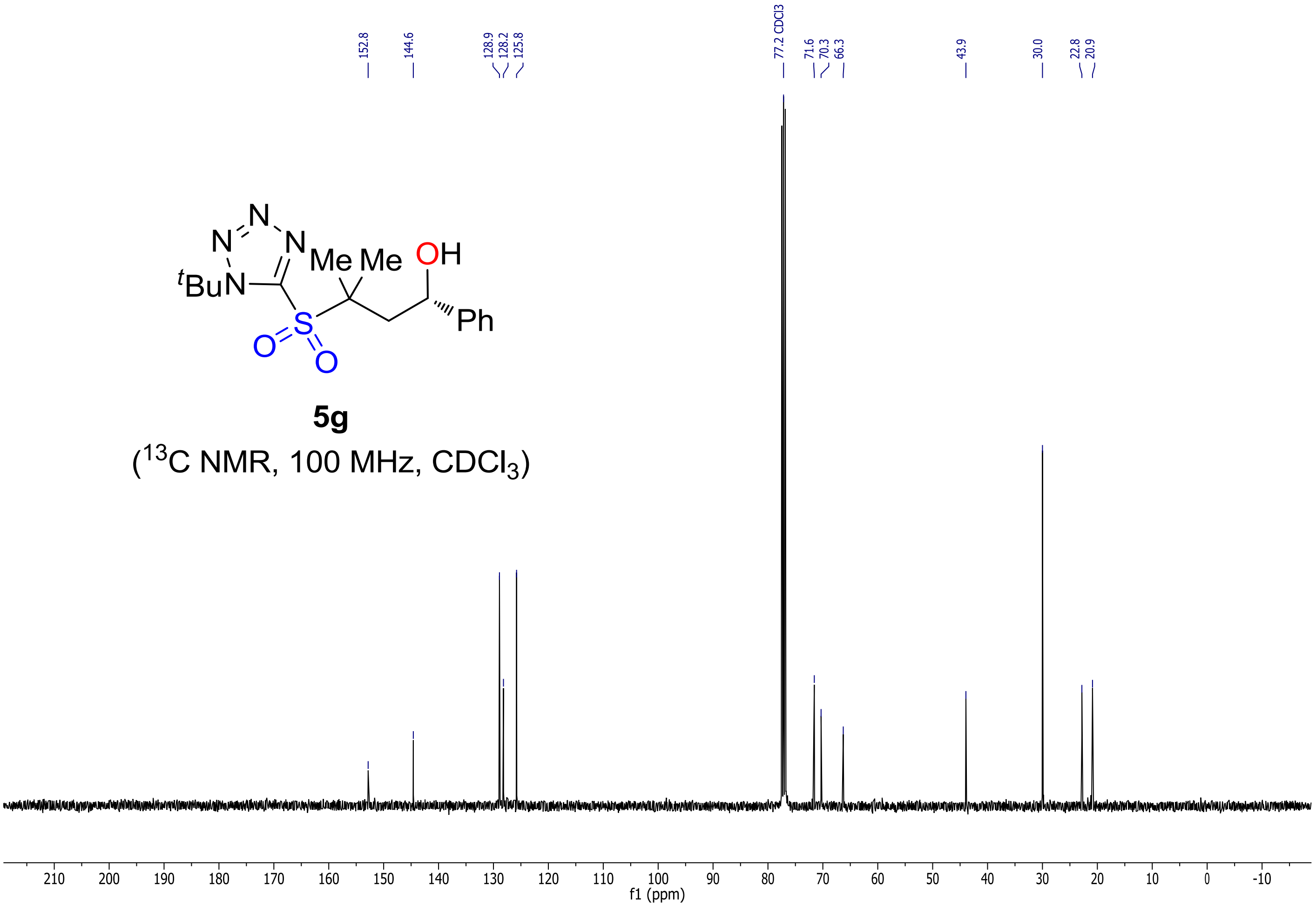
152.8
144.6
128.9
128.2
125.8
77.2 CDCl3
71.6
70.3
66.3
43.9
30.0
22.8
20.9
N
N
N
tBuN
Me Me
OH
Ph
S
O
O
5g
(13C NMR, 100 MHz, CDCl3)
f1 (ppm)

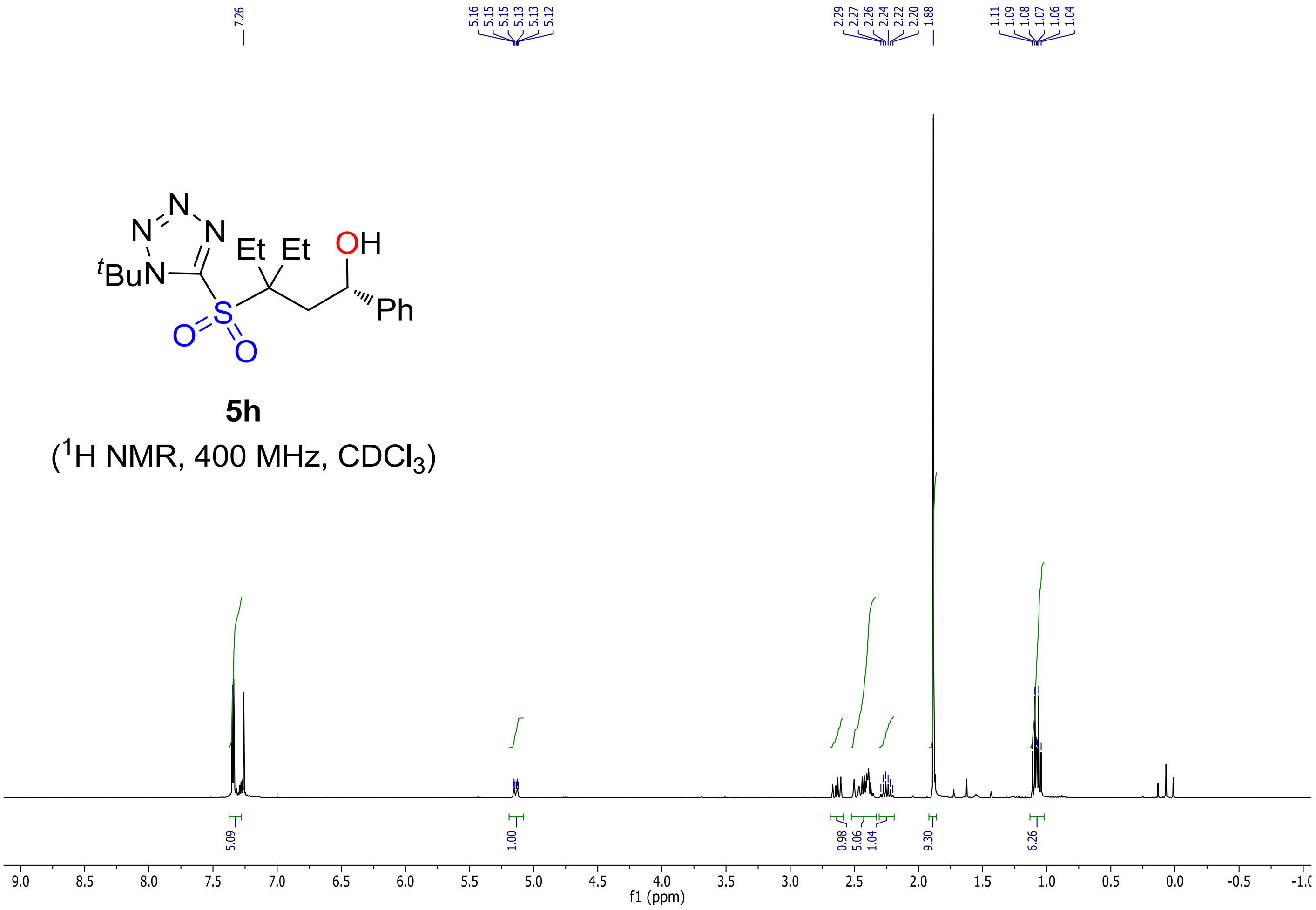
7.26
5.16
5.15
5.15
5.13
5.13
5.12
2.29
2.27
2.26
2.24
2.22
2.20
1.88
1.11
1.09
1.08
1.07
1.06
1.04
Et Et OH
tBuN
Ph
5h
(1H NMR, 400 MHz, CDCl3)
5.09
1.00
0.98
5.06
1.04
9.30
6.26
f1 (ppm)

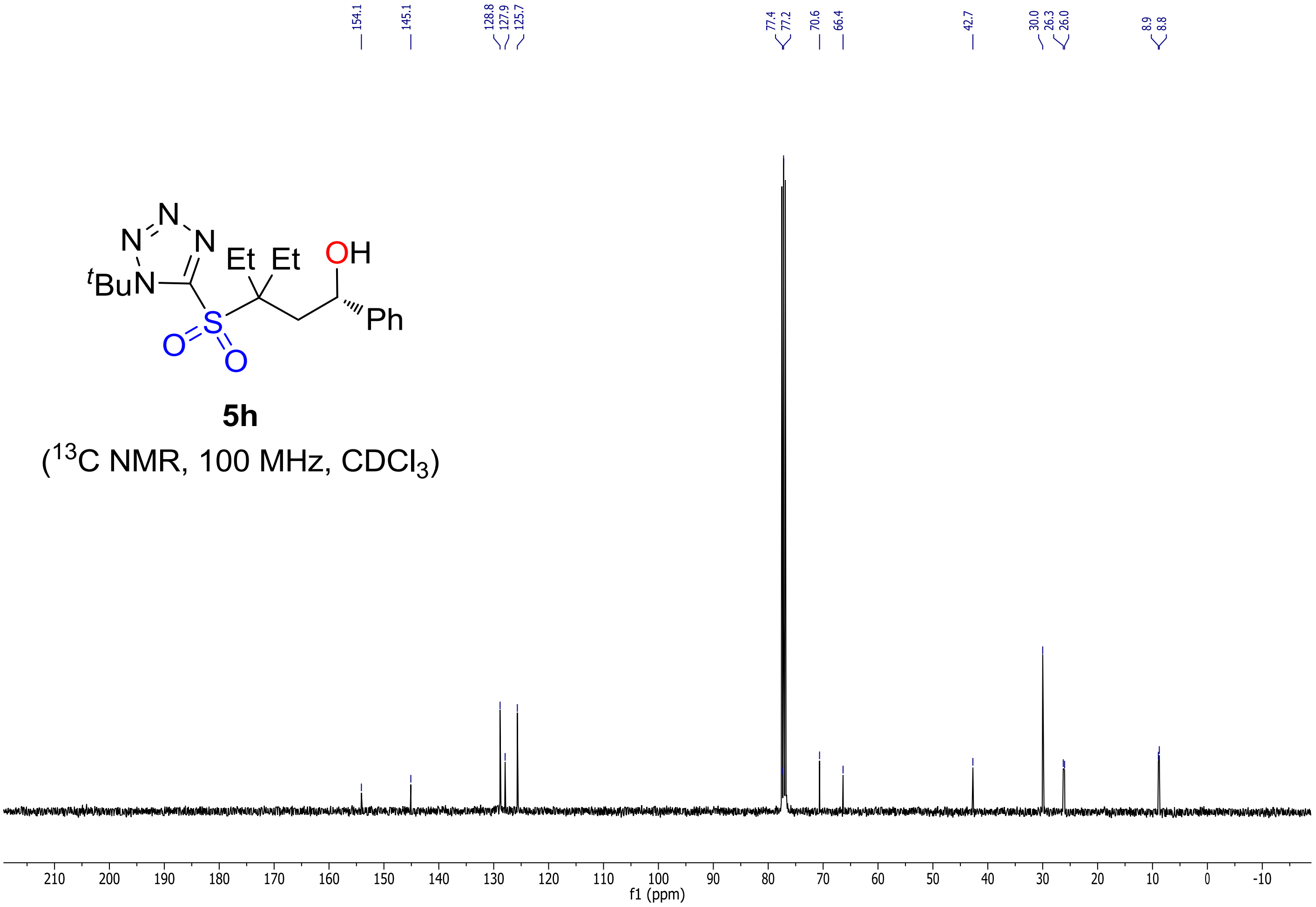
154.1
145.1
128.8
127.9
125.7
77.4
77.2
70.6
66.4
42.7
30.0
26.3
26.0
8.9
8.8
Et Et OH
tBuN
Ph
5h
(13C NMR, 100 MHz, CDCl3)
f1 (ppm)

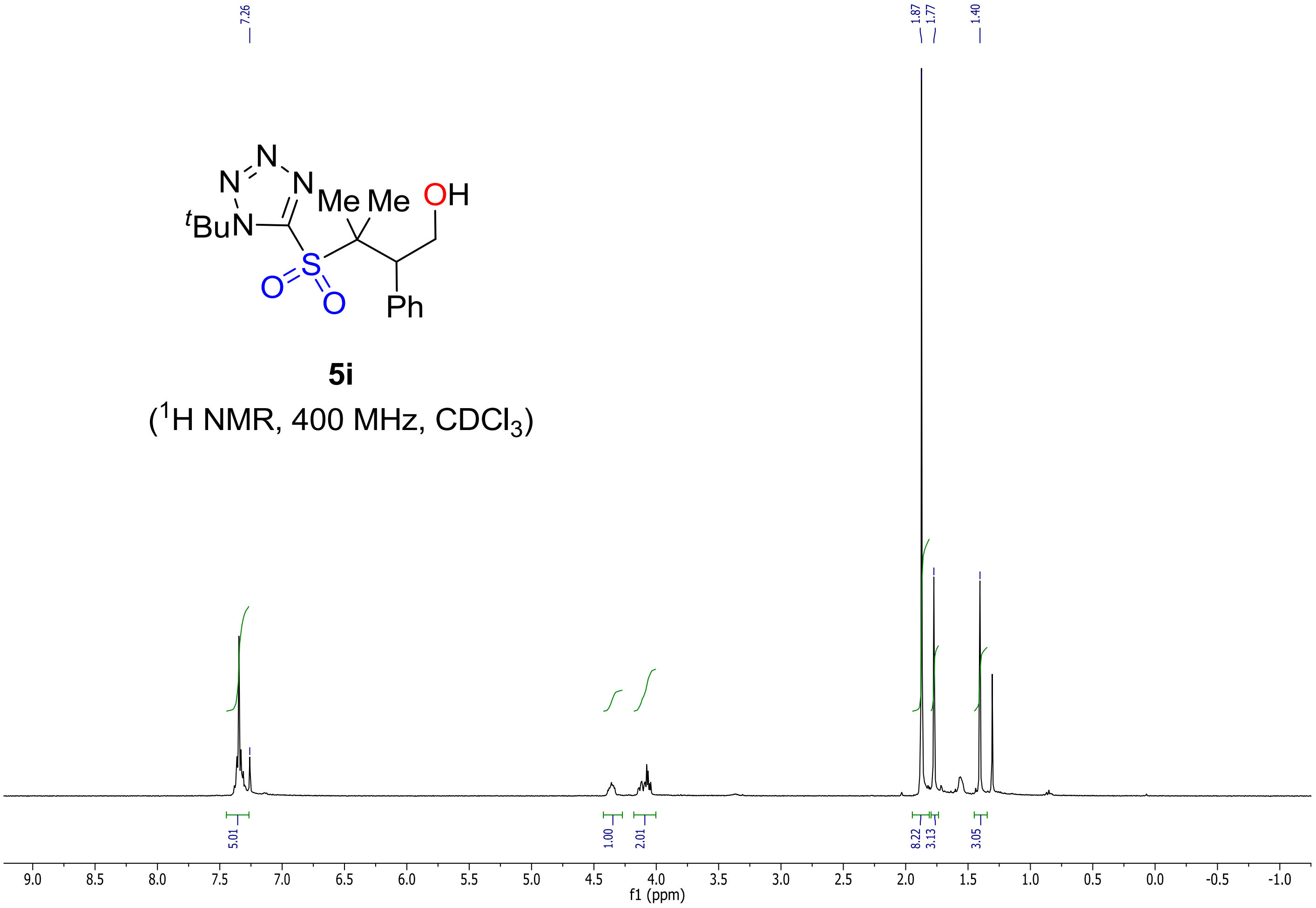
7.26
1.87
1.77
1.40
N
N
N
N
^{t}BuN
Me Me
OH
S
O
O
Ph
5i
(^{1}H NMR, 400 MHz, $CDCl_3$)
5.01
1.00
2.01
8.22
3.13
3.05
f1 (ppm)

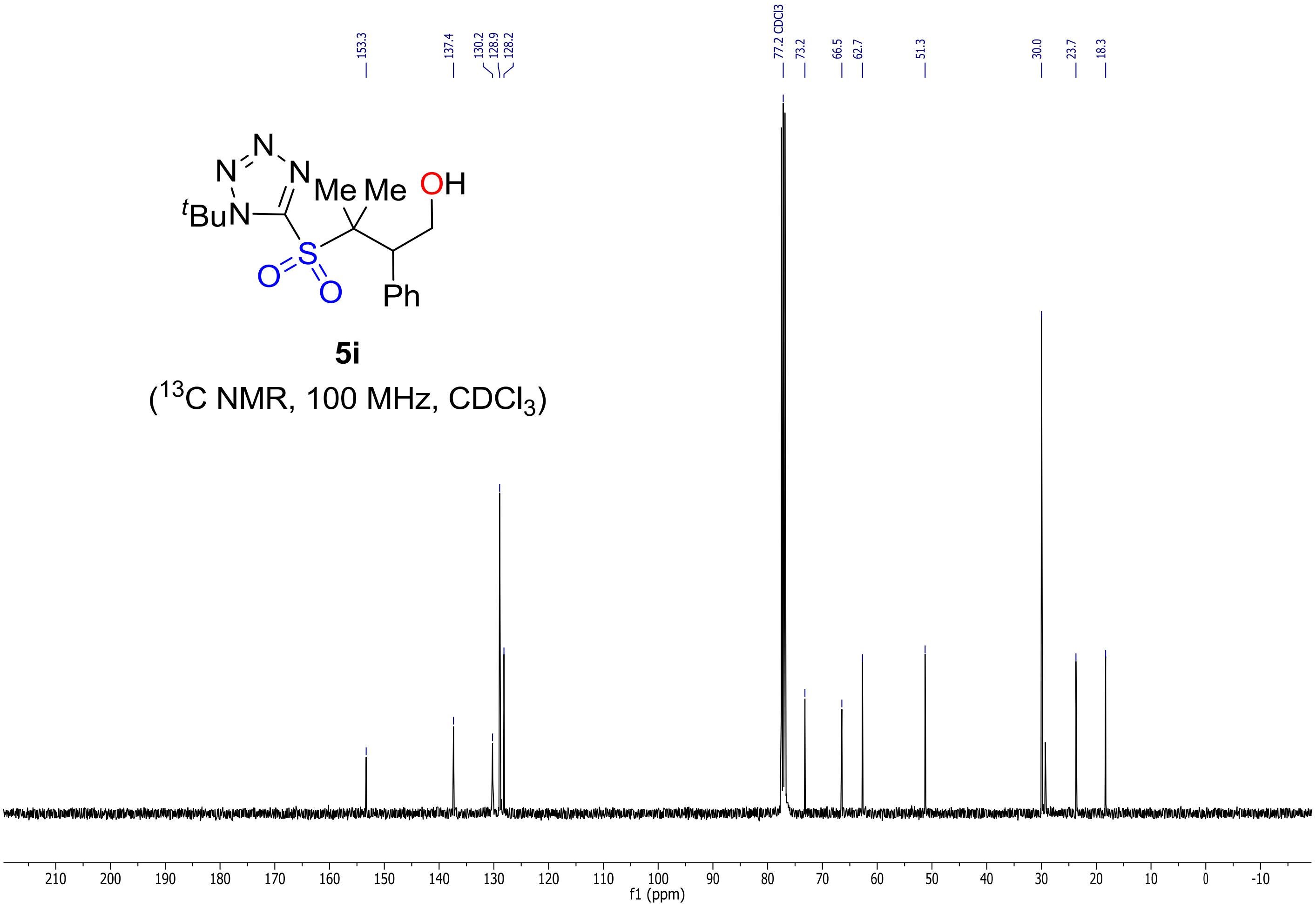
153.3
137.4
130.2
128.9
128.2
77.2 CDCl3
73.2
66.5
62.7
51.3
30.0
23.7
18.3
N
N
N
N
^{t}BuN
Me Me
OH
S
O
O
Ph
5i
(^{13}C NMR, 100 MHz, $CDCl_3$)
f1 (ppm)

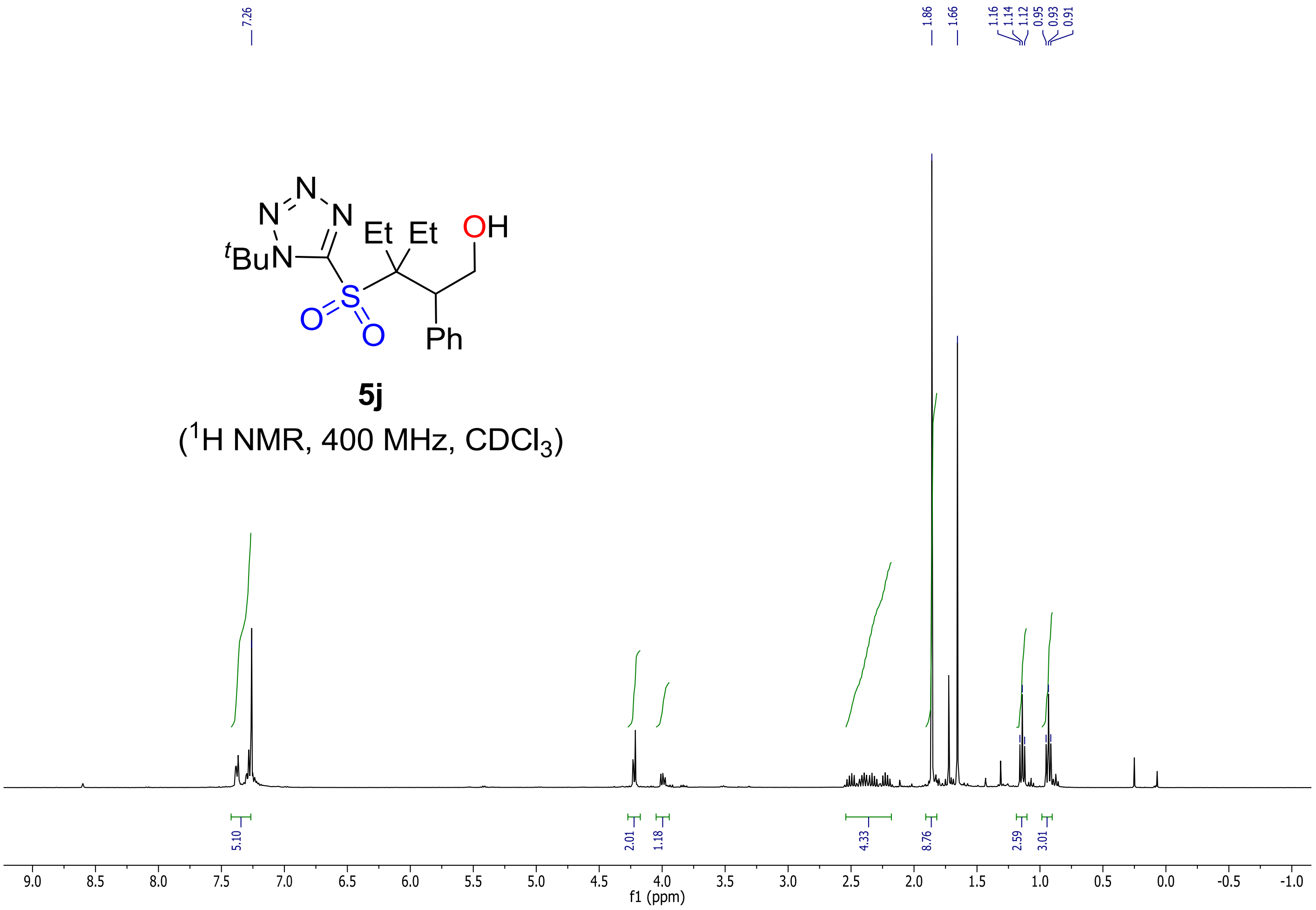
7.26
1.86
1.66
1.16
1.14
1.12
0.95
0.93
0.91
N N N N
ᵗBuN
Et Et OH
S O O
Ph
5j
(¹H NMR, 400 MHz, CDCl₃)
5.10
2.01
1.18
4.33
8.76
2.59
3.01
9.0 8.5 8.0 7.5 7.0 6.5 6.0 5.5 5.0 4.5 4.0 3.5 3.0 2.5 2.0 1.5 1.0 0.5 0.0 -0.5 -1.0
f1 (ppm)

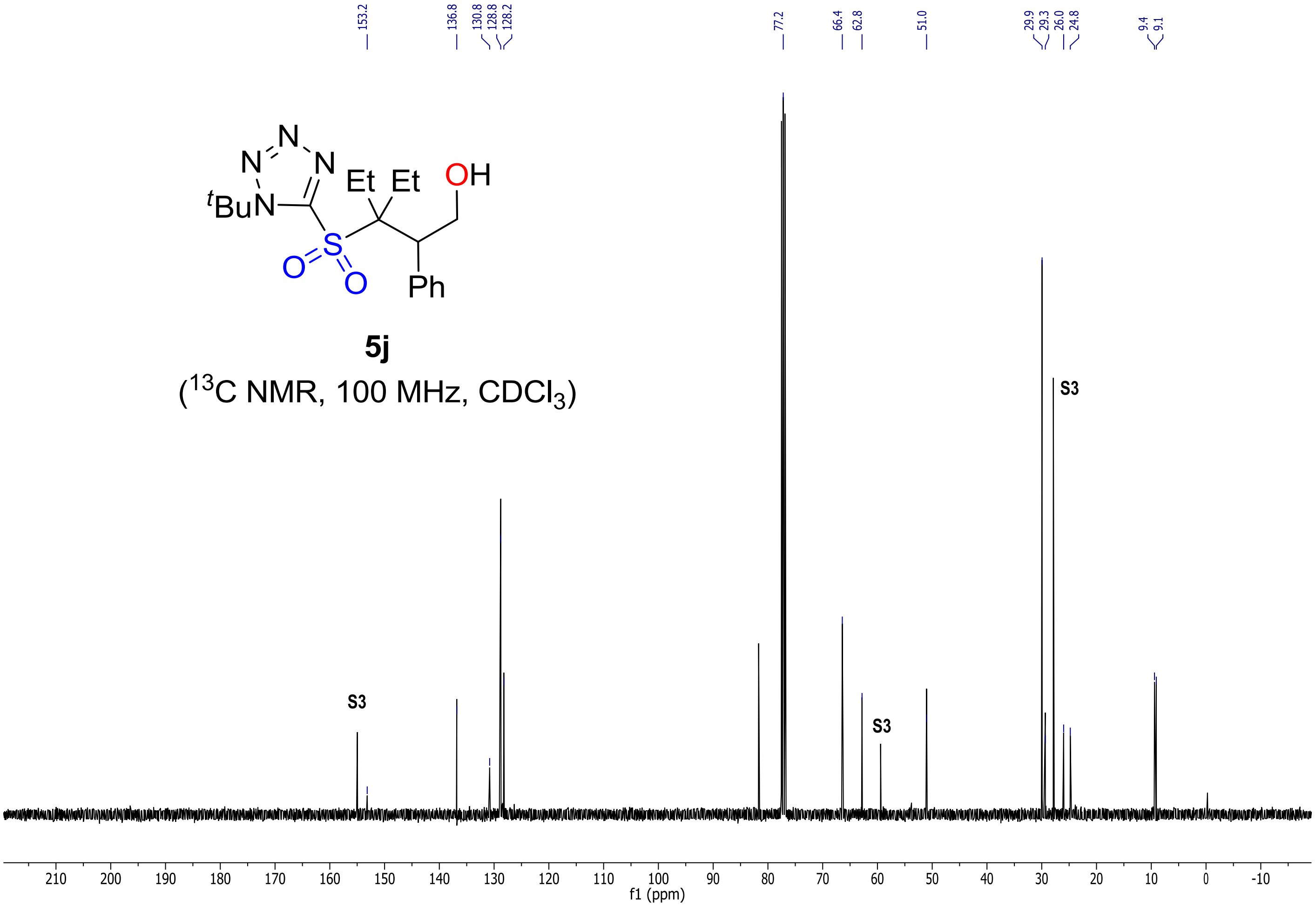
153.2
136.8
130.8
128.8
128.2
77.2
66.4
62.8
51.0
29.9
29.3
26.0
24.8
9.4
9.1
N N N N
ᵗBuN
Et Et OH
S O O
Ph
5j
(¹³C NMR, 100 MHz, CDCl₃)
S3
S3
S3
210 200 190 180 170 160 150 140 130 120 110 100 90 80 70 60 50 40 30 20 10 0 -10
f1 (ppm)

10. ^{1}H and ^{13}C NMR data for sultines 7a-j

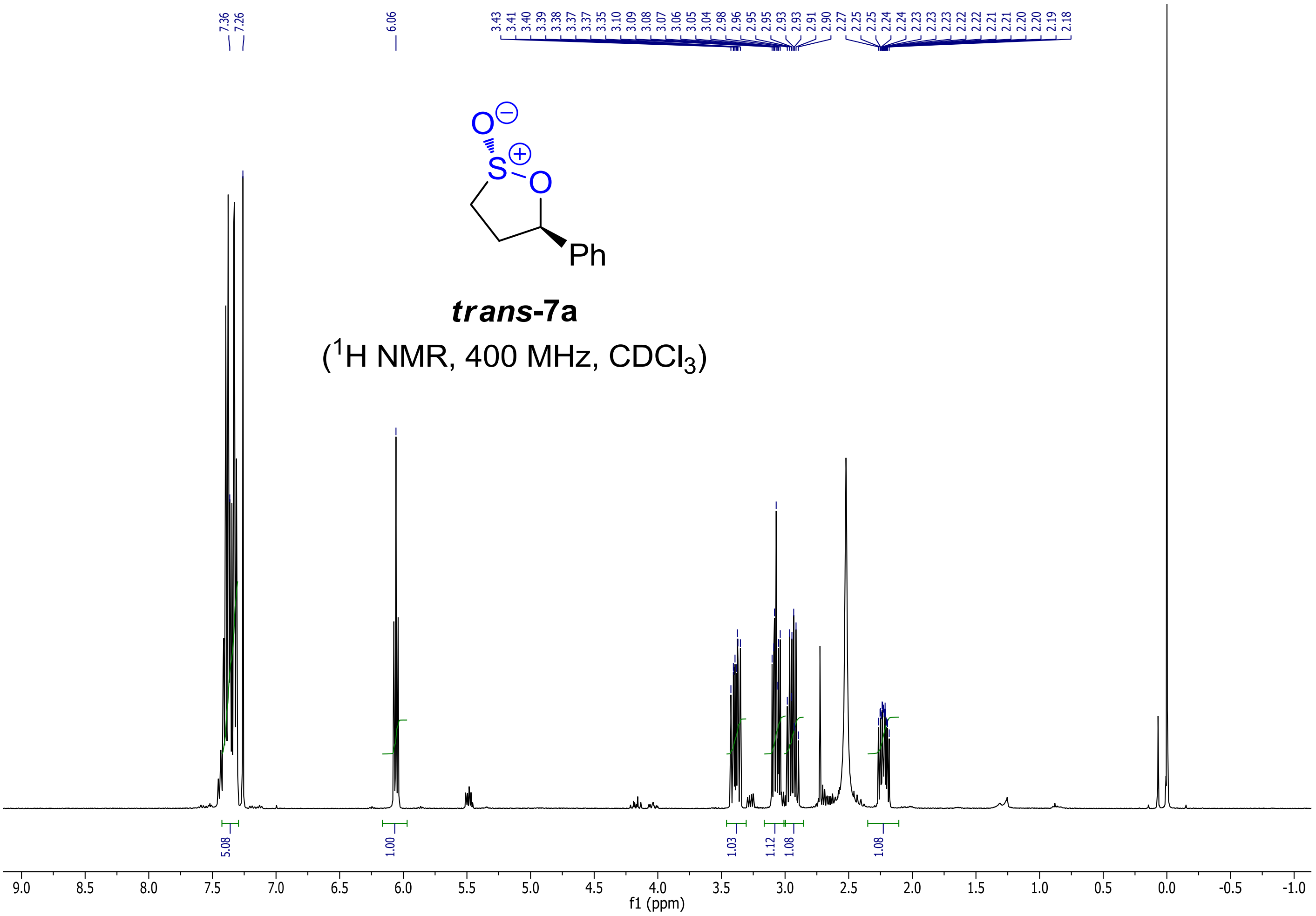

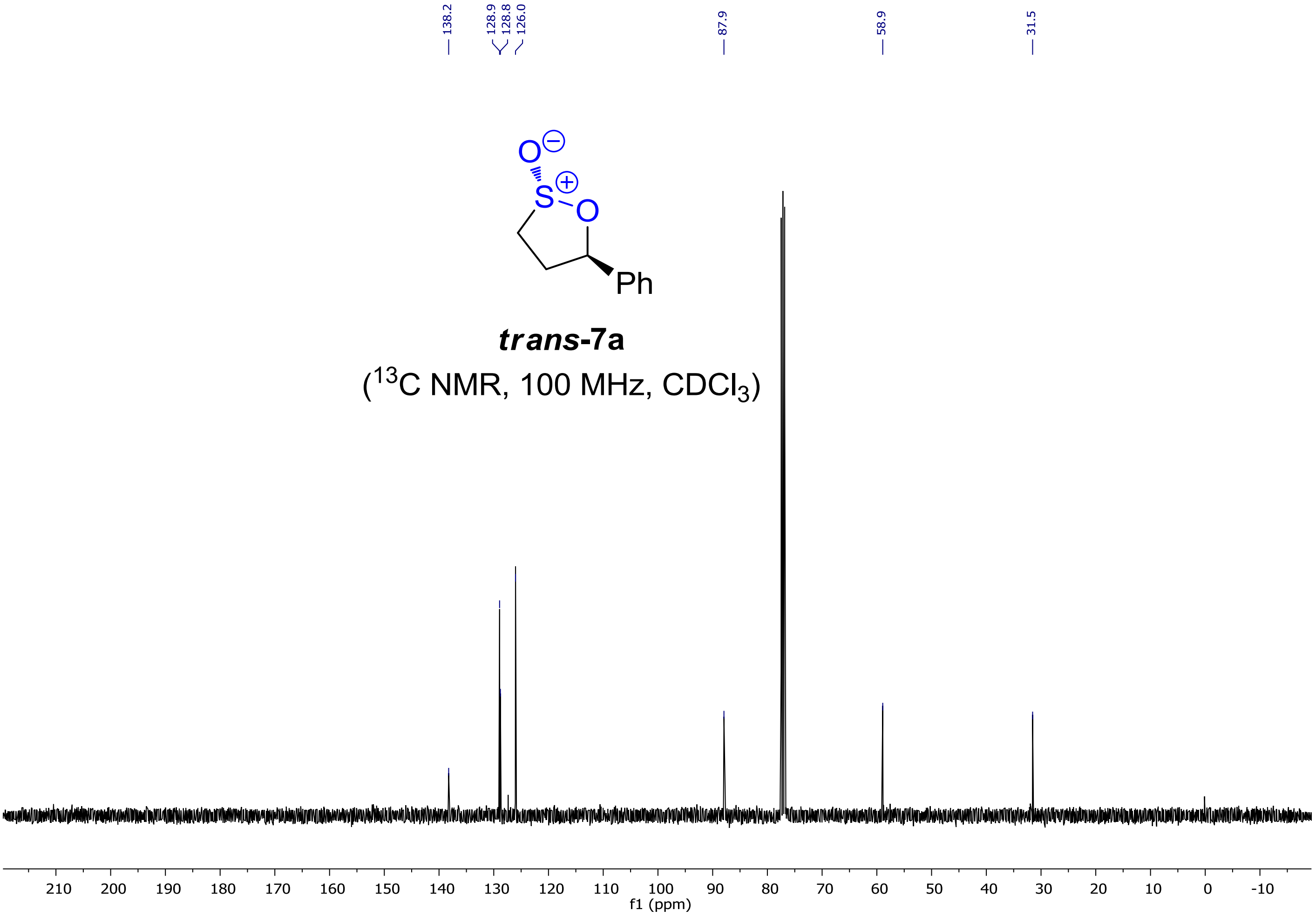

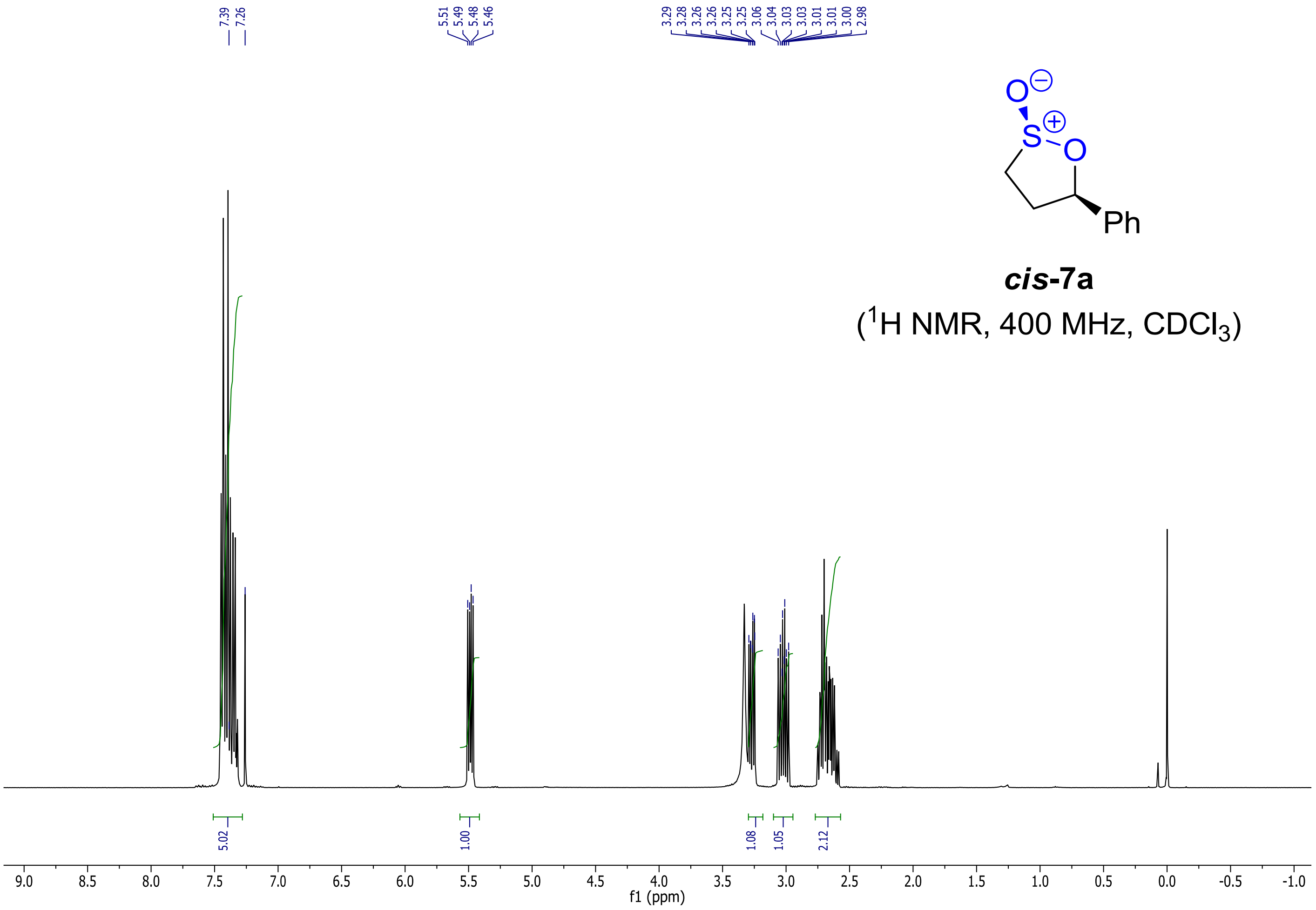
7.39
7.26
5.51
5.49
5.48
5.46
3.29
3.28
3.26
3.26
3.25
3.25
3.06
3.04
3.03
3.03
3.01
3.01
3.00
2.98
O
S
O
Ph
cis-7a
(1H NMR, 400 MHz, CDCl3)
5.02
1.00
1.08
1.05
2.12
9.0 8.5 8.0 7.5 7.0 6.5 6.0 5.5 5.0 4.5 4.0 3.5 3.0 2.5 2.0 1.5 1.0 0.5 0.0 -0.5 -1.0
f1 (ppm)

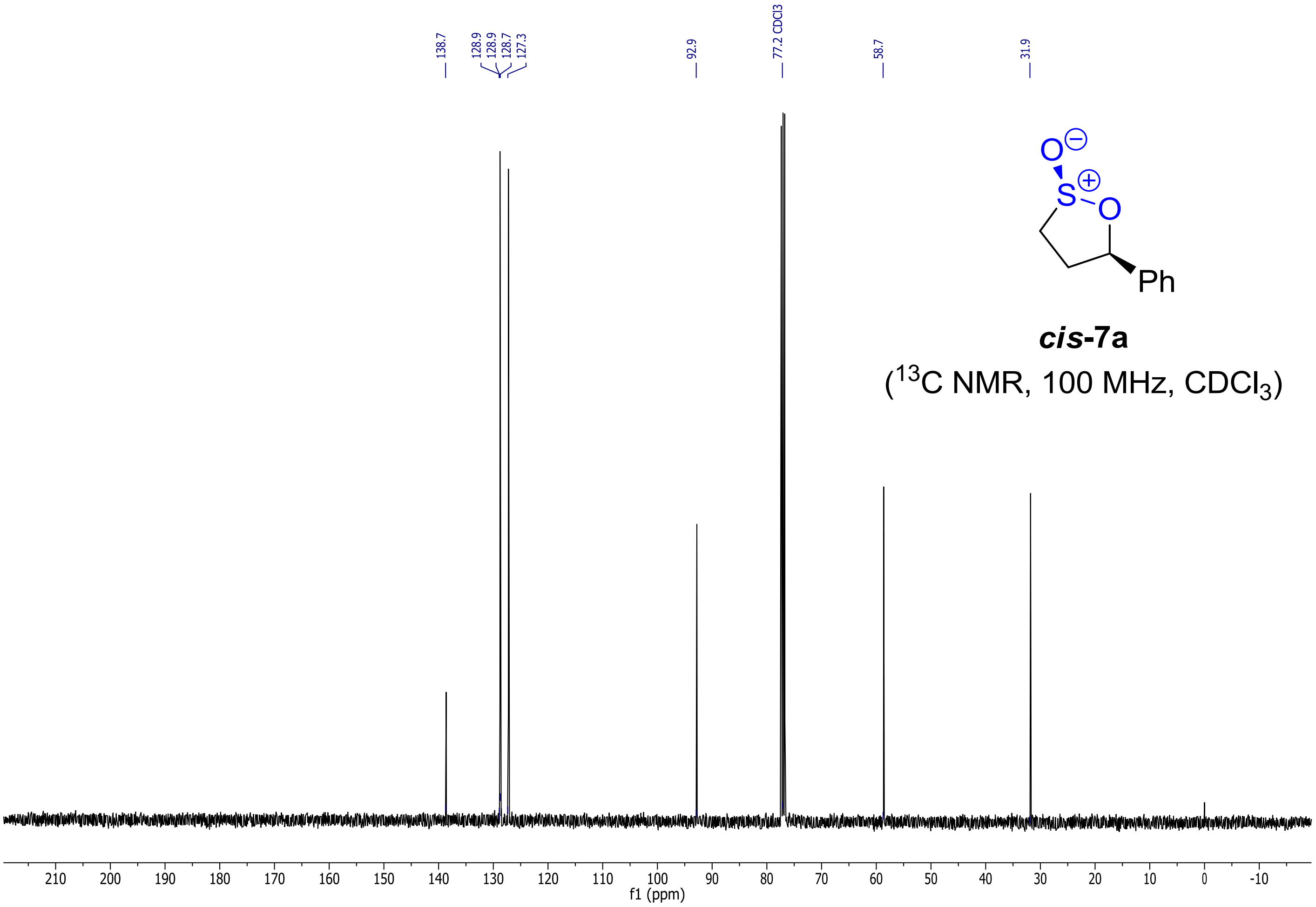
138.7
128.9
128.9
128.7
127.3
92.9
77.2 CDCl3
58.7
31.9
O
S
O
Ph
cis-7a
(13C NMR, 100 MHz, CDCl3)
210 200 190 180 170 160 150 140 130 120 110 100 90 80 70 60 50 40 30 20 10 0 -10
f1 (ppm)

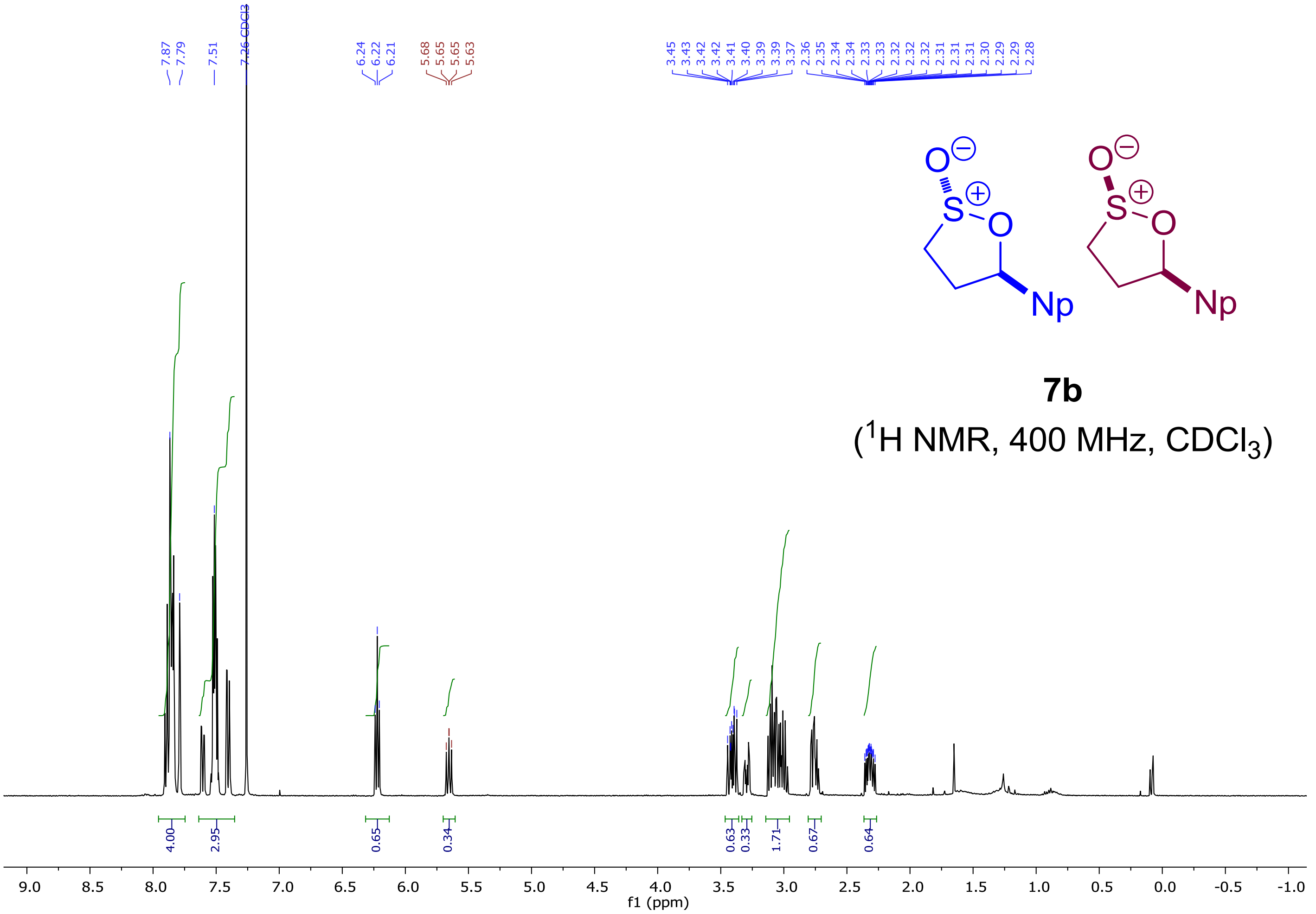
Np
Np
7b
(^{1}H NMR, 400 MHz, $CDCl_3$)
f1 (ppm)

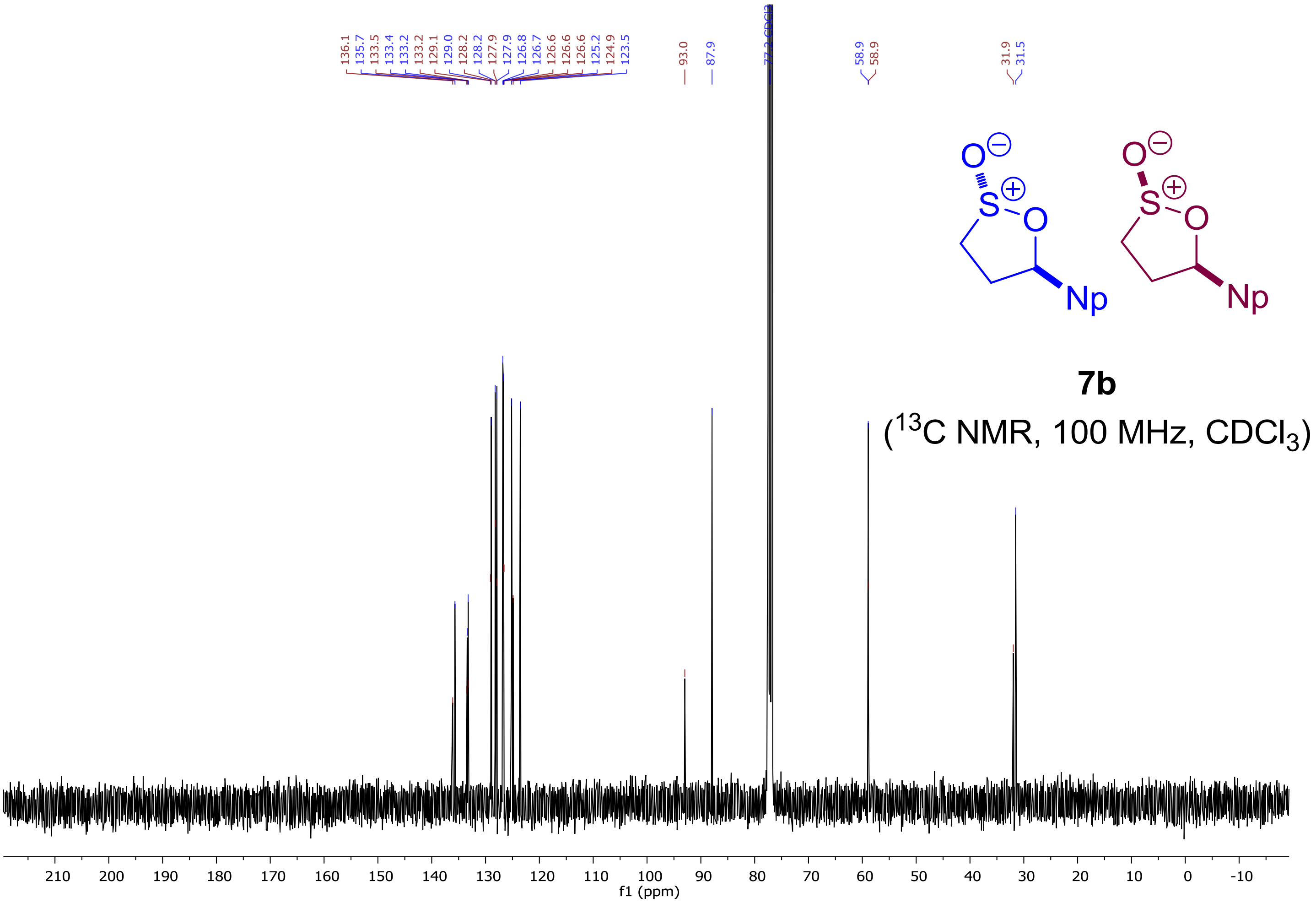
Np
Np
7b
(^{13}C NMR, 100 MHz, $CDCl_3$)
f1 (ppm)

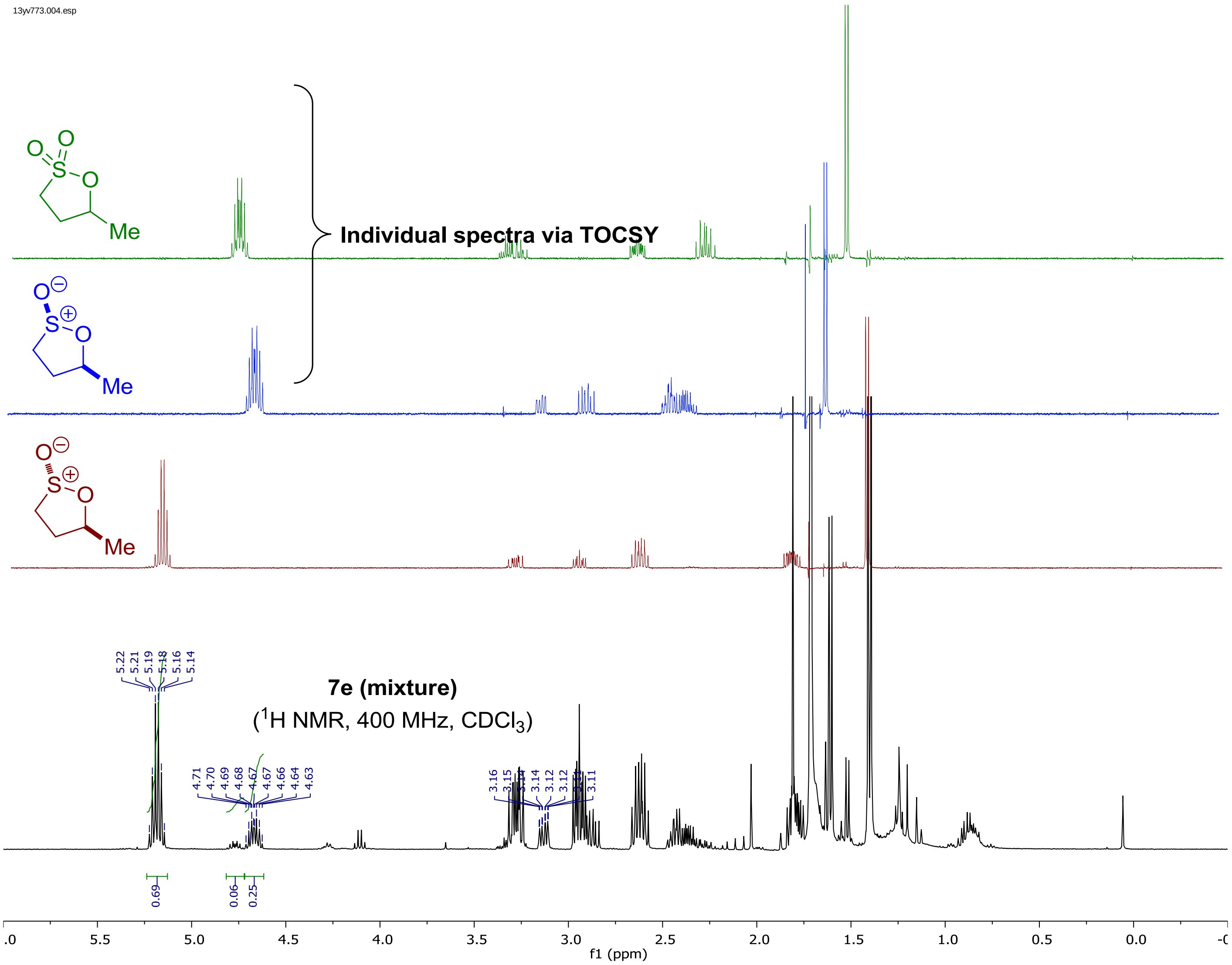
13yv773.004.esp
Individual spectra via TOCSY
Me
Me
Me
7e (mixture)
(1H NMR, 400 MHz, CDCl3)
f1 (ppm)

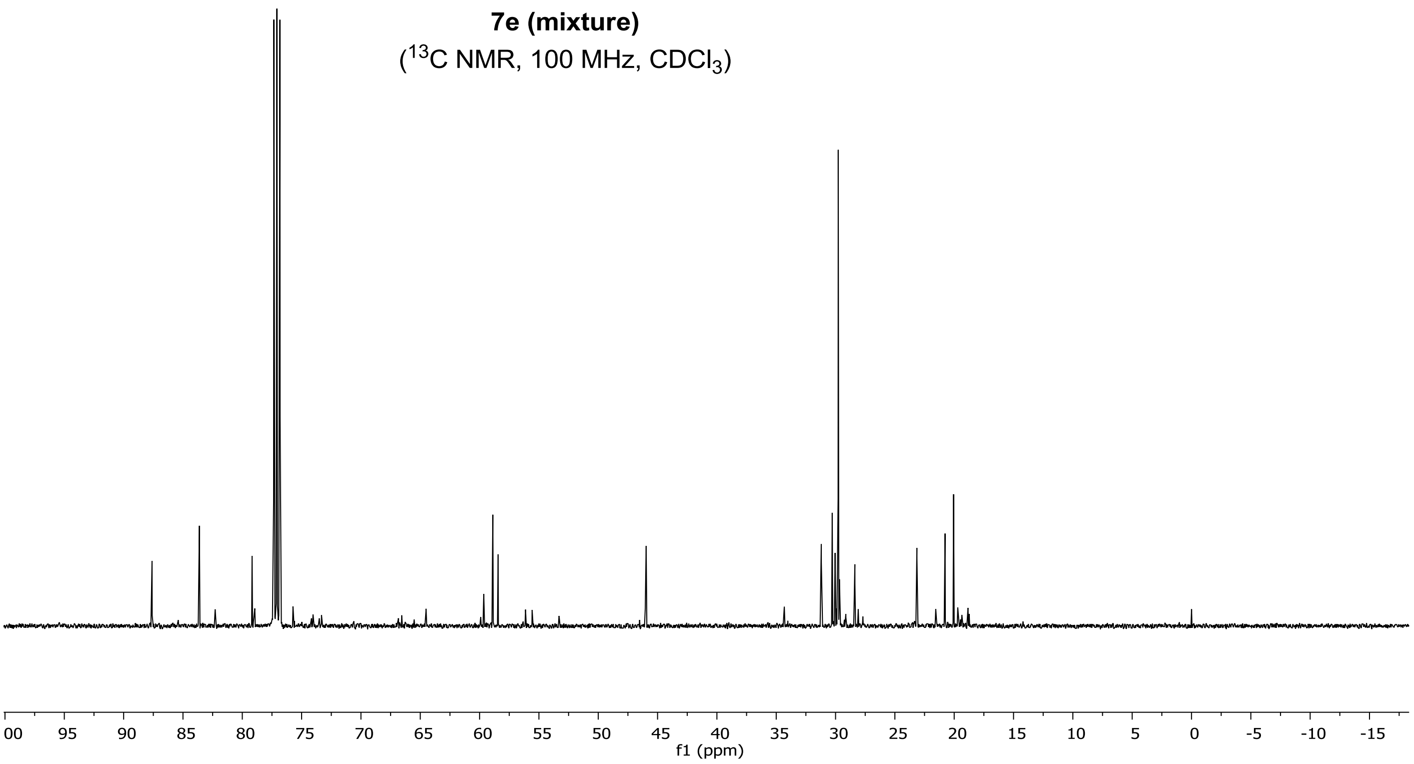
7e (mixture)
(13C NMR, 100 MHz, CDCl3)
f1 (ppm)

7f

(^{1}H NMR, 400 MHz, $CDCl_3$)

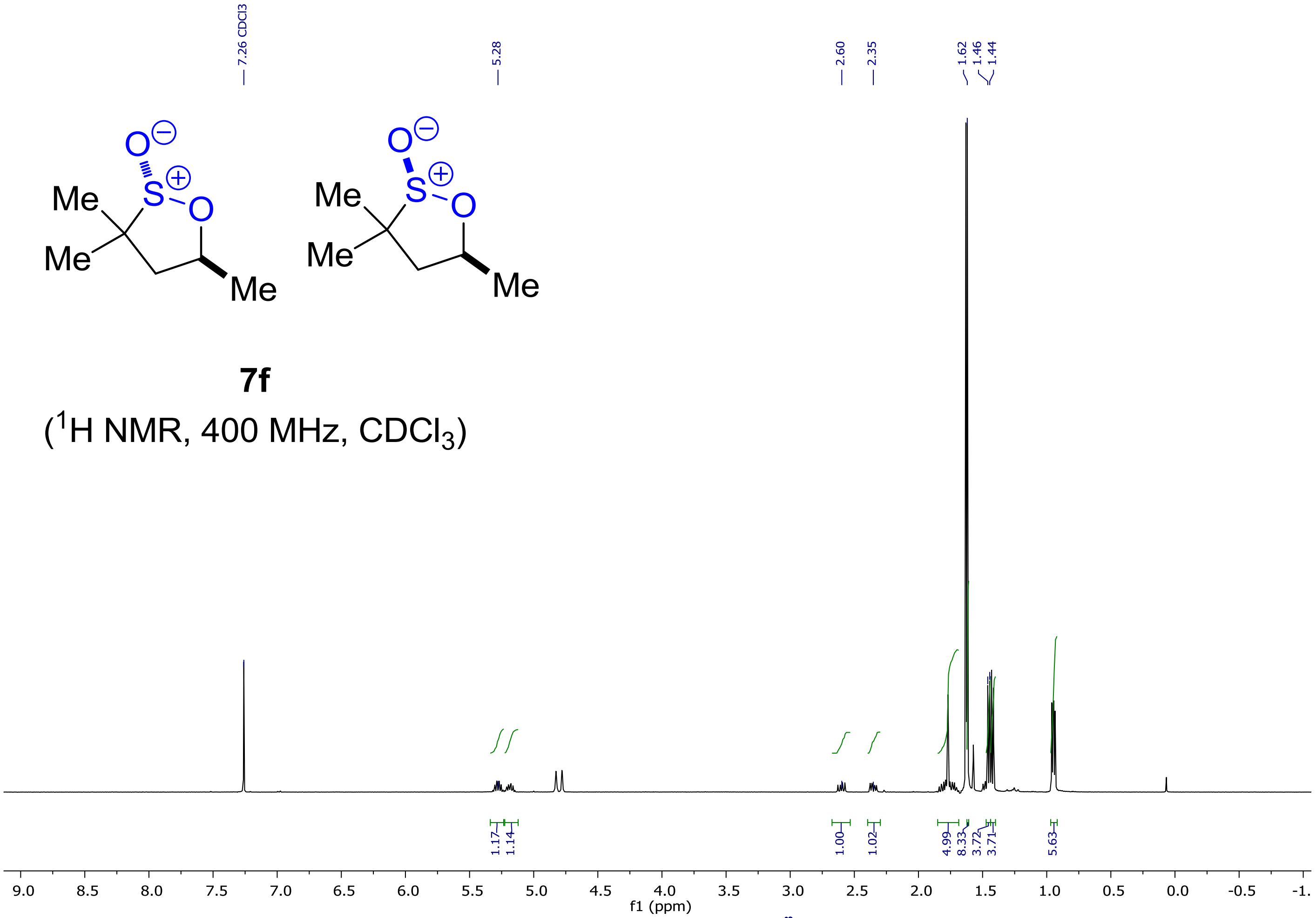

7f

(^{13}C NMR, 100 MHz, $CDCl_3$)

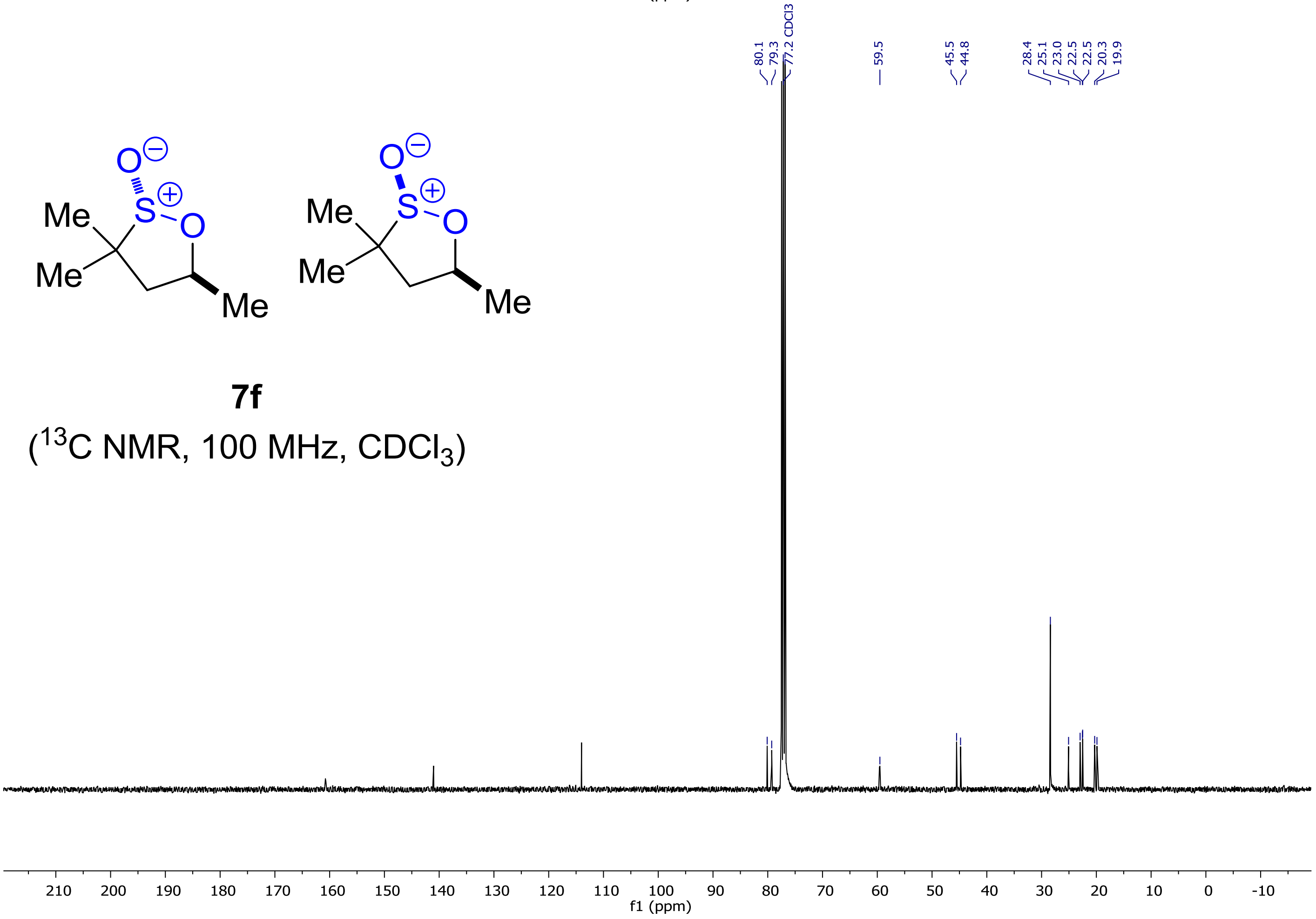

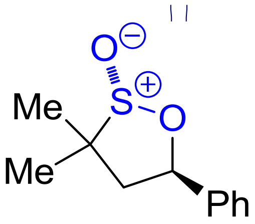

(2*R*,5*R*)-7g (*trans*, minor)

(^{1}H NMR, 400 MHz, $CDCl_3$)

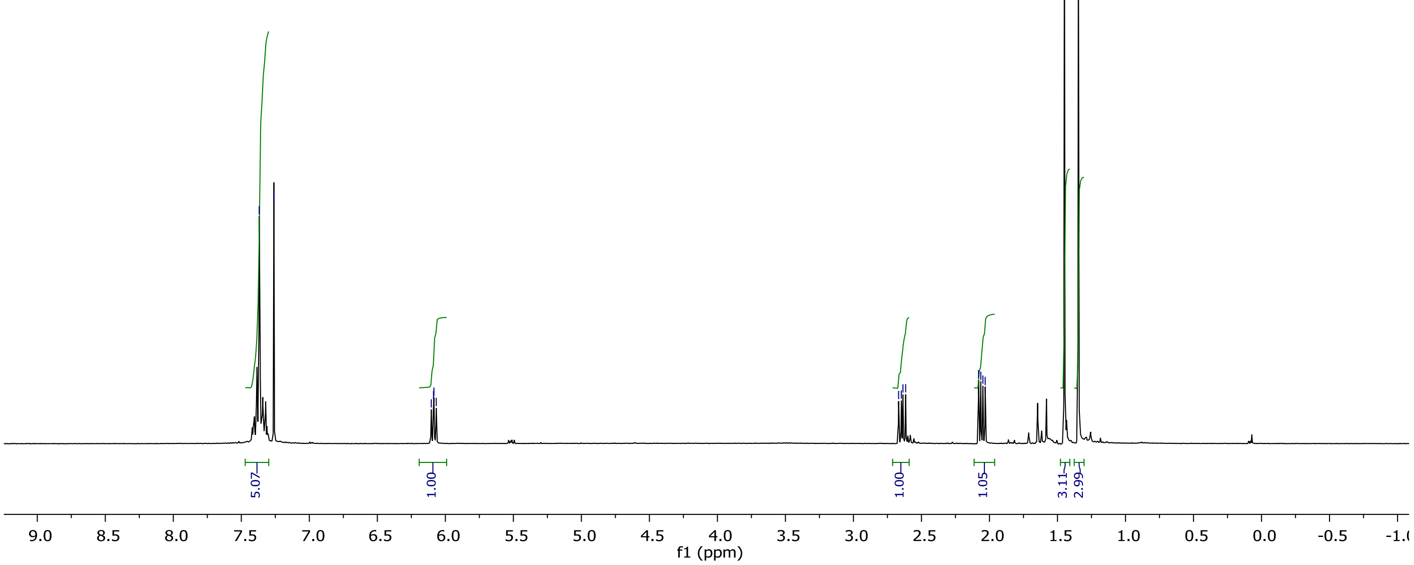

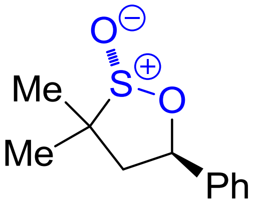

(2*R*,5*R*)-7g (*trans*, minor)

(^{13}C NMR, 100 MHz, $CDCl_3$)

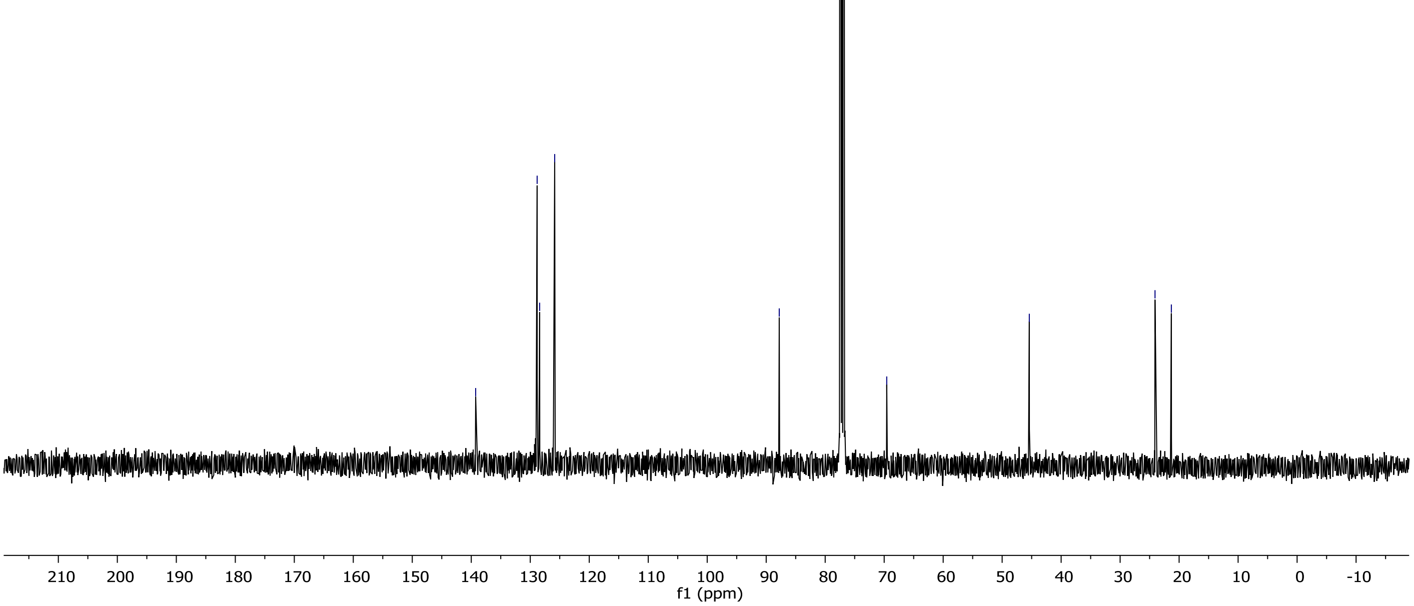

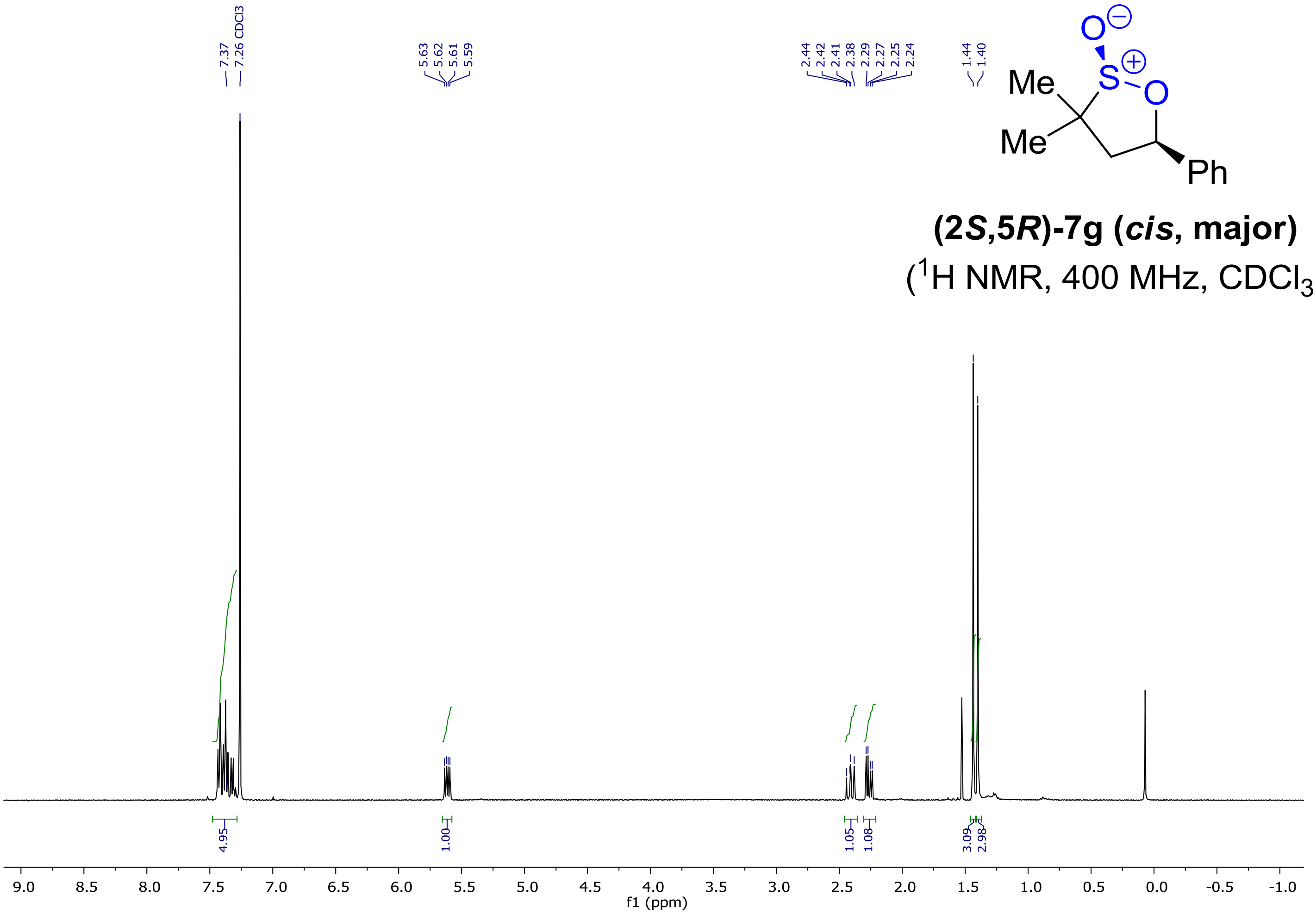

(2*S*,5*R*)-7g (*cis*, major)

(^{1}H NMR, 400 MHz, $CDCl_3$)

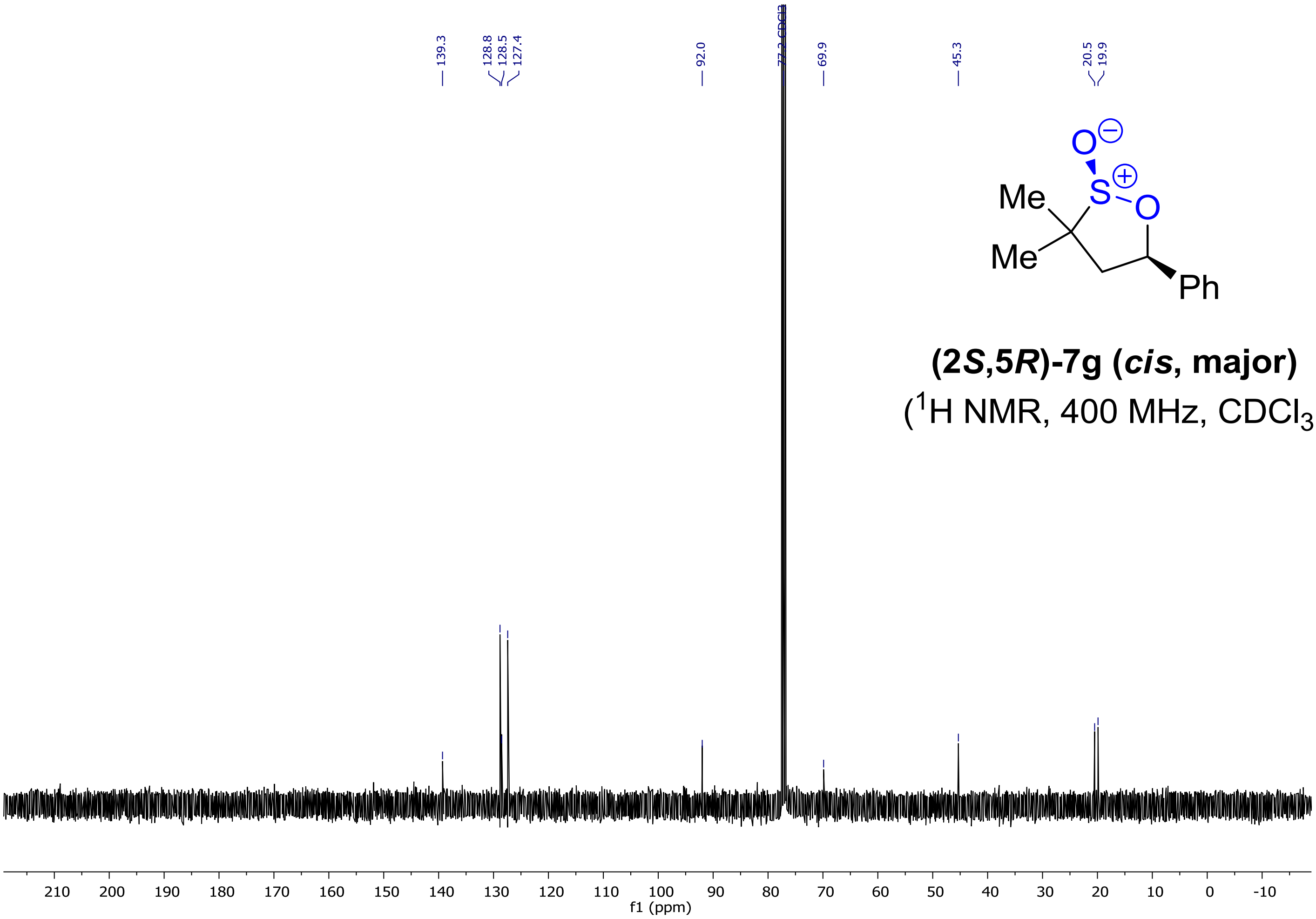

(2*S*,5*R*)-7g (*cis*, major)

(^{1}H NMR, 400 MHz, $CDCl_3$)

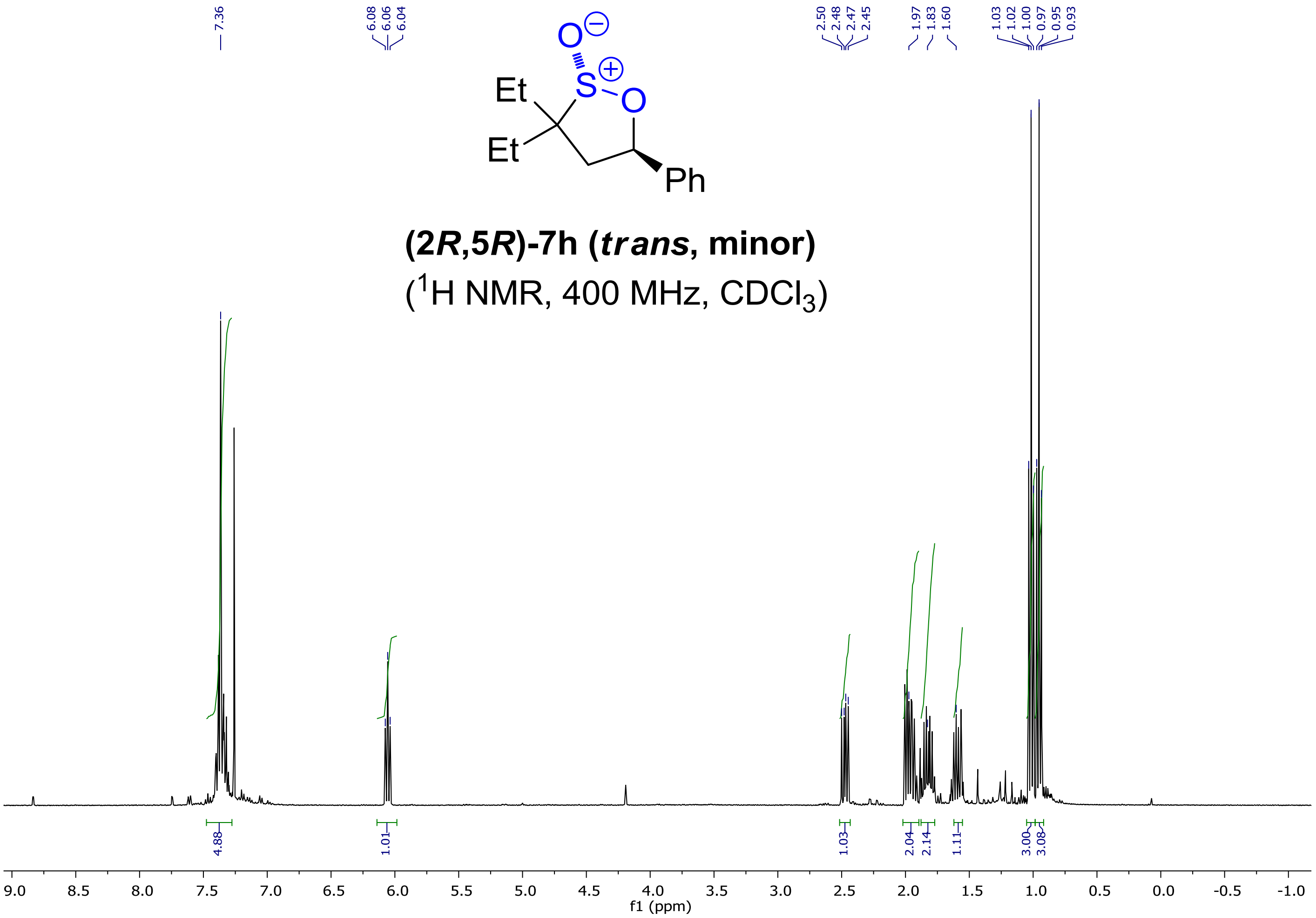
Et
Et
S
O
O
Ph
(2R,5R)-7h (trans, minor)
(1H NMR, 400 MHz, CDCl3)
7.36
6.08
6.06
6.04
2.50
2.48
2.47
2.45
1.97
1.83
1.60
1.03
1.02
1.00
0.97
0.95
0.93
4.88
1.01
1.03
2.04
2.14
1.11
3.00
3.08
f1 (ppm)

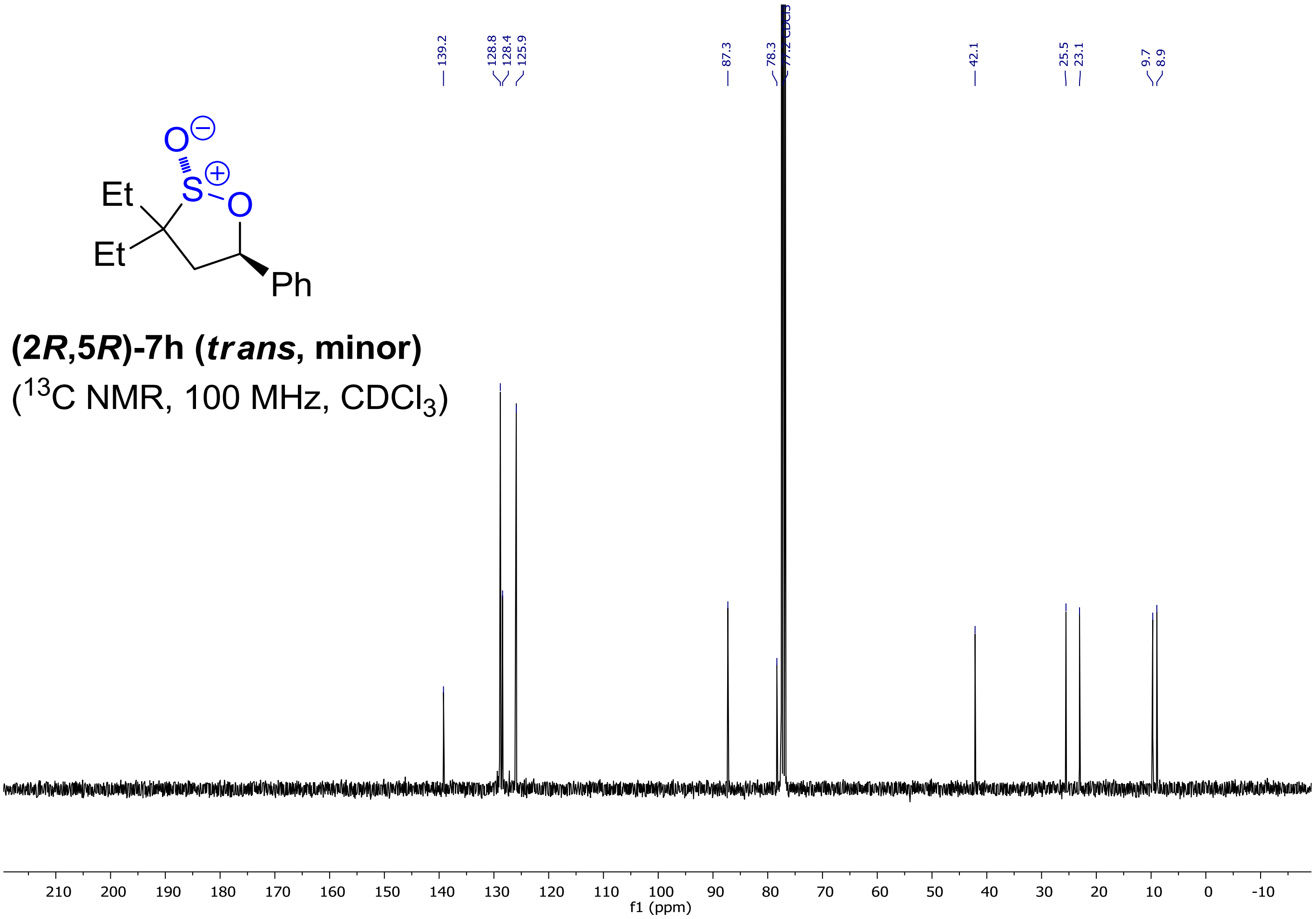
139.2
128.8
128.4
125.9
87.3
78.3
77.2 CDCl3
42.1
25.5
23.1
9.7
8.9
Et
Et
S
O
O
Ph
(2R,5R)-7h (trans, minor)
(13C NMR, 100 MHz, CDCl3)
f1 (ppm)

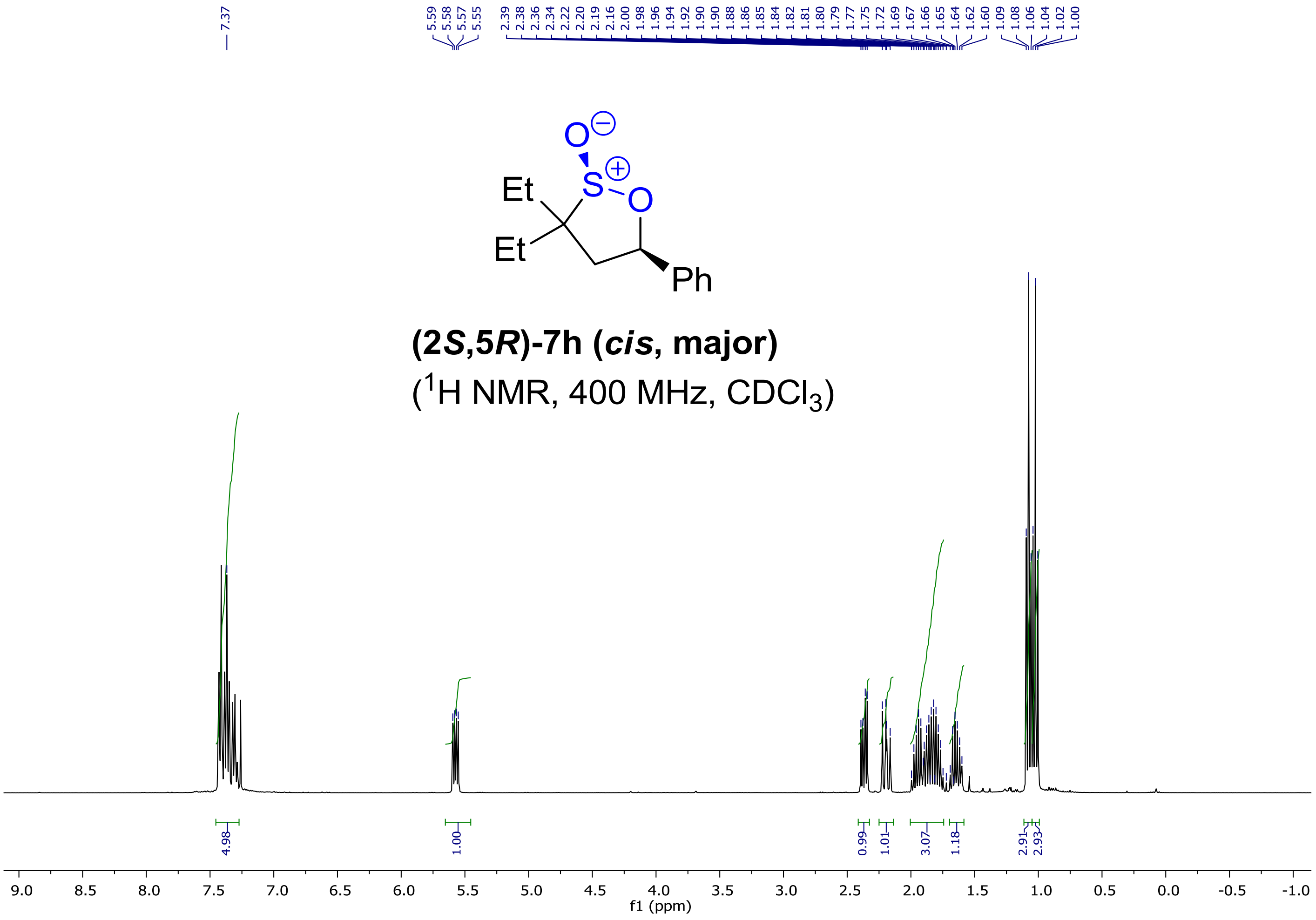
7.37
5.59 5.58 5.57 5.55
2.39 2.38 2.36 2.34 2.22 2.20 2.19 2.16 2.00 1.98 1.96 1.94 1.92 1.90 1.90 1.88 1.86 1.85 1.84 1.82 1.81 1.80 1.79 1.77 1.75 1.72 1.69 1.67 1.66 1.65 1.64 1.62 1.60 1.09 1.08 1.06 1.04 1.02 1.00
Et
Et
S
O
O
Ph
(2*S*,5*R*)-7h (*cis*, major)
(^{1}H NMR, 400 MHz, $CDCl_3$)
4.98
1.00
0.99
1.01
3.07
1.18
2.91
2.93
9.0 8.5 8.0 7.5 7.0 6.5 6.0 5.5 5.0 4.5 4.0 3.5 3.0 2.5 2.0 1.5 1.0 0.5 0.0 -0.5 -1.0
f1 (ppm)

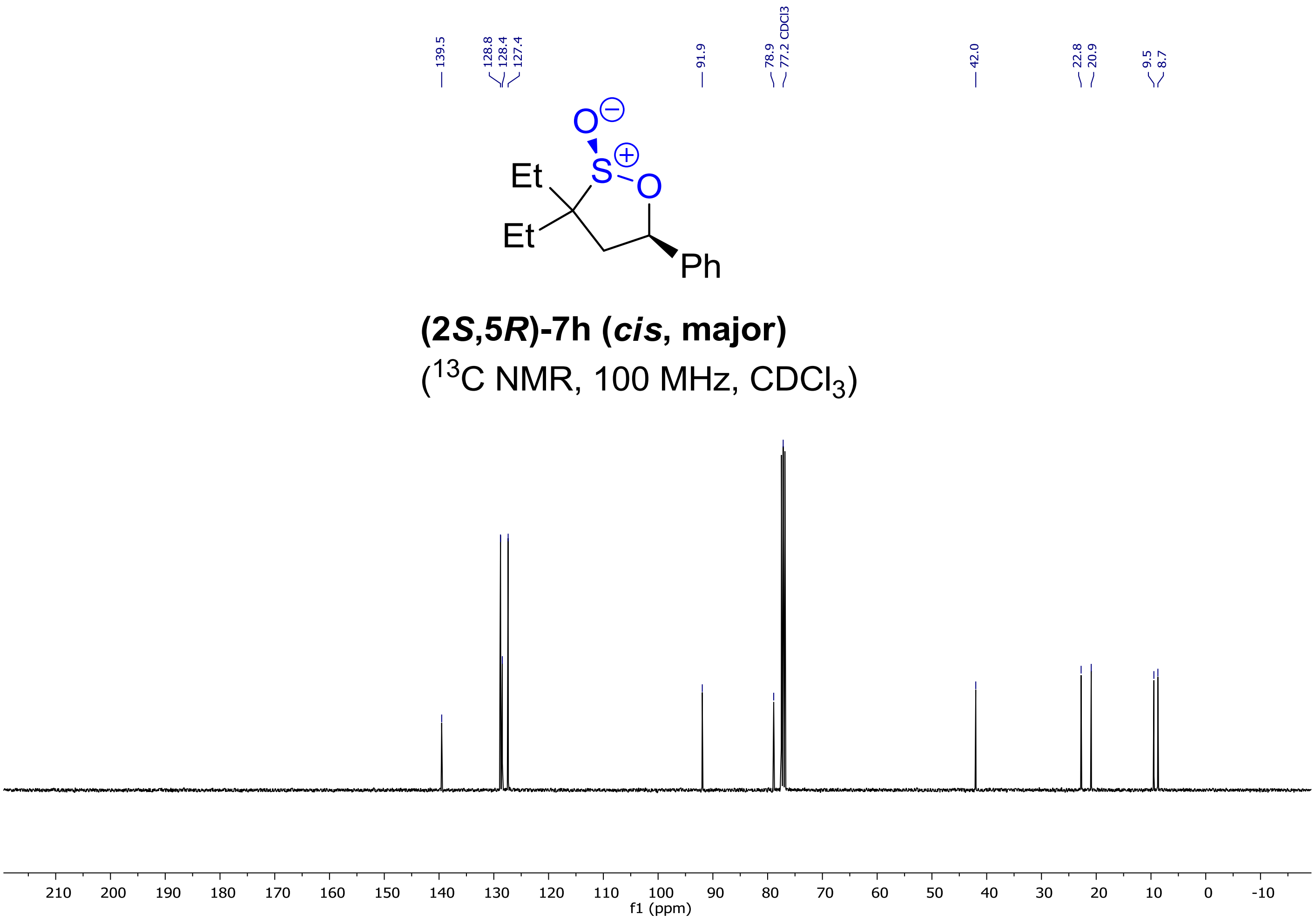
139.5
128.8 128.4 127.4
91.9
78.9 77.2 CDCl3
42.0
22.8 20.9
9.5 8.7
Et
Et
S
O
O
Ph
(2*S*,5*R*)-7h (*cis*, major)
(^{13}C NMR, 100 MHz, $CDCl_3$)
210 200 190 180 170 160 150 140 130 120 110 100 90 80 70 60 50 40 30 20 10 0 -10
f1 (ppm)

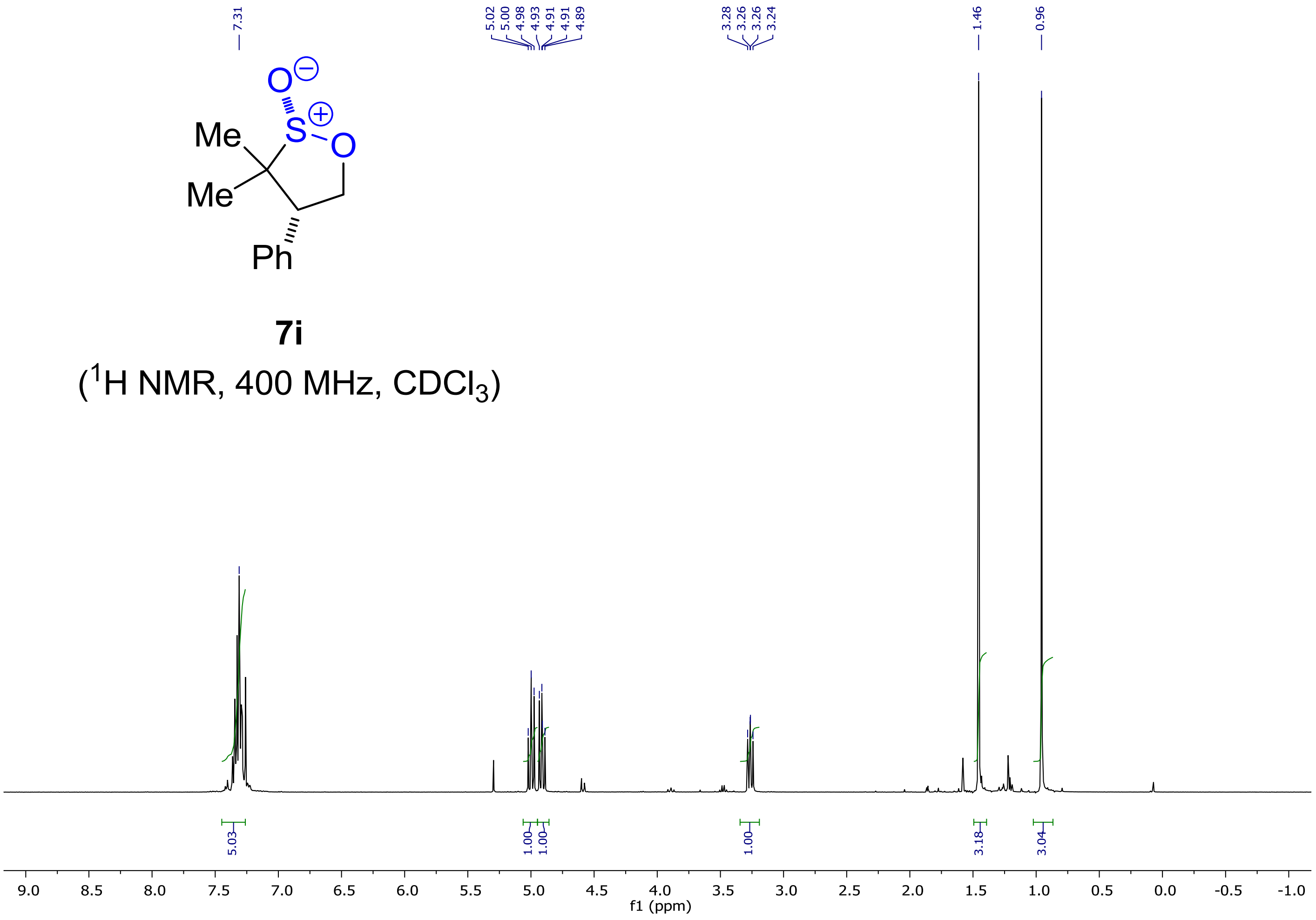
7.31
5.02
5.00
4.98
4.93
4.91
4.91
4.89
3.28
3.26
3.26
3.24
1.46
0.96
Me
Me
Ph
7i
(^{1}H NMR, 400 MHz, CDCl$_3$)
5.03
1.00
1.00
1.00
3.18
3.04
9.0 8.5 8.0 7.5 7.0 6.5 6.0 5.5 5.0 4.5 4.0 3.5 3.0 2.5 2.0 1.5 1.0 0.5 0.0 -0.5 -1.0
f1 (ppm)

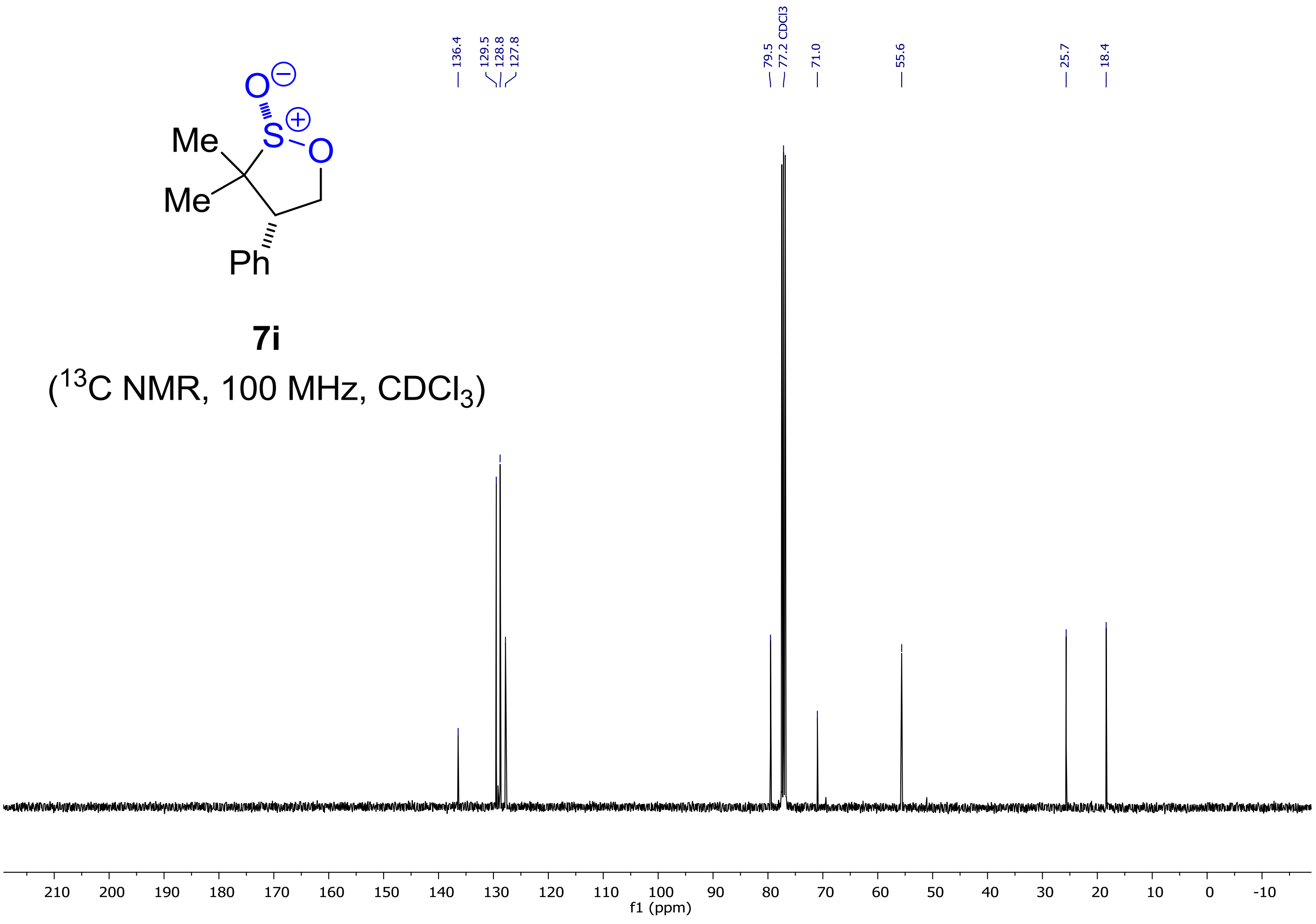
136.4
129.5
128.8
127.8
79.5
77.2 CDCl3
71.0
55.6
25.7
18.4
Me
Me
Ph
7i
(^{13}C NMR, 100 MHz, CDCl$_3$)
210 200 190 180 170 160 150 140 130 120 110 100 90 80 70 60 50 40 30 20 10 0 -10
f1 (ppm)

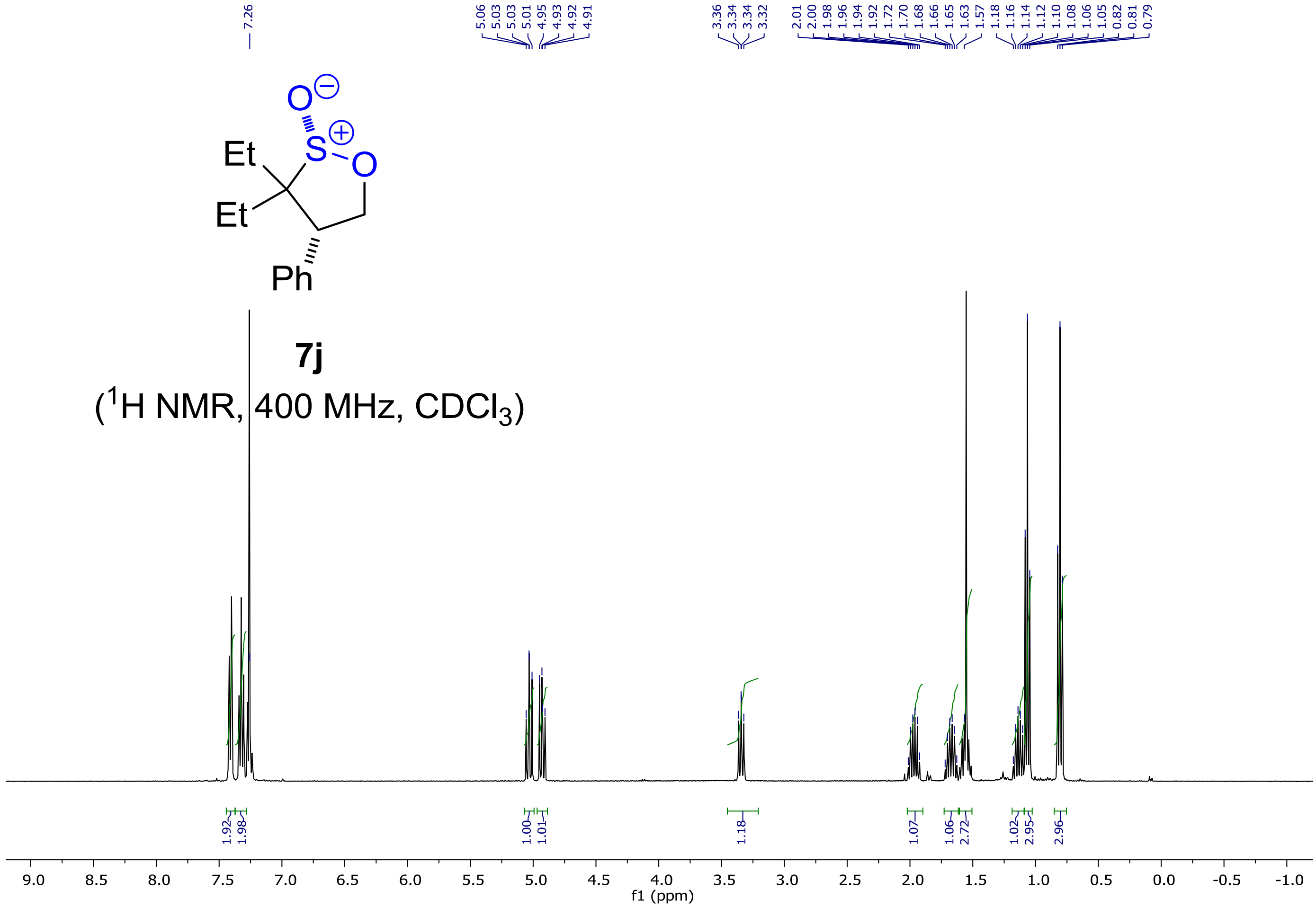

7j

(^{1}H NMR, 400 MHz, $CDCl_3$)

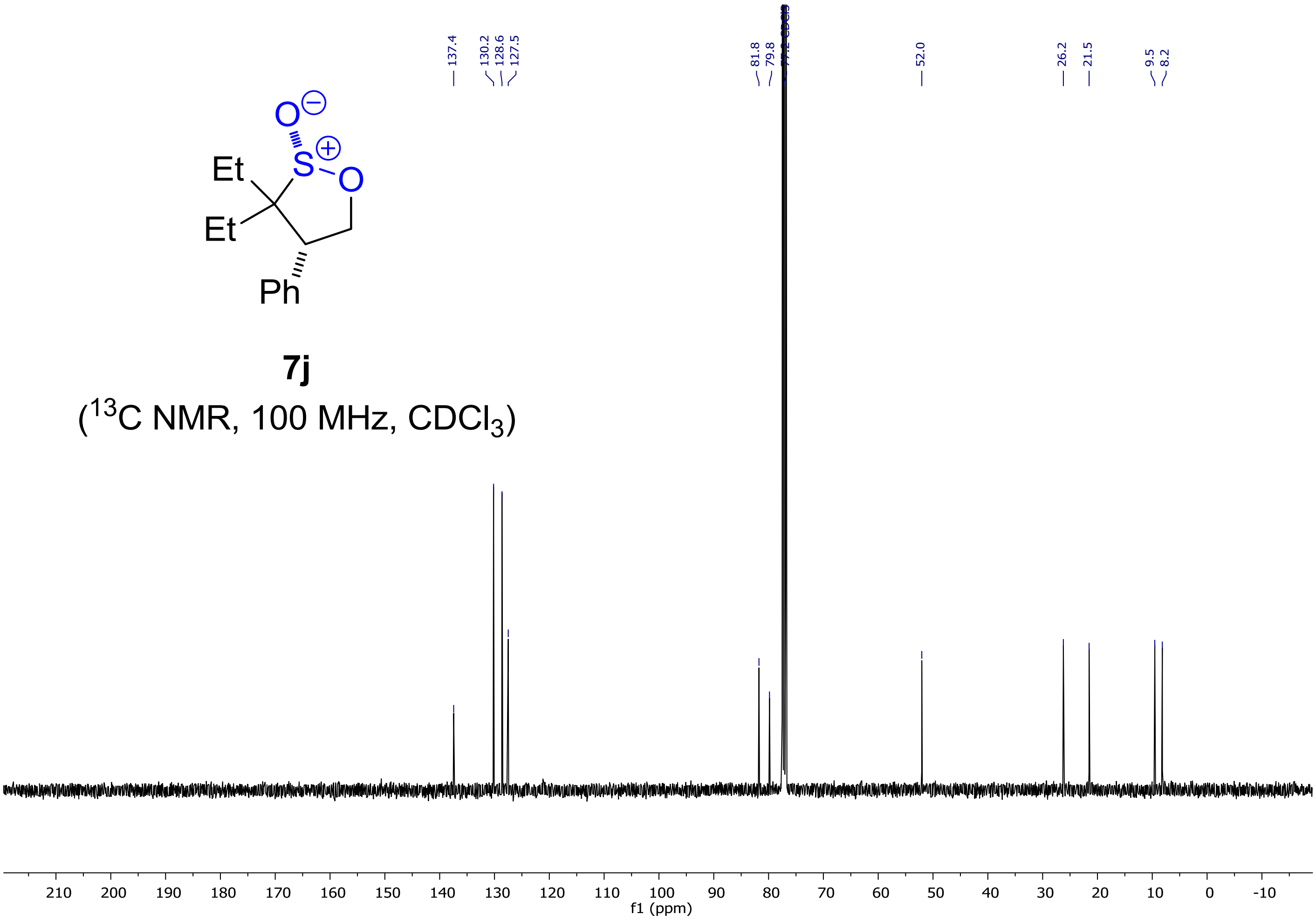

7j

(^{13}C NMR, 100 MHz, $CDCl_3$)

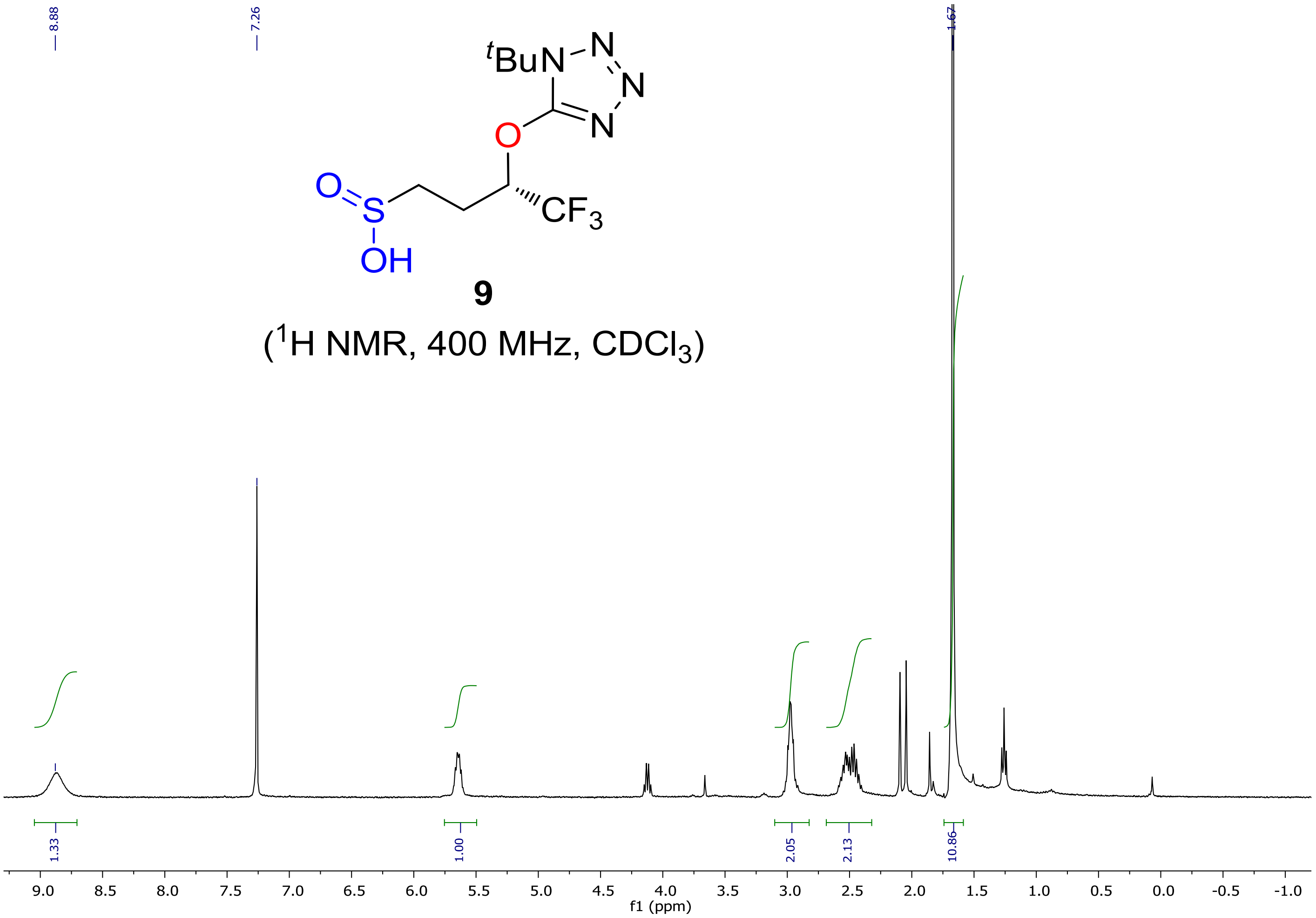

8.88
7.26
1.67
tBuN
N
N
N
O
O
S
OH
CF3
9
(1H NMR, 400 MHz, CDCl3)
1.33
1.00
2.05
2.13
10.86
9.0 8.5 8.0 7.5 7.0 6.5 6.0 5.5 5.0 4.5 4.0 3.5 3.0 2.5 2.0 1.5 1.0 0.5 0.0 -0.5 -1.0
f1 (ppm)

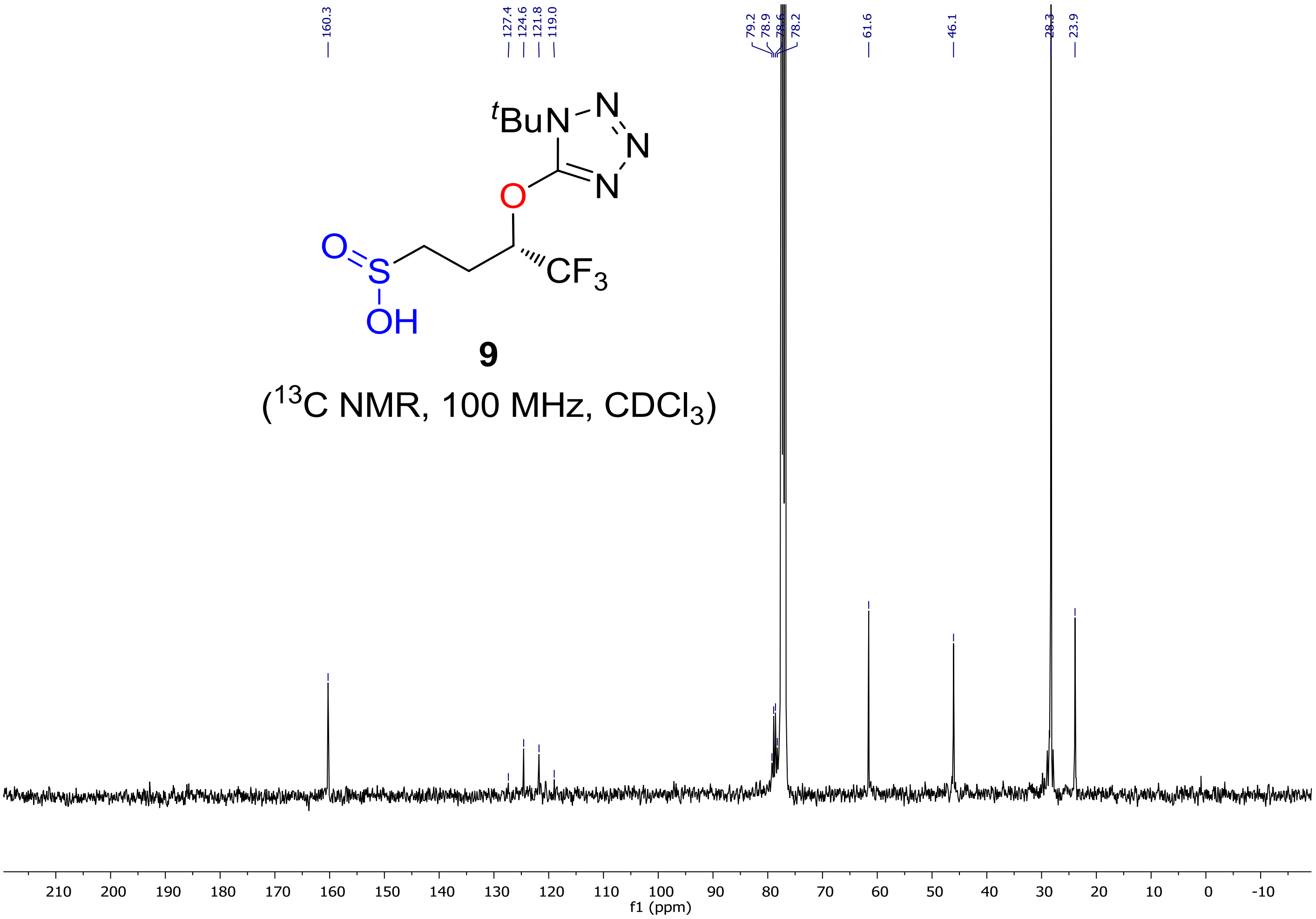

160.3
127.4
124.6
121.8
119.0
79.2
78.9
78.6
78.2
61.6
46.1
28.3
23.9
tBuN
N
N
N
O
O
S
OH
CF3
9
(13C NMR, 100 MHz, CDCl3)
210 200 190 180 170 160 150 140 130 120 110 100 90 80 70 60 50 40 30 20 10 0 -10
f1 (ppm)

11. ^{1}H and ^{13}C NMR data for reaction by-products

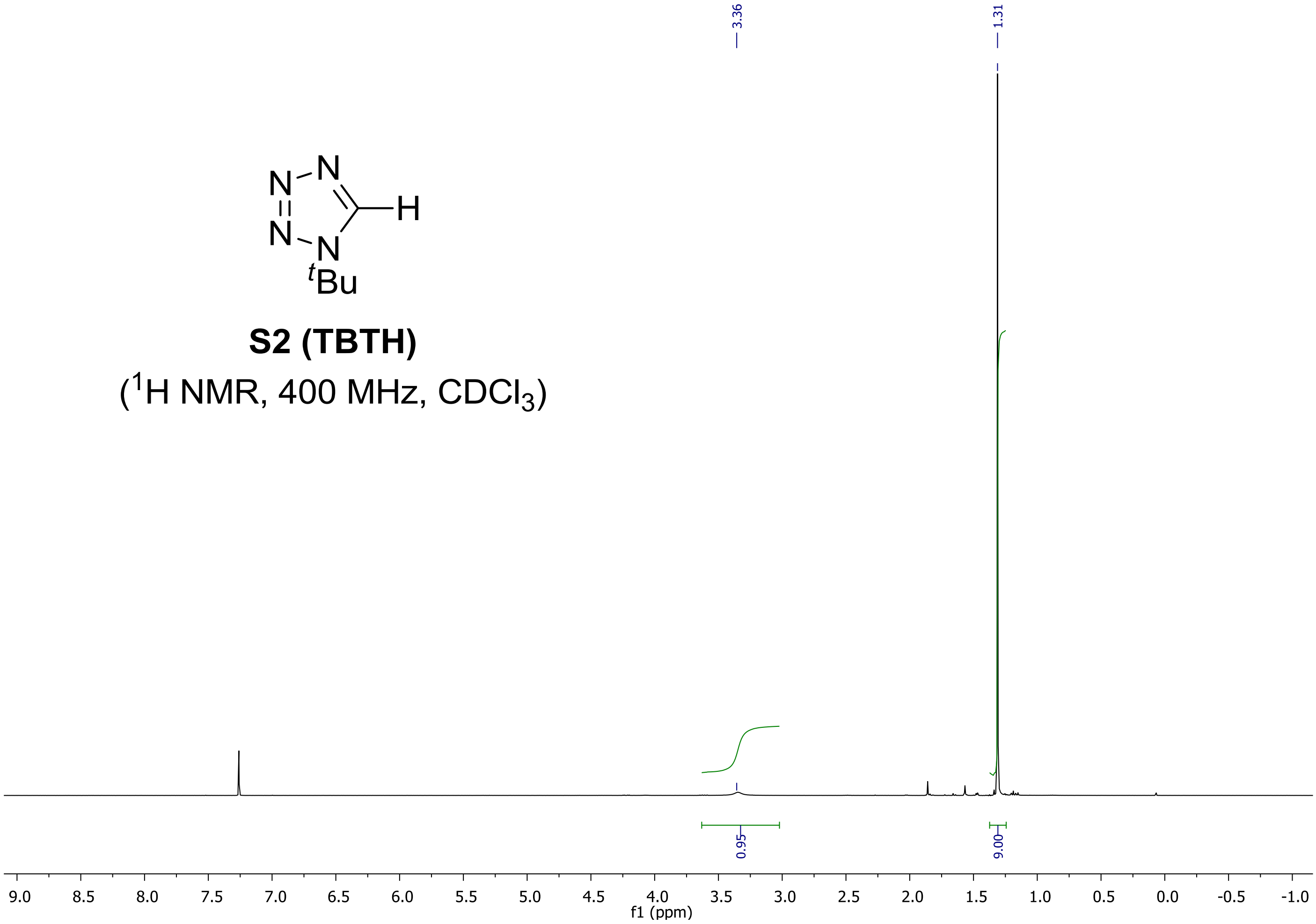

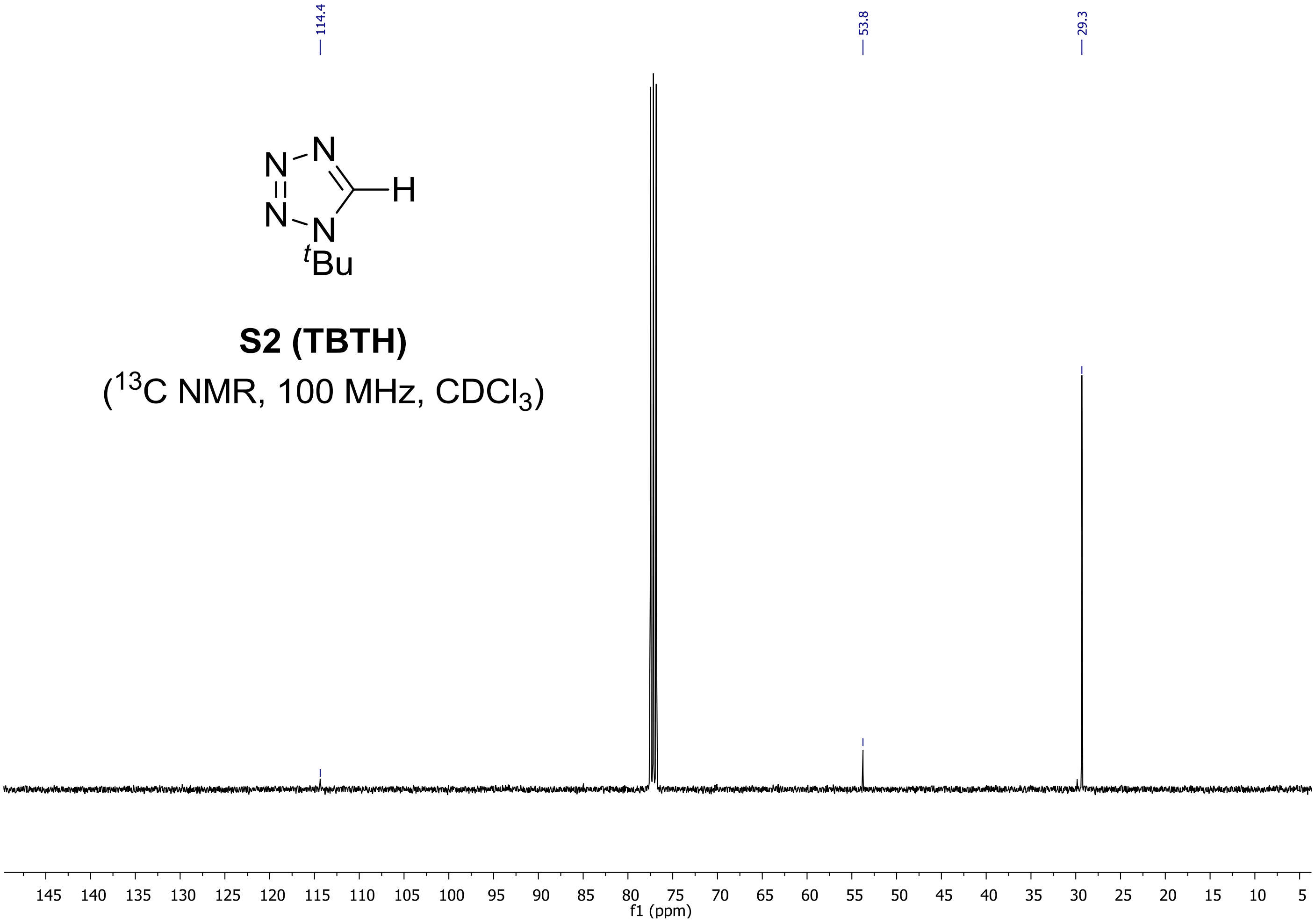

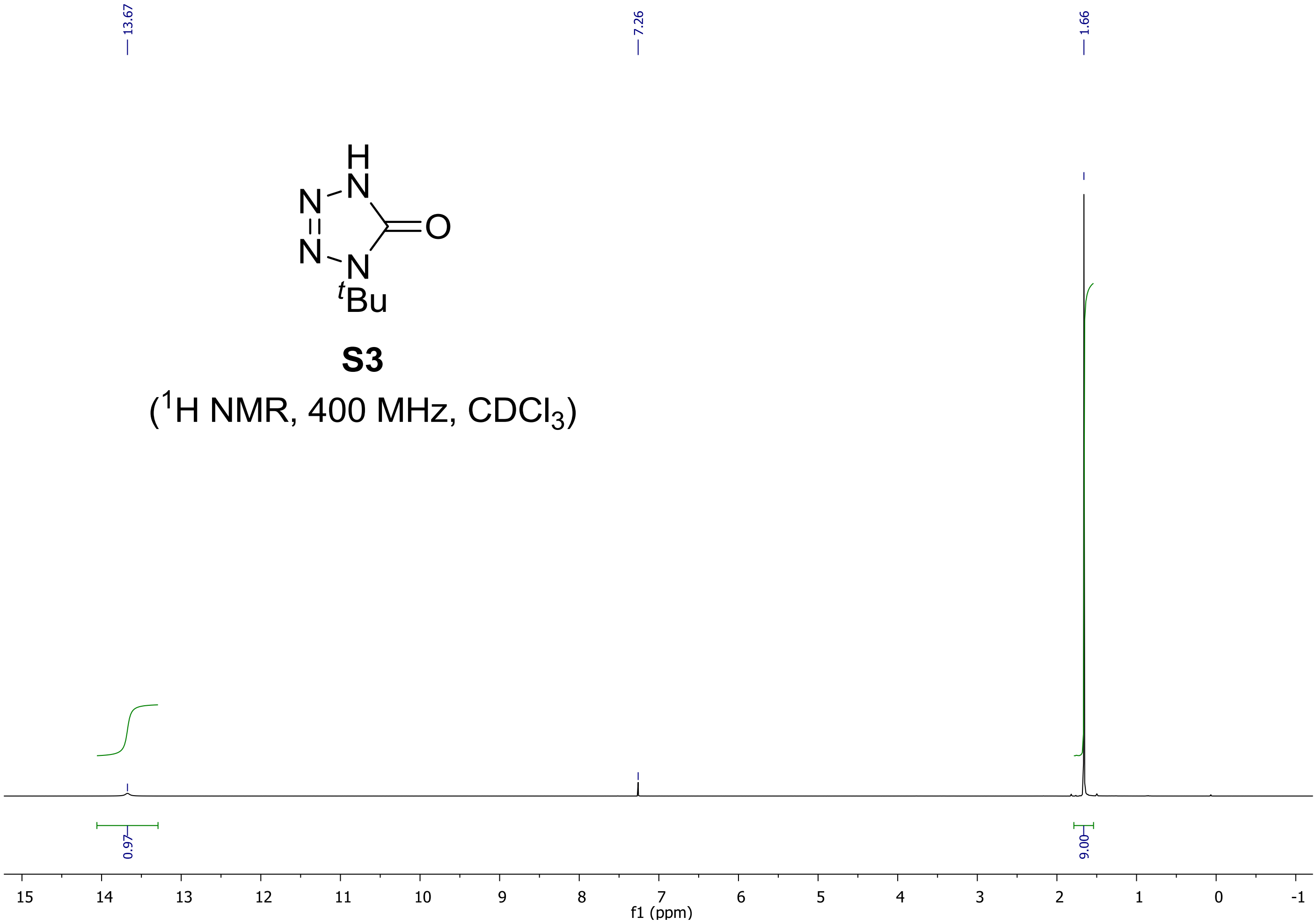
13.67
7.26
1.66
S3
(^{1}H NMR, 400 MHz, CDCl$_3$)
0.97
9.00
f1 (ppm)

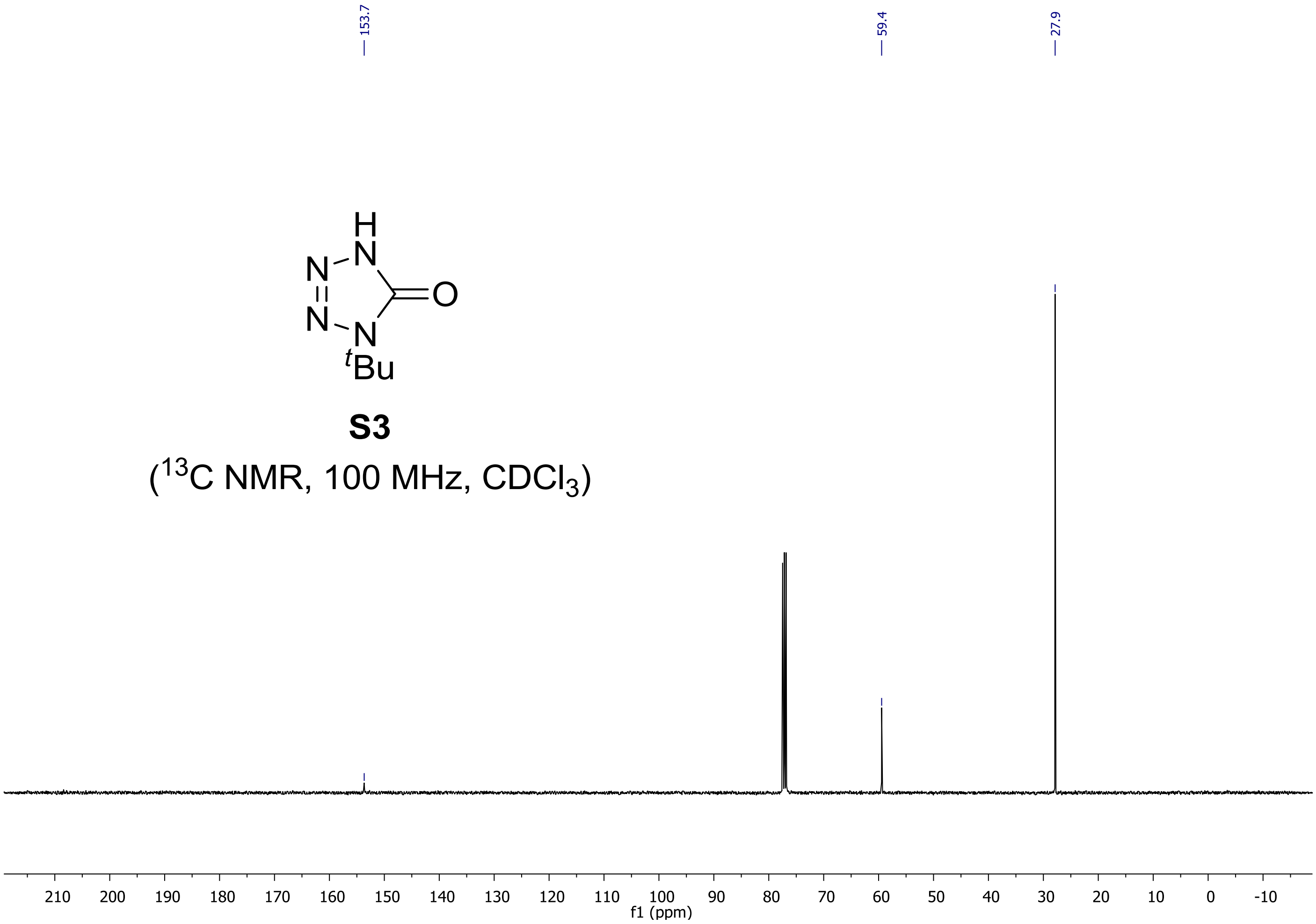
153.7
59.4
27.9
S3
(^{13}C NMR, 100 MHz, CDCl$_3$)
f1 (ppm)

12. X-ray data

Crystal data and structure refinement for Sultone **4e**

XDF
X-ray Diffraction Facility
School of Biological and Chemical Sciences • Centre for Materials Research • School of Engineering and Materials Sciences

Table 1. Sample and crystal data for 4e

**Identification code**	RU_tBu_Sultone_4R	
**Chemical formula**	$C_7H_{14}O_3S$	
**Formula weight**	178.24 g/mol	
**Temperature**	299(2) K	
**Wavelength**	0.71073 Å	
**Crystal size**	0.050 x 0.200 x 0.350 mm	
**Crystal system**	monoclinic	
**Space group**	C 1 2/c 1	
**Unit cell dimensions**	a = 6.8516(13) Å	α = 90°
	b = 9.7302(19) Å	β = 95.083(5)°
	c = 28.074(6) Å	γ = 90°
**Volume**	1864.3(6) $Å^3$	
**Z**	8	
**Density (calculated)**	1.270 g/cm^3	
**Absorption coefficient**	0.308 mm^{-1}	
**F(000)**	768	

Table 2. Data collection and structure refinement for 4e

**Theta range for data collection**	1.46 to 27.55°	
**Index ranges**	-8<=h<=8, -12<=k<=12, -36<=l<=29	
**Reflections collected**	10619	
**Independent reflections**	2142 [R(int) = 0.0699]	
**Max. and min. transmission**	0.9850 and 0.9000	
**Refinement method**	Full-matrix least-squares on F^2	
**Refinement program**	SHELXL-2014/6 (Sheldrick, 2014)	
**Function minimized**	Σ w(F_o^2 - F_c^2)^2	
**Data / restraints / parameters**	2142 / 0 / 133	
**Goodness-of-fit on F^2**	1.051	
**Δ/σ_{max}**	0.003	
**Final R indices**	1069 data; I>2σ(I)	R1 = 0.0755, wR2 = 0.1694
	all data	R1 = 0.1531, wR2 = 0.1977
**Weighting scheme**	w=1/[$\sigma^2(F_o^2)+(0.0647P)^2+3.6472P$]<br>where P=($F_o^2+2F_c^2$)/3	
**Largest diff. peak and hole**	0.231 and -0.303 $eÅ^{-3}$	
**R.M.S. deviation from mean**	0.050 $eÅ^{-3}$	

Ortep Plot of Sultone **4e** with ellipsoids shown at 30% probability.

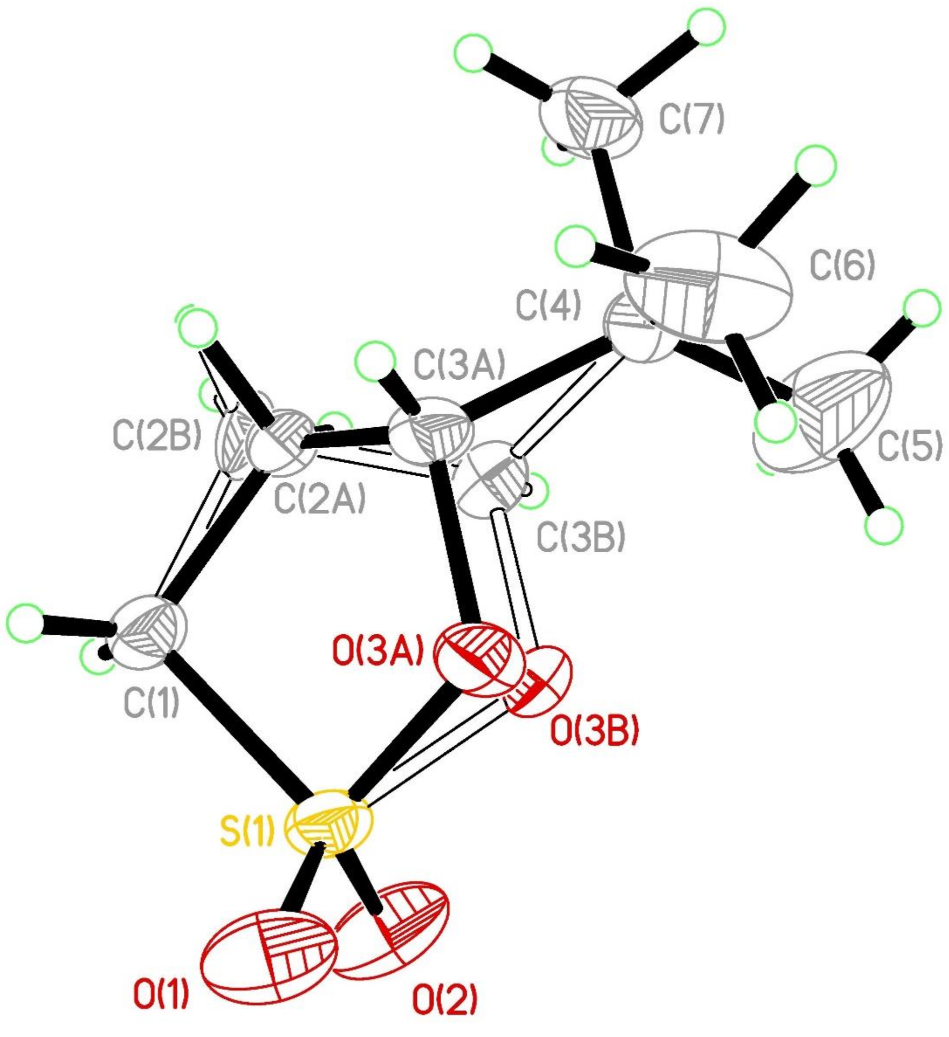

Crystal data and structure refinement for Sultone **4f**

UK National Crystallography Service

Table 1. Crystal data and structure refinement.

Identification code	**2014ncs0252 / enantiopure benzyl sulfone**	
Empirical formula	$C_{10}H_{12}O_3S$	
Formula weight	212.26	
Temperature	100(2) K	
Wavelength	0.71075 Å	
Crystal system	Monoclinic	
Space group	$P2_1$	
Unit cell dimensions	a = 10.7104(8) Å	α = 90°
	b = 8.8384(5) Å	β = 102.636(2)°
	c = 10.8634(8) Å	γ = 90°
Volume	1003.45(12) Å^3	
Z	4	
Density (calculated)	1.405 Mg / m^3	
Absorption coefficient	0.300 mm^{-1}	
F(000)	448	
Crystal	Blade; Colourless	
Crystal size	0.230 × 0.070 × 0.010 mm^3	
θ range for data collection	3.001 – 27.472°	
Index ranges	$-13 \le h \le 13$, $-11 \le k \le 11$, $-14 \le l \le 14$	
Reflections collected	18300	
Independent reflections	4180 [R_{int} = 0.0769]	
Completeness to θ = 25.242°	97.6 %	
Absorption correction	Semi–empirical from equivalents	
Max. and min. transmission	1.000 and 0.671	
Refinement method	Full-matrix least-squares on F^2	
Data / restraints / parameters	4180 / 1 / 253	
Goodness-of-fit on F^2	1.031	
Final R indices [$F^2 > 2\sigma(F^2)$]	$R1$ = 0.0471, $wR2$ = 0.1244	
R indices (all data)	$R1$ = 0.0510, $wR2$ = 0.1273	
Absolute structure parameter	–0.02(5)	
Extinction coefficient	n/a	
Largest diff. peak and hole	0.542 and –0.345 e Å^{-3}	

Diffractometer: *Rigaku AFC12* goniometer equipped with an enhanced sensitivity (HG) *Saturn724+* detector mounted at the window of an *FR-E+ SuperBright* molybdenum rotating anode generator with HF *Varimax* optics (100µm focus). **Cell determination and data collection**: *CrystalClear-SM Expert 3.1 b27* (Rigaku, 2013). **Data reduction, cell refinement and absorption correction**: *CrystalClear-SM Expert 3.1 b27* (Rigaku, 2012). **Structure solution**: *SUPERFLIP* (Palatinus, L. & Chapuis, G. (2007). J. Appl. Cryst. 40, 786-790.) **Structure refinement**: *SHELXL-2013* (G Sheldrick, G.M. (2008). Acta Cryst. A64, 112-122.). **Graphics:** *ORTEP3 for Windows* (L. J. Farrugia, J. Appl. Crystallogr. 1997, 30, 565

Ortep Plot of Sultone **4f** with ellipsoids shown at 30% probability.

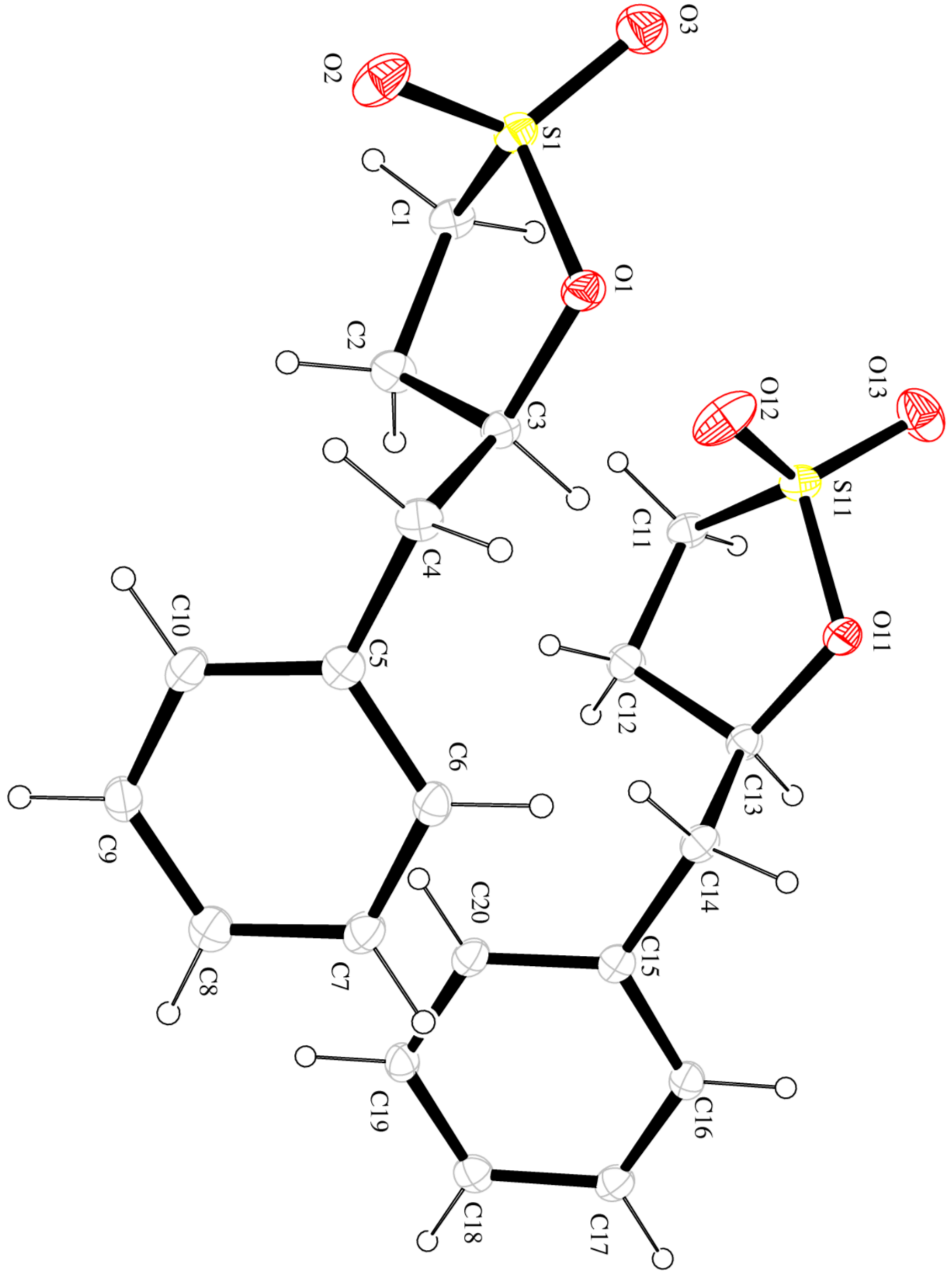

Crystal data and structure refinement for Sultine ***cis***-(±)-**7a**[18]

XDF
X-ray Diffraction Facility
School of Biological and Chemical Sciences • Centre for Materials Research • School of Engineering and Materials Sciences

Table 2. Sample and crystal data for **cis-7a**

Chemical formula	$C_9H_{10}O_2S$	
Formula weight	182.23 g/mol	
Temperature	100(2) K	
Wavelength	1.54178 Å	
Crystal size	0.080 x 0.100 x 0.150 mm	
Crystal habit	lustrous colourless Shard	
Crystal system	orthorhombic	
Space group	P 21 21 21	
Unit cell dimensions	a = 9.2275(3) Å	α = 90°
	b = 10.6619(4) Å	β = 90°
	c = 17.4548(6) Å	γ = 90°
Volume	1717.25(10) $Å^3$	
Z	8	
Density (calculated)	1.410 g/cm^3	
Absorption coefficient	2.979 mm^{-1}	
F(000)	768	

Table 3. Data collection and structure refinement for **cis-7a**

**Theta range for data collection**	4.86 to 65.15°	
**Index ranges**	$-10 \le h \le 9$, $-9 \le k \le 12$, $-16 \le l \le 20$	
**Reflections collected**	12368	
**Independent reflections**	2783 [R(int) = 0.0286]	
**Coverage of independent reflections**	99.1%	
**Absorption correction**	multi-scan	
**Max. and min. transmission**	0.7970 and 0.6640	
**Refinement method**	Full-matrix least-squares on F^2	
**Refinement program**	SHELXL-2014 (Sheldrick, 2014)	
**Function minimized**	$\Sigma w(F_o^2 - F_c^2)^2$	
**Data / restraints / parameters**	2783 / 0 / 217	
**Goodness-of-fit on F^2**	1.079	
**Δ/σ_{max}**	0.110	
**Final R indices**	2386 data; $I>2\sigma(I)$	R1 = 0.0316, wR2 = 0.0859
	all data	R1 = 0.0359, wR2 = 0.0901
**Weighting scheme**	$w=1/[\sigma^2(F_o^2)+(0.0517P)^2+0.5374P]$ where $P=(F_o^2+2F_c^2)/3$	
**Absolute structure parameter**	0.4(0)	
**Largest diff. peak and hole**	0.390 and -0.360 $eÅ^{-3}$	
**R.M.S. deviation from mean**	0.051 $eÅ^{-3}$	

(18) The racemate of this compound readily crystallised but the single enantiomer did not.

Ortep Plot of Sultine ***cis*-7a** with ellipsoids shown at 30% probability.

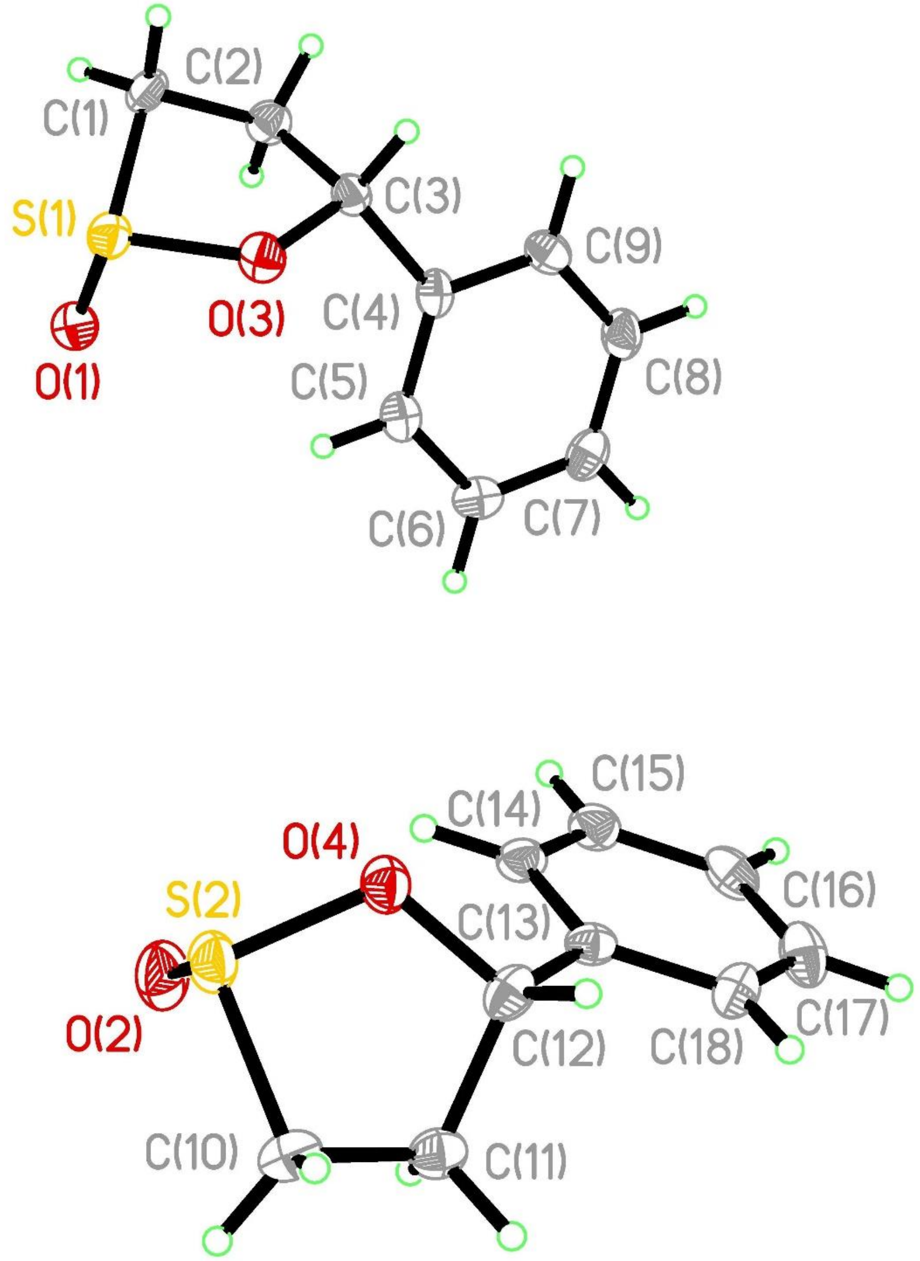

Crystal data and structure refinement for Sultine ***trans*-7g**

XDF
X-ray Diffraction Facility
School of Biological and Chemical Sciences • Centre for Materials Research • School of Engineering and Materials Sciences

Table 2. Sample and crystal data for **(S)** ***trans*-7g**

**Chemical formula**	$C_{11}H_{14}O_2S$	
**Formula weight**	210.28 g/mol	
**Temperature**	100(2) K	
**Wavelength**	1.54178 Å	
**Crystal size**	0.150 x 0.211 x 0.390 mm	
**Crystal habit**	translucent colourless shard	
**Crystal system**	monoclinic	
**Space group**	P 1 21 1	
**Unit cell dimensions**	a = 5.8160(3) Å	
	b = 11.2350(6) Å	α = 90°
	c = 8.2094(5) Å	β = 95.669(2)°
**Volume**	533.80(5) $Å^3$	γ = 90°
**Z**	2	
**Density (calculated)**	1.308 g/cm^3	
**Absorption coefficient**	2.464 mm^{-1}	
**F(000)**	224	

Table 3. Data collection and structure refinement for (S) *trans*-7g

**Theta range for data collection**	5.42 to 66.69°	
**Index ranges**	-6<=h<=6, -13<=k<=13, -9<=l<=9	
**Reflections collected**	6808	
**Independent reflections**	1833 [R(int) = 0.0405]	
**Coverage of independent reflections**	99.7%	
**Absorption correction**	multi-scan	
**Max. and min. transmission**	0.7090 and 0.4470	
**Refinement method**	Full-matrix least-squares on F^2	
**Refinement program**	SHELXL-2014/7 (Sheldrick, 2014)	
**Function minimized**	$\Sigma w(F_o^2 - F_c^2)^2$	
**Data / restraints / parameters**	1833 / 1 / 129	
**Goodness-of-fit on F^2**	1.061	
**Final R indices**	1820 data; I>2σ(I)	R1 = 0.0288, wR2 = 0.0750
	all data	R1 = 0.0290, wR2 = 0.0751
**Weighting scheme**	$w=1/[\sigma^2(F_o^2)+(0.0385P)^2+0.1449P]$ where $P=(F_o^2+2F_c^2)/3$	
**Absolute structure parameter**	0.1(0)	
**Largest diff. peak and hole**	0.492 and -0.219 $eÅ^{-3}$	
**R.M.S. deviation from mean**	0.046 $eÅ^{-3}$	

ORTEP Plot of Sulti

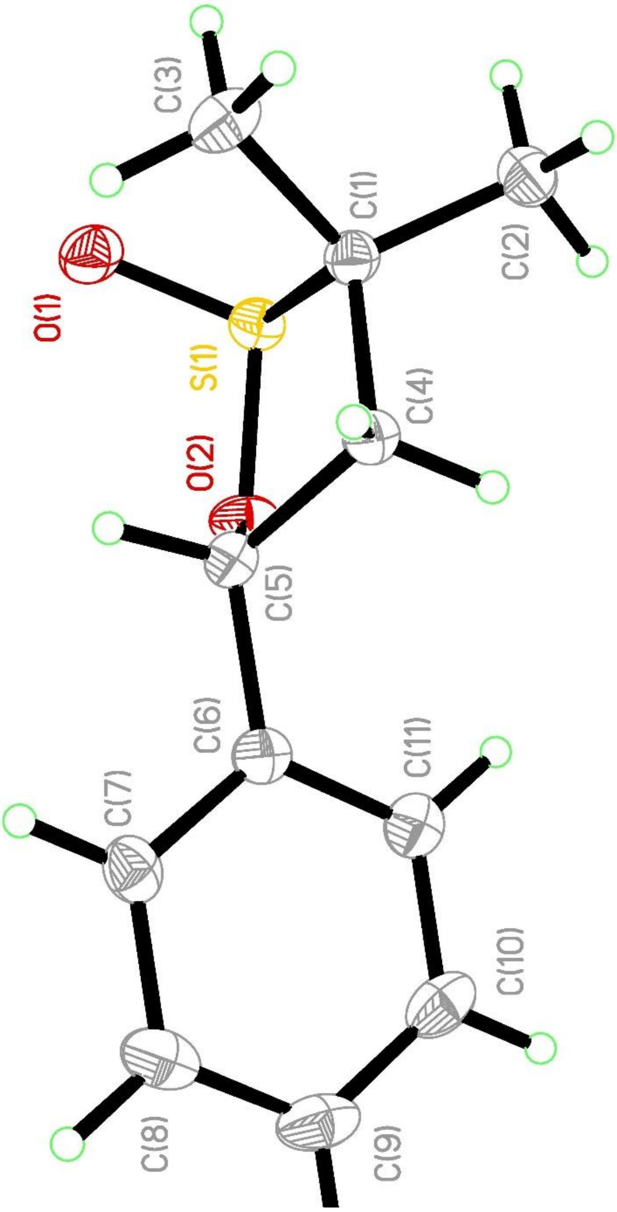

Crystal data and structure refinement for Sultine ***cis*-7g**

XDF
X-ray Diffraction Facility
School of Biological and Chemical Sciences • Centre for Materials Research • School of Engineering and Materials Sciences

**Table 2. Sample and crystal data for (S) *cis*-7g**		
**Chemical formula**	$C_{11}H_{14}O_2S$	
**Formula weight**	210.28 g/mol	
**Temperature**	100(2) K	
**Wavelength**	1.54178 Å	
**Crystal size**	0.040 x 0.060 x 0.200 mm	
**Crystal habit**	translucent colourless shard	
**Crystal system**	monoclinic	
**Space group**	P 1 21 1	
**Unit cell dimensions**	a = 11.7845(5) Å	
	b = 7.6325(4) Å	α = 90°
	c = 12.2546(5) Å	β = 92.284(2)°
**Volume**	1101.37(9) $Å^3$	γ = 90°
**Z**	4	
**Density (calculated)**	1.268 g/cm^3	
**Absorption coefficient**	2.388 mm^{-1}	
**F(000)**	448	

**Table 3. Data collection and structure refinement for (S) *cis*-7g**		
**Theta range for data collection**	3.61 to 65.24°	
**Index ranges**	-13<=h<=13, -8<=k<=8, -14<=l<=14	
**Reflections collected**	10198	
**Independent reflections**	3626 [R(int) = 0.0390]	
**Coverage of independent reflections**	99.0%	
**Absorption correction**	multi-scan	
**Max. and min. transmission**	0.9110 and 0.6470	
**Refinement method**	Full-matrix least-squares on F^2	
**Refinement program**	SHELXL-2014/7 (Sheldrick, 2014)	
**Function minimized**	$\Sigma w(F_o^2 - F_c^2)^2$	
**Data / restraints / parameters**	3626 / 1 / 257	
**Goodness-of-fit on F^2**	1.157	
**Δ/σ_{max}**	0.001	
**Final R indices**	3091 data; I>2σ(I)	R1 = 0.0623, wR2 = 0.1490
	all data	R1 = 0.0696, wR2 = 0.1547
**Weighting scheme**	$w=1/[\sigma^2(F_o^2)+(0.0428P)^2+1.5414P]$ where $P=(F_o^2+2F_c^2)/3$	
**Absolute structure parameter**	0.1(0)	
**Largest diff. peak and hole**	0.390 and -0.372 $eÅ^{-3}$	
**R.M.S. deviation from mean**	0.067 $eÅ^{-3}$	

ORTEP Plot of Sultine ***cis*-7g** with ellipsoids shown at 30% probability.

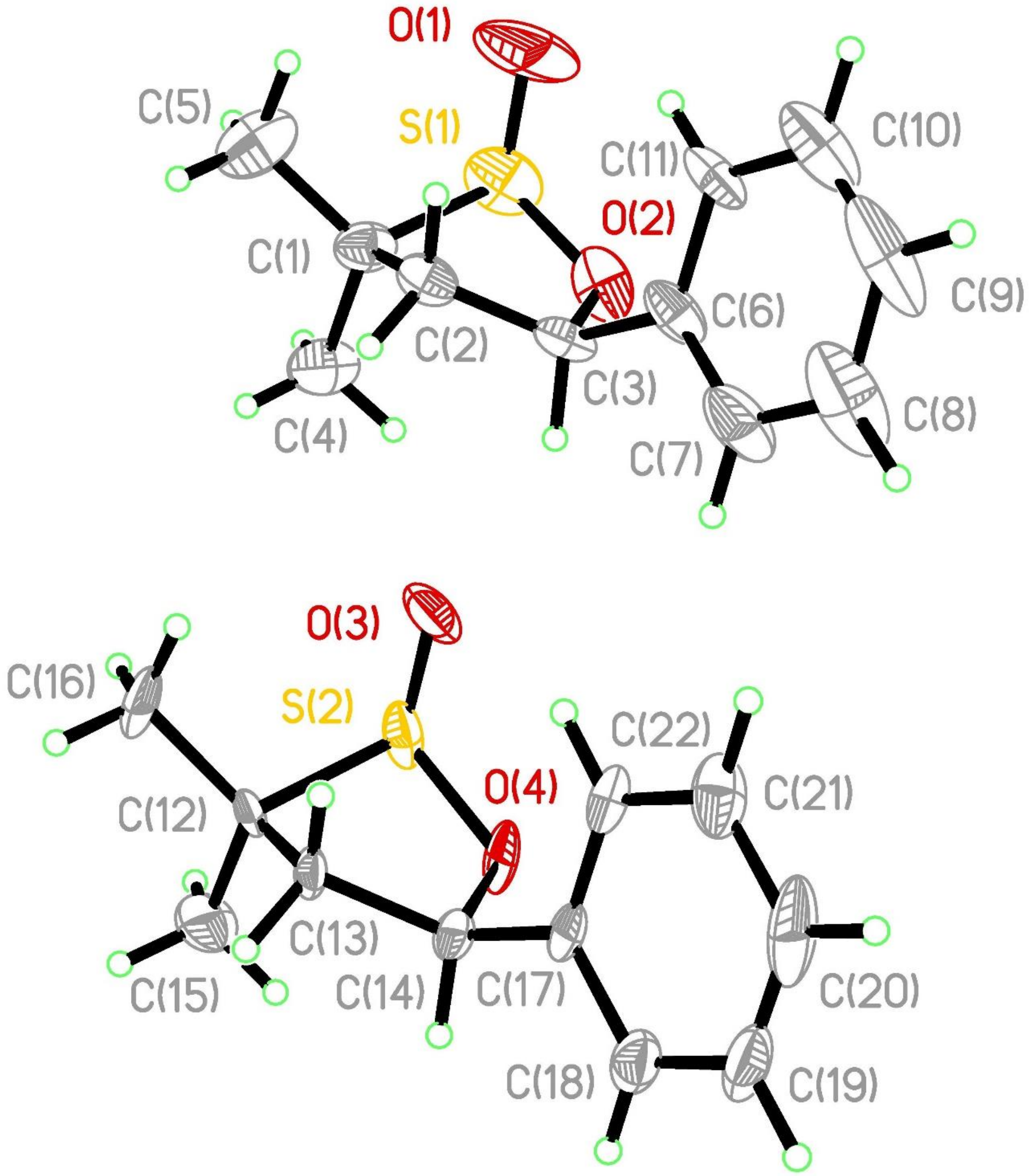

Crystal data and structure refinement for Sultine ***cis*-7i**

XDF
X-ray Diffraction Facility
School of Biological and Chemical Sciences • Centre for Materials Research • School of Engineering and Materials Sciences

Table 2. Sample and crystal data for Sultine ***cis*-7i**

**Chemical formula**	$C_{11}H_{14}O_2S$	
**Formula weight**	210.28 g/mol	
**Temperature**	100(2) K	
**Wavelength**	0.71073 Å	
**Crystal size**	0.120 x 0.170 x 0.380 mm	
**Crystal habit**	translucent colourless plate	
**Crystal system**	orthorhombic	
**Space group**	P 21 21 21	
**Unit cell dimensions**	a = 6.0280(2) Å	
	b = 10.4941(4) Å	α = 90°
	c = 16.4806(6) Å	β = 90°
**Volume**	1042.54(6) $Å^3$	γ = 90°
**Z**	4	
**Density (calculated)**	1.340 g/cm^3	
**Absorption coefficient**	0.281 mm^{-1}	
**F(000)**	448	

Table 3. Data collection and structure refinement for CB_GS_BetaPhSultine.

**Theta range for data collection**	2.30 to 28.29°	
**Index ranges**	-6<=h<=8, -13<=k<=11, -21<=l<=20	
**Reflections collected**	5834	
**Independent reflections**	2539 [R(int) = 0.0165]	
**Coverage of independent reflections**	99.7%	
**Absorption correction**	multi-scan	
**Max. and min. transmission**	0.9670 and 0.9010	
**Refinement method**	Full-matrix least-squares on F^2	
**Refinement program**	SHELXL-2014/7 (Sheldrick, 2014)	
**Function minimized**	$\Sigma\ w(F_o^2 - F_c^2)^2$	
**Data / restraints / parameters**	2539 / 0 / 129	
**Goodness-of-fit on F^2**	1.010	
**Final R indices**	2393 data; I>2σ(I)	R1 = 0.0247, wR2 = 0.0611
	all data	R1 = 0.0270, wR2 = 0.0624
**Weighting scheme**	$w=1/[\sigma^2(F_o^2)+(0.0337P)^2+0.2114P]$ where $P=(F_o^2+2F_c^2)/3$	
**Absolute structure parameter**	-0.0(0)	
**Largest diff. peak and hole**	0.258 and -0.200 $eÅ^{-3}$	
**R.M.S. deviation from mean**	0.039 $eÅ^{-3}$	

ORTEP Plot of Sultine ***cis*-7i** with ellipsoids shown at 30% probability.

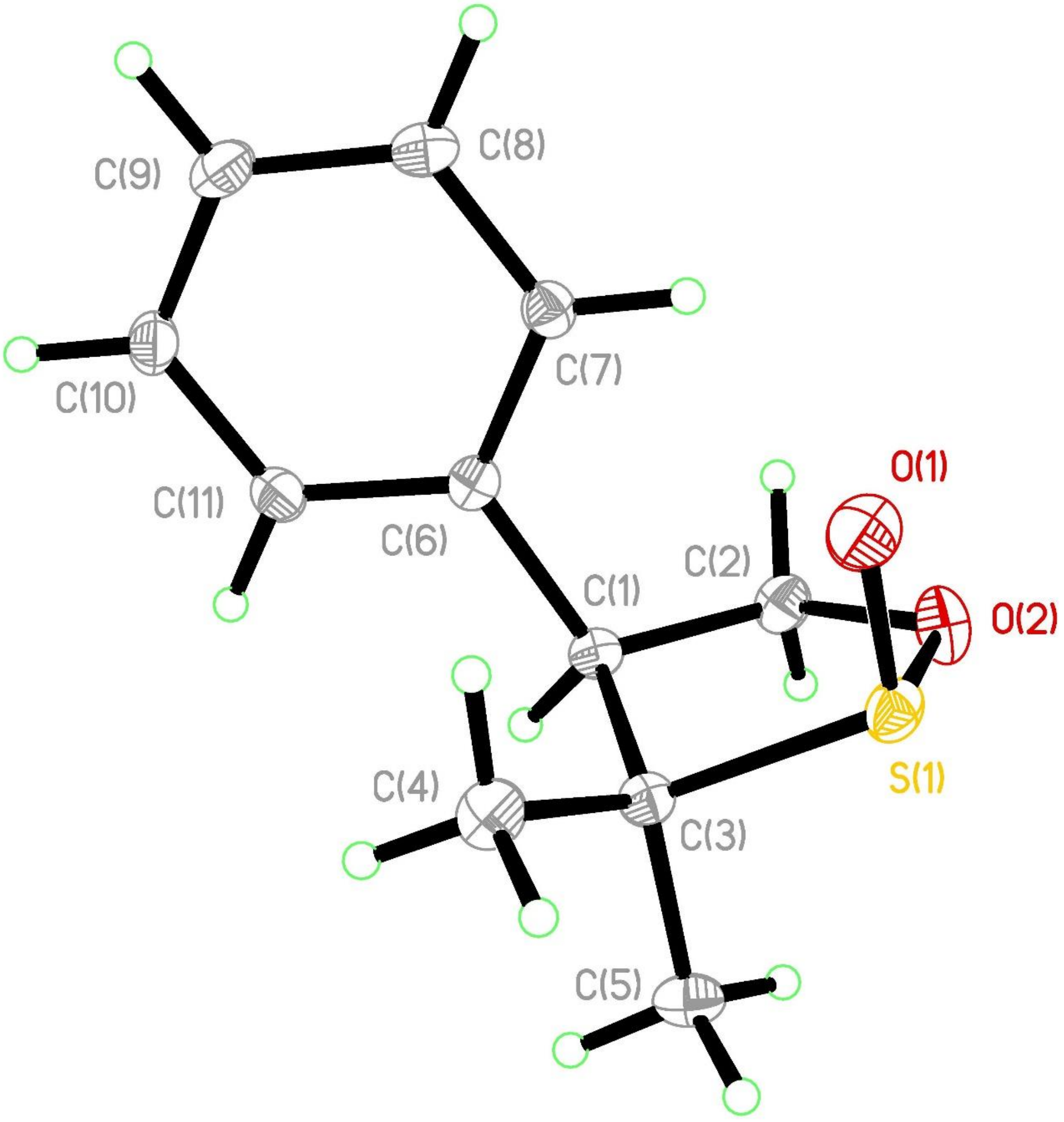

The following data is available from the Cambridge Crystallographic Database:

CCDC 1422271 – **4e** (tert-butyl sultone)

CCDC 1422272 –(±)- **4f** (benzyl sultones)

CCDC 1422273 – (*S*)-**4f** (enantiopure benzyl sultone)

CCDC 1422269 – (±)-**7a** (*cis*-phenyl sultine)

CCDC 1422267 – (*S*)-*trans* **7g** (trans-dimeethyl)

CCDC 1422268 - (*S*)-*cis* **7g** (cis-dimethyl)

CCDC 1422266 – *cis*-**7i** (beta phenyl)
